# ITS secondary structure reconstruction to resolve taxonomy and phylogeny of the *Betula* L. genus

**DOI:** 10.7717/peerj.10889

**Published:** 2021-03-23

**Authors:** Andrii S. Tarieiev, Oliver Gailing, Konstantin V. Krutovsky

**Affiliations:** 1 Department of Forest Genetics and Forest Tree Breeding, Georg-August University of Göttingen, Göttingen, Germany; 2 Center for Integrated Breeding Research, Georg-August University of Göttingen, Göttingen, Germany; 3 Laboratory of Forest Genomics, Genome Research and Education Center, Institute of Fundamental Biology and Biotechnology, Siberian Federal University, Krasnoyarsk, Russia; 4 Laboratory of Population Genetics, N.I. Vavilov Institute of General Genetics, Russian Academy of Sciences, Moscow, Russia; 5 Department of Ecosystem Science and Management, Texas A&M University, College Station, TX, United States of America

**Keywords:** *Betula*, Taxonomy, Phylogeny, ITS, Secondary structure, Phylogenetics

## Abstract

The taxonomy and phylogeny of the *Betula* L. genus remain unresolved and are very difficult to assess due to several factors, especially because of frequent hybridization among different species. In the current study, we used nucleotide sequences of two internal transcribed spacer regions (ITS1 and ITS2), which are commonly used as phylogenetic markers. In addition to their nucleotide variation we reconstructed their secondary structure and used it to resolve phylogenetic relationships of some birch species. We explored whether consideration of secondary structure in phylogenetic analyses based on neighbor-joining, maximum parsimony, maximum likelihood, and Bayesian inference methods would help us obtain more solid support of the reconstructed phylogenetic trees. The results were not unambiguous. There were only a few clades with higher support when secondary structure was included into analysis. The phylogenetic trees generated using different methods were mostly in agreement with each other. However, the resolving power of these markers is still insufficient to reliably discriminate some closely related species. To achieve this aim more reliably there is a need for application of modern genomic approaches in combination with traditional ones.

## Introduction

Birches (*Betula* L. genus) are common pioneer trees and shrubs of temperate and boreal zones in the Northern Hemisphere. Trees classified within this genus are highly diverse, especially in the Old World. Birches occupy habitats predominantly in cool and moist regions, including peatlands, stream banks, lakeshores, cool, damp woods, and moist slopes in cool coves. Most of them represent organisms adapted to low temperatures and able to grow under different extreme conditions (bogs, high altitude mountains, sands, subarctic zone, etc.) and on very poor soils, frequently as the first colonizing pioneer woody plants (Ashburner & McAllister, 2013).

The taxonomy and exact phylogenetic relations in the *Betula* L. genus are still unresolved and very difficult to assess because of several reasons. Number of taxa and their range vary greatly in different studies or sources from ∼30 (De Jong, 1993) to ∼120 (*World Checklist of Selected Plant Families;* Ashburner & McAllister, 2013) species. Birches comprise taxonomically a notoriously complex group because of their high morphological variation, phenotypic plasticity, and frequent hybridization (especially introgressive one) (Johnsson, 1945; Johnsson, 1949; Brown, Kennedy & Williams, 1982; Anamthawat-Jonsson & Tomasson, 1999; Anamthawat-Jonsson & Thorsson, 2003; Thorsson, Salmela & Anamthawat-Jonsson, 2001; Thorsson et al., 2010; Palmé et al., 2004; Bona, Petrova & Jadwiszczak, 2018).

Earlier classifications were mostly based on morphological traits (Regel, 1865; Schneider, 1915; Schneider, 1916; De Jong, 1993; Walters, 1993; Chen, Manchester & Sun, 1999; Skvortsov, 2002; Tzvelev, 2002; Ashburner & McAllister, 2013). The internal classification of the genus (subgenera, sections, and subsections) was changed several times, but even till now it is still mostly based on morphological traits. However, they are often insufficient to discriminate different birch species because of their high phenotypic plasticity and frequent hybridization, which blurs boundaries between different taxa. Only a few birch taxa were studied using different molecular genetic markers, such as *rbcL* and ITS sequences (Chen, Manchester & Sun, 1999), nuclear *ADH* and chloroplast *matK* sequences (Jarvinen et al., 2004), nuclear ribosomal DNA sequences (Li, Shoupa & Chen, 2005), nuclear nitrate reductase DNA sequences (Li, Shoupa & Chen, 2007), other multiple chloroplast and nuclear regions (Singevar et al., 2020) and AFLP markers (Schenk et al., 2008), but sampling sizes were quite low in most studies. In addition, there was a recent study of the genetic structure and hybridization of some birch species across Eurasia that provided new data regarding the hybridization patterns and the origin of *B. pubescens* (Tsuda et al., 2017). Some birch taxa were recently reassessed with propositions to lower taxonomic ranks using also internal transcribed spacers (ITS) as molecular genetic markers (e.g., Kuneš et al., 2019; Tarieiev et al., 2019; Jadwiszczak et al., 2020; Linda et al., 2020).

Genome-wide markers inferred by restriction site associated DNA sequencing (RAD-seq) were used to study the phylogeny of 27 diploid and 31 polyploid birch taxa (Wang et al., 2021) and specifically *Costatae* section with description of a new species, *Betula buggsii* N. Wang (Wang et al., 2020). Complete reference genome sequence assemblies are also available for three birch species, *B. nana* L. (Wang et al., 2013), *B. pendula* Roth (Salojärvi et al., 2017) and *B. platyphylla* (Chen et al., 2021) respectively.

ITS are among the most common phylogenetic markers in plants including also several studies on birch genus (Chen, Manchester & Sun, 1999; Li, Shoupa & Chen, 2005; Wang et al., 2016; Kuneš et al., 2019; Tarieiev et al., 2019). There is a large number of ITS sequences deposited and freely available in the NCBI GenBank, ITS2, and EMBL databases. The most complex phylogenetic reconstruction of ∼60 birch taxa (233 sequences in total) was done relatively recently using ITS sequences as phylogenetic markers (Wang et al., 2016). However, this phylogenetic study was unable to discriminate some closely related and hybrid taxa. The objective of our study was to test whether inclusion of secondary structure of ITS would improve phylogenetic analysis. In our study we inferred secondary structure of both ITS1 and ITS2.

## Materials & Methods

### ITS markers

We analyzed 223 complete nucleotide sequences of ITS1 that represented 73 birch taxa* (57 species, including two species with hybrid origin, 6 subspecies, 9 varieties, and one form) and 263 sequences of ITS2 that represented 78 birch taxa* (60 species, including two with hybrid origin, one interspecific hybrid, 6 subspecies, 10 varieties, and one form) retrieved from the NCBI GenBank and compared for secondary structure reconstruction with the ITS2 Database (for ITS2 only; Ankenbrand et al., 2015) (see Table S1).

In addition, we sequenced several ITS sequences representing the two endemic birch species from Ukraine *B. borysthenica* Klokov (Klokov, 1946) and *B. klokovii* Zaverucha (Zaverucha, 1964), dark-barked birches *B. pubescens* var. *sibakademica* Baranov (Kuzeneva) (≡*B. pubescens* f*. sibakademica* (Baranov) Tarieiev), (Baranov, 1924; Kuzeneva, 1936; Tarieiev et al., 2019) *B. kotulae* Zaverucha (=*B. pendula* f. *obscura* (Kotula) Tarieiev), (Zaverucha, 1964; Tarieiev et al., 2019) *B. atrata* Domin (Domin, 1927) and carpathian birch *B. pubescens* ssp. *carpatica* from Poland. We used specimens originating from natural habitats of Ukraine (especially endemic species from type localities (locus classicus) and type specimens listed in Olshansky et al., 2016), the Botanical Garden of the Georg-August University of Göttingen, and other herbaria–GOET, KW, LE and the former herbarium of Taras Shevchenko National University of Kyiv (see Table S2).

Birch taxa are listed in Tables S1 and S2 according to the NCBI taxonomy with more detailed explanation provided in Data S1. However, the NCBI taxonomic ranks do not always reflect the current changes in taxonomy and, therefore, could be wrong or outdated in some cases.

ITS1 and ITS2 from ribosomal gene clusters were amplified and sequenced in nine birch species either as a single fragment that included partial 18S, ITS1, 5.8S, ITS2, and partial 28S using universal PCR primers ITS5 and ITS4 (White et al., 1990) or in parts using ITS5 and ITS2 primer pairs modified for birch species to amplify the ITS1 region and ITS3 and ITS4 primer pairs to amplify the ITS2 region, respectively. PCR products were sequenced on an ABI 3130x Genetic Analyzer (Applied Biosystems, Thermo Fisher Scientific Inc., Waltham, MA, USA). Using BioEdit software (Hall, 1999) these sequences were checked visually for quality and aligned together with 223 and 263 complete sequences of ITS1 and ITS2 retrieved from the NCBI GenBank and ITS2 databases and presented in Tables S1 and S2, respectively. Secondary structure (Mai & Coleman, 1997; Coleman, 2000; Coleman, 2003; Coleman, 2007; Coleman, 2009; Müller et al., 2007; Wolf et al., 2013) was inferred for all analyzed sequences using mfold web server (Zuker, 2003). Obtained structures were checked for correctness (e.g., correct position of conservative motives, U-U mismatch, and 5.8S-28S rRNA hybrid stem formation were manually inspected for ITS2) and Gibbs energy value. Structures were compared with models proposed in the ITS2-Database (Koetschan et al., 2010; Koetschan et al., 2012) and aligned automatically with manual adjustment afterwards using the 4SALE program (Seibel et al., 2006; Seibel et al., 2008). Alignments are presented in Data S2 and S3. Obtained structures were visualized using VARNA v3-93 (Darty, Denise & Ponty, 2009), and then their features were colored using Inkscape v. 0.92.3 (Free Software Foundation, Inc.; https://inkscape.org).

Phylogenetic relationships were reconstructed using Bayesian inference implemented in MrBayes 3.2.6. (Huelsenbeck & Ronquist, 2001; Ronquist & Huelsenbeck, 2003; Ronquist et al., 2012) on the same sequence set with exclusion of identical sequences. Secondary structure was inferred by applying two different models: doublet model for paired regions and 4 by 4 with GTR +I for unpaired ones, respectively. The analysis was performed in four chains with sampling frequency 100 and different number of iterations –20,000,000, 50,000,000, and 100,000,000. Burnin was performed first separately in each run by monitoring the standard error value, convergence was considered to be reached when the standard error decreased below 0.01. The obtained final phylogenetic tree was visualised using FigTree (http://tree.bio.ed.ac.uk/software/figtree) and TreeGraph2 (Stöver & Müller, 2010).

To analyse the impact of rRNA secondary structure on the topology of the phylogenetic tree and support of the clades the following four methods of phylogenetic analysis were performed: (1) Bayesian analysis using an identical number of iterations and 4 by 4 GTR+I+G model suggested by the ModelTest in PAUP v.4.0a (http://paup.phylosolutions.com; Swofford, 2003) for all the regions, (2) neighbor-joining (NJ) clustering in ProfDist (Friedrich et al., 2005; Wolf et al., 2008) using GTR model, 1000 bootstrapping iterations and rate matrix Q for ITS2 in case of considering secondary structure or rate matrix Q for *Chlorophyta* in case of not considering secondary structure, (3) maximum parsimony (MP) analysis using PAUP v.4.0a with GTR+I+G model, 1000 bootstrap iterations, and 12-letter coding of secondary structure performed using 4SALE, 4) maximum likelihood (ML) analysis using MEGA X without considering secondary structure and R script provided with 4SALE package (http://4sale.bioapps.biozentrum.uni-wuerzburg.de/mlseqstr.html) together with 12-letter coding to consider the structure. It should be noted that 12-letter coding does not include IUPAC codes for ambiguous nucleotides, and, therefore, some information is lost, especially in case of considering hCBCs.

Pairwise topology of phylogenetic trees was adjusted manually for better comparison by merging and rearranging some clusters with statistically insignificant support, and respective drawings were generated using Inkscape v. 0.92.3 (Free Software Foundation, Inc.; https://inkscape.org).

## Results

### Secondary structure reconstruction

**Figure 1:**
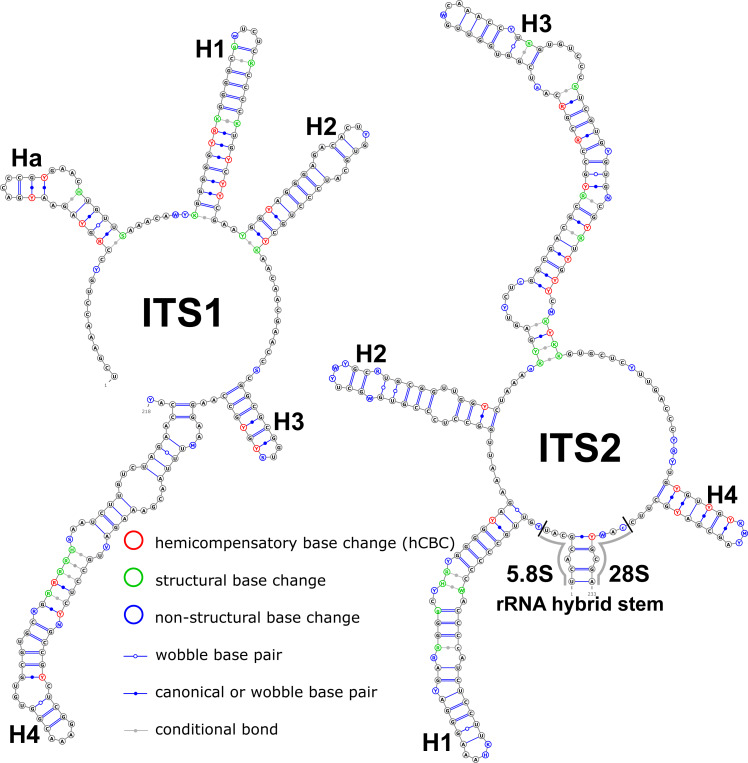
Consensus models of the secondary structure comprising all variants of ITS1 and ITS2 markers for the entire *Betula* L. genus. Indels are depicted by small letters.

Structural variation was observed for different groups of birch taxa. The consensus structural models comprising all variants were reconstructed for the ITS1 and ITS2 markers (Fig. 1).

**Figure 2:**
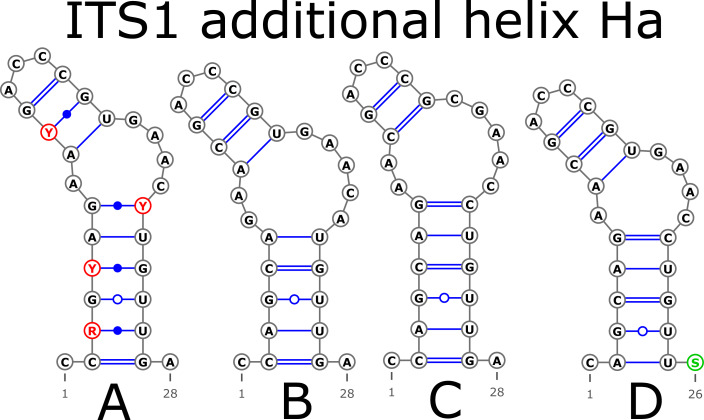
Structural variants of additional helix Ha.

The ITS1 structure consists of five helices: for main (H1, H2, H3, and H4) plus one additional (Ha). Additional helix Ha was present in four structural variants (Fig. 2): the A variant was common in most samples (Fig. 2A), B was specific for *B. globispica* (AB243883.1, KT308904.1, KT308905.1, AY761111.1), *B. chinensis* (KT308917.1, KT308918.1)*, B. fargesii* (KT308906) from section *Asperae,* subsection *Chinenses, B. glandulosa* (AY761110.1) from section *Apterocaryon, B. lenta* (AY761115.1, AY352330.1), *B. medwediewii* (AY761120.1), and *B. uber* (AY352334) from section *Lentae* (Fig. 2B), C was specific for *B. alnoides* (AY763114.1) and *B. luminifera* (KT308944.1) from the section *Acuminatae* (Fig. 2C), and D occurred only in two haplotypes of *B. apoiensis* (AB243908.1 and AB243912.1) (Fig. 2D).

**Figure 3:**
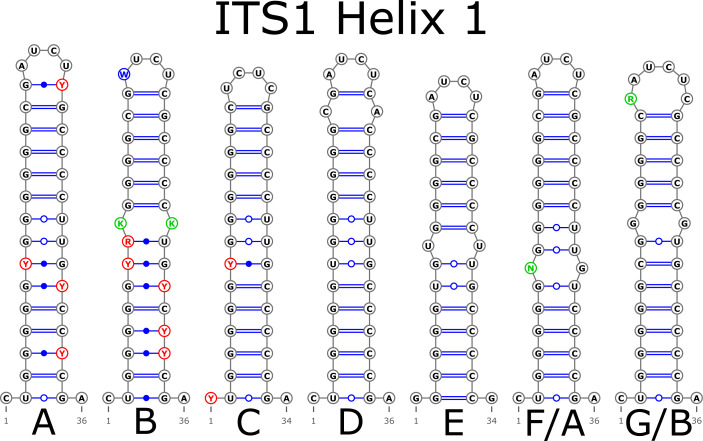
Structural variants of helix 1 (H1) in ITS1.

**Figure 4:**
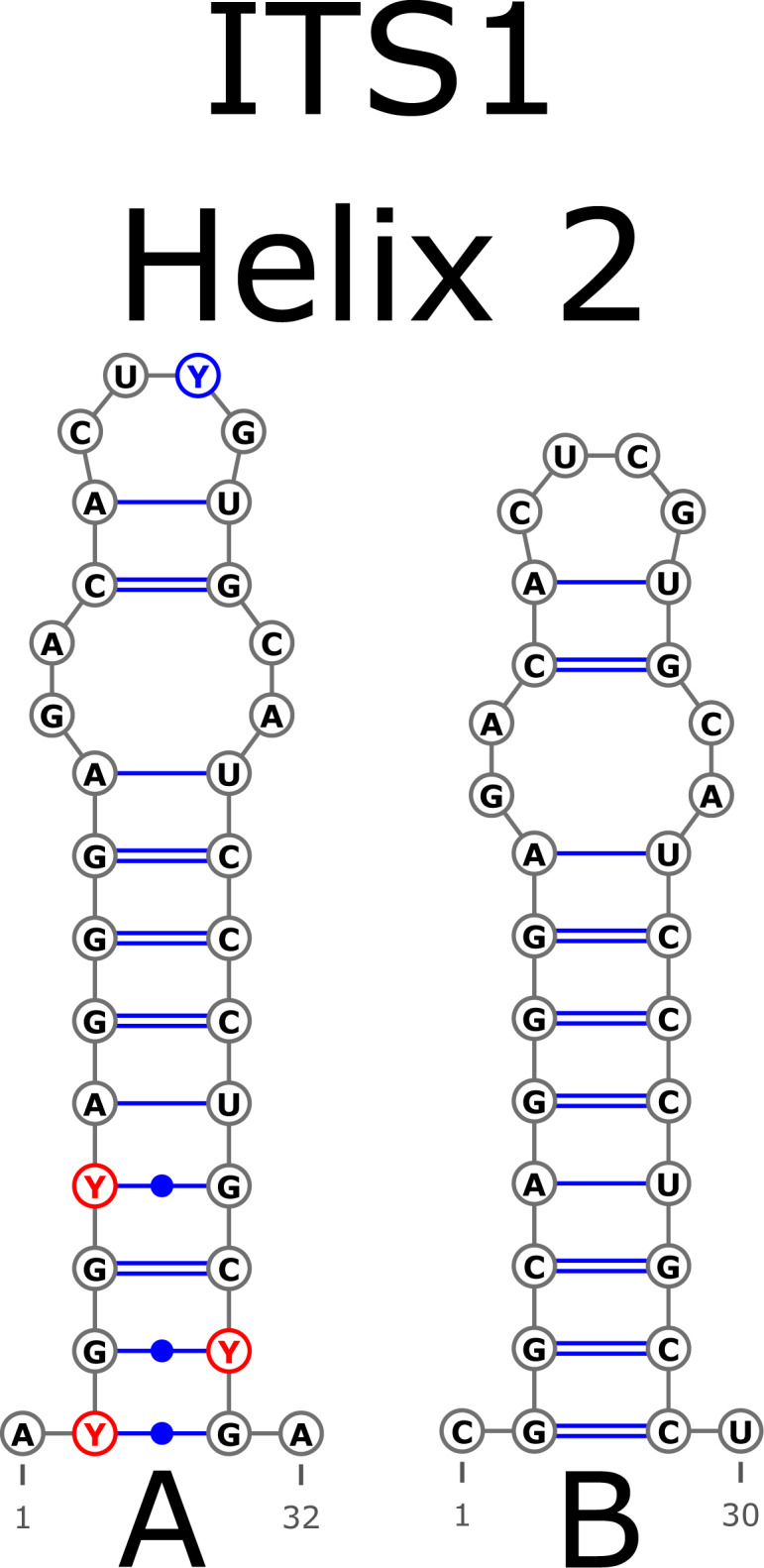
Structural variants of helix 2 (H2) in ITS1.

**Figure 5:**
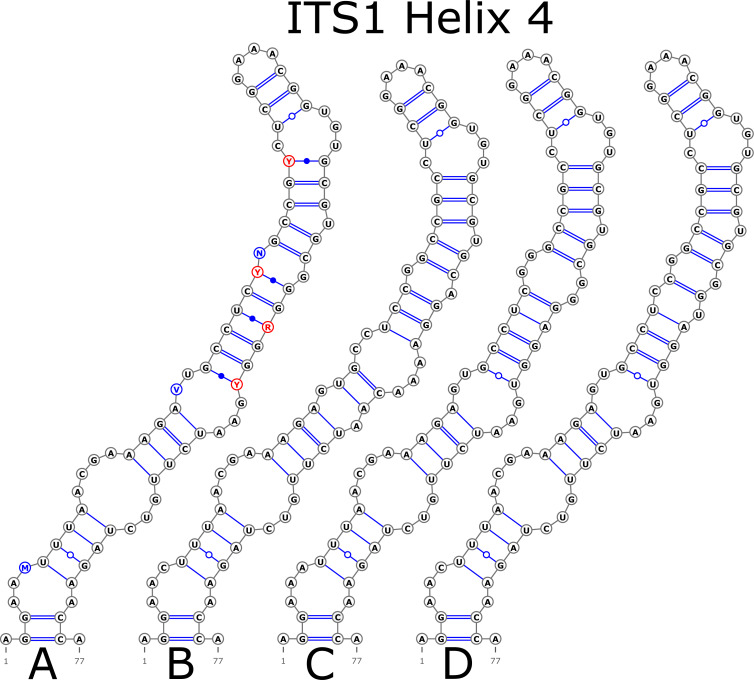
Structural variants of helix 4 (H4) in ITS1.

The H1 helix was the most variable in ITS1 and represented by seven structural variants (Fig. 3). However, the last two ones (F and G, respectively) could be reduced to the first and second one (A and B) depending on the nucleotide in ambiguous sites N and R. The H2 helix was represented by two structural variants A and B (Fig. 4). B was specific only for *B. insignis* (KT308927.1, KT308928.1, KT308929.1). The H3 helix was the same for all analyzed sequences and without any structural variation. Four structural variants were observed for the H4 helix (Fig. 5). The B variant was species-specific for *B. nigra* (see detailed information about distribution of other variants in Data S4).

**Figure 6:**
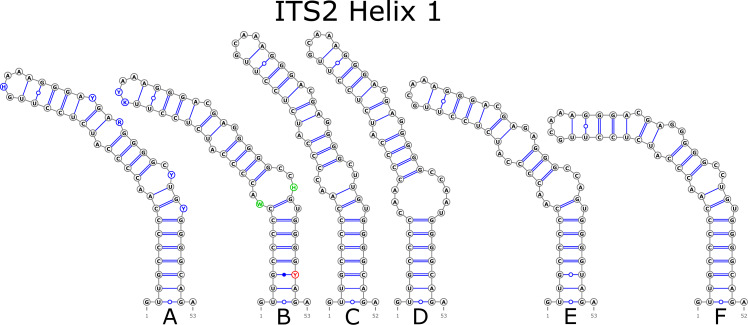
Structural variants of helix 1 (H1) in ITS2.

**Figure 7:**
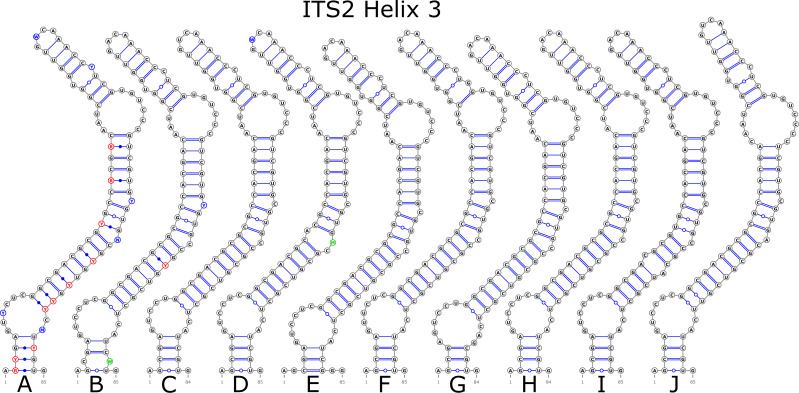
Structural variants of helix 3 (H3) in ITS2.

The ITS2 marker had no structural variation for H2 and H4 helices. Five structural variants were observed for the H1 helix (Fig. 6) (see also details in Data S3). The helix 3 (H3) was the most variable with 10 structural variants in total (Fig. 7). Further details regarding distribution of different variants among analyzed sequences are presented in Data S5.

There were no compensatory base changes (CBCs) detected in both structural models of markers across all samples. However, there were several hemicompensatory base changes (hCBCs): in total, 16 hCBCs sites were detected in ITS1 and 15 in ITS2, respectively (more detailed information is in Data S6 and S7). The exact distribution of those hCBCs is depicted in Figs. 1–7 in red color.

### Neighbor-joining (NJ) phylogenetic analysis

**Figure 8:**
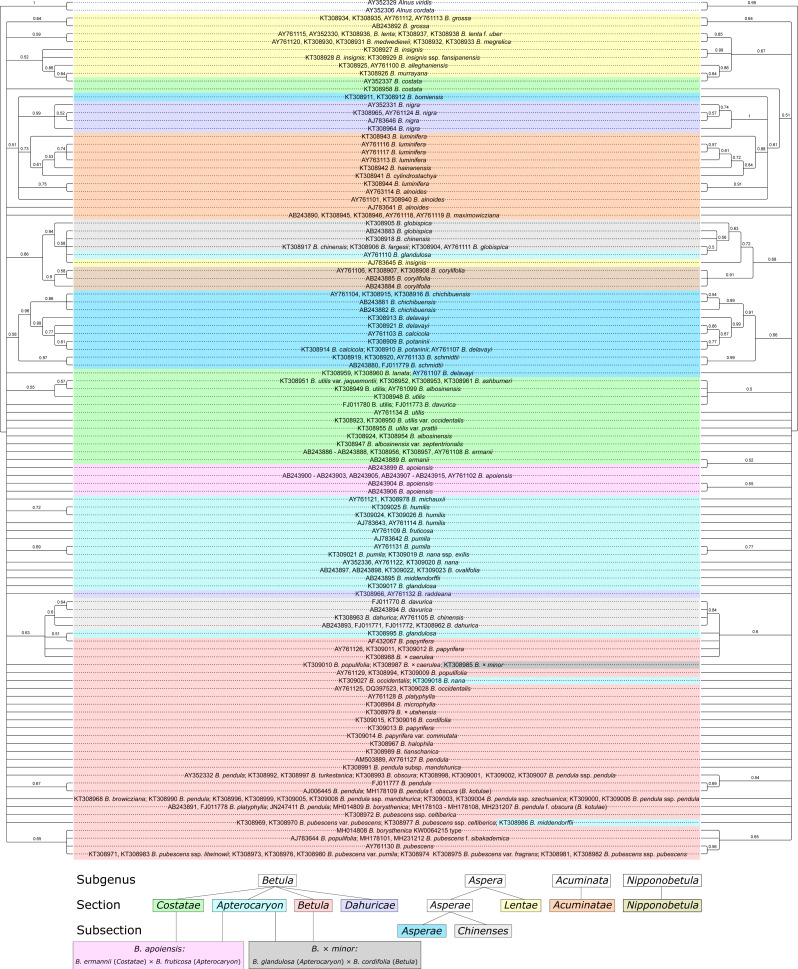
Two neighbor-joining (NJ) phylogenetic consensus trees based on 1,000 iterations without (left) and with (right) consideration of ITS secondary structure generated using ProfDistS with GTR model and rate matrix Q. They are presented also separately in Figs. S1–S2, respectively. Clusters with bootstrap value less than 0.5 (50%) were collapsed. Subgenera, sections, and subsections are marked by different colors according to Ashburner & McAllister (2013). Identical taxa are connected by lines.

NJ phylogenetic tree with consideration of the secondary ITS structure showed higher statistical support of several clusters and more detailed clustering compared to the NJ trees without such consideration (42 vs. 35 supported clusters, respectively, considering 50% as a cut-off for bootstrap support; Fig. 8, Figs. S1–S2). In addition, 14 clusters that were supported in both trees had higher bootstrap values when the secondary structure was considered. However, the resolution power remained insufficient to delineate some of closely related species and hybrids. In general, clustering was mainly in agreement with genus taxonomy, but not always.

### Maximum parsimony (MP) phylogenetic analysis

**Figure 9:**
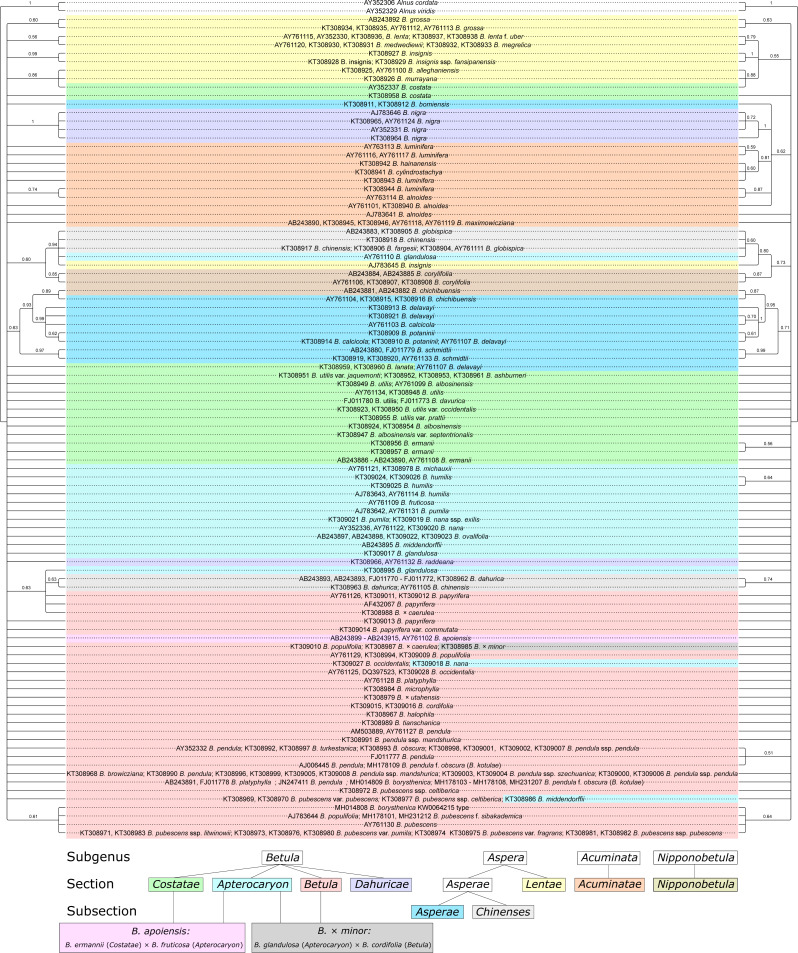
Two maximum parsimony (MP) phylogenetic consensus trees based on 1000 iterations and generated using PAUP without (left) and with (right) consideration of ITS secondary structure recorded as 12-letter code. They are presented also separately in Figs. S3–S4, respectively. Clusters with bootstrap value less than 0.5 (50%) were collapsed. Subgenera, sections, and subsections are marked by different colors according to Ashburner & McAllister (2013). Identical taxa are connected by lines.

MP phylogenetic trees demonstrated the same pattern as the NJ trees: there were more supported clusters when the secondary structure was considered compared to the MP tree without such consideration: 27 vs. 18, respectively (Fig. 9, Figs. S3–S4). In addition, twelve clusters that were supported in both trees demonstrated higher bootstrap values when the secondary structure was considered. However, the resolution power of the MP analysis was lower than in the NJ analysis.

### Maximum likelihood (ML) phylogenetic analysis

**Figure 10:**
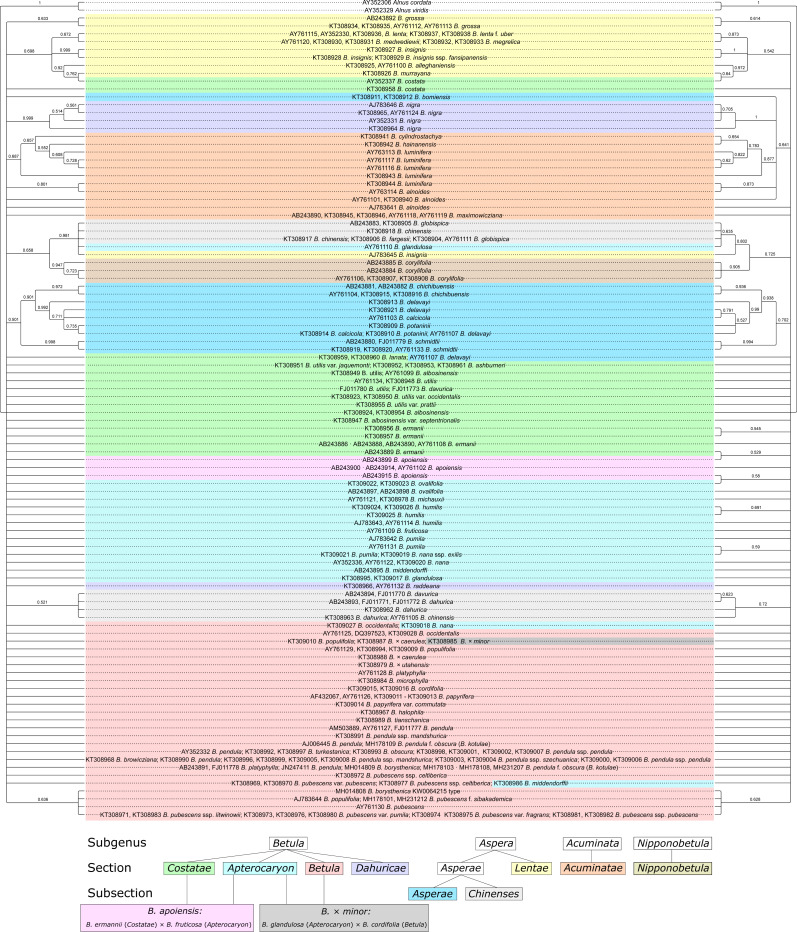
Two maximum likelihood (ML) phylogenetic consensus trees based on 1,000 iterations generated without consideration of ITS secondary structure using MEGA X (left) and with consideration of ITS secondary structure recorded as 12-letter code using 4SALE and phangorn script in R package (right) . They are presented also separately in Figs. S5–S6, respectively. Clusters with bootstrap value less than 0.5 (50%) were collapsed. Subgenera, sections, and subsections are marked according to Ashburner & McAllister (2013). Identical taxa are connected by lines.

MP phylogenetic trees demonstrated 34 supported clusters when secondary structure was considered, but only 28 ones when traditional analysis without consideration of secondary structure was used (Fig. 10, Figs. S5–S6). In addition, twelve clusters that were supported in both trees demonstrated higher bootstrap values when the secondary structure was considered.

### Bayesian phylogenetic analysis

**Figure 11:**
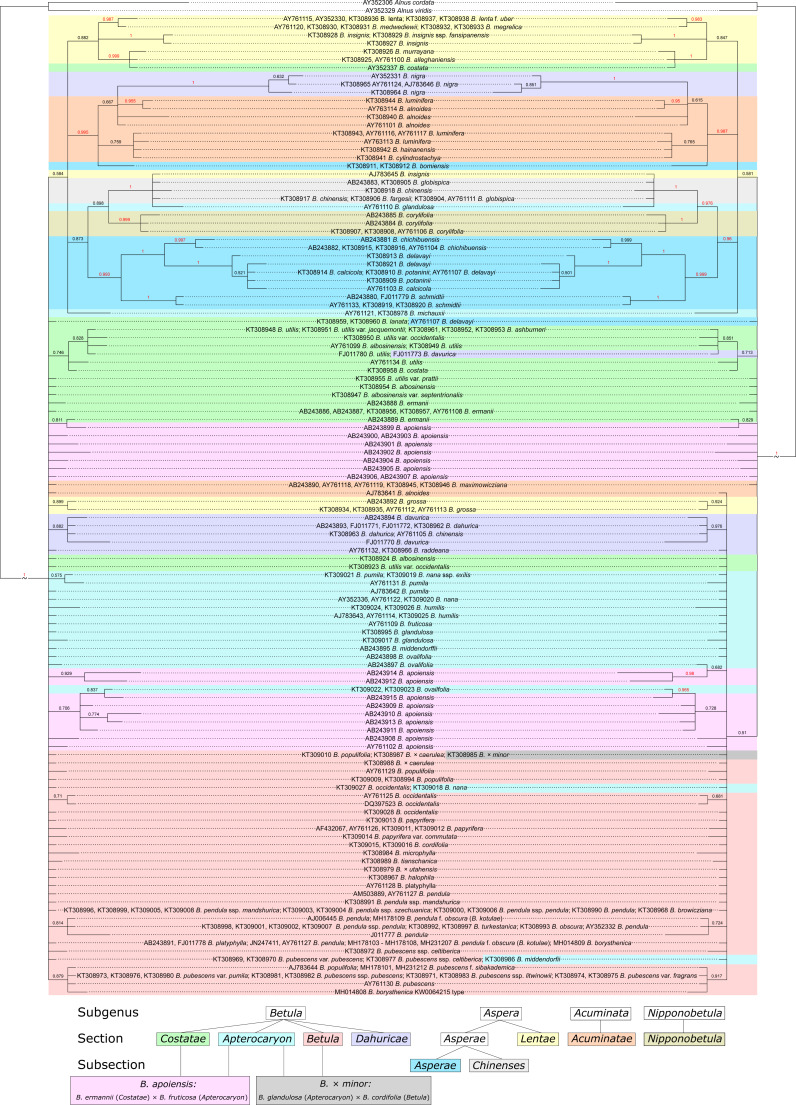
Two Bayesian phylogenetic trees based on 100000000 iterations including 10% burnin and 4 by 4 model for all regions without consideration of ITS secondary structure (left) and doublet model for paired regions, but 4 by 4 with GTR +I for unpaired ones with consideration of ITS secondary structure (right). They are presented also separately in Figs. S7–S8, respectively. Supported clades (>0.95) are highlighted in red. Subgenera, sections, and subsections are marked according to Ashburner & McAllister (2013). Identical taxa are connected by lines.

Bayesian phylogeny reconstructions showed some changes in phylogenetic trees; both changes in the topology and support values (posterior probability) were observed when the secondary structure of ITS was considered. Clusters supported with posterior probability values >95% are highlighted in red in Fig. 11, Figs. S7–S11.

Several Bayesian trees were constructed using different number of iterations (20,000,000, 50,000,000, and 100,000,000). At 20,000,000 iterations the tree with consideration of the secondary structure had greater statistical support values for several clades (Figs. S10, S11) compared to the tree without consideration of the secondary structure (Fig. S9). However, with increasing iteration number this difference vanished, and for 100,000,000 iterations the situation is opposite –the tree without structure had greater statistical support (Fig. 11, Figs. S7, S8). At the same time, the stability of the tree in terms of topology, number of clusters and support values was greater for the trees with structure. However, despite the fact that consideration of the secondary structure increased the stability of clusters, many closely related taxa inside the genus were still not resolved. Even subgenera, sections, and subsections (marked in Fig. 11, Figs. S7–S11 according to Ashburner & McAllister (2013) using the same symbols as in Wang et al. (2016) could not be clearly distinguished since there are some cases where taxa from different sections and subsections have totally identical ITS sequences.

## Discussion

Reconstructed secondary structure of ITS1 and ITS1 reveals a common pattern for the whole genus *Betula* L. The secondary structure for both of these spacers is more conservative than their sequences, and some common patterns are present across very large groups (Coleman, 2007). At the same time, secondary structure reconstruction reveals several types of nucleotide base changes, such as structural, non-structural, hemicompensatory (hCBC) and compensatory (CBC) changes, and, therefore, they can considerably enhance phylogenetic studies (Keller et al., 2010). The approach is described for a wide range of land plants, algae and animals (Coleman, 2009). In case of birch, structural, non-structural and hemicompensatory (hCBC) changes were detected, but compensatory base changes (CBCs) were not found. This was expected because CBCs are usually observed for fully reproductively isolated species (Coleman, 2000; Coleman, 2009; Müller et al., 2007), and, therefore, they could be used in delineation of biological species according to Mayr’s biological species concept (Mayr, 1942). Meanwhile, birches have very weak reproductive barriers with common hybridization events, and a number of taxa have hybrid origin (Ashburner & McAllister, 2013; Anamthawat-Jonsson & Thorsson, 2003; Anamthawat-Jonsson & Tomasson, 1999; Brown, Kennedy & Williams, 1982; Johnsson, 1945; Johnsson, 1949; Thorsson, Salmela & Anamthawat-Jonsson, 2001; Thorsson et al., 2010; Palmé et al., 2004; Thomson et al., 2015; Wang et al., 2014; Zohren et al., 2016). Therefore, the biological species concept is hardly applicable to many birch species. There are several other species concepts that could better accommodate this genus, but there is currently no strict consensus on this. Genetic clustering is a common approach to delineate birch species, but it could be spurious in case of species with high gene flow that can easily hybridize (Tsuda et al., 2017).

Some birch taxa, even from different subgenera, sections, and subsections, have identical ITS sequences and, therefore, cannot be resolved using ITS markers. Since most of the sequences were retrieved from the GenBank, it is not always possible to verify the exact origin of the samples and correct species identification. The errors could occur due to morphological misidentification, and it is often impossible to check the accuracy of morphological identification of those specimens. However, identity of ITS sequences in species that have very distinctive morphology could be real, especially if it was confirmed by sequence data obtained by different independent research groups for the same species. It is likely that birch is still in the process of speciation, and reproductive barriers are not established yet or weak isolation is beneficial for survival in extreme environments. This reproductive feature is likely under positive selection, which could also explain the relatively low variation of ITS sequences within diverse groups. There are circa 15 known hybrids between different subgenera, sections, and subsections (Ashburner & McAllister, 2013). However, most of them were not included in this study. The exceptions are *B.* ×* minor* (Tuck.) Fernald, which is a hybrid between *B. cordifolia* Regel (2*n* = 2*x* = 28, sect. *Betula*) and *B. glandulosa* Michx (2*n* = 2*x* = 28, sect. *Apterocaryon*), and *B. apoiensis* which is likely a hybrid between *B. ermannii* Cham. (2*n* = 4*x* = 56, sect. *Costatae*) and *B. fruticosa* Pallas (2*n* = 4*x* = 56, sect. *Apterocaryon*) (Ashburner & McAllister, 2013). This could explain a variety of different haplotypes of *B. apoiensis* with different clustering in the dendrograms. It should also be noted that those taxonomic categories such as subgenera, sections and subsections within birch genus were based only on morphological traits and do not necessarily reflect genetic differences. Multiple copies of ITS regions of different origin could be present in allopolyploids and interspecific hybrids, but only one of them could be amplified by PCR, which can also result in spurious phylogenetic trees. Recent genome-wide RAD-seq studies (Wang et al., 2020; Wang et al., 2021) solved some specific problems in birch taxonomy and phylogeny, especially clarifying parentage of some tetraploid species, enhancing resolution for diploid birch species with proposing changes on the level of sections and subsections and describing the new species *B. buggsii* that is morphologically similar to *B. utilis* ssp. *albosinensis*. However, some problems especially with polyploids with high ploidy levels remain. Therefore, we believe that further genome wide analyses can help to resolve the phylogenetic and systematic uncertainties in genus *Betula* even better.

## Conclusions

The general models of secondary structure of the ITS1 and ITS2 sequences across the whole genus *Betula* L. were presented for the first time in this study. Species-specific structural variants were detected for *B. insignis* and *B. nigra.* In combination with sequence variation it provides higher confidence in clustering, but only when the number of iterations is relatively low or moderate. With increasing the number of iterations the number of supported clusters and support values for the tree considering secondary structure remain more or less the same, while for the tree without considering secondary structure the number of supported clusters and support values tend to increase. In case of very closely related and hybrid taxa, ITS markers did not provide sufficient discrimination, as well as even some subsections and sections could not be clearly separated. In general, our data were in agreement with the relatively recently published phylogenetic study of the entire genus *Betula* L. based on the nucleotide variation of the ITS markers (Wang et al., 2016), but structural variation can additionally be used to distinguish or support some taxa.

##  Supplemental Information

10.7717/peerj.10889/supp-1 Supplemental Information 1 Taxonomic note

10.7717/peerj.10889/supp-2 Supplemental Information 2 ITS1 structural alignment

10.7717/peerj.10889/supp-3 Supplemental Information 3 ITS2 structural alignment

10.7717/peerj.10889/supp-4 Supplemental Information 4 Distribution of different structure variants in ITS1

10.7717/peerj.10889/supp-5 Supplemental Information 5 Distribution of different structure variants in ITS2

10.7717/peerj.10889/supp-6 Supplemental Information 6 Hemicompensatory base changes (hCBCs) in ITS1

10.7717/peerj.10889/supp-7 Supplemental Information 7 Hemicompensatory base changes (hCBCs) in ITS2

10.7717/peerj.10889/supp-8 Supplemental Information 8 Neighbor-joining phylogenetic consensus tree based on 1000 iterations generated considering ITS secondary structure and using ProfDistS with GTR model and rate matrix Q designed for ITS2 Clusters with bootstrap value less than 0.5 (50%) were collapsed. Subgenera, sections, and subsections are marked according to Ashburner & McAllister (2013) using symbols as in Wang et al. (2016) with our modification concerning hybrids.

10.7717/peerj.10889/supp-9 Supplemental Information 9 Neighbor-joining phylogenetic consensus tree based on 1000 iterations generated without considering ITS secondary structure and using ProfDistS with GTR model and rate matrix Q designed for Chlorophyta Clusters with bootstrap value less than 0.5 (50%) were collapsed. Subgenera, sections, and subsections are marked according to Ashburner & McAllister (2013) using symbols as in Wang et al. (2016) with our modification concerning hybrids.

10.7717/peerj.10889/supp-10 Supplemental Information 10 Maximum parsimony (MP) phylogenetic consensus tree based on 1000 iterations and generated using PAUP without considering ITS secondary structure Clusters with bootstrap value less than 0.5 (50%) were collapsed. Subgenera, sections, and subsections are marked according to Ashburner & McAllister (2013) using symbols as in Wang et al. (2016) with our modification concerning hybrids.

10.7717/peerj.10889/supp-11 Supplemental Information 11 Maximum parsimony (MP) phylogenetic consensus tree based on 1000 iterations and generated using PAUP considering ITS secondary structure recorded as 12-letter code Clusters with bootstrap value less than 0.5 (50%) were collapsed. Subgenera, sections, and subsections are marked according to Ashburner & McAllister (2013) using symbols as in Wang et al. (2016) with our modification concerning hybrids.

10.7717/peerj.10889/supp-12 Supplemental Information 12 Maximum likelihood (ML) phylogenetic consensus tree based on 1000 iterations generated using MEGA X without considering ITS secondary structure Clusters with bootstrap value less than 0.5 (50%) were collapsed. Subgenera, sections, and subsections are marked according to Ashburner & McAllister (2013) using symbols as in Wang et al. (2016) with our modification concerning hybrids.

10.7717/peerj.10889/supp-13 Supplemental Information 13 Maximum likelihood (ML) phylogenetic consensus tree based on 1000 iterations generated considering ITS secondary structure recorded as 12-letter code using 4SALE and *phangorn* script in R package Clusters with bootstrap value less than 0.5 (50%) were collapsed. Subgenera, sections, and subsections are marked according to Ashburner & McAllister (2013) using symbols as in Wang et al. (2016) with our modification concerning hybrids.

10.7717/peerj.10889/supp-14 Supplemental Information 14 Bayesian phylogenetic tree generated without considering ITS secondary structure and based on 100000000 iterations including 10% burnin and 4 by 4 model for all regions Supported clades ( >0.95) are highlighted in red. Subgenera, sections, and subsections are marked according to Ashburner & McAllister (2013) using the same symbols as in Wang et al. (2016).

10.7717/peerj.10889/supp-15 Supplemental Information 15 Bayesian phylogenetic tree generated considering ITS secondary structure and based on 100000000 iterations including 10% burnin, doublet model for paired regions, and 4 by 4 with GTR +I for unpaired ones Supported clades ( >0.95) are highlighted in red. Subgenera, sections, and subsections are marked according to Ashburner & McAllister (2013) using symbols as in Wang et al. (2016) with our modification concerning hybrids.

10.7717/peerj.10889/supp-16 Supplemental Information 16 Bayesian phylogenetic tree generated without considering ITS secondary structure and based on 20000000 iterations including 10% burnin, doublet model for paired regions, and 4 by 4 with GTR +I for unpaired ones Supported clades ( >0.95) are highlighted in red. Subgenera, sections, and subsections are marked according to Ashburner & McAllister (2013) using symbols as in Wang et al. (2016) with our modification concerning hybrids.

10.7717/peerj.10889/supp-17 Supplemental Information 17 Bayesian phylogenetic tree generated considering ITS secondary structure and based on 20000000 iterations including 10% burnin, doublet model for paired regions, and 4 by 4 with GTR +I for unpaired ones Supported clades ( >0.95) are highlighted in red. Subgenera, sections, and subsections are marked according to Ashburner & McAllister (2013) using symbols as in Wang et al. (2016) with our modification concerning hybrids.

10.7717/peerj.10889/supp-18 Supplemental Information 18 Two Bayesian phylogenetic trees based on 20000000 iterations including 10% burnin and 4 by 4 model for all regions without consideration of ITS secondary structure (left) and doublet model for paired regions, but 4 by 4 with GTR +I for unpaired ones with They are presented also separately in Figures S7 and S8, respectively. Supported clades ( >0.95) are highlighted in red. Subgenera, sections, and subsections are marked according to Ashburner & McAllister (2013). Identical taxa are connected by lines.

10.7717/peerj.10889/supp-19 Supplemental Information 19 The birch ITS sequences retrieved from NCBI GenBank used in the study

10.7717/peerj.10889/supp-20 Supplemental Information 20 The birch ITS sequenced by the authors and used in the study

## Additional Information and Declarations

### Data Availability

The following information was supplied regarding data availability: Raw data is available in the Supplemental Files.
