# Supplementary material for: ITS secondary structure reconstruction to resolve taxonomy and phylogeny of the Betula L. genus
Source: PeerJ. 2021 Mar 23;9:e10889. doi: 10.7717/peerj.10889 (PMC7996101; doi:10.7717/peerj.10889)
Supplement: Supplemental Information 1 [file peerj-09-10889-s001.docx]

**Data S1. Taxonomic note**

Due to spurious taxonomy of birch genus, some current species names are either questionable or revised in recent studies and not valid anymore. We list below some changes according to our recent study (Tarieiev et al. 2019) and last edition of The Plant List (theplantlist.org) and World Checklist of Selected Plant Families (WCSP).

*Betula borysthenica* Klokov [Bot. Zhurn. (Kiev) 3(1-2): 18 (1946)] is an endemic species growing on sands in lower reaches of Dnieper (Pivdenny Bug, Sivers’ky Donets, Ukraine), Volga and Yeruslan Rivers (Saratov Region, Russia). It was described by Klokov (1946) and is now listed in the Red Book of Ukraine (Moysiyenko et al. 2009). The holotype and other authentic specimens are kept in the National Herbarium of Ukraine (KW) in Kyiv. It should be noted that currently this species is not recognized as a separate species by several world checklists. For example, the World Checklist of Selected Plant Families (WCSP) proposed to treat it as a type variety of *Betula pubescens* Ehrh. However, all these taxonomical conclusions were based only on literature data without any verification with authentic materials. Meanwhile, there are significant morphological differences of this species to other taxa in indumentum of twigs and leaves, shape of leaf plates, presence of resinous glands on young twigs, etc.

*Betula klokovii* Zaverucha [Ukrayins'k. Bot. Zhurn. 21(5): 80 (1964)] is a strictly endemic species with unclear origin, described in west-north of Podillya, Ukraine by Zaverucha (1964). This is a very rare species that grows only on the top of two chalk hills near Kremenets–Maslyatyn and Strakhova and listed in the IUCN Red List of Threatened species (Rivers and Tarieiev 2015) and the Red Book of Ukraine (Kagalo and Skibitska 2009) as critically endangered (CR). The entire population currently consists of ~50 trees, and this number is decreasing.

*Betula kotulae* Zaverucha [Ukrayins'k. Bot. Zhurn. 21(5): 83 (1964)] is a species described also in northwest of Podillya by Zaverucha (1964). It is also listed in the Red Book of Ukraine (Kagalo 2009). Since there are no morphological differences between this species and earlier described *B. obscura* Kotula ex Fiek, and both of them differ from *B. pendula* by only one trait – dark color of their bark, we proposed to treat these species as form *B. pendula* f. *obscura* (Kotula ex Fiek) Tarieiev (Tarieiev et al. 2019).

*Betula pubecsens* var. *sibakademica* (Baranov) Kuzeneva [Fl. URSS 5:296 (1936)]– a dark-barked birch variety, described from Siberia by V. Baranov (Baranov 1924) as a separate species *Betula* *sibakademica* Baranov [Izv. Zap. Sib. Otd. Rus. Geogr. Obsch. 4: 47-54 (1924)]. Based on ITS sequence and morphological data we proposed to treat this variety as the form *B. pubescens* f. *sibakademica* (Baranov) Tarieiev (Tarieiev et al. 2019).

*Betula* *obscura* Kotula ex Fiek [Jahresber. Schles. Ges. Vaterl. Cult. 65: 314 (1888)] is a synonym of *Betula* × *aurata* Borkh. [Theor. Prakt. Handb. Forstbot. 1: 378 (1800)] according to WCSP. However, we think that it is not correct, since the only trait distinguishing this birch from typical *B. pendula* Roth is the color of the bark, and it never occurs outside the range of silver birch and as a separate population. Therefore, we propose to treat this birch as a separate form *B. pendula* f. *obscura* (Tarieiev et al. 2019).

*Betula albosinensis* Burkill [J. Linn. Soc., Bot. 26: 497 (1899)] is accepted by The Plant List, but it is a synonym of *Betula utilis* subsp. *albosinensis* (Burkill) Ashburner & McAll. [Gen. Betula: 264 (2013)].

*Betula apoiensis* Nakai ex H. Hara [J. Jap. Bot. 10: 227 (1937)] is accepted by The Plant List, but is a synonym of *Betula gmelinii* Bunge [Verz. Altai Pfl.: 113 (1835)] by WCSP. According Ashburner and McAllister, 2013 supposed to be a hybrid between *Betula ermannii* Cham.and *B. fruticosa* Pallas.

*Betula atrata* Domin [Bull. Internat. Acad. Sc. xxviii. 249, Prague (1927)] is a synonym of *Betula × aurata* Borh according to WCSP. There is also another recent conclusion that *B. atrata* should be treated as *B. pendula* (Kuneš et al. 2019). However, according to the initial description (Domin 1927) it has a dark bark color. Additionally, the type specimen of this birch (PRC 454665) likely represents two different birch taxa simultaneously.

*Betula borysthenica* Klokov [Bot. Zhurn. (Kiev) 3: 18 (1947)] was earlier described as *Betula pubescens* var. *glabrata* Wahlenb. [Fl. Carpat. Princ. 306 (1814)] and remains under this name in The Plant List. According to WCSP it is a synonym for *B. pubescens* var. *pubescens*. However, it has a number of distinctive traits (lack of the indumentum, clonal growth, etc.) and therefore the taxonomic rank and position of this birch remain to be unresolved due to the lack of research data.

*Betula fargesii* Franch. [J. Bot. (Morot) 13: 205 (1899)] is accepted by WCSP, but it is a synonym of *Betula chinensis* Maxim. [Bull. Soc. Imp. Naturalistes Moscou 54(1): 47 (1879)] in The Plant List.

*Betula jaquemontii* Spach. [Ann. Sci. Nat., Bot., sér. 2, 15: 189 (1841)] is a synonym for *Betula utilis subsp. jacquemontii* (Spach) Kitam. [Pl. West Pak. Afgh.: 37 (1964)].

*Betula lanata* (Regel) V. N. Vassil. [Bot. Zhurn. S.S.S.R. 27: 11 (1942)] is accepted by The Plant List, but is a synonym for *Betula ermanii* var. *lanata* Regel [Nouv. Mém. Soc. Imp. Naturalistes Moscou 13(2): 122 (1861)] according to WCSP.

*Betula lutea* F. Michx. (nom. Illeg.) [Hist. Arb. 2(5): 153 (1812)] is a synonym of *Betula alleghaniensis* Britton [Bull. Torrey Bot. Club 31: 166 (1904)].

*Betula middendorffii* Trautv. & C. A. Mey. [in Middend. Reise, Fl. Ochot. Phaen. 1(2):84 (1856)] is a synonym of *Betula divaricata* Ledeb. [Denkschr. Baier. Bot. Ges. 3: 59 (1841)] in The Plant List or of *Betula fruticosa* Pall. [Reise Russ. Reich. 3: 758 (1776)] according to WCSP.

*Betula platyphylla* Sukaczev [Trudy Bot. Muz. Imp. Akad. Nauk 8: 220 (1911)] is an accepted name in The Plant List, but is a synonym of *Betula pendula* subsp. *mandshurica* (Regel) Ashburner & McAll. [Gen. Betula: 291 (2013)] according to WCSP.

*Betula pubescens* ssp. *carpatica* (Waldst. & Kit. ex Willd.) Asch. & Graebn [Fl. Nordostdeut. Flachl.: 253 (1898)] is considered to be a synonym for *Betula pubescens* var. *glabrata* Wahlenb. [Fl. Carpat. Princ. 306 (1814)] or *B. pubescens* var. *pubescens* according to the World Checklist of Selected Plant Families (WCSP).

*Betula uber* (Ashe) Fernald [Rhodora 47: 325 (1945)] is a synonym of *Betula lenta* subsp. *uber* (Ashe) A. E. Murray [Kalmia 12: 18 (1982)] in The Plant List or *Betula lenta* f. *uber* (Ashe) McAll. & Ashburner [Bot. Mag. 21: 58 (2004)] according to the WCSP.

**References**

**Ashburner K, McAllister HA. 2013.** *The Genus Betula: a taxonomic revision of birches.* Kew: Kew publishing, Royal Botanical Gardens.

**Baranov VI. 1924.** Notes about birches from forest steppe of Western Siberia. *Reports of Western-Siberian Department of Russian Geographical Society* **4:**47-54.

**Domin K. 1927.** *Betula atrata* Domin, nová břiza z Českomoravské vysočiny. *Rozpravy II třídy České akademie* **36(52):**1–10. In Czech

**Kagalo OO, Skibitska NV. 2009.** *Betula klokovii* Zaverucha. In: Didukh Ya P, ed. *Red book of Ukraine. Plants.* Kyiv: Globalconsulting, 344.

**Kagalo OO. 2009.** *Betula obscura* A.Kotula (incl. *B. kotulae* Zaverucha; *B . pendula* Roth subsp. *obscura* (A.Kotula) Á.Löve, *B. verrucosa* Ehrh. subsp. *obscura* (A.Kotula) Á.Löve et D.Löve). In: Didukh Ya P, ed. *Red book of Ukraine. Plants.* Kyiv: Global consulting, 345.

**Klokov MV. 1946.** New materials to knowledge Ukrainian flora. *Botanical Journal AS Ukrainian SSR* **3(1-2):**17-18.

**Kuneš I, Linda R, Fér T, Karlík P, Baláš M, Ešnerová J, Vítámvás J, Bílý J, Urfus T. 2019.** Is *Betula carpatica* genetically distinctive? A morphometric, cytometric and molecular study of birches in the Bohemian Massif with a focus on Carpathian birch. *PLOS ONE* **14(10):**e0224387 DOI 10.1371/journal.pone.0224387.

**Moysiyenko II, OYu Umanets, Boiko MF. 2009.** *Betula borysthenica* Klokov. In: Didukh Ya P, ed. *Red book of Ukraine. Plants.* Kyiv: Global consulting, 342.

**Rivers MC, Tarieiev A. 2015.** *Betula klokovii*. *The IUCN Red List of Threatened Species* 2015: e.T194573A79345402.

**Tarieiev AS, Olshanskyi I, Gailing O, Krutovsky KV. 2019.** Taxonomy of dark- and white-barked birches related to *Betula pendula* Roth and *B. pubescens* Ehrh. (Betulaceae) in Ukraine based on both morphological traits and DNA markers. *Botanical Journal of the Linnean Society* **191(1):**142-154 DOI 10.1093/botlinnean/boz031.

**Zaverucha BV. 1964.** New and rare birch species of Ukrainian flora. *Ukrainian Botanical Journal* **21(5):**78-86.
