# Supplementary material for: ITS secondary structure reconstruction to resolve taxonomy and phylogeny of the Betula L. genus
Source: PeerJ. 2021 Mar 23;9:e10889. doi: 10.7717/peerj.10889 (PMC7996101; doi:10.7717/peerj.10889)
Supplement: Supplemental Information 2 [file peerj-09-10889-s002.docx]

**Data S2**. ITS1 structural alignment.

>AJ006445 *Betula* *pendula* 18S, 5.8S and 28S rRNA genes and internal transcribed spacers (ITS1 and ITS2)

UCGAAACCUGCCCAGCAGAACGACCCGUGAACCUGUUGAAACAACUGGGGGUGUGGGGCGAUCUCGCCCCUUGCCCCCGAACGGUAGGGAGACACUUGUGCAUCCCUGCCGAACAACGAACCCCGGCGCGGUCCGCGCCAAGGAACUUUAACGAAAGAGUGCCUCCGGCCGCCUCGGAAACGGUGUGCGUGCGGGAGGUGAAUCUUGUCUAGAACCAU

............((((((.(((...)))....)))))).......((((((((.((((((....)))))).))))))))..(((((((((..((....))..))))))))).............((((((...))))))..((...((((....((((..((((((.(((((.(((....)))...))).)).))))))...))))...)))).))..

>AJ783646 *Betula* *nigra* 18S rRNA gene (partial), 5.8S rRNA gene, 26S rRNA gene (partial), ITS1 and ITS2

UCGAAACCUGCCCAGCAGAACGACCCGUGAACCUGUUGAAACAACUGGGGGUGGGGGGCGAUCUCGCCCCGUGCCUCCGAACGGUAGGGAGACACUUGUGCAUCCCUGCUGAACAACGAACCCCGGCGCGGUCUGCGCCAAGGAACUUUAACGAAAGAGUGCCUCCGGCCGCCUCGGAAACGGUGUGCGUGCAGGAAAACAAUCUUGUCUAGAACCAC

............((((((.(((...)))....)))))).......((((((((.((((((....)))))).))))))))..(((((((((..((....))..))))))))).............((((((...))))))..((...((((....((((.((..(((.(((((.(((....)))...))).)).)))...)).))))...)))).))..

>AJ783645 *Betula* *insignis* 18S rRNA gene (partial), 5.8S rRNA gene, 26S rRNA gene (partial), ITS1 and ITS2

UCGAAACCUGCCCAGCAGAACGACCCGUGAACAUGUUGAAACAACUGGGGGCGGGGGGCGAUCUCGCCCCGUGCCCCCGAACGGUAGGGAGACACUCGUGCAUCCCUGCCGAACAACGAACCCCGGCGCGGUCUGCGCCAAGGAACUUUAACGAAAGAGUGCCUCCGGCCGCCUCGGAAACGGUGUGCGUGCGGGAGGUGAAUCUUGUCUAGAACCAU

............((((((.(((...)))....)))))).......((((((((.((((((....)))))).))))))))..(((((((((..((....))..))))))))).............((((((...))))))..((...((((....((((..((((((.(((((.(((....)))...))).)).))))))...))))...)))).))..

>AJ783644 *Betula* *populifolia* 18S rRNA gene (partial), 5.8S rRNA gene, 26S rRNA gene (partial), ITS1 and ITS2

UCGAAACCUGCCCAGCAGAACGACCCGUGAACCUGUUGAAACAACUGGGGGUGGGGGGCGAUCUCGCCCCUUGCCCCCGAACGGUAGGGAGACACUUGUGCAUCCCUGCCGAACAACGAACCCCGGCGCGGUCCGCGCCAAGGAACUUUAACGAAAGAGUGCCUCCGGCCGCCUCGGAAACGGUGUGCGUGCGGGAGGUGAAUCUUGUCUAGAACCAU

............((((((.(((...)))....)))))).......(((((((((((((((....)))))))))))))))..(((((((((..((....))..))))))))).............((((((...))))))..((...((((....((((..((((((.(((((.(((....)))...))).)).))))))...))))...)))).))..

>AJ783643 *Betula* *humilis* 18S rRNA gene (partial), 5.8S rRNA gene, 26S rRNA gene (partial), ITS1 and ITS2

UCGAAACCUGCCCAGCAGAACGACCCGUGAACCUGUUGAAACAACUGGGGGUGGGGGGCGAUCUCGCCCCUUGCCCCCGAACGGUAGGGAGACACUCGUGCAUCCCUGCCGAACAACGAACCCCGGCGCGGUCUGCGCCAAGGAACUUUAACGAAAGAGUGCCUCCGGCCGCCUCGGAAACGGUGUGCGUGCGGGAGGUGAAUCUUGUCUAGAACCAU

............((((((.(((...)))....)))))).......(((((((((((((((....)))))))))))))))..(((((((((..((....))..))))))))).............((((((...))))))..((...((((....((((..((((((.(((((.(((....)))...))).)).))))))...))))...)))).))..

>AJ783642 *Betula* *pumila* 18S rRNA gene (partial), 5.8S rRNA gene, 26S rRNA gene (partial), ITS1 and ITS2

UCGAAACCUGCCCAGCAGAACGACCCGUGAACCUGUUGAAACAACUGGGGGUGGGGGGCGAUCUCGCCCCUUGCCCCCGAACGGUAGGGAGACACUUGUGCAUCCCUGCCGAACAACGAACCCCGGCGCGGUCUGCGCCAAGGAACUUUAACGAAAGAGUGCCUCCGGCCGCCUCGGAAACGGUGUGCGUGCGGGAGGUGAAUCUUGUCUAGAACCAU

............((((((.(((...)))....)))))).......(((((((((((((((....)))))))))))))))..(((((((((..((....))..))))))))).............((((((...))))))..((...((((....((((..((((((.(((((.(((....)))...))).)).))))))...))))...)))).))..

>AJ783641 *Betula* *alnoides* 18S rRNA gene (partial), 5.8S rRNA gene, 26S rRNA gene (partial), ITS1 and ITS2

UCGAAACCUGCCCAGCAGAACGACCCGUGAACCUGUUGAAACAACUGGGGGUGGGGGGCGAUCUCGCCCCUUGCCCCCGAACGGUAGGGAGACACUUGUGCAUCCCUGCCGAACAACGAACCCCGGCGCGGUCYGCGCCAAGGAACUUUAACGAAAGAGUGCCUCCGGCCGCCUCGGAAACGGUGUGCGUGCGGGAGGUGAAUCUUGUCUAGAACCAU

............((((((.(((...)))....)))))).......(((((((((((((((....)))))))))))))))..(((((((((..((....))..))))))))).............((((((...))))))..((...((((....((((..((((((.(((((.(((....)))...))).)).))))))...))))...)))).))..

>AJ251683 *Betula* *alba* 18S rRNA gene, 5.8S rRNA gene, 25S rRNA gene, internal transcribed spacer 1 (ITS1) and internal transcribed spacer 2 (ITS2)

UCGAAACCUGCCCAGCAGAACGACCCGUGAACCUGUUGAAACAACUGGGGGUGUGGGGCGAUCUCGCCCCUUGCCCCCGAACGGUAGGGAGACACUUGUGCAUCCCUGCCGAACAACGAACCCCGGCGCGGUCCGCGCCAAGGAACUUUAACGAAAGAGUGCCUCCGGCCGCCUCGGAAACGGUGUGCGUGCGGGAGGUGAAUCUUGUCUAGAACCAU

............((((((.(((...)))....)))))).......((((((((.((((((....)))))).))))))))..(((((((((..((....))..))))))))).............((((((...))))))..((...((((....((((..((((((.(((((.(((....)))...))).)).))))))...))))...)))).))..

>AM503889.2| *Betula* *pendula* ITS1 (partial), 5.8S rRNA gene, ITS2 and 28S rRNA gene (partial)

------CCUGCCCAGCAGAACGACCCGUGAACCUGUUGAAACAACUGGGGGUGUGGGGCGAUCUCGCCCCUUGCCCCCGAACGGUAGGGAGACACUUGUGCAUCCCUGCCGAACAACGAACCCCGGCGCGGUCCGCGCCAAGGAACUUUAACGAAAGAGUGCCUCCGGCCGCCUCGGAAACGGUGUGCGUGCGGGAGGUGAAUCUUGUCUAGAACCAU

------......((((((.(((...)))....)))))).......((((((((.((((((....)))))).))))))))..(((((((((..((....))..))))))))).............((((((...))))))..((...((((....((((..((((((.(((((.(((....)))...))).)).))))))...))))...)))).))..

>AB243915 *Betula* *apoiensis* genes for ITS1, 5.8S rRNA, ITS2, haplotype:ap17

UCGAAACCUGCCCAGCAGAACGACCCGUGAACCUGUUGAAACAAUUGGGGGCGGGGGGC--UCUCGCCCCUUGCCCCCGAACGGUAGGGAGACACUUGUGCAUCCCUGCCGAACAACGAACCCCGGCGCGGUCUGCGCCAAGGAACUUUAACGAAAGAGUGCCUCCCGCCGCCUCGGAAACGGUGUGCGUGCGGGAGGUGAAUCUUGUCUAGAACCAU

............((((((.(((...)))....)))))).......((((((((((((((--....))))))))))))))..(((((((((..((....))..))))))))).............((((((...))))))..((...((((....((((..((((((.(((((.(((....)))...))).)).))))))...))))...)))).))..

>AB243914 *Betula* *apoiensis* genes for ITS1, 5.8S rRNA, ITS2, haplotype:ap14

UCGAAACCUGCCCAGCAGAACGACCCGUGAACCUGUUGAAACAACUGGGGGCGGGGGGC--UCUCGCCCCUUGCCCCCGAACGGUAGGGAGACACUUGUGCAUCCCUGCCGAACAACGAACCCCGGCGCGGUCUGCGCCAAGGAACUUUAACGAAAGAGUGCCUCCCGCCGCCUCGGAAACGGUGUGCGUGCGGGAGGUGAAUCUUGUCUAGAACCAU

............((((((.(((...)))....)))))).......((((((((((((((--....))))))))))))))..(((((((((..((....))..))))))))).............((((((...))))))..((...((((....((((..((((((.(((((.(((....)))...))).)).))))))...))))...)))).))..

>AB243913 *Betula* *apoiensis* genes for ITS1, 5.8S rRNA, ITS2, haplotype:ap16

UCGAAACCUGCCCAGCAGAACGACCCGUGAACCUGUUGAAACAACUGGGGGCGGGGGGC--UCUCGCCCCUUGCCCCCGAACGGUAGGGAGACACUUGUGCAUCCCUGCCGAACAACGAACCCCGGCGCGGUCUGCGCCAAGGAACUUUAACGAAAGAGUGCCUCCGGCCGCCUCGGAAACGGUGUGCGUGCGGGAGGUGAAUCUUGUCUAGAACCAU

............((((((.(((...)))....)))))).......((((((((((((((--....))))))))))))))..(((((((((..((....))..))))))))).............((((((...))))))..((...((((....((((..((((((.(((((.(((....)))...))).)).))))))...))))...)))).))..

>AB243912 *Betula* *apoiensis* genes for ITS1, 5.8S rRNA, ITS2, haplotype:ap15

UCGAAACCUNNNNAGCAGAACGACCCGUGAACCUGUUGAAACAAUUGGGGGCGGGGGGC--UCUCGCCCCUUGCCCCCGAACGGUAGGGAGACACUUGUGCAUCCCUGCCGAACAACGAACCCCGGCGCGGUCUGCGCCAAGGAACUUUAACGAAAGAGUGCCUCCCGCCGCCUCGGAAACGGUGUGCGUGCGGGAGGUGAAUCUUGUCUAGAACCAU

.............(((((.(((...)))....)))))........((((((((((((((--....))))))))))))))..(((((((((..((....))..))))))))).............((((((...))))))..((...((((....((((..((((((.(((((.(((....)))...))).)).))))))...))))...)))).))..

>AB243911 *Betula* *apoiensis* genes for ITS1, 5.8S rRNA, ITS2, haplotype:ap12

UCGAAACCUGCCCAGCAGAACGACCCGUGAACCUGUUGAAACAACUGGGGGUGGGGGGCGAUCUCGCCCCUUGCCCUCGAACGGUAGGGAGACACUCGUGCAUCCCUGCCGAACAACGAACCCCGGCGCGGUCUGCGCCAAGGAACUUUAACGAAAGAGUGCCUCCGGCCGCCUCGGAAACGGUGUGCGUGCGGGAGGUGAAUCUUGUCUAGAACCAU

............((((((.(((...)))....)))))).......(((((((((((((((....)))))))))))))))..(((((((((..((....))..))))))))).............((((((...))))))..((...((((....((((..((((((.(((((.(((....)))...))).)).))))))...))))...)))).))..

>AB243910 *Betula* *apoiensis* genes for ITS1, 5.8S rRNA, ITS2, haplotype:ap10

UCGAAACCUGCCCAGCAGAACGACCCGUGAACCUGUUGAAACAACUGGGGGCGGGGGGCGAUCUCGCCCCUUGCCCUCGAACGGUAGGGAGACACUCGUGCAUCCCUGCCGAACAACGAACCCCGGCGCGGUCUGCGCCAAGGAACUUUAACGAAAGAGUGCCUCUGGCCGCCUCGGAAACGGUGUGCGUGCGGGAGGUGAAUCUUGUCUAGAACCAU

............((((((.(((...)))....)))))).......(((((((((((((((....)))))))))))))))..(((((((((..((....))..))))))))).............((((((...))))))..((...((((....((((..((((((.(((((.(((....)))...))).)).))))))...))))...)))).))..

>AB243909 *Betula* *apoiensis* genes for ITS1, 5.8S rRNA, ITS2, haplotype:ap11

UCGAAACCUGCCCAGCAGAACGACCCGUGAACCUGUUGAAACAACUGGGGGUGGGGGGCGAUCUCGCCCCUUGCCCCCGAACGGUAGGGAGACACUCGUGCAUCCCUGCCGAACAACGAACCCCGGCGCGGUCUGCGCCAAGGAACUUUAACGAAAGAGUGCCUCCUGCCGCCUCGGAAACGGUGUGCGUGCGGGAGGUGAAUCUUGUCUAGAACCAU

............((((((.(((...)))....)))))).......(((((((((((((((....)))))))))))))))..(((((((((..((....))..))))))))).............((((((...))))))..((...((((....((((..((((((.(((((.(((....)))...))).)).))))))...))))...)))).))..

>AB243908 *Betula* *apoiensis* genes for ITS1, 5.8S rRNA, ITS2, haplotype:ap13

UCGAAACCUGCCCAGCAGAACGACCCGUGAACCUGUUCAAACAACUGGGGGUGGGGGGCGAUCUCGCCCCUUGCCCCCGAACGGUAGGGAGACACUUGUGCAUCCCUGCCGAACAACGAACCCCGGCGCGGUCUGCGCCAAGGAACUUUAACGAAAGAGUGCCUCCCGCCGCCUCGGAAACGGUGUGCGUGCGGGAGGUGAAUCUUGUCUAGAACCAU

.............(((((.(((...)))....)))))........(((((((((((((((....)))))))))))))))..(((((((((..((....))..))))))))).............((((((...))))))..((...((((....((((..((((((.(((((.(((....)))...))).)).))))))...))))...)))).))..

>AB243907 *Betula* *apoiensis* genes for ITS1, 5.8S rRNA, ITS2, haplotype:ap5

UCGAAACCUGCCCAGCAGAACGACCCGUGAACCUGUUGAAACAACUGGGGGCGGGGGGCGAUCUCGCCCCGUGCCCUCGAACGGUAGGGAGACACUCGUGCAUCCCUGCCGAACAACGAACCCCGGCGCGGUCUGCGCCAAGGAACUUUAACGAAAGAGUGCCUCCGGCCGCCUCGGAAACGGUGUGCGUGCGGGAGGUGAAUCUUGUCUAGAACCAU

............((((((.(((...)))....)))))).......((((((((.((((((....)))))).))))))))..(((((((((..((....))..))))))))).............((((((...))))))..((...((((....((((..((((((.(((((.(((....)))...))).)).))))))...))))...)))).))..

>AB243906 *Betula* *apoiensis* genes for ITS1, 5.8S rRNA, ITS2, haplotype:ap9

UCGAAACCUGCCCAGCAGAACGACCCGUGAACCUGUUGAAACAACUGGGGGCGGGGGGCGAUCUCGCCCCUUGCCCUCGAACGGUAGGGAGACACUCGUGCAUCCCUGCCGAACAACGAACCCCGGCGCGGUCUGCGCCAAGGAACUUUAACGAAAGAGUGCCUCCGGCCGCCUCGGAAACGGUGUGCGUGCGGGAGGUGAAUCUUGUCUAGAACCAU

............((((((.(((...)))....)))))).......(((((((((((((((....)))))))))))))))..(((((((((..((....))..))))))))).............((((((...))))))..((...((((....((((..((((((.(((((.(((....)))...))).)).))))))...))))...)))).))..

>AB243905 *Betula* *apoiensis* genes for ITS1, 5.8S rRNA, ITS2, haplotype:ap8

UCGAAACCUGCCCAGCAGAACGACCCGUGAACCUGUUGAAACAACUGGGGGUGGGGGGCGAUCUCGCCCCUUGCCCUCGAACGGUAGGGAGACACUCGUGCAUCCCUGCCGAACAACGAACCCCGGCGCGGUCUGCGCCAAGGAACUUUAACGAAAGAGUGCCUCCGGCCGCCUCGGAAACGGUGUGCGUGCGGGAGGUGAAUCUUGUCUAGAACCAU

............((((((.(((...)))....)))))).......(((((((((((((((....)))))))))))))))..(((((((((..((....))..))))))))).............((((((...))))))..((...((((....((((..((((((.(((((.(((....)))...))).)).))))))...))))...)))).))..

>AB243904 *Betula* *apoiensis* genes for ITS1, 5.8S rRNA, ITS2, haplotype:ap7

UCGAAACCUGCCCAGCAGAACGACCCGUGAACCUGUUGAAACAACUGGGGGCGGGGGGCGAUCUCGCCCCUUGCCCUCGAACGGUAGGGAGACACUCGUGCAUCCCUGCCGAACAACGAACCCCGGCGCGGUCUGCGCCAAGGAACUUUAACGAAAGAGUGCCUCCGGCCGCCUCGGAAACGGUGUGCGUGCGGGGGGUGAAUCUUGUCUAGAACCAU

............((((((.(((...)))....)))))).......(((((((((((((((....)))))))))))))))..(((((((((..((....))..))))))))).............((((((...))))))..((...((((....((((..((((((.(((((.(((....)))...))).)).))))))...))))...)))).))..

>AB243903 *Betula* *apoiensis* genes for ITS1, 5.8S rRNA, ITS2, haplotype:ap6

UCGAAACCUGCCCAGCAGAACGACCCGUGAACCUGUUGAAACAACUGGGGGUGGGGGGCGAUCUCGCCCCUUGCCCUCGAACGGUAGGGAGACACUCGUGCAUCCCUGCCGAACAACGAACCCCGGCGCGGUCUGCGCCAAGGAACUUUAACGAAAGAGUGCCUCCGGCCGCCUCGGAAACGGUGUGCGUGCGGGAGGUGAAUCUUGUCUAGAACCAU

............((((((.(((...)))....)))))).......(((((((((((((((....)))))))))))))))..(((((((((..((....))..))))))))).............((((((...))))))..((...((((....((((..((((((.(((((.(((....)))...))).)).))))))...))))...)))).))..

>AB243902 *Betula* *apoiensis* genes for ITS1, 5.8S rRNA, ITS2, haplotype:ap3

UCGAAACCUGCCCAGUAGAACGACCCGUGAACCUGUUGAAACAACUGGGGGUGGGGGGCGAUCUCGCCCCUUGCCCUCGAACGGUAGGGAGACACUCGUGCAUCCCUGCCGAACAACGAACCCCGGCGCGGUCUGCGCCAAGGAACUUUAACGAAAGAGUGCCUCCGGCCGCCUCGGAAACGGUGUGCGUGCGGGAGGUGAAUCUUGUCUAGAACCAU

............((((((.(((...)))....)))))).......(((((((((((((((....)))))))))))))))..(((((((((..((....))..))))))))).............((((((...))))))..((...((((....((((..((((((.(((((.(((....)))...))).)).))))))...))))...)))).))..

>AB243901 *Betula* *apoiensis* genes for ITS1, 5.8S rRNA, ITS2, haplotype:ap2

UCGAAACCUGCCCAGCAGAACGACCCGUGAACCUGUUGAAACAACUGGGGGUGGGGGGCGAUCUCGCCCCUUGCCCUCGAACGGUAGGGAGACACUCGUGCAUCCCUGCCGAACAACGAACCCCGGCGCGGUCUGCGCCAAGGAACUUUAACGAAAGAGUGCCUCCGGCCGCCUCGGAAACGGUGUGCGUGCGGGAGGUGAAUCUUGUCUAGAACCAU

............((((((.(((...)))....)))))).......(((((((((((((((....)))))))))))))))..(((((((((..((....))..))))))))).............((((((...))))))..((...((((....((((..((((((.(((((.(((....)))...))).)).))))))...))))...)))).))..

>AB243900 *Betula* *apoiensis* genes for ITS1, 5.8S rRNA, ITS2, haplotype:ap1

UCGAAACCUGCCCAGCAGAACGACCCGUGAACCUGUUGAAACAACUGGGGGUGGGGGGCGAUCUCGCCCCUUGCCCUCGAACGGUAGGGAGACACUCGUGCAUCCCUGCCGAACAACGAACCCCGGCGCGGUCUGCGCCAAGGAACUUUAACGAAAGAGUGCCUCCGGCCGCCUCGGAAACGGUGUGCGUGCGGGAGGUGAAUCUUGUCUAGAACCAU

............((((((.(((...)))....)))))).......(((((((((((((((....)))))))))))))))..(((((((((..((....))..))))))))).............((((((...))))))..((...((((....((((..((((((.(((((.(((....)))...))).)).))))))...))))...)))).))..

>AB243899 *Betula* *apoiensis* genes for ITS1, 5.8S rRNA, ITS2, haplotype:ap4

UCGAAACCUGCCCAGCAGAACGACCCGUGAACCUGUUGAAACAACUGGGGGCGGGGGGCGAUCUCGCCCCGUGCCCUCGAACGGUAGGGAGACACUCGUGCAUCCCUGCUGAACAACGAACCCCGGCGCGGUCUGCGCCAAGGAACUUUAACGAAAGAGUGCCUCCGGCCGCCUCGGAAACGGUGUGCGUGCGGGAGGUGAAUCUUGUCUAGAACCAU

............((((((.(((...)))....)))))).......((((((((.((((((....)))))).))))))))..(((((((((..((....))..))))))))).............((((((...))))))..((...((((....((((..((((((.(((((.(((....)))...))).)).))))))...))))...)))).))..

>AB243898 *Betula* *ovalifolia* genes for ITS1, 5.8S rRNA, ITS2, haplotype:ov3

UCGAAACCUGCCCAGCAGAACGACCCGUGAACCUGUUGAAACAACUGGGGGUGGGGGGC--UCUCGCCCCUUGCCCCCGAACGGUAGGGAGACACUUGUGCAUCCCUGCCGAACAACGAACCCCGGCGCGGUCUGCGCCAAGGAACUUUAACGAAAGAGUGCCUCCCGCCGCCUCGGAAACGGUGUGCGUGCGGGAGGUGAAUCUUGUCUAGAACCAU

............((((((.(((...)))....)))))).......((((((((((((((--....))))))))))))))..(((((((((..((....))..))))))))).............((((((...))))))..((...((((....((((..((((((.(((((.(((....)))...))).)).))))))...))))...)))).))..

>AB243897 *Betula* *ovalifolia* genes for ITS1, 5.8S rRNA, ITS2, haplotype:ov2

UCGAAACCUGCCCAGCAGAACGACCCGUGAACCUGUUGAAACAACUGGGGGCGGGGGGC--UCUCGCCCCUUGCCCCCGAACGGUAGGGAGACACUUGUGCAUCCCUGCCGAACAACGAACCCCGGCGCGGUCUGCGCCAAGGAACUUUAACGAAAGAGUGCCUCCCGCCGCCUCGGAAACGGUGUGCGUGCGGGAGGUGAAUCUUGUCUAGAACCAU

............((((((.(((...)))....)))))).......((((((((((((((--....))))))))))))))..(((((((((..((....))..))))))))).............((((((...))))))..((...((((....((((..((((((.(((((.(((....)))...))).)).))))))...))))...)))).))..

>AB243895 *Betula* *middendorffii* genes for ITS1, 5.8S rRNA, ITS2, haplotype:mi

UCGAAACCUGCCCAGCAGAACGACCCGUGAACCUGUUGAAACAACUGGGGGUGGGGGGC--UCUCGCCCCUUGCCCCCGAACGGUAGGGAGACACUCGUGCAUCCCUGCCGAACAACGAACCCCGGCGCGGUCUGCGCCAAGGAACUUUAACGAAAGAGUGCCUCCCGCCGCCUCGGAAACGGUGUGCGUGCGGGAGGUGAAUCUUGUCUAGAACCAU

............((((((.(((...)))....)))))).......((((((((((((((--....))))))))))))))..(((((((((..((....))..))))))))).............((((((...))))))..((...((((....((((..((((((.(((((.(((....)))...))).)).))))))...))))...)))).))..

>AB243894 *Betula* *davurica* genes for ITS1, 5.8S rRNA, ITS2, haplotype:da2

UCGAAACCUGCCCAGCAGAACGACCCGUGAACCUGUUGAAACAACUGGGGGUGGGGGGCGAUCUCACCCCUUGCCCCCGAACGGUAGGGAGACACUUGUGCAUCCCUGCCGAACAACGAACCCCGGCGCGGUCUGCGCCNNGGAACUUUAACGAAAGAGUGCCUCCCGCCGCCUCGGAAACGGUGUGCGUGCGGGAGGUGAAUCUUGUCUAGAACCAU

............((((((.(((...)))....)))))).......(((((((((((((.(....).)))))))))))))..(((((((((..((....))..))))))))).............((((((...))))))..((...((((....((((..((((((.(((((.(((....)))...))).)).))))))...))))...)))).))..

>AB243893 *Betula* *davurica* genes for ITS1, 5.8S rRNA, ITS2, haplotype:da1

UCGAAACCUGCCCAGCAGAACGACCCGUGAACCUGUUGAAACAACUGGGGGUGGGGGGCGAUCUCGCCCCUUGCCCCCGAACGGUAGGGAGACACUUGUGCAUCCCUGCCGAACAACGAACCCCGGCGCGGUCUGCGCCAAGGAACUUUAACGAAAGAGUGCCUCCCGCCGCCUCGGAAACGGUGUGCGUGCGGGAGGUGAAUCUUGUCUAGAACCAU

............((((((.(((...)))....)))))).......(((((((((((((((....)))))))))))))))..(((((((((..((....))..))))))))).............((((((...))))))..((...((((....((((..((((((.(((((.(((....)))...))).)).))))))...))))...)))).))..

>AB243892 *Betula* *grossa* genes for ITS1, 5.8S rRNA, ITS2, haplotype:gr

UCGAAACCUGCCCAGCAGAACGACCCGUGAACCUGUUGAAACAACUGGGGGUGGGGGGCGAUCUCGCCCCUUGCCCCCGAACGGUAGGGAGACACUUGUGCAUCCCUGCCGAACAACGAACCCCGGCGCGGUCUGCGCCAAGGAACUUUAACGAAAGAGUGCCUCCGGCCGCCUCGGAAACGGUGUGCGUGCGGGAGGCGAAUCUUGUCUAGAACCAU

............((((((.(((...)))....)))))).......(((((((((((((((....)))))))))))))))..(((((((((..((....))..))))))))).............((((((...))))))..((...((((....((((..((((((.(((((.(((....)))...))).)).))))))...))))...)))).))..

>AB243891 *Betula* *platyphylla* genes for ITS1, 5.8S rRNA, ITS2, haplotype:pl

UCGAAACCUGCCCAGCAGAACGACCCGUGAACCUGUUGAAACAACUGGGGGUGUGGGGCGAUCUCGCCCCUUGCCCCCGAACGGUAGGGAGACACUUGUGCAUCCCUGCCGAACAACGAACCCCGGCGCGGUCCGCGCCAAGGAACUUUAACGAAAGAGUGCCUCCGGCCGCCUCGGAAACGGUGUGCGUGCGGGAGGUGAAUCUUGUCUAGAACCAU

............((((((.(((...)))....)))))).......((((((((.((((((....)))))).))))))))..(((((((((..((....))..))))))))).............((((((...))))))..((...((((....((((..((((((.(((((.(((....)))...))).)).))))))...))))...)))).))..

>AB243890 *Betula* *maximovicziana* genes for ITS1, 5.8S rRNA, ITS2, haplotype:ma

UCGAAACCUGCCCAGCAGAACGACCCGUGAACCUGUUGAAACAACUGGGGGUGGGGGGCGAUCUCGCCCCUUGCCCCCGAACGGUAGGGAGACACUCGUGCAUCCCUGCCGAACAACGAACCCCGGCGCGGUCUGCGCCAAGGAACUUUAACGAAAGAGUGCCUCCGGCCGCCUCGGAAACGGUGUGCGUGCGGGAGGUGAAUCUUGUCUAGAACCAU

............((((((.(((...)))....)))))).......(((((((((((((((....)))))))))))))))..(((((((((..((....))..))))))))).............((((((...))))))..((...((((....((((..((((((.(((((.(((....)))...))).)).))))))...))))...)))).))..

>AB243889 *Betula* *ermanii* genes for ITS1, 5.8S rRNA, ITS2, haplotype:er4

UCGAAACCUGCCCAGCAGAACGACCCGUGAACCUGUUGAAACAACUGGGGGCGGGGGGCGAUCUCGCCCCGUGCCCUCGAACGGUAGGGAGACACUCGUGCAUCCCUGCUGAACAACGAACCCCGGCGCGGUCUGCGCCAAGGAACUUUAACGAAAGAGUGCCUCCGGCCGCCUCGGAAACGGUGUGCGUGCGGGAGGUGAAUCUUGUCUAGAACCAU

............((((((.(((...)))....)))))).......((((((((.((((((....)))))).))))))))..(((((((((..((....))..))))))))).............((((((...))))))..((...((((....((((..((((((.(((((.(((....)))...))).)).))))))...))))...)))).))..

>AB243888 *Betula* *ermanii* genes for ITS1, 5.8S rRNA, ITS2, haplotype:er3

UCGAAACCUGCCCAGCAGAACGACCCGUGAACCUGUUGAAACAACUGGGGGCGGGGGGCGAUCUCGCCCCUUGCCCUCGAACGGUAGGGAGACACUCGUGCAUCCCUGCCGAACAACGAACCCCGGCGCGGUCUGCGCCAAGGAACUUUAACGAAAGAGUGCCUCCGGCCGCCUCGGAAACGGUGUGCGUGCGGGAGGUGAAUCUUGUCUAGAACCAU

............((((((.(((...)))....)))))).......(((((((((((((((....)))))))))))))))..(((((((((..((....))..))))))))).............((((((...))))))..((...((((....((((..((((((.(((((.(((....)))...))).)).))))))...))))...)))).))..

>AB243887 *Betula* *ermanii* genes for ITS1, 5.8S rRNA, ITS2, haplotype:er2

UCGAAACCUGCCCAGCAGAACGACCCGUGAACCUGUUGAAACAACUGGGGGUGGGGGGCGAUCUCGCCCCUUGCCCUCGAACGGUAGGGAGACACUCGUGCAUCCCUGCCGAACAACGAACCCCGGCGCGGUCUGCGCCAAGGAACUUUAACGAAAGAGUGCCUCCGGCCGCCUCGGAAACGGUGUGCGUGCGGGAGGUGAAUCUUGUCUAGAACCAU

............((((((.(((...)))....)))))).......(((((((((((((((....)))))))))))))))..(((((((((..((....))..))))))))).............((((((...))))))..((...((((....((((..((((((.(((((.(((....)))...))).)).))))))...))))...)))).))..

>AB243886 *Betula* *ermanii* genes for ITS1, 5.8S rRNA, ITS2, haplotype:er1

UCGAAACCUGCCCAGCAGAACGACCCGUGAACCUGUUGAAACAACUGGGGGUGGGGGGCGAUCUCGCCCCUUGCCCUCGAACGGUAGGGAGACACUCGUGCAUCCCUGCCGAACAACGAACCCCGGCGCGGUCUGCGCCAAGGAACUUUAACGAAAGAGUGCCUCCGGCCGCCUCGGAAACGGUGUGCGUGCGGGAGGUGAAUCUUGUCUAGAACCAU

............((((((.(((...)))....)))))).......(((((((((((((((....)))))))))))))))..(((((((((..((....))..))))))))).............((((((...))))))..((...((((....((((..((((((.(((((.(((....)))...))).)).))))))...))))...)))).))..

>AB243885 *Betula* *corylifolia* genes for ITS1, 5.8S rRNA, ITS2, haplotype:co2

UCGAAACCUGCCCAGCAGAACGACCCGUGAACCUGUUGAAACAACUGGGGGCGGGGGGCGAUCUCGCCCCGUGCCCCCGAACGGUAGGGAGACACUCGUGCAUCCCUGCCGAACAACGAACCCCGGCGCGGUCUGCGCCAAGGAACUUUAACGAAAGAAUGCCUCCGGCCGCCUCGGAAACGGUGUGCGUGCGGGAGGUGAAUCUUGUCUAGAACCAU

............((((((.(((...)))....)))))).......((((((((.((((((....)))))).))))))))..(((((((((..((....))..))))))))).............((((((...))))))..((...((((....((((..((((((.(((((.(((....)))...))).)).))))))...))))...)))).))..

>AB243884 *Betula* *corylifolia* genes for ITS1, 5.8S rRNA, ITS2, haplotype:co1

UCGAAACCUGCCCAGCAGAACGACCCGUGAACCUGUUGAAACAACUGGGGGCGGGGGGCGAUCUCGCCCCGUGCCCCCGAACGGUAGGGAGACACUCGUGCAUCCCUGCCGAACAACGAACCCCGGCGCGGUCUGCGCCAAGGAACUUUAACGAAAGAAUGCCUCCGGCCGCCUCGGAAACGGUGUGCGUGCGGGAGGUGAAUCUUGUCUAGAACCAU

............((((((.(((...)))....)))))).......((((((((.((((((....)))))).))))))))..(((((((((..((....))..))))))))).............((((((...))))))..((...((((....((((..((((((.(((((.(((....)))...))).)).))))))...))))...)))).))..

>AB243883 *Betula* *globispica* genes for ITS1, 5.8S rRNA, ITS2, haplotype:gl

UCGAAACCUGCCCAGCAGAACGACCCGUGAACAUGUUGAAACAACUGGGGGCGGGGGGCGAUCUCGCCCCGUGCCCCCGAACGGUAGGGAGACACUCGUGCAUCCCUGCCGAACAACGAACCCCGGCGCGGUCUGCGCCAAGGAACUUUAACGAAAGAGUGCCUCCGGCCGCCUCGGAAACGGUGUGCGUGCGGGAGGUGAAUCUUGUCUAGAACCAU

............(((((..(((...))).....))))).......((((((((.((((((....)))))).))))))))..(((((((((..((....))..))))))))).............((((((...))))))..((...((((....((((..((((((.(((((.(((....)))...))).)).))))))...))))...)))).))..

>AB243882 *Betula* *chichibuensis* genes for ITS1, 5.8S rRNA, ITS2, haplotype:ch2

UCGAAACCUGCCCAGCAGAAUGACCCGUGAACCUGUUGAAACAACUGGGGGCGGGGGGCGAUCUCGCCCCGUGCCCCCGAACGGUAGGGAGACACUUGUGCAUCCCUGCUGAACAACGAACCCCGGCGCGGUCUGCGCCAAGGAACUUUAACGAAAGAGUGCCUCCGGCCGCCUCGGAAACGGUGUGCGUGCGGGAGGUGAAUCUUGUCUAGAACCAU

............((((((.(((...)))....)))))).......((((((((.((((((....)))))).))))))))..(((((((((..((....))..))))))))).............((((((...))))))..((...((((....((((..((((((.(((((.(((....)))...))).)).))))))...))))...)))).))..

>AB243881 *Betula* *chichibuensis* genes for ITS1, 5.8S rRNA, ITS2, haplotype:ch1

UCGAAACCUGCCCAGCAGAAUGACCCGUGAACCUGUUGAAACAACUGGGGGCGGGGGGCGAUCUCGCCCCGUGCCCCCGAACGGUAGGGAGACACUUGUGCAUCCCUGCUGAACAACGAACCCCGGCGCGGUCUGCGCCAAGGAACUUUAACGAAAGAGUGCCUCCGGCCGCCUCGGAAACGGUGUGCGUGCGGUAGGUGAAUCUUGUCUAGAACCAU

............((((((.(((...)))....)))))).......((((((((.((((((....)))))).))))))))..(((((((((..((....))..))))))))).............((((((...))))))..((...((((....((((..((((.(.(((((.(((....)))...))).)).).))))...))))...)))).))..

>AB243880 *Betula* *schmidtii* genes for ITS1, 5.8S rRNA, ITS2, haplotype:sc

UCGAAACCUGCCCAGCAGAAUGACCCGUGAACUUGUUGAAACAACUGGGGGCGGGGGGCGAUCUCGCCCCGUGCCCCCGAACGGUAGGGAGACACUUGUGCAUCCCUGCCGAACAACGAACCCCGGCGCGGUCUGCGCCAAGGAACUUUAACGAAAGAGUGCCUCCAGCCGCCUCGGAAACGGUGUGCGUGCGGGAGGUGAAUCUUGUCUAGAACCAU

............((((((.(((...)))....)))))).......((((((((.((((((....)))))).))))))))..(((((((((..((....))..))))))))).............((((((...))))))..((...((((....((((..((((((.(((((.(((....)))...))).)).))))))...))))...)))).))..

>KT309028 *Betula* *occidentalis* isolate 2 5.8S ribosomal RNA gene and internal transcribed spacer 1, partial sequence; 5.8S ribosomal RNA gene, complete sequence; and internal transcribed spacer 2, partial sequence

UCGAAACCUGCCCAGCAGAACGACCCGUGAACCUGUUGAAACAACUGGGGGYGKGGGGCGAUCUCGCCCCUUGCCCCCGAACGGUAGGGAGACACUUGUGCAUCCCUGCCGAACAACGAACCCCGGCGCGGUCUGCGCCAAGGAAMUUUAACGAAAGAGUGCCUCCGGCCGCCUCGGAAACGGUGUGCGUGCGGGAGGUGAAUCUUGUCUAGAACCAY

............((((((.(((...)))....)))))).......(((((((((((((((....)))))))))))))))..(((((((((..((....))..))))))))).............((((((...))))))..((...((((....((((..((((((.(((((.(((....)))...))).)).))))))...))))...)))).))..

>KT309027 *Betula* *occidentalis* isolate 1 5.8S ribosomal RNA gene and internal transcribed spacer 1, partial sequence; 5.8S ribosomal RNA gene, complete sequence; and internal transcribed spacer 2, partial sequence

UCGAAACCUGCCCAGCAGAACGACCCGUGAACCUGUUGAAACAACUGGGGGUGGGGGGCGAUCUCGCCCCUUGCCCCCGAACGGUAGGGAGACACUUGUGCAUCCCUGCCGAACAACGAACCCCGGCGCGGUCUGCGCCAAGGAACUUUAACGAAAGAGUGCCUCCGGCCGCCUCGGAAACGGUGUGCGUGCGGGAGGUGAAUCUUGUCUAGAACCAU

............((((((.(((...)))....)))))).......(((((((((((((((....)))))))))))))))..(((((((((..((....))..))))))))).............((((((...))))))..((...((((....((((..((((((.(((((.(((....)))...))).)).))))))...))))...)))).))..

>KT309026 *Betula* *humilis* isolate 3 5.8S ribosomal RNA gene and internal transcribed spacer 1, partial sequence; 5.8S ribosomal RNA gene, complete sequence; and internal transcribed spacer 2, partial sequence

UCGAAACCUGCCCAGCAGAACGACCCGUGAACCUGUUGAAACAACUGGGGGUGGGGGGCGAUCUYGCCCCUUGCCCCCGAACGGUAGGGAGACACUCGUGCAUCCCUGCCGAACAACGAACCCCGGCGCGGUCUGCGCCAAGGAACUUUAACGAAAGAGUGCCUCCGGCCGCCUCGGAAACGGUGUGCGUGCGGGAGGUGAAUCUUGUCUAGAACCAU

............((((((.(((...)))....)))))).......(((((((((((((((....)))))))))))))))..(((((((((..((....))..))))))))).............((((((...))))))..((...((((....((((..((((((.(((((.(((....)))...))).)).))))))...))))...)))).))..

>KT309025 *Betula* *humilis* isolate 2 5.8S ribosomal RNA gene and internal transcribed spacer 1, partial sequence; 5.8S ribosomal RNA gene, complete sequence; and internal transcribed spacer 2, partial sequence

UCGAAACCUGCCCAGCAGAACGACCCGUGAACCUGUUGAAACAACUGGGGGUGGGGGGCGAUCUYGCCCCUUGCCCCCGAACGGUAGGGAGACACUCGUGCAUCCCUGCCGAACAACGAACCCCGGCGCGGUCUGCGCCAAGGAACUUUAACGAAAGAGUGCCUCCGGCCGCCUCGGAAACGGUGUGCGUGCGGGAGGUGAAUCUUGUCUAGAACCAU

............((((((.(((...)))....)))))).......(((((((((((((((....)))))))))))))))..(((((((((..((....))..))))))))).............((((((...))))))..((...((((....((((..((((((.(((((.(((....)))...))).)).))))))...))))...)))).))..

>KT309024 *Betula* *humilis* isolate 1 5.8S ribosomal RNA gene and internal transcribed spacer 1, partial sequence; 5.8S ribosomal RNA gene, complete sequence; and internal transcribed spacer 2, partial sequence

UCGAAACCUGCCCAGCAGAACGACCCGUGAACCUGUUGAAACAACUGGGGGUGGGGGGCGAUCUCGCCCCUUGCCCCCGAACGGUAGGGAGACACUCGUGCAUCCCUGCCGAACAACGAACCCCGGCGCGGUCUGCGCCAAGGAACUUUAACGAAAGAGUGCCUCCGGCCGCCUCGGAAACGGUGUGCGUGCGGGAGGUGAAUCUUGUCUAGAACCAU

............((((((.(((...)))....)))))).......(((((((((((((((....)))))))))))))))..(((((((((..((....))..))))))))).............((((((...))))))..((...((((....((((..((((((.(((((.(((....)))...))).)).))))))...))))...)))).))..

>KT309023 *Betula* *ovalifolia* isolate 2 5.8S ribosomal RNA gene and internal transcribed spacer 1, partial sequence; 5.8S ribosomal RNA gene, complete sequence; and internal transcribed spacer 2, partial sequence

UCGAAACCUGCCCAGCAGAACGACCCGUGAACCUGUUGAAACAACUGGGGGCGGGGGGC--UCUCGCCCCUUGCCCCCGAACGGUAGGGAGACACUUGUGCAUCCCUGCCGAACAACGAACCCCGGCGCGGUCUGCGCCAAGGAACUUUAACGAAAGAGUGCCUCCCGCCGCCUCGGAAACGGUGUGCGUGCGGGAGGUGAAUCUUGUCUAGAACCAU

............((((((.(((...)))....)))))).......((((((((((((((--....))))))))))))))..(((((((((..((....))..))))))))).............((((((...))))))..((...((((....((((..((((((.(((((.(((....)))...))).)).))))))...))))...)))).))..

>KT309022 *Betula* *ovalifolia* isolate 1 5.8S ribosomal RNA gene and internal transcribed spacer 1, partial sequence; 5.8S ribosomal RNA gene, complete sequence; and internal transcribed spacer 2, partial sequence

UCGAAACCUGCCCAGCAGAACGACCCGUGAACCUGUUGAAACAACUGGGGGCGGGGGGC--UCUCGCCCCUUGCCCCCGAACGGUAGGGAGACACUUGUGCAUCCCUGCCGAACAACGAACCCCGGCGCGGUCUGCGCCAAGGAACUUUAACGAAAGAGUGCCUCCCGCCGCCUCGGAAACGGUGUGCGUGCGGGAGGUGAAUCUUGUCUAGAACCAU

............((((((.(((...)))....)))))).......((((((((((((((--....))))))))))))))..(((((((((..((....))..))))))))).............((((((...))))))..((...((((....((((..((((((.(((((.(((....)))...))).)).))))))...))))...)))).))..

>KT309021 *Betula* *pumila* 5.8S ribosomal RNA gene and internal transcribed spacer 1, partial sequence; 5.8S ribosomal RNA gene, complete sequence; and internal transcribed spacer 2, partial sequence

UCGAAACCUGCCCAGCAGAACGACCCGUGAACCUGUUGAAACAACUGGGGGUGGGGGGC--UCUCGCCCCUUGCCCCCGAACGGUAGGGAGACACUUGUGCAUCCCUGCCGAACAACGAACCCCGGCGCGGUCUGCGCCAAGGAACUUUAACGAAAGAGUGCCUCCGGCCGCCUCGGAAACGGUGUGCGUGCGGGAGGUGAAUCUUGUCUAGAACCAU

............((((((.(((...)))....)))))).......((((((((((((((--....))))))))))))))..(((((((((..((....))..))))))))).............((((((...))))))..((...((((....((((..((((((.(((((.(((....)))...))).)).))))))...))))...)))).))..

>KT309020 *Betula* *nana* isolate 2 5.8S ribosomal RNA gene and internal transcribed spacer 1, partial sequence; 5.8S ribosomal RNA gene, complete sequence; and internal transcribed spacer 2, partial sequence

UCGAAACCUGCCCAGCAGAACGACCCGUGAACCUGUUGAAACAACUGGGGGUGGGGGGCGAUCUCGCCCCUUGYCCCCGAACGGUAGGGAGACACUUGUGCAUCCCUGCCGAACAACGAACCCCGGCGCGGUCUGCGCCAAGGAACUUUAACGAAAGAGUGCCUCCGGCCGCCUCGGAAACGGUGUGCGUGCGGGAGGUGAAUCUUGUCUAGAACCAU

............((((((.(((...)))....)))))).......(((((((((((((((....)))))))))))))))..(((((((((..((....))..))))))))).............((((((...))))))..((...((((....((((..((((((.(((((.(((....)))...))).)).))))))...))))...)))).))..

>KT309019 *Betula* *nana* subsp. *exilis* 5.8S ribosomal RNA gene and internal transcribed spacer 1, partial sequence; 5.8S ribosomal RNA gene, complete sequence; and internal transcribed spacer 2, partial sequence

UCGAAACCUGCCCAGCAGAACGACCCGUGAACCUGUUGAAACAACUGGGGGUGGGGGGC--UCUCGCCCCUUGCCCCCGAACGGUAGGGAGACACUUGUGCAUCCCUGCCGAACAACGAACCCCGGCGCGGUCUGCGCCAAGGAACUUUAACGAAAGAGUGCCUCCGGCCGCCUCGGAAACGGUGUGCGUGCGGGAGGUGAAUCUUGUCUAGAACCAU

............((((((.(((...)))....)))))).......((((((((((((((--....))))))))))))))..(((((((((..((....))..))))))))).............((((((...))))))..((...((((....((((..((((((.(((((.(((....)))...))).)).))))))...))))...)))).))..

>KT309018 *Betula* *nana* isolate 1 5.8S ribosomal RNA gene and internal transcribed spacer 1, partial sequence; 5.8S ribosomal RNA gene, complete sequence; and internal transcribed spacer 2, partial sequence

UCGAAACCUGCCCAGCAGAACGACCCGUGAACCUGUUGAAACAACUGGGGGUGGGGGGCGAUCUCGCCCCUUGCCCCCGAACGGUAGGGAGACACUUGUGCAUCCCUGCCGAACAACGAACCCCGGCGCGGUCUGCGCCAAGGAACUUUAACGAAAGAGUGCCUCCGGCCGCCUCGGAAACGGUGUGCGUGCGGGAGGUGAAUCUUGUCUAGAACCAU

............((((((.(((...)))....)))))).......(((((((((((((((....)))))))))))))))..(((((((((..((....))..))))))))).............((((((...))))))..((...((((....((((..((((((.(((((.(((....)))...))).)).))))))...))))...)))).))..

>KT309017 *Betula* *glandulosa* isolate 2 5.8S ribosomal RNA gene and internal transcribed spacer 1, partial sequence; 5.8S ribosomal RNA gene, complete sequence; and internal transcribed spacer 2, partial sequence

UCGAAACCUGCCCAGCAGAACGACCCGUGAACCUGUUGAAACAACUGGGGGCGGGGGGCGAUCUCGCCCCUUGCCCCCGAACGGUAGGGAGACACUUGUGCAUCCCUGCCGAACAACGAACCCCGGCGCGGUCUGCGCCAAGGAACUUUAACGAAAGAGUGCCUCCGGCCGCCUCGGAAACGGUGUGCGUGCGGGAGGUGAAUCUUGUCUAGAACCAC

............((((((.(((...)))....)))))).......(((((((((((((((....)))))))))))))))..(((((((((..((....))..))))))))).............((((((...))))))..((...((((....((((..((((((.(((((.(((....)))...))).)).))))))...))))...)))).))..

>KT309016 *Betula* *cordifolia* isolate 2 5.8S ribosomal RNA gene and internal transcribed spacer 1, partial sequence; 5.8S ribosomal RNA gene, complete sequence; and internal transcribed spacer 2, partial sequence

UCGAAACCUGCCCAGCAGAACGACCCGUGAACCUGUUGAAACAACUGGGGGCGGGGGGCGAUCUCGCCCCUUGCCCCCGAACGGUAGGGAGACACUUGUGCAUCCCUGCCGAACAACGAACCCCGGCGCGGUCUGCGCCAAGGAACUUUAACGAAAGAGUGCCUCCGGCCGCCUCGGAAACGGUGUGCGUGCGGGAGGUGAAUCUUGUCUAGAACCAU

............((((((.(((...)))....)))))).......(((((((((((((((....)))))))))))))))..(((((((((..((....))..))))))))).............((((((...))))))..((...((((....((((..((((((.(((((.(((....)))...))).)).))))))...))))...)))).))..

>KT309015 *Betula* *cordifolia* isolate 1 5.8S ribosomal RNA gene and internal transcribed spacer 1, partial sequence; 5.8S ribosomal RNA gene, complete sequence; and internal transcribed spacer 2, partial sequence

UCGAAACCUGCCCAGCAGAACGACCCGUGAACCUGUUGAAACAACUGGGGGCGGGGGGCGAUCUCGCCCCUUGCCCCCGAACGGUAGGGAGACACUUGUGCAUCCCUGCCGAACAACGAACCCCGGCGCGGUCUGCGCCAAGGAACUUUAACGAAAGAGUGCCUCCGGCCGCCUCGGAAACGGUGUGCGUGCGGGAGGUGAAUCUUGUCUAGAACCAU

............((((((.(((...)))....)))))).......(((((((((((((((....)))))))))))))))..(((((((((..((....))..))))))))).............((((((...))))))..((...((((....((((..((((((.(((((.(((....)))...))).)).))))))...))))...)))).))..

>KT309014 *Betula* *papyrifera* var. *commutata* 5.8S ribosomal RNA gene and internal transcribed spacer 1, partial sequence; 5.8S ribosomal RNA gene, complete sequence; and internal transcribed spacer 2, partial sequence

UCGAAACCUGCCCAGCAGAACGACCCGUGAACCUGUUGAAACAACUGGGGGUGGGGGGCGAUCUCGCCCCUUGCCCCCGAACGGUAGGGAGACACUUGUGCAUCCCUGCCGAACAACGAACCCCGGCGCGGUCUGCGCCAAGGAACUUUAACGAAAGAGUGCCUCCGGCCGCCUCGGAAACGGUGUGCGUGCGGGAGGUGAAUCUUGUCUAGAACCAU

............((((((.(((...)))....)))))).......(((((((((((((((....)))))))))))))))..(((((((((..((....))..))))))))).............((((((...))))))..((...((((....((((..((((((.(((((.(((....)))...))).)).))))))...))))...)))).))..

>KT309013 *Betula* *papyrifera* isolate 3 5.8S ribosomal RNA gene and internal transcribed spacer 1, partial sequence; 5.8S ribosomal RNA gene, complete sequence; and internal transcribed spacer 2, partial sequence

UCGAAACCUGCCCAGCAGAACGACCCGUGAACCUGUUGAAACAACUGGGGGUGKGGGGCGAUCUCGCCCCUUGCCCCCGAACGGUAGGGAGACACUUGUGCAUCCCUGCCGAACAACGAACCCCGGCGCGGUCCGCGCCAAGGAACUUUAACGAAAGAGUGCCUCCGGCCGCCUCGGAAACGGUGUGCGUGCGGGAGGUGAAUCUUGUCUAGAACCAU

............((((((.(((...)))....)))))).......((((((((.((((((....)))))).))))))))..(((((((((..((....))..))))))))).............((((((...))))))..((...((((....((((..((((((.(((((.(((....)))...))).)).))))))...))))...)))).))..

>KT309012 *Betula* *papyrifera* isolate 2 5.8S ribosomal RNA gene and internal transcribed spacer 1, partial sequence; 5.8S ribosomal RNA gene, complete sequence; and internal transcribed spacer 2, partial sequence

UCGAAACCUGCCCGGCAGAACGACCCGUGAACCUGUUGAAACAACUGGGGGUGGGGGGCGAUCUCGCCCCUUGCCCCCGAACGGUAGGGAGACACUUGUGCAUCCCUGCCGAACAACGAACCCCGGCGCGGUCUGCGCCAAGGAACUUUAACGAAAGAGUGCCUCCCGCCGCCUCGGAAACGGUGUGCGUGCGGGAGGUGAAUCUUGUCUAGAACCAU

............((((((.(((...)))....)))))).......(((((((((((((((....)))))))))))))))..(((((((((..((....))..))))))))).............((((((...))))))..((...((((....((((..((((((.(((((.(((....)))...))).)).))))))...))))...)))).))..

>KT309011 *Betula* *papyrifera* isolate 1 5.8S ribosomal RNA gene and internal transcribed spacer 1, partial sequence; 5.8S ribosomal RNA gene, complete sequence; and internal transcribed spacer 2, partial sequence

UCGAAACCUGCCCGGCAGAACGACCCGUGAACCUGUUGAAACAACUGGGGGUGGGGGGCGAUCUCGCCCCUUGCCCCCGAACGGUAGGGAGACACUUGUGCAUCCCUGCCGAACAACGAACCCCGGCGCGGUCUGCGCCAAGGAACUUUAACGAAAGAGUGCCUCCCGCCGCCUCGGAAACGGUGUGCGUGCGGGAGGUGAAUCUUGUCUAGAACCAU

............((((((.(((...)))....)))))).......(((((((((((((((....)))))))))))))))..(((((((((..((....))..))))))))).............((((((...))))))..((...((((....((((..((((((.(((((.(((....)))...))).)).))))))...))))...)))).))..

>KT309010 *Betula* *populifolia* isolate 3 5.8S ribosomal RNA gene and internal transcribed spacer 1, partial sequence; 5.8S ribosomal RNA gene, complete sequence; and internal transcribed spacer 2, partial sequence

UCGAAACCUGCCCAGCAGAACGACCCGUGAACCUGUUGAAACAACUGGGGGYGGGGGGCGAUCUCGCCCCUUGYCCCCGAACGGUAGGGAGACACUUGUGCAUCCCUGCCGAACAACGAACCCCGGCGCGGUCYGCGCCAAGGAACUUUAACGAAAGAGUGCCUCCGGCCGCCUCGGAAACGGUGUGCGUGCGGGAGGUGAAUCUUGUCUAGAACCAU

............((((((.(((...)))....)))))).......(((((((((((((((....)))))))))))))))..(((((((((..((....))..))))))))).............((((((...))))))..((...((((....((((..((((((.(((((.(((....)))...))).)).))))))...))))...)))).))..

>KT309009 *Betula* *populifolia* isolate 2 5.8S ribosomal RNA gene and internal transcribed spacer 1, partial sequence; 5.8S ribosomal RNA gene, complete sequence; and internal transcribed spacer 2, partial sequence

UCGAAACCUGCCCAGCAGAACGACCCGUGAACCUGUUGAAACAACUGGGGGYGGGGGGCGAUCUCGCCCCUUGYCCCCGAACGGUAGGGAGACACUUGUGCAUCCCUGCCGAACAACGAACCCCGGCGCGGUCCGCGCCAAGGAACUUUAACGAAAGAGUGCCUCCGGCCGCCUCGGAAACGGUGUGCGUGCGGGAGGUGAAUCUUGUCUAGAACCAU

............((((((.(((...)))....)))))).......(((((((((((((((....)))))))))))))))..(((((((((..((....))..))))))))).............((((((...))))))..((...((((....((((..((((((.(((((.(((....)))...))).)).))))))...))))...)))).))..

>KT309008 *Betula* *pendula* subsp. *mandshurica* isolate 5 5.8S ribosomal RNA gene and internal transcribed spacer 1, partial sequence; 5.8S ribosomal RNA gene, complete sequence; and internal transcribed spacer 2, partial sequence

UCGAAACCUGCCCAGCAGAACGACCCGUGAACCUGUUGAAACAACUGGGGGUGUGGGGCGAUCUCGCCCCUUGCCCCCGAACGGUAGGGAGACACUUGUGCAUCCCUGCCGAACAACGAACCCCGGCGCGGUCCGCGCCAAGGAACUUUAACGAAAGAGUGCCUCCGGCCGCCUCGGAAACGGUGUGCGUGCGGGAGGUGAAUCUUGUCUAGAACCAU

............((((((.(((...)))....)))))).......((((((((.((((((....)))))).))))))))..(((((((((..((....))..))))))))).............((((((...))))))..((...((((....((((..((((((.(((((.(((....)))...))).)).))))))...))))...)))).))..

>KT309007 *Betula* *pendula* subsp. *pendula* isolate 6 5.8S ribosomal RNA gene and internal transcribed spacer 1, partial sequence; 5.8S ribosomal RNA gene, complete sequence; and internal transcribed spacer 2, partial sequence

UCGAAACCUGCCCAGCAGAACGACCCGUGAACCUGUUGAAACAACUGGGGGUGUGGGGCGAUCUCGCCCCUUGCCCCCGAACGGUAGGGAGACACUUGUGCAUCCCUGCCGAACAACGAACCCCGGCGCGGUCCGCGCCAAGGAACUUUAACGAAAGAGUGCCUCCGGCCGCCUCGGAAACGGUGUGCGUGCGGGAGGUGAAUCUUGUCUAGAACCAU

............((((((.(((...)))....)))))).......((((((((.((((((....)))))).))))))))..(((((((((..((....))..))))))))).............((((((...))))))..((...((((....((((..((((((.(((((.(((....)))...))).)).))))))...))))...)))).))..

>KT309006 *Betula* *pendula* subsp. *pendula* isolate 5 5.8S ribosomal RNA gene and internal transcribed spacer 1, partial sequence; 5.8S ribosomal RNA gene, complete sequence; and internal transcribed spacer 2, partial sequence

UCGAAACCUGCCCAGCAGAACGACCCGUGAACCUGUUGAAACAACUGGGGGUGUGGGGCGAUCUCGCCCCUUGCCCCCGAACGGUAGGGAGACACUUGUGCAUCCCUGCCGAACAACGAACCCCGGCGCGGUCCGCGCCAAGGAACUUUAACGAAAGAGUGCCUCCGGCCGCCUCGGAAACGGUGUGCGUGCGGGAGGUGAAUCUUGUCUAGAACCAU

............((((((.(((...)))....)))))).......((((((((.((((((....)))))).))))))))..(((((((((..((....))..))))))))).............((((((...))))))..((...((((....((((..((((((.(((((.(((....)))...))).)).))))))...))))...)))).))..

>KT309005 *Betula* *pendula* subsp. *mandshurica* isolate 4 5.8S ribosomal RNA gene and internal transcribed spacer 1, partial sequence; 5.8S ribosomal RNA gene, complete sequence; and internal transcribed spacer 2, partial sequence

UCGAAACCUGCCCAGCAGAACGACCCGUGAACCUGUUGAAACAACUGGGGGUGUGGGGCGAUCUCGCCCCUUGCCCCCGAACGGUAGGGAGACACUUGUGCAUCCCUGCCGAACAACGAACCCCGGCGCGGUCCGCGCCAAGGAACUUUAACGAAAGAGUGCCUCCGGCCGCCUCGGAAACGGUGUGCGUGCGGGAGGUGAAUCUUGUCUAGAACCAU

............((((((.(((...)))....)))))).......((((((((.((((((....)))))).))))))))..(((((((((..((....))..))))))))).............((((((...))))))..((...((((....((((..((((((.(((((.(((....)))...))).)).))))))...))))...)))).))..

>KT309004 *Betula* *pendula* subsp. *szechuanica* isolate 3 5.8S ribosomal RNA gene and internal transcribed spacer 1, partial sequence; 5.8S ribosomal RNA gene, complete sequence; and internal transcribed spacer 2, partial sequence

UCGAAACCUGCCCAGCAGAACGACCCGUGAACCUGUUGAAACAACUGGGGGUGUGGGGCGAUCUCGCCCCUUGCCCCCGAACGGUAGGGAGACACUUGUGCAUCCCUGCCGAACAACGAACCCCGGCGCGGUCCGCGCCAAGGAACUUUAACGAAAGAGUGCCUCCGGCCGCCUCGGAAACGGUGUGCGUGCGGGAGGUGAAUCUUGUCUAGAACCAU

............((((((.(((...)))....)))))).......((((((((.((((((....)))))).))))))))..(((((((((..((....))..))))))))).............((((((...))))))..((...((((....((((..((((((.(((((.(((....)))...))).)).))))))...))))...)))).))..

>KT309003 *Betula* *pendula* subsp. *szechuanica* isolate 2 5.8S ribosomal RNA gene and internal transcribed spacer 1, partial sequence; 5.8S ribosomal RNA gene, complete sequence; and internal transcribed spacer 2, partial sequence

UCGAAACCUGCCCAGCAGAACGACCCGUGAACCUGUUGAAACAACUGGGGGUGUGGGGCGAUCUCGCCCCUUGCCCCCGAACGGUAGGGAGACACUUGUGCAUCCCUGCCGAACAACGAACCCCGGCGCGGUCCGCGCCAAGGAACUUUAACGAAAGAGUGCCUCCGGCCGCCUCGGAAACGGUGUGCGUGCGGGAGGUGAAUCUUGUCUAGAACCAU

............((((((.(((...)))....)))))).......((((((((.((((((....)))))).))))))))..(((((((((..((....))..))))))))).............((((((...))))))..((...((((....((((..((((((.(((((.(((....)))...))).)).))))))...))))...)))).))..

>KT309002 *Betula* *pendula* subsp. *pendula* isolate 4 5.8S ribosomal RNA gene and internal transcribed spacer 1, partial sequence; 5.8S ribosomal RNA gene, complete sequence; and internal transcribed spacer 2, partial sequence

UCGAAACCUGCCCAGCAGAACGACCCGUGAACCUGUUGAAACAACUGGGGGUGUGGGGCGAUCUCGCCCCUUGCCCCCGAACGGUAGGGAGACACUUGUGCAUCCCUGCCGAACAACGAACCCCGGCGCGGUCCGCGCCAAGGAACUUUAACGAAAGAGUGCCUCCGGCCGCCUCGGAAACGGUGUGCGUGCGGGAGGUGAAUCUUGUCUAGAACCAU

............((((((.(((...)))....)))))).......((((((((.((((((....)))))).))))))))..(((((((((..((....))..))))))))).............((((((...))))))..((...((((....((((..((((((.(((((.(((....)))...))).)).))))))...))))...)))).))..

>KT309001 *Betula* *pendula* subsp. *pendula* isolate 3 5.8S ribosomal RNA gene and internal transcribed spacer 1, partial sequence; 5.8S ribosomal RNA gene, complete sequence; and internal transcribed spacer 2, partial sequence

UCGAAACCUGCCCAGCAGAACGACCCGUGAACCUGUUGAAACAACUGGGGGUGUGGGGCGAUCUCGCCCCUUGCCCCCGAACGGUAGGGAGACACUUGUGCAUCCCUGCCGAACAACGAACCCCGGCGCGGUCCGCGCCAAGGAACUUUAACGAAAGAGUGCCUCCGGCCGCCUCGGAAACGGUGUGCGUGCGGGAGGUGAAUCUUGUCUAGAACCAU

............((((((.(((...)))....)))))).......((((((((.((((((....)))))).))))))))..(((((((((..((....))..))))))))).............((((((...))))))..((...((((....((((..((((((.(((((.(((....)))...))).)).))))))...))))...)))).))..

>KT309000 *Betula* *pendula* subsp. *pendula* isolate 2 5.8S ribosomal RNA gene and internal transcribed spacer 1, partial sequence; 5.8S ribosomal RNA gene, complete sequence; and internal transcribed spacer 2, partial sequence

UCGAAACCUGCCCAGCAGAACGACCCGUGAACCUGUUGAAACAACUGGGGGUGUGGGGCGAUCUCGCCCCUUGCCCCCGAACGGUAGGGAGACACUUGUGCAUCCCUGCCGAACAACGAACCCCGGCGCGGUCCGCGCCAAGGAACUUUAACGAAAGAGUGCCUCCGGCCGCCUCGGAAACGGUGUGCGUGCGGGAGGUGAAUCUUGUCUAGAACCAU

............((((((.(((...)))....)))))).......((((((((.((((((....)))))).))))))))..(((((((((..((....))..))))))))).............((((((...))))))..((...((((....((((..((((((.(((((.(((....)))...))).)).))))))...))))...)))).))..

>KT308999 *Betula* *pendula* subsp. *mandshurica* isolate 3 5.8S ribosomal RNA gene and internal transcribed spacer 1, partial sequence; 5.8S ribosomal RNA gene, complete sequence; and internal transcribed spacer 2, partial sequence

UCGAAACCUGCCCAGCAGAACGACCCGUGAACCUGUUGAAACAACUGGGGGUGUGGGGCGAUCUCGCCCCUUGCCCCCGAACGGUAGGGAGACACUUGUGCAUCCCUGCCGAACAACGAACCCCGGCGCGGUCCGCGCCAAGGAACUUUAACGAAAGAGUGCCUCCGGCCGCCUCGGAAACGGUGUGCGUGCGGGAGGUGAAUCUUGUCUAGAACCAU

............((((((.(((...)))....)))))).......((((((((.((((((....)))))).))))))))..(((((((((..((....))..))))))))).............((((((...))))))..((...((((....((((..((((((.(((((.(((....)))...))).)).))))))...))))...)))).))..

>KT308998 *Betula* *pendula* subsp. *pendula* isolate 1 5.8S ribosomal RNA gene and internal transcribed spacer 1, partial sequence; 5.8S ribosomal RNA gene, complete sequence; and internal transcribed spacer 2, partial sequence

UCGAAACCUGCCCAGCAGAACGACCCGUGAACCUGUUGAAACAACUGGGGGUGUGGGGCGAUCUCGCCCCUUGCCCCCGAACGGUAGGGAGACACUUGUGCAUCCCUGCCGAACAACGAACCCCGGCGCGGUCCGCGCCAAGGAACUUUAACGAAAGAGUGCCUCCGGCCGCCUCGGAAACGGUGUGCGUGCGGGAGGUGAAUCUUGUCUAGAACCAU

............((((((.(((...)))....)))))).......((((((((.((((((....)))))).))))))))..(((((((((..((....))..))))))))).............((((((...))))))..((...((((....((((..((((((.(((((.(((....)))...))).)).))))))...))))...)))).))..

>KT308997 *Betula* *pendula* subsp. *szechuanica* isolate 1 5.8S ribosomal RNA gene and internal transcribed spacer 1, partial sequence; 5.8S ribosomal RNA gene, complete sequence; and internal transcribed spacer 2, partial sequence

UCGAAACCUGCCCAGCAGAACGACCCGUGAACCUGUUGAAACAACUGGGGGUGUGGGGCGAUCUCGCCCCUUGCCCCCGAACGGUAGGGAGACACUUGUGCAUCCCUGCCGAACAACGAACCCCGGCGCGGUCCGCGCCAAGGAACUUUAACGAAAGAGUGCCUCCGGCCGCCUCGGAAACGGUGUGCGUGCGGGAGGUGAAUCUUGUCUAGAACCAU

............((((((.(((...)))....)))))).......((((((((.((((((....)))))).))))))))..(((((((((..((....))..))))))))).............((((((...))))))..((...((((....((((..((((((.(((((.(((....)))...))).)).))))))...))))...)))).))..

>KT308996 *Betula* *pendula* subsp. *mandshurica* isolate 2 5.8S ribosomal RNA gene and internal transcribed spacer 1, partial sequence; 5.8S ribosomal RNA gene, complete sequence; and internal transcribed spacer 2, partial sequence

UCGAAACCUGCCCAGCAGAACGACCCGUGAACCUGUUGAAACAACUGGGGGUGUGGGGCGAUCUCGCCCCUUGCCCCCGAACGGUAGGGAGACACUUGUGCAUCCCUGCCGAACAACGAACCCCGGCGCGGUCCGCGCCAAGGAACUUUAACGAAAGAGUGCCUCCGGCCGCCUCGGAAACGGUGUGCGUGCGGGAGGUGAAUCUUGUCUAGAACCAU

............((((((.(((...)))....)))))).......((((((((.((((((....)))))).))))))))..(((((((((..((....))..))))))))).............((((((...))))))..((...((((....((((..((((((.(((((.(((....)))...))).)).))))))...))))...)))).))..

>KT308995 *Betula* *glandulosa* isolate 1 5.8S ribosomal RNA gene and internal transcribed spacer 1, partial sequence; 5.8S ribosomal RNA gene, complete sequence; and internal transcribed spacer 2, partial sequence

UCGAAACCUGCCCRGCAGAACGACCCGUGAACCUGUUGAAACAACUGGGGGUGGGGGGCGAUCUCGCCCCUUGCCCCCGAACGGUAGGGAGACACUUGUGCAUCCCUGCCGAACAACGAACCCCGGCGCGGUCUGCGCCAAGGAACUUUAACGAAAGAGUGCCUCCCGCCGCCUCGGAAACGGUGUGCGUGCGGGAGGUGAAUCUUGUCUAGAACCAU

............((((((.(((...)))....)))))).......(((((((((((((((....)))))))))))))))..(((((((((..((....))..))))))))).............((((((...))))))..((...((((....((((..((((((.(((((.(((....)))...))).)).))))))...))))...)))).))..

>KT308994 *Betula* *populifolia* isolate 1 5.8S ribosomal RNA gene and internal transcribed spacer 1, partial sequence; 5.8S ribosomal RNA gene, complete sequence; and internal transcribed spacer 2, partial sequence

UCGAAACCUGCCCAGCAGAACGACCCGUGAACCUGUUGAAACAACUGGGGGYGGGGGGCGAUCUCGCCCCUUGYCCCCGAACGGUAGGGAGACACUUGUGCAUCCCUGCCGAACAACGAACCCCGGCGCGGUCCGCGCCAAGGAACUUUAACGAAAGAGUGCCUCCGGCCGCCUCGGAAACGGUGUGCGUGCGGGAGGUGAAUCUUGUCUAGAACCAU

............((((((.(((...)))....)))))).......(((((((((((((((....)))))))))))))))..(((((((((..((....))..))))))))).............((((((...))))))..((...((((....((((..((((((.(((((.(((....)))...))).)).))))))...))))...)))).))..

>KT308993 *Betula* *obscura* 5.8S ribosomal RNA gene and internal transcribed spacer 1, partial sequence; 5.8S ribosomal RNA gene, complete sequence; and internal transcribed spacer 2, partial sequence

UCGAAACCUGCCCAGCAGAACGACCCGUGAACCUGUUGAAACAACUGGGGGUGUGGGGCGAUCUCGCCCCUUGCCCCCGAACGGUAGGGAGACACUUGUGCAUCCCUGCCGAACAACGAACCCCGGCGCGGUCCGCGCCAAGGAACUUUAACGAAAGAGUGCCUCCGGCCGCCUCGGAAACGGUGUGCGUGCGGGAGGUGAAUCUUGUCUAGAACCAU

............((((((.(((...)))....)))))).......((((((((.((((((....)))))).))))))))..(((((((((..((....))..))))))))).............((((((...))))))..((...((((....((((..((((((.(((((.(((....)))...))).)).))))))...))))...)))).))..

>KT308992 *Betula* *turkestanica* 5.8S ribosomal RNA gene and internal transcribed spacer 1, partial sequence; 5.8S ribosomal RNA gene, complete sequence; and internal transcribed spacer 2, partial sequence

UCGAAACCUGCCCAGCAGAACGACCCGUGAACCUGUUGAAACAACUGGGGGUGUGGGGCGAUCUCGCCCCUUGCCCCCGAACGGUAGGGAGACACUUGUGCAUCCCUGCCGAACAACGAACCCCGGCGCGGUCCGCGCCAAGGAACUUUAACGAAAGAGUGCCUCCGGCCGCCUCGGAAACGGUGUGCGUGCGGGAGGUGAAUCUUGUCUAGAACCAU

............((((((.(((...)))....)))))).......((((((((.((((((....)))))).))))))))..(((((((((..((....))..))))))))).............((((((...))))))..((...((((....((((..((((((.(((((.(((....)))...))).)).))))))...))))...)))).))..

>KT308991 *Betula* *pendula* subsp. *mandshurica* isolate 1 5.8S ribosomal RNA gene and internal transcribed spacer 1, partial sequence; 5.8S ribosomal RNA gene, complete sequence; and internal transcribed spacer 2, partial sequence

UCGAAACCUGCCCAGCAGAACGACCCGUGAACCUGUUGAAACAACUGGGGGCGKGGGGCGAUCUCGCCCCUUGCCCCCGAACGGUAGGGAGACACUUGUGCAUCCCUGCCGAACAACGAACCCCGGCGCGGUCUGCGCCAAGGAACUUUAACGAAAGAGUGCCUCCGGCCGCCUCGGAAACGGUGUGCGUGCGGGAGGUGAAUCUUGUCUAGAACCAU

............((((((.(((...)))....)))))).......((((((((.((((((....)))))).))))))))..(((((((((..((....))..))))))))).............((((((...))))))..((...((((....((((..((((((.(((((.(((....)))...))).)).))))))...))))...)))).))..

>KT308990 *Betula* *pendula* 5.8S ribosomal RNA gene and internal transcribed spacer 1, partial sequence; 5.8S ribosomal RNA gene, complete sequence; and internal transcribed spacer 2, partial sequence

UCGAAACCUGCCCAGCAGAACGACCCGUGAACCUGUUGAAACAACUGGGGGUGUGGGGCGAUCUCGCCCCUUGCCCCCGAACGGUAGGGAGACACUUGUGCAUCCCUGCCGAACAACGAACCCCGGCGCGGUCCGCGCCAAGGAACUUUAACGAAAGAGUGCCUCCGGCCGCCUCGGAAACGGUGUGCGUGCGGGAGGUGAAUCUUGUCUAGAACCAU

............((((((.(((...)))....)))))).......((((((((.((((((....)))))).))))))))..(((((((((..((....))..))))))))).............((((((...))))))..((...((((....((((..((((((.(((((.(((....)))...))).)).))))))...))))...)))).))..

>KT308989 *Betula* *tianschanica* 5.8S ribosomal RNA gene and internal transcribed spacer 1, partial sequence; 5.8S ribosomal RNA gene, complete sequence; and internal transcribed spacer 2, partial sequence

UCGAAACCUGCCCAGCAGAACGACCCGUGAACCUGUUGAAACAACUGGGGGUGGGGGGC--UCUCGCCCCUUGCCCCCGAACGGUAGGGAGACACUCGUGCAUCCCUGCCGAACAACGAACCCCGGCGCGGUCUGCGCCAAGGAACUUUAACGAAAGAGUGCCUCCUGCCGCCUCGGAAACGGUGUGCGUGCGGGAGGUGAAUCUUGUCUAGAACCAU

............((((((.(((...)))....)))))).......((((((((((((((--....))))))))))))))..(((((((((..((....))..))))))))).............((((((...))))))..((...((((....((((..((((((.(((((.(((....)))...))).)).))))))...))))...)))).))..

>KT308988 *Betula × caerulea* isolate 2 5.8S ribosomal RNA gene and internal transcribed spacer 1, partial sequence; 5.8S ribosomal RNA gene, complete sequence; and internal transcribed spacer 2, partial sequence

UCGAAACCUGCCCAGCAGAACGACCCGUGAACCUGUUGAAACAACUGGGGGUGGGGGGCGAUCUCGCCCCUUGCCCCCGAACGGUAGGGAGACACUUGUGCAUCCCUGCCGAACAACGAACCCCGGCGCGGUCUGCGCCAAGGAACUUUAACGAAAGAGUGCCUCCCGCCGCCUCGGAAACGGUGUGCGUGCGGGAGGUGAAUCUUGUCUAGAACCAU

............((((((.(((...)))....)))))).......(((((((((((((((....)))))))))))))))..(((((((((..((....))..))))))))).............((((((...))))))..((...((((....((((..((((((.(((((.(((....)))...))).)).))))))...))))...)))).))..

>KT308987 *Betula × caerulea* isolate 1 5.8S ribosomal RNA gene and internal transcribed spacer 1, partial sequence; 5.8S ribosomal RNA gene, complete sequence; and internal transcribed spacer 2, partial sequence

UCGAAACCUGCCCAGCAGAACGACCCGUGAACCUGUUGAAACAACUGGGGGYGGGGGGCGAUCUCGCCCCUUGYCCCCGAACGGUAGGGAGACACUUGUGCAUCCCUGCCGAACAACGAACCCCGGCGCGGUCYGCGCCAAGGAACUUUAACGAAAGAGUGCCUCCGGCCGCCUCGGAAACGGUGUGCGUGCGGGAGGUGAAUCUUGUCUAGAACCAU

............((((((.(((...)))....)))))).......(((((((((((((((....)))))))))))))))..(((((((((..((....))..))))))))).............((((((...))))))..((...((((....((((..((((((.(((((.(((....)))...))).)).))))))...))))...)))).))..

>KT308986 *Betula* *middendorffii* 5.8S ribosomal RNA gene and internal transcribed spacer 1, partial sequence; 5.8S ribosomal RNA gene, complete sequence; and internal transcribed spacer 2, partial sequence

UCGAAACCUGCCCAGCAGAACGACCCGUGAACCUGUUGAAACAACUGGGGGUGGGGGGCGAUCUCGCCCCUUGCCCCCGAACGGUAGGGAGACACUUGUGCAUCCCUGCCGAACAACGAACCCCGGCGCGGUCCGCGCCAAGGAACUUUAACGAAAGAGUGCCUCCGGCCGCCUCGGAAACGGUGUGCGUGCGGGAGGUGAAUCUUGUCUAGAACCAU

............((((((.(((...)))....)))))).......(((((((((((((((....)))))))))))))))..(((((((((..((....))..))))))))).............((((((...))))))..((...((((....((((..((((((.(((((.(((....)))...))).)).))))))...))))...)))).))..

>KT308985 *Betula* *minor* 5.8S ribosomal RNA gene and internal transcribed spacer 1, partial sequence; 5.8S ribosomal RNA gene, complete sequence; and internal transcribed spacer 2, partial sequence

UCGAAACCUGCCCAGCAGAACGACCCGUGAACCUGUUGAAACAACUGGGGGYGGGGGGCGAUCUCGCCCCUUGYCCCCGAACGGUAGGGAGACACUUGUGCAUCCCUGCCGAACAACGAACCCCGGCGCGGUCYGCGCCAAGGAACUUUAACGAAAGAGUGCCUCCGGCCGCCUCGGAAACGGUGUGCGUGCGGGAGGUGAAUCUUGUCUAGAACCAU

............((((((.(((...)))....)))))).......(((((((((((((((....)))))))))))))))..(((((((((..((....))..))))))))).............((((((...))))))..((...((((....((((..((((((.(((((.(((....)))...))).)).))))))...))))...)))).))..

>KT308984 *Betula* *microphylla* 5.8S ribosomal RNA gene and internal transcribed spacer 1, partial sequence; 5.8S ribosomal RNA gene, complete sequence; and internal transcribed spacer 2, partial sequence

UCGAAACCUGCCCAGCAGAACGACCCGUGAACCUGUUGAAACAACUGGGGGUGGGGGGCGAUCUCGCCCCUUGCCCCCGAACGGUAGGGAGACACUCGUGCAUCCCUGCCGAACAACGAACCCCGGCGCGGUCUGCGCCAAGGAACUUUAACGAAAGACUGCCUCCCGCCGCCUCGGAAACGGUGUGCGUGCGGGAGGUGAAUCUUGUCUAGAACCAU

............((((((.(((...)))....)))))).......(((((((((((((((....)))))))))))))))..(((((((((..((....))..))))))))).............((((((...))))))..((...((((....((((..((((((.(((((.(((....)))...))).)).))))))...))))...)))).))..

>KT308983 *Betula* *pubescens* var. litiwinowii isolate 2 5.8S ribosomal RNA gene and internal transcribed spacer 1, partial sequence; 5.8S ribosomal RNA gene, complete sequence; and internal transcribed spacer 2, partial sequence

UCGAAACCUGCCCAGCAGAACGACCCGUGAACCUGUUGAAACAACUGGGGGUGGGGGGCGAUCUCGCCCCUUGCCCCCGAACGGUAGGGAGACACUUGUGCAUCCCUGCCGAACAACGAACCCCGGCGCGGUCCGCGCCAAGGAACUUUAACGAAAGAGUGCCUCCGGCCGCCUCGGAAACGGUGUGCGUGCGGGAGGUGAAUCUUGUCUAGAACCAU

............((((((.(((...)))....)))))).......(((((((((((((((....)))))))))))))))..(((((((((..((....))..))))))))).............((((((...))))))..((...((((....((((..((((((.(((((.(((....)))...))).)).))))))...))))...)))).))..

>KT308982 *Betula* *pubescens* var. *pubescens* isolate 4 5.8S ribosomal RNA gene and internal transcribed spacer 1, partial sequence; 5.8S ribosomal RNA gene, complete sequence; and internal transcribed spacer 2, partial sequence

UCGAAACCUGCCCAGCAGAACGACCCGUGAACCUGUUGAAACAACUGGGGGUGGGGGGCGAUCUCGCCCCUUGCCCCCGAACGGUAGGGAGACACUUGUGCAUCCCUGCCGAACAACGAACCCCGGCGCGGUCCGCGCCAAGGAACUUUAACGAAAGAGUGCCUCCGGCCGCCUCGGAAACGGUGUGCGUGCGGGAGGUGAAUCUUGUCUAGAACCAU

............((((((.(((...)))....)))))).......(((((((((((((((....)))))))))))))))..(((((((((..((....))..))))))))).............((((((...))))))..((...((((....((((..((((((.(((((.(((....)))...))).)).))))))...))))...)))).))..

>KT308981 *Betula* *pubescens* var. *pubescens* isolate 3 5.8S ribosomal RNA gene and internal transcribed spacer 1, partial sequence; 5.8S ribosomal RNA gene, complete sequence; and internal transcribed spacer 2, partial sequence

UCGAAACCUGCCCAGCAGAACGACCCGUGAACCUGUUGAAACAACUGGGGGUGGGGGGCGAUCUCGCCCCUUGCCCCCGAACGGUAGGGAGACACUUGUGCAUCCCUGCCGAACAACGAACCCCGGCGCGGUCCGCGCCAAGGAACUUUAACGAAAGAGUGCCUCCGGCCGCCUCGGAAACGGUGUGCGUGCGGGAGGUGAAUCUUGUCUAGAACCAU

............((((((.(((...)))....)))))).......(((((((((((((((....)))))))))))))))..(((((((((..((....))..))))))))).............((((((...))))))..((...((((....((((..((((((.(((((.(((....)))...))).)).))))))...))))...)))).))..

>KT308980 *Betula* *pubescens* var. *pumila* isolate 3 5.8S ribosomal RNA gene and internal transcribed spacer 1, partial sequence; 5.8S ribosomal RNA gene, complete sequence; and internal transcribed spacer 2, partial sequence

UCGAAACCUGCCCAGCAGAACGACCCGUGAACCUGUUGAAACAACUGGGGGUGGGGGGCGAUCUCGCCCCUUGCCCCCGAACGGUAGGGAGACACUUGUGCAUCCCUGCCGAACAACGAACCCCGGCGCGGUCCGCGCCAAGGAACUUUAACGAAAGAGUGCCUCCGGCCGCCUCGGAAACGGUGUGCGUGCGGGAGGUGAAUCUUGUCUAGAACCAU

............((((((.(((...)))....)))))).......(((((((((((((((....)))))))))))))))..(((((((((..((....))..))))))))).............((((((...))))))..((...((((....((((..((((((.(((((.(((....)))...))).)).))))))...))))...)))).))..

>KT308979 *Betula × utahensis* 5.8S ribosomal RNA gene and internal transcribed spacer 1, partial sequence; 5.8S ribosomal RNA gene, complete sequence; and internal transcribed spacer 2, partial sequence

UCGAAACCUGCCCRGCAGAACGACCCGUGAACCUGUUGAAACAACUGGGGGUGGGGGGCGAUCUCGCCCCUUGCCCCCGAACGGUAGGGAGACACUUGUGCAUCCCUGCCGAACAACGAACCCCGGCGCGGUCUGCGCCAAGGAACUUUAACGAAAGAGUGCCUCCSGCCGCCUCGGAAACGGUGUGCGUGCGGGAGGUGAAUCUUGUCUAGAACCAU

............((((((.(((...)))....)))))).......(((((((((((((((....)))))))))))))))..(((((((((..((....))..))))))))).............((((((...))))))..((...((((....((((..((((((.(((((.(((....)))...))).)).))))))...))))...)))).))..

>KT308978 *Betula* *michauxii* 5.8S ribosomal RNA gene and internal transcribed spacer 1, partial sequence; 5.8S ribosomal RNA gene, complete sequence; and internal transcribed spacer 2, partial sequence

UCGAAACCUGCCCAGCAGAACGACCCGUGAACCUGUUGAAACAACUGGGGGCGGGGGGCGAUCUCGCCCCGUGCCCCCGAACGGUAGGGAGACACUCGUGCAUCCCUGCCGAACAACGAACCCCGGCGCGGUCUGCGCCAAGGAACUUUAACGAAAGAGUGCCUCCGGCCGCCUCGGAAACGGUGUGCGUGCGGGAGGUGAAUCUUGUCUAGAACCAU

............((((((.(((...)))....)))))).......((((((((.((((((....)))))).))))))))..(((((((((..((....))..))))))))).............((((((...))))))..((...((((....((((..((((((.(((((.(((....)))...))).)).))))))...))))...)))).))..

>KT308977 *Betula* *pubescens* subsp. *celtiberica* isolate 2 5.8S ribosomal RNA gene and internal transcribed spacer 1, partial sequence; 5.8S ribosomal RNA gene, complete sequence; and internal transcribed spacer 2, partial sequence

UCGAAACCUGCCCAGCAGAACGACCCGUGAACCUGUUGAAACAACUGGGGGUGGGGGGCGAUCUCGCCCCUUGCCCCCGAACGGUAGGGAGACACUUGUGCAUCCCUGCCGAACAACGAACCCCGGCGCGGUCCGCGCCAAGGAACUUUAACGAAAGAGUGCCUCCGGCCGCCUCGGAAACGGUGUGCGUGCGGGAGGUGAAUCUUGUCUAGAACCAU

............((((((.(((...)))....)))))).......(((((((((((((((....)))))))))))))))..(((((((((..((....))..))))))))).............((((((...))))))..((...((((....((((..((((((.(((((.(((....)))...))).)).))))))...))))...)))).))..

>KT308976 *Betula* *pubescens* var. *pumila* isolate 2 5.8S ribosomal RNA gene and internal transcribed spacer 1, partial sequence; 5.8S ribosomal RNA gene, complete sequence; and internal transcribed spacer 2, partial sequence

UCGAAACCUGCCCAGCAGAACGACCCGUGAACCUGUUGAAACAACUGGGGGUGGGGGGCGAUCUCGCCCCUUGCCCCCGAACGGUAGGGAGACACUUGUGCAUCCCUGCCGAACAACGAACCCCGGCGCGGUCCGCGCCAAGGAACUUUAACGAAAGAGUGCCUCCGGCCGCCUCGGAAACGGUGUGCGUGCGGGAGGUGAAUCUUGUCUAGAACCAU

............((((((.(((...)))....)))))).......(((((((((((((((....)))))))))))))))..(((((((((..((....))..))))))))).............((((((...))))))..((...((((....((((..((((((.(((((.(((....)))...))).)).))))))...))))...)))).))..

>KT308975 *Betula* *pubescens* var. fragans isolate 2 5.8S ribosomal RNA gene and internal transcribed spacer 1, partial sequence; 5.8S ribosomal RNA gene, complete sequence; and internal transcribed spacer 2, partial sequence

UCGAAACCUGCCCAGCAGAACGACCCGUGAACCUGUUGAAACAACUGGGGGUGGGGGGCGAUCUCGCCCCUUGCCCCCGAACGGUAGGGAGACACUUGUGCAUCCCUGCCGAACAACGAACCCCGGCGCGGUCCGCGCCAAGGAACUUUAACGAAAGAGUGCCUCCGGCCGCCUCGGAAACGGUGUGCGUGCGGGAGGUGAAUCUUGUCUAGAACCAU

............((((((.(((...)))....)))))).......(((((((((((((((....)))))))))))))))..(((((((((..((....))..))))))))).............((((((...))))))..((...((((....((((..((((((.(((((.(((....)))...))).)).))))))...))))...)))).))..

>KT308974 *Betula* *pubescens* var. fragans isolate 1 5.8S ribosomal RNA gene and internal transcribed spacer 1, partial sequence; 5.8S ribosomal RNA gene, complete sequence; and internal transcribed spacer 2, partial sequence

UCGAAACCUGCCCAGCAGAACGACCCGUGAACCUGUUGAAACAACUGGGGGUGGGGGGCGAUCUCGCCCCUUGCCCCCGAACGGUAGGGAGACACUUGUGCAUCCCUGCCGAACAACGAACCCCGGCGCGGUCCGCGCCAAGGAACUUUAACGAAAGAGUGCCUCCGGCCGCCUCGGAAACGGUGUGCGUGCGGGAGGUGAAUCUUGUCUAGAACCAU

............((((((.(((...)))....)))))).......(((((((((((((((....)))))))))))))))..(((((((((..((....))..))))))))).............((((((...))))))..((...((((....((((..((((((.(((((.(((....)))...))).)).))))))...))))...)))).))..

>KT308973 *Betula* *pubescens* var. *pumila* isolate 1 5.8S ribosomal RNA gene and internal transcribed spacer 1, partial sequence; 5.8S ribosomal RNA gene, complete sequence; and internal transcribed spacer 2, partial sequence

UCGAAACCUGCCCAGCAGAACGACCCGUGAACCUGUUGAAACAACUGGGGGUGGGGGGCGAUCUCGCCCCUUGCCCCCGAACGGUAGGGAGACACUUGUGCAUCCCUGCCGAACAACGAACCCCGGCGCGGUCCGCGCCAAGGAACUUUAACGAAAGAGUGCCUCCGGCCGCCUCGGAAACGGUGUGCGUGCGGGAGGUGAAUCUUGUCUAGAACCAU

............((((((.(((...)))....)))))).......(((((((((((((((....)))))))))))))))..(((((((((..((....))..))))))))).............((((((...))))))..((...((((....((((..((((((.(((((.(((....)))...))).)).))))))...))))...)))).))..

>KT308972 *Betula* *pubescens* subsp. *celtiberica* isolate 1 5.8S ribosomal RNA gene and internal transcribed spacer 1, partial sequence; 5.8S ribosomal RNA gene, complete sequence; and internal transcribed spacer 2, partial sequence

UCGAAACCUGCCCAGCAGAACGACCCGUGAACCUGUUGAAACAACUGGGGGUGGGGGGCGAUCUCGCCCCUUGCCCCCGAACGGUAGGGAGACACUUGUGCAUCCCUGCCGAACAACGAACCCCGGCGCGGUCCGCGCCAAGGAACUUUAACGAAAGAGUGCCUCCGGCCGCCUCGGAAACGGUGUGCGUGCGGGAGGUGAAUCUUGUCUAGAACCAU

............((((((.(((...)))....)))))).......(((((((((((((((....)))))))))))))))..(((((((((..((....))..))))))))).............((((((...))))))..((...((((....((((..((((((.(((((.(((....)))...))).)).))))))...))))...)))).))..

>KT308971 *Betula* *pubescens* var. litiwinowii isolate 1 5.8S ribosomal RNA gene and internal transcribed spacer 1, partial sequence; 5.8S ribosomal RNA gene, complete sequence; and internal transcribed spacer 2, partial sequence

UCGAAACCUGCCCAGCAGAACGACCCGUGAACCUGUUGAAACAACUGGGGGUGGGGGGCGAUCUCGCCCCUUGCCCCCGAACGGUAGGGAGACACUUGUGCAUCCCUGCCGAACAACGAACCCCGGCGCGGUCCGCGCCAAGGAACUUUAACGAAAGAGUGCCUCCGGCCGCCUCGGAAACGGUGUGCGUGCGGGAGGUGAAUCUUGUCUAGAACCAU

............((((((.(((...)))....)))))).......(((((((((((((((....)))))))))))))))..(((((((((..((....))..))))))))).............((((((...))))))..((...((((....((((..((((((.(((((.(((....)))...))).)).))))))...))))...)))).))..

>KT308970 *Betula* *pubescens* var. *pubescens* isolate 2 5.8S ribosomal RNA gene and internal transcribed spacer 1, partial sequence; 5.8S ribosomal RNA gene, complete sequence; and internal transcribed spacer 2, partial sequence

UCGAAACCUGCCCAGCAGAACGACCCGUGAACCUGUUGAAACAACUGGGGGUGGGGGGCGAUCUCGCCCCUUGCCCCCGAACGGUAGGGAGACACUUGUGCAUCCCUGCCGAACAACGAACCCCGGCGCGGUCCGCGCCAAGGAACUUUAACGAAAGAGUGCCUCCGGCCGCCUCGGAAACGGUGUGCGUGCGGGAGGUGAAUCUUGUCUAGAACCAU

............((((((.(((...)))....)))))).......(((((((((((((((....)))))))))))))))..(((((((((..((....))..))))))))).............((((((...))))))..((...((((....((((..((((((.(((((.(((....)))...))).)).))))))...))))...)))).))..

>KT308969 *Betula* *pubescens* var. *pubescens* isolate 1 5.8S ribosomal RNA gene and internal transcribed spacer 1, partial sequence; 5.8S ribosomal RNA gene, complete sequence; and internal transcribed spacer 2, partial sequence

UCGAAACCUGCCCAGCAGAACGACCCGUGAACCUGUUGAAACAACUGGGGGUGGGGGGCGAUCUCGCCCCUUGCCCCCGAACGGUAGGGAGACACUUGUGCAUCCCUGCCGAACAACGAACCCCGGCGCGGUCCGCGCCAAGGAACUUUAACGAAAGAGUGCCUCCGGCCGCCUCGGAAACGGUGUGCGUGCGGGAGGUGAAUCUUGUCUAGAACCAU

............((((((.(((...)))....)))))).......(((((((((((((((....)))))))))))))))..(((((((((..((....))..))))))))).............((((((...))))))..((...((((....((((..((((((.(((((.(((....)))...))).)).))))))...))))...)))).))..

>KT308968 *Betula* *browicziana* 5.8S ribosomal RNA gene and internal transcribed spacer 1, partial sequence; 5.8S ribosomal RNA gene, complete sequence; and internal transcribed spacer 2, partial sequence

UCGAAACCUGCCCAGCAGAACGACCCGUGAACCUGUUGAAACAACUGGGGGUGUGGGGCGAUCUCGCCCCUUGCCCCCGAACGGUAGGGAGACACUUGUGCAUCCCUGCCGAACAACGAACCCCGGCGCGGUCCGCGCCAAGGAACUUUAACGAAAGAGUGCCUCCGGCCGCCUCGGAAACGGUGUGCGUGCGGGAGGUGAAUCUUGUCUAGAACCAU

............((((((.(((...)))....)))))).......((((((((.((((((....)))))).))))))))..(((((((((..((....))..))))))))).............((((((...))))))..((...((((....((((..((((((.(((((.(((....)))...))).)).))))))...))))...)))).))..

>KT308967 *Betula* *halophila* 5.8S ribosomal RNA gene and internal transcribed spacer 1, partial sequence; 5.8S ribosomal RNA gene, complete sequence; and internal transcribed spacer 2, partial sequence

UCGAAACCUGCCCAGCAGAACGACCCGUGAACCUGUUGAAACAACUGGGGGUGGGGGGCGAUCUCGCCCCUUGCCCCCGAACGGUAGGGAGACACUYGUGCAUCCCUGCCGAACAACGAACCCCGGCGCGGUCYGCGCCAAGGAACUUUAACGAAAGAGUGCCUCCGGCCGCCUCGGAAACGGUGUGCGUGCGGGAGGUGAAUCUUGUCUAGAACCAU

............((((((.(((...)))....)))))).......(((((((((((((((....)))))))))))))))..(((((((((..((....))..))))))))).............((((((...))))))..((...((((....((((..((((((.(((((.(((....)))...))).)).))))))...))))...)))).))..

>KT308966 *Betula* *raddeana* 5.8S ribosomal RNA gene and internal transcribed spacer 1, partial sequence; 5.8S ribosomal RNA gene, complete sequence; and internal transcribed spacer 2, partial sequence

UCGAAACCUGCCCAGCAGAACGACCCGUGAACCUGUUGAAACAACUGGGGGUGGGGGGC--UCUCGCCCCUUGCCCCCGAACGGUAGGGAGACACUCGUGCAUCCCUGCCGAACAACGAACCCCGGCGCGGUCUGCGCCAAGGAACUUUAACGAAAGAGUGCCUCCCGCCGCCUCGGAAACGGUGUGCGUGCGGGAGGUGAAUCUUGUCUAGAACCAU

............((((((.(((...)))....)))))).......((((((((((((((--....))))))))))))))..(((((((((..((....))..))))))))).............((((((...))))))..((...((((....((((..((((((.(((((.(((....)))...))).)).))))))...))))...)))).))..

>KT308965 *Betula* *nigra* isolate 2 5.8S ribosomal RNA gene and internal transcribed spacer 1, partial sequence; 5.8S ribosomal RNA gene, complete sequence; and internal transcribed spacer 2, partial sequence

UCGAAACCUGCCCAGCAGAACGACCCGUGAACCUGUUGAAACAACUGGGGGUGGGGGGCGAUCUCGCCCCGUGCCUCCGAACGGUAGGGAGACACUUGUGCAUCCCUGCUGAACAACGAACCCCGGCGCGGUCUGCGCCAAGGAACUUUAACGAAAGAGUGCCUCCGGCCGCCUCGGAAACGGUGUGCGUGCAGGAAAACAAUCUUGUCUAGAACCAC

............((((((.(((...)))....)))))).......((((((((.((((((....)))))).))))))))..(((((((((..((....))..))))))))).............((((((...))))))..((...((((....((((.((..(((.(((((.(((....)))...))).)).)))...)).))))...)))).))..

>KT308964 *Betula* *nigra* isolate 1 5.8S ribosomal RNA gene and internal transcribed spacer 1, partial sequence; 5.8S ribosomal RNA gene, complete sequence; and internal transcribed spacer 2, partial sequence

UCGAAACCUGCCCAGCAGAACGACCCGUGAACCUGUUGAAACAACUGGGGGUGGGGGGCGAUCUCGCCCCGUGCCUCCGAACGGUAGGGAGACACUUGUGCAUCCCUGCUGAACAACGAACCCCGGCGCGGUCUGCGCCAAGGAACUUUAACGAAAGAGUGCCUCCGGCCGCCUCGGAAACGGUGUGCGUGCAGGAAAACAAUCUUGUCUAGAACCAC

............((((((.(((...)))....)))))).......((((((((.((((((....)))))).))))))))..(((((((((..((....))..))))))))).............((((((...))))))..((...((((....((((.((..(((.(((((.(((....)))...))).)).)))...)).))))...)))).))..

>KT308963 *Betula* *dahurica* isolate 2 5.8S ribosomal RNA gene and internal transcribed spacer 1, partial sequence; 5.8S ribosomal RNA gene, complete sequence; and internal transcribed spacer 2, partial sequence

UCGAAACCUGCCCAGCAGAACGACCCGUGAACCUGUUGAAACAACUGGGGGUGGGGGGCGAUCUCGCCCCUUGCCCCCGAACGGUAGGGAGACACUUGUGCAUCCCUGCCGAACAACGAACCCCGGCGCGGUCUGCGCCAAGGAACUUUAACGAAAGAGUGCCUCCCGCCGCCUCGGAAACGGUGUGCGUGCGGGAGGUGAAUCUUGUCUAGAACCAU

............((((((.(((...)))....)))))).......(((((((((((((((....)))))))))))))))..(((((((((..((....))..))))))))).............((((((...))))))..((...((((....((((..((((((.(((((.(((....)))...))).)).))))))...))))...)))).))..

>KT308962 *Betula* *dahurica* isolate 1 5.8S ribosomal RNA gene and internal transcribed spacer 1, partial sequence; 5.8S ribosomal RNA gene, complete sequence; and internal transcribed spacer 2, partial sequence

UCGAAACCUGCCCRGCAGAACGACCCGUGAACCUGUUGAAACAACUGGGGGUGGGGGGCGAUCUCGCCCCUUGCCCCCGAACGGUAGGGAGACACUUGUGCAUCCCUGCCGAACAACGAACCCCGGCGCGGUCUGCGCCAAGGAACUUUAACGAAAGAGUGCCUCCCGCCGCCUCGGAAACGGUGUGCGUGCGGGAGGUGAAUCUUGUCUAGAACCAU

............((((((.(((...)))....)))))).......(((((((((((((((....)))))))))))))))..(((((((((..((....))..))))))))).............((((((...))))))..((...((((....((((..((((((.(((((.(((....)))...))).)).))))))...))))...)))).))..

>KT308961 *Betula* *ashburneri* isolate 3 5.8S ribosomal RNA gene and internal transcribed spacer 1, partial sequence; 5.8S ribosomal RNA gene, complete sequence; and internal transcribed spacer 2, partial sequence

UCGAAACCUGCCCAGCAGAACGACCCGUGAACCUGUUGAAACAACUGGGGGUGGGGGGCGAUCUCGCCCCUUGCCCUCGAACGGUAGGGAGACACUCGUGCAUCCCUGCCGAACAACGAACCCCGGCGCGGUCUGCGCCAAGGAACUUUAACGAAAGAGUGCCUCCGGCCGCCUCGGAAACGGUGUGCGUGCGGGAGGUGAAUCUUGUCUAGAACCAU

............((((((.(((...)))....)))))).......(((((((((((((((....)))))))))))))))..(((((((((..((....))..))))))))).............((((((...))))))..((...((((....((((..((((((.(((((.(((....)))...))).)).))))))...))))...)))).))..

>KT308960 *Betula* *lanata* isolate 2 5.8S ribosomal RNA gene and internal transcribed spacer 1, partial sequence; 5.8S ribosomal RNA gene, complete sequence; and internal transcribed spacer 2, partial sequence

UCGAAACCUGCCCAGCAGAACGACCCGUGAACCUGUUGAAACAACUGGGGGUGGGGGGCGAUCUCGCCCCUUGCCCUCGAACGGUAGGGAGACACUCGUGCAUCCCUGCCGAACAACGAACCCCGGCGCGGUCUGCGCCAAGGAACUUUAACGAAAGAGUGCCUCCGGCCGCCUCGGAAACGGUGUGCGUGCGGGAGGUGAAUCUUGUCUAGAACCAU

............((((((.(((...)))....)))))).......(((((((((((((((....)))))))))))))))..(((((((((..((....))..))))))))).............((((((...))))))..((...((((....((((..((((((.(((((.(((....)))...))).)).))))))...))))...)))).))..

>KT308959 *Betula* *lanata* isolate 1 5.8S ribosomal RNA gene and internal transcribed spacer 1, partial sequence; 5.8S ribosomal RNA gene, complete sequence; and internal transcribed spacer 2, partial sequence

UCGAAACCUGCCCAGCAGAACGACCCGUGAACCUGUUGAAACAACUGGGGGUGGGGGGCGAUCUCGCCCCUUGCCCUCGAACGGUAGGGAGACACUCGUGCAUCCCUGCCGAACAACGAACCCCGGCGCGGUCUGCGCCAAGGAACUUUAACGAAAGAGUGCCUCCGGCCGCCUCGGAAACGGUGUGCGUGCGGGAGGUGAAUCUUGUCUAGAACCAU

............((((((.(((...)))....)))))).......(((((((((((((((....)))))))))))))))..(((((((((..((....))..))))))))).............((((((...))))))..((...((((....((((..((((((.(((((.(((....)))...))).)).))))))...))))...)))).))..

>KT308958 *Betula* *costata* 5.8S ribosomal RNA gene and internal transcribed spacer 1, partial sequence; 5.8S ribosomal RNA gene, complete sequence; and internal transcribed spacer 2, partial sequence

UCGAAACCUGCCCAGCAGAACGACCCGUGAACCUGUUGAAACAACUGGGGGYGGGGGGCGAUCUCGCCCCKUGCCCYCGAACGGUAGGGAGACACUCGUGCAUCCCUGCYGAACAACGAACCCCGGCGCGGUCUGCGCCAAGGAACUUUAACGAAAGAGUGCCUCCGGCCGCCUCGGAAACGGUGUGCGUGCGGGAGGUGAAUCUUGUCUAGAACCAU

............((((((.(((...)))....)))))).......((((((((.((((((....)))))).))))))))..(((((((((..((....))..))))))))).............((((((...))))))..((...((((....((((..((((((.(((((.(((....)))...))).)).))))))...))))...)))).))..

>KT308957 *Betula* *ermanii* isolate 2 5.8S ribosomal RNA gene and internal transcribed spacer 1, partial sequence; 5.8S ribosomal RNA gene, complete sequence; and internal transcribed spacer 2, partial sequence

UCGAAACCUGCCCAGCAGAACGACCCGUGAACCUGUUGAAACAACUGGGGGYGGGGGGCGAUCUCGCCCCKUGCCCUCGAACGGUAGGGAGACACUCGUGCAUCCCUGCCGAACAACGAACCCCGGCGCGGUCUGCGCCAAGGAACUUUAACGAAAGAGUGCCUCCGGCCGCCUCGGAAACGGUGUGCGUGCGGGAGGUGAAUCUUGUCUAGAACCAU

............((((((.(((...)))....)))))).......((((((((.((((((....)))))).))))))))..(((((((((..((....))..))))))))).............((((((...))))))..((...((((....((((..((((((.(((((.(((....)))...))).)).))))))...))))...)))).))..

>KT308956 *Betula* *ermanii* isolate 1 5.8S ribosomal RNA gene and internal transcribed spacer 1, partial sequence; 5.8S ribosomal RNA gene, complete sequence; and internal transcribed spacer 2, partial sequence

UCGAAACCUGCCCAGCAGAACGACCCGUGAACCUGUUGAAACAACUGGGGGCGGGGGGCGAUCUCGCCCCKUGCCCUCGAACGGUAGGGAGACACUCGUGCAUCCCUGCYGAACAACGAACCCCGGCGCGGUCUGCGCCAAGGAACUUUAACGAAAGAGUGCCUCCGGCCGCCUCGGAAACGGUGUGCGUGCGGGAGGUGAAUCUUGUCUAGAACCAU

............((((((.(((...)))....)))))).......((((((((.((((((....)))))).))))))))..(((((((((..((....))..))))))))).............((((((...))))))..((...((((....((((..((((((.(((((.(((....)))...))).)).))))))...))))...)))).))..

>KT308955 *Betula* *utilis* var. *prattii* 5.8S ribosomal RNA gene and internal transcribed spacer 1, partial sequence; 5.8S ribosomal RNA gene, complete sequence; and internal transcribed spacer 2, partial sequence

UCGAAACCUGCCCAGCAGAACGACCCGUGAACCUGUUGAAACAACUGGGGGUGGGGGGCGAUCUCGCCCCUUGCCCUCGAACGGUAGGGAGACACUCGUGCAUCCCUGCCGAACAACGAACCCCGGCGCGGUCUGCGCCAAGGAACUUUAACGAAAGAGUGCCUCCGGCCGCCUCGGAAACGGUGUGCGUGCGGGAGGUGAAUCUUGUCUAGAACCAU

............((((((.(((...)))....)))))).......(((((((((((((((....)))))))))))))))..(((((((((..((....))..))))))))).............((((((...))))))..((...((((....((((..((((((.(((((.(((....)))...))).)).))))))...))))...)))).))..

>KT308954 *Betula* *albosinensis* isolate 2 5.8S ribosomal RNA gene and internal transcribed spacer 1, partial sequence; 5.8S ribosomal RNA gene, complete sequence; and internal transcribed spacer 2, partial sequence

UCGAAACCUGCCCAGCAGAACGACCCGUGAACCUGUUGAAACAACUGGGGGUGGGGGGCGAUCUCGCCCCUUGCCCUCGAACGGUAGGGAGACACUCGUGCAUCCCUGCCGAACAACGAACCCCGGCGCGGUCUGCGCCAAGGAACUUUAACGAAAGAGUGCCUCCGGCCGCCUCGGAAACGGUGUGCGUGCGGGAGGUGAAUCUUGUCUAGAACCAU

............((((((.(((...)))....)))))).......(((((((((((((((....)))))))))))))))..(((((((((..((....))..))))))))).............((((((...))))))..((...((((....((((..((((((.(((((.(((....)))...))).)).))))))...))))...)))).))..

>KT308953 *Betula* *ashburneri* isolate 2 5.8S ribosomal RNA gene and internal transcribed spacer 1, partial sequence; 5.8S ribosomal RNA gene, complete sequence; and internal transcribed spacer 2, partial sequence

UCGAAACCUGCCCAGCAGAACGACCCGUGAACCUGUUGAAACAACUGGGGGUGGGGGGCGAUCUCGCCCCUUGCCCUCGAACGGUAGGGAGACACUCGUGCAUCCCUGCCGAACAACGAACCCCGGCGCGGUCUGCGCCAAGGAACUUUAACGAAAGAGUGCCUCCGGCCGCCUCGGAAACGGUGUGCGUGCGGGAGGUGAAUCUUGUCUAGAACCAU

............((((((.(((...)))....)))))).......(((((((((((((((....)))))))))))))))..(((((((((..((....))..))))))))).............((((((...))))))..((...((((....((((..((((((.(((((.(((....)))...))).)).))))))...))))...)))).))..

>KT308952 *Betula* *ashburneri* isolate 1 5.8S ribosomal RNA gene and internal transcribed spacer 1, partial sequence; 5.8S ribosomal RNA gene, complete sequence; and internal transcribed spacer 2, partial sequence

UCGAAACCUGCCCAGCAGAACGACCCGUGAACCUGUUGAAACAACUGGGGGUGGGGGGCGAUCUCGCCCCUUGCCCUCGAACGGUAGGGAGACACUCGUGCAUCCCUGCCGAACAACGAACCCCGGCGCGGUCUGCGCCAAGGAACUUUAACGAAAGAGUGCCUCCGGCCGCCUCGGAAACGGUGUGCGUGCGGGAGGUGAAUCUUGUCUAGAACCAU

............((((((.(((...)))....)))))).......(((((((((((((((....)))))))))))))))..(((((((((..((....))..))))))))).............((((((...))))))..((...((((....((((..((((((.(((((.(((....)))...))).)).))))))...))))...)))).))..

>KT308951 *Betula* *utilis* var. jacquemontii 5.8S ribosomal RNA gene and internal transcribed spacer 1, partial sequence; 5.8S ribosomal RNA gene, complete sequence; and internal transcribed spacer 2, partial sequence

UCGAAACCUGCCCAGCAGAACGACCCGUGAACCUGUUGAAACAACUGGGGGUGGGGGGCGAUCUCGCCCCUUGCCCUCGAACGGUAGGGAGACACUCGUGCAUCCCUGCCGAACAACGAACCCCGGCGCGGUCUGCGCCAAGGAACUUUAACGAAAGAGUGCCUCCGGCCGCCUCGGAAACGGUGUGCGUGCGGGAGGUGAAUCUUGUCUAGAACCAU

............((((((.(((...)))....)))))).......(((((((((((((((....)))))))))))))))..(((((((((..((....))..))))))))).............((((((...))))))..((...((((....((((..((((((.(((((.(((....)))...))).)).))))))...))))...)))).))..

>KT308950 *Betula* *utilis* var. *occidentalis* isolate 2 5.8S ribosomal RNA gene and internal transcribed spacer 1, partial sequence; 5.8S ribosomal RNA gene, complete sequence; and internal transcribed spacer 2, partial sequence

UCGAAACCUGCCCAGCAGAACGACCCGUGAACCUGUUGAAACAACUGGGGGUGGGGGGCGAUCUCGCCCCUUGCCCYCGAACGGUAGGGAGACACUCGUGCAUCCCUGCCGAACAACGAACCCCGGCGCGGUCUGCGCCAAGGAACUUUAACGAAAGAGUGCCUCCGGCCGCCUCGGAAACGGUGUGCGUGCGGGAGGUGAAUCUUGUCUAGAACCAU

............((((((.(((...)))....)))))).......(((((((((((((((....)))))))))))))))..(((((((((..((....))..))))))))).............((((((...))))))..((...((((....((((..((((((.(((((.(((....)))...))).)).))))))...))))...)))).))..

>KT308949 *Betula* *utilis* isolate 2 5.8S ribosomal RNA gene and internal transcribed spacer 1, partial sequence; 5.8S ribosomal RNA gene, complete sequence; and internal transcribed spacer 2, partial sequence

UCGAAACCUGCCCAGCAGAACGACCCGUGAACCUGUUGAAACAACUGGGGGUGGGGGGCGAUCUCGCCCCUUGCCCUCGAACGGUAGGGAGACACUCGUGCAUCCCUGCCGAACAACGAACCCCGGCGCGGUCUGCGCCAAGGAACUUUAACGAAAGAGUGCCUCCGGCCGCCUCGGAAACGGUGUGCGUGCGGGAGGUGAAUCUUGUCUAGAACCAU

............((((((.(((...)))....)))))).......(((((((((((((((....)))))))))))))))..(((((((((..((....))..))))))))).............((((((...))))))..((...((((....((((..((((((.(((((.(((....)))...))).)).))))))...))))...)))).))..

>KT308948 *Betula* *utilis* isolate 1 5.8S ribosomal RNA gene and internal transcribed spacer 1, partial sequence; 5.8S ribosomal RNA gene, complete sequence; and internal transcribed spacer 2, partial sequence

UCGAAACCUGCCCAGCAGAACGACCCGUGAACCUGUUGAAACAACUGGGGGUGGGGGGCGAUCUCGCCCCUUGCCCUCGAACGGUAGGGAGACACUCGUGCAUCCCUGCCGAACAACGAACCCCGGCGCGGUCUGCGCCAAGGAACUUUAACGAAAGAGUGCCUCCGGCCGCCUCGGAAACGGUGUGCGUGCGGGAGGUGAAUCUUGUCUAGAACCAU

............((((((.(((...)))....)))))).......(((((((((((((((....)))))))))))))))..(((((((((..((....))..))))))))).............((((((...))))))..((...((((....((((..((((((.(((((.(((....)))...))).)).))))))...))))...)))).))..

>KT308947 *Betula* *albosinensis* var. septentrionalis 5.8S ribosomal RNA gene and internal transcribed spacer 1, partial sequence; 5.8S ribosomal RNA gene, complete sequence; and internal transcribed spacer 2, partial sequence

UCGAAACCUGCCCAGCAGAACGACCCGUGAACCUGUUGAAACAACUGGGGGUGGGGGGCGAUCUCGCCCCUUGCCCYCGAACGGUAGGGAGACACUCGUGCAUCCCUGCCGAACAACGAACCCCGGCGCGGUCUGCGCCAAGGAACUUUAACGAAAGAGUGCCUCCGGCCGCCUCGGAAACGGYGUGCGUGCGGGAGGUGAAUCUUGUCUAGAACCAU

............((((((.(((...)))....)))))).......(((((((((((((((....)))))))))))))))..(((((((((..((....))..))))))))).............((((((...))))))..((...((((....((((..((((((.(((((.(((....)))...))).)).))))))...))))...)))).))..

>KT308946 *Betula* *maximovicziana* isolate 2 5.8S ribosomal RNA gene and internal transcribed spacer 1, partial sequence; 5.8S ribosomal RNA gene, complete sequence; and internal transcribed spacer 2, partial sequence

UCGAAACCUGCCCAGCAGAACGACCCGUGAACCUGUUGAAACAACUGGGGGUGGGGGGCGAUCUCGCCCCUUGCCCCCGAACGGUAGGGAGACACUCGUGCAUCCCUGCCGAACAACGAACCCCGGCGCGGUCUGCGCCAAGGAACUUUAACGAAAGAGUGCCUCCGGCCGCCUCGGAAACGGUGUGCGUGCGGGAGGUGAAUCUUGUCUAGAACCAU

............((((((.(((...)))....)))))).......(((((((((((((((....)))))))))))))))..(((((((((..((....))..))))))))).............((((((...))))))..((...((((....((((..((((((.(((((.(((....)))...))).)).))))))...))))...)))).))..

>KT308945 *Betula* *maximovicziana* isolate 1 5.8S ribosomal RNA gene and internal transcribed spacer 1, partial sequence; 5.8S ribosomal RNA gene, complete sequence; and internal transcribed spacer 2, partial sequence

UCGAAACCUGCCCAGCAGAACGACCCGUGAACCUGUUGAAACAACUGGGGGUGGGGGGCGAUCUCGCCCCUUGCCCCCGAACGGUAGGGAGACACUCGUGCAUCCCUGCCGAACAACGAACCCCGGCGCGGUCUGCGCCAAGGAACUUUAACGAAAGAGUGCCUCCGGCCGCCUCGGAAACGGUGUGCGUGCGGGAGGUGAAUCUUGUCUAGAACCAU

............((((((.(((...)))....)))))).......(((((((((((((((....)))))))))))))))..(((((((((..((....))..))))))))).............((((((...))))))..((...((((....((((..((((((.(((((.(((....)))...))).)).))))))...))))...)))).))..

>KT308944 *Betula* *luminifera* isolate 3 5.8S ribosomal RNA gene and internal transcribed spacer 1, partial sequence; 5.8S ribosomal RNA gene, complete sequence; and internal transcribed spacer 2, partial sequence

UCGAAACCUGCCCAGCAGAACGACCCGCGAACCUGUUGAAACAACUGGGGGCGGGGGGCGAUCUCGCCCCGUGCCCCCGAACGGUAGGGAGACACUCGUGCAUCCCUGCCGAACAACGAACCCCGGCGCGGUCUGCGCCAAGGAACUUUAACGAAAGAGUGCCUCCGGCCGCCUCGGAAACGGUGUGCGUGCGGGAGGUGAAUCUUGUCUAGAACCAU

............((((((..((...)).....)))))).......((((((((.((((((....)))))).))))))))..(((((((((..((....))..))))))))).............((((((...))))))..((...((((....((((..((((((.(((((.(((....)))...))).)).))))))...))))...)))).))..

>KT308943 *Betula* *luminifera* isolate 2 5.8S ribosomal RNA gene and internal transcribed spacer 1, partial sequence; 5.8S ribosomal RNA gene, complete sequence; and internal transcribed spacer 2, partial sequence

UCGAAACCUGCCCAGCAGAACGACCCGUGAACCUGUUGAAACAACUGGGGGCRGGGGGCGAUCUCGCCCCGUGCCYCCGAACGGUAGGGAGACACUCGUGCAUCCCUGCCGAACAACGAACCCCGGCGCGGUCUGCGCCAAGGAACUUUAACGAAAGAGUGCCUCCGGCCGYCUCGGAAACGGUGUGCGUGCGGGAGGUGAAUCUUGUCUAGAACCAU

............((((((.(((...)))....)))))).......((((((((.((((((....)))))).))))))))..(((((((((..((....))..))))))))).............((((((...))))))..((...((((....((((..((((((.(((((.(((....)))...))).)).))))))...))))...)))).))..

>KT308942 *Betula* *hainanensis* 5.8S ribosomal RNA gene and internal transcribed spacer 1, partial sequence; 5.8S ribosomal RNA gene, complete sequence; and internal transcribed spacer 2, partial sequence

UCGAAACCUGCCCAGCAGAACGACCCGUGAACCUGUUGAAACAACUGGGGGCRGGGGGCGAUCUCGCCCCGUGCCYCCGAACGGUAGGGAGACACUCGUGCAUCCCUGCCGAACAACGAACCCCGGCGCGGUCUGCGCCAAGGAACUUUAACGAAAGAGUGCCUCCGGCCGYCUCGGAAACGGUGUGCGUGCGGGAGGUGAAUCUUGUCUAGAACCAU

............((((((.(((...)))....)))))).......((((((((.((((((....)))))).))))))))..(((((((((..((....))..))))))))).............((((((...))))))..((...((((....((((..((((((.(((((.(((....)))...))).)).))))))...))))...)))).))..

>KT308941 *Betula* *cylindrostachya* 5.8S ribosomal RNA gene and internal transcribed spacer 1, partial sequence; 5.8S ribosomal RNA gene, complete sequence; and internal transcribed spacer 2, partial sequence

UCGAAACCUGCCCAGCAGAACGACCCGUGAACCUGUUGAAACAACUGGGGGCRGGGGGCGAUCUCGCCCCGUGCCYCCGAACGGUAGGGAGACACUCGUGCAUCCCUGCCGAACAACGAACCCCGGCGCGGUCUGCGCCAAGGAACUUUAACGAAAGAGUGCCUCCGGCCGYCUCGGAAACGGUGUGCGUGCGGGAGGUGAAUCUUGUCUAGAACCAU

............((((((.(((...)))....)))))).......((((((((.((((((....)))))).))))))))..(((((((((..((....))..))))))))).............((((((...))))))..((...((((....((((..((((((.(((((.(((....)))...))).)).))))))...))))...)))).))..

>KT308940 *Betula* *alnoides* 5.8S ribosomal RNA gene and internal transcribed spacer 1, partial sequence; 5.8S ribosomal RNA gene, complete sequence; and internal transcribed spacer 2, partial sequence

UCGAAACCUGCCCAGCAGAACGACCCGUGAACCUGUUGAAACAACUGGGGGCGGGGGGCGAUCUCGCCCCGUGCCCCCGAACGGUAGGGAGACACUCGUGCAUCCCUGCCGAACAACGAACCCCGGCGCGGUCUGCGCCAAGGAACUUUAACGAAAGAGUGCCUCCGGCCGCCUCGGAAACGGUGUGCGUGCGGGAGGUGAAUCUUGUCUAGAACCAU

............((((((.(((...)))....)))))).......((((((((.((((((....)))))).))))))))..(((((((((..((....))..))))))))).............((((((...))))))..((...((((....((((..((((((.(((((.(((....)))...))).)).))))))...))))...)))).))..

>KT308938 *Betula* *lenta* f. *uber* isolate 2 5.8S ribosomal RNA gene and internal transcribed spacer 1, partial sequence; 5.8S ribosomal RNA gene, complete sequence; and internal transcribed spacer 2, partial sequence

UCGAAACCUGCCCAGCAGAACGACCCGUGAACAUGUUGAAACAACUGGGGGCGGGGGGCGAUCUCGCCCCGUGCCCCCGAACGGCAGGGAGACACUCGUGCAUCCCUGCCGAACAACGAACCCCGGCGCGGUCUGCGCCAAGGAACUUUAACGAAAGAGUGCCUCCGGCCGUCUCGGAAACGGUGUGCGUGCGGGAGGCGAAUCUUGUCUAGAACCAU

............(((((..(((...))).....))))).......((((((((.((((((....)))))).))))))))..(((((((((..((....))..))))))))).............((((((...))))))..((...((((....((((..((((((.(((((.(((....)))...))).)).))))))...))))...)))).))..

>KT308937 *Betula* *lenta* f. *uber* isolate 1 5.8S ribosomal RNA gene and internal transcribed spacer 1, partial sequence; 5.8S ribosomal RNA gene, complete sequence; and internal transcribed spacer 2, partial sequence

UCGAAACCUGCCCAGCAGAACGACCCGUGAACAUGUUGAAACAACUGGGGGCGGGGGGCGAUCUCGCCCCGUGCCCCCGAACGGCAGGGAGACACUCGUGCAUCCCUGCCGAACAACGAACCCCGGCGCGGUCUGCGCCAAGGAACUUUAACGAAAGAGUGCCUCCGGCCGUCUCGGAAACGGUGUGCGUGCGGGAGGCGAAUCUUGUCUAGAACCAU

............(((((..(((...))).....))))).......((((((((.((((((....)))))).))))))))..(((((((((..((....))..))))))))).............((((((...))))))..((...((((....((((..((((((.(((((.(((....)))...))).)).))))))...))))...)))).))..

>KT308936 *Betula* *lenta* 5.8S ribosomal RNA gene and internal transcribed spacer 1, partial sequence; 5.8S ribosomal RNA gene, complete sequence; and internal transcribed spacer 2, partial sequence

UCGAAACCUGCCCAGCAGAACGACCCGUGAACAUGUUGAAACAACUGGGGGCGGGGGGCGAUCUCGCCCCGUGCCCCCGAACGGCAGGGAGACACUCGUGCAUCCCUGCCGAACAACGAACCCCGGCGCGGUCUGCGCCAAGGAACUUUAACGAAAGAGUGCCUCCGGCCGUCUCGGAAACGGUGUGCGUGCGGGAGGCGAAUCUUGUCUAGAACCAU

............(((((..(((...))).....))))).......((((((((.((((((....)))))).))))))))..(((((((((..((....))..))))))))).............((((((...))))))..((...((((....((((..((((((.(((((.(((....)))...))).)).))))))...))))...)))).))..

>KT308935 *Betula* *grossa* isolate 2 5.8S ribosomal RNA gene and internal transcribed spacer 1, partial sequence; 5.8S ribosomal RNA gene, complete sequence; and internal transcribed spacer 2, partial sequence

UCGAAACCUGCCCAGCAGAACGACCCGUGAACCUGUUGAAACAACUGGGGGUGGGGGGCGAUCUCGCCCCUUGCCCCCGAACGGUAGGGAGACACUUGUGCAUCCCUGCCGAACAACGAACCCCGGCGCGGUCUGCGCCAAGGAACUUUAACGAAAGAGUGCCUCCGGCCGCCUCGGAAACGGUGUGCGUGCGGGAGGCGAAUCUUGUCUAGAACCAU

............((((((.(((...)))....)))))).......(((((((((((((((....)))))))))))))))..(((((((((..((....))..))))))))).............((((((...))))))..((...((((....((((..((((((.(((((.(((....)))...))).)).))))))...))))...)))).))..

>KT308934 *Betula* *grossa* isolate 1 5.8S ribosomal RNA gene and internal transcribed spacer 1, partial sequence; 5.8S ribosomal RNA gene, complete sequence; and internal transcribed spacer 2, partial sequence

UCGAAACCUGCCCAGCAGAACGACCCGUGAACCUGUUGAAACAACUGGGGGUGGGGGGCGAUCUCGCCCCUUGCCCCCGAACGGUAGGGAGACACUUGUGCAUCCCUGCCGAACAACGAACCCCGGCGCGGUCUGCGCCAAGGAACUUUAACGAAAGAGUGCCUCCGGCCGCCUCGGAAACGGUGUGCGUGCGGGAGGCGAAUCUUGUCUAGAACCAU

............((((((.(((...)))....)))))).......(((((((((((((((....)))))))))))))))..(((((((((..((....))..))))))))).............((((((...))))))..((...((((....((((..((((((.(((((.(((....)))...))).)).))))))...))))...)))).))..

>KT308933 *Betula* *megrelica* isolate 2 5.8S ribosomal RNA gene and internal transcribed spacer 1, partial sequence; 5.8S ribosomal RNA gene, complete sequence; and internal transcribed spacer 2, partial sequence

UCGAAACCUGCCCAGCAGAACGACCCGUGAACAUGUUGAAACAACUGGGGGCGGGGGGCGAUCUCGCCCCGUGCCCCCGAACGGCAGGGAGACACUCGUGCAUCCCUGCCGAACAACGAACCCCGGCGCGGUCUGCGCCAAGGAACUUUAACGAAAGAGUGCCUCCGGCCGCCUCGGAAACGGUGUGCGUGCGGGAGGCGAAUCUUGUCUAGAACCAU

............(((((..(((...))).....))))).......((((((((.((((((....)))))).))))))))..(((((((((..((....))..))))))))).............((((((...))))))..((...((((....((((..((((((.(((((.(((....)))...))).)).))))))...))))...)))).))..

>KT308932 *Betula* *megrelica* isolate 1 5.8S ribosomal RNA gene and internal transcribed spacer 1, partial sequence; 5.8S ribosomal RNA gene, complete sequence; and internal transcribed spacer 2, partial sequence

UCGAAACCUGCCCAGCAGAACGACCCGUGAACAUGUUGAAACAACUGGGGGCGGGGGGCGAUCUCGCCCCGUGCCCCCGAACGGCAGGGAGACACUCGUGCAUCCCUGCCGAACAACGAACCCCGGCGCGGUCUGCGCCAAGGAACUUUAACGAAAGAGUGCCUCCGGCCGCCUCGGAAACGGUGUGCGUGCGGGAGGCGAAUCUUGUCUAGAACCAU

............(((((..(((...))).....))))).......((((((((.((((((....)))))).))))))))..(((((((((..((....))..))))))))).............((((((...))))))..((...((((....((((..((((((.(((((.(((....)))...))).)).))))))...))))...)))).))..

>KT308931 *Betula* *medwediewii* isolate 2 5.8S ribosomal RNA gene and internal transcribed spacer 1, partial sequence; 5.8S ribosomal RNA gene, complete sequence; and internal transcribed spacer 2, partial sequence

UCGAAACCUGCCCAGCAGAACGACCCGUGAACAUGUUGAAACAACUGGGGGCGGGGGGCGAUCUCGCCCCGUGCCCCCGAACGGCAGGGAGACACUCGUGCAUCCCUGCCGAACAACGAACCCCGGCGCGGUCUGCGCCAAGGAACUUUAACGAAAGAGUGCCUCCGGCCGCCUCGGAAACGGUGUGCGUGCGGGAGGCGAAUCUUGUCUAGAACCAU

............(((((..(((...))).....))))).......((((((((.((((((....)))))).))))))))..(((((((((..((....))..))))))))).............((((((...))))))..((...((((....((((..((((((.(((((.(((....)))...))).)).))))))...))))...)))).))..

>KT308930 *Betula* *medwediewii* isolate 1 5.8S ribosomal RNA gene and internal transcribed spacer 1, partial sequence; 5.8S ribosomal RNA gene, complete sequence; and internal transcribed spacer 2, partial sequence

UCGAAACCUGCCCAGCAGAACGACCCGUGAACAUGUUGAAACAACUGGGGGCGGGGGGCGAUCUCGCCCCGUGCCCCCGAACGGCAGGGAGACACUCGUGCAUCCCUGCCGAACAACGAACCCCGGCGCGGUCUGCGCCAAGGAACUUUAACGAAAGAGUGCCUCCGGCCGCCUCGGAAACGGUGUGCGUGCGGGAGGCGAAUCUUGUCUAGAACCAU

............(((((..(((...))).....))))).......((((((((.((((((....)))))).))))))))..(((((((((..((....))..))))))))).............((((((...))))))..((...((((....((((..((((((.(((((.(((....)))...))).)).))))))...))))...)))).))..

>KT308929 *Betula* *insignis* subsp. *fansipanensis* 5.8S ribosomal RNA gene and internal transcribed spacer 1, partial sequence; 5.8S ribosomal RNA gene, complete sequence; and internal transcribed spacer 2, partial sequence

UCGAAACCUGCCCAGCAGAACGACCCGUGAACCUGUUGAAACAUCUGGGGGCGGGGGGCGAUCUCGCCCCGUGCCCCCGAACGGCAGGGAGACACUCGUGCAUCCCUGCCUAACAACGAACCCCGGCGCGGUCUGCGCCAAGGAACUUUAACGAAAGAGUGCCUCCGGCCGCCUCGGAAACGGUGUGCGUGCGGGAGGCGAAUCUUGUCUAGAACCAU

............((((((.(((...)))....)))))).......((((((((.((((((....)))))).))))))))...((((((((..((....))..))))))))..............((((((...))))))..((...((((....((((..((((((.(((((.(((....)))...))).)).))))))...))))...)))).))..

>KT308928 *Betula* *insignis* isolate 2 5.8S ribosomal RNA gene and internal transcribed spacer 1, partial sequence; 5.8S ribosomal RNA gene, complete sequence; and internal transcribed spacer 2, partial sequence

UCGAAACCUGCCCAGCAGAACGACCCGUGAACCUGUUGAAACAUCUGGGGGCGGGGGGCGAUCUCGCCCCGUGCCCCCGAACGGCAGGGAGACACUCGUGCAUCCCUGCCUAACAACGAACCCCGGCGCGGUCUGCGCCAAGGAACUUUAACGAAAGAGUGCCUCCGGCCGCCUCGGAAACGGUGUGCGUGCGGGAGGCGAAUCUUGUCUAGAACCAU

............((((((.(((...)))....)))))).......((((((((.((((((....)))))).))))))))...((((((((..((....))..))))))))..............((((((...))))))..((...((((....((((..((((((.(((((.(((....)))...))).)).))))))...))))...)))).))..

>KT308927 *Betula* *insignis* isolate 1 5.8S ribosomal RNA gene and internal transcribed spacer 1, partial sequence; 5.8S ribosomal RNA gene, complete sequence; and internal transcribed spacer 2, partial sequence

UCGAAACCUGCCCAGCAGAACGACCCGUGAACCUGUUGAAACAUCUGGGGGCGGGGGGCGAUCUCGCCCCGUGCCCCCGAACGGCAGGGAGACACUCGUGCAUCCCUGCCUAACAACGAACCCCGGCGCGGUCUGCGCCAAGGAACUUUAACGAAAGAGUGCCUCCGGCCGCCUCGGAAACGGUGUGCGUGCGGGAGGCGAAUCUUGUCUAGAACCAU

............((((((.(((...)))....)))))).......((((((((.((((((....)))))).))))))))...((((((((..((....))..))))))))..............((((((...))))))..((...((((....((((..((((((.(((((.(((....)))...))).)).))))))...))))...)))).))..

>KT308926 *Betula* *murrayana* 5.8S ribosomal RNA gene and internal transcribed spacer 1, partial sequence; 5.8S ribosomal RNA gene, complete sequence; and internal transcribed spacer 2, partial sequence

UCGAAACCUGCCCAGCAGAACGACCCGUGAACCUGUUGAAACAACUGGGGGCGGGGGGCGAUCUCGCCCCGUGCCCCCGAACGGCAGGGAGACACUCGUGCAUCCCUGCCGAACAACGAACCCCGGCGCGGUCUGCGCCAAGGAACUUUAACGAAAGAGUGCCUCCGGCCGCCUCGGAAACGGUGUGCGUGCGGGAGGCGAAUCUUGUCUAGAACCAC

............((((((.(((...)))....)))))).......((((((((.((((((....)))))).))))))))..(((((((((..((....))..))))))))).............((((((...))))))..((...((((....((((..((((((.(((((.(((....)))...))).)).))))))...))))...)))).))..

>KT308925 *Betula* *alleghaniensis* 5.8S ribosomal RNA gene and internal transcribed spacer 1, partial sequence; 5.8S ribosomal RNA gene, complete sequence; and internal transcribed spacer 2, partial sequence

UCGAAACCUGCCCAGCAGAACGACCCGUGAACCUGUUGAAACAACUGGGGGCGGGGGGCGAUCUCGCCCCGUGCCCCCGAACGGCAGGGAGACACUCGUGCAUCCCUGCCGAACAACGAACCCCGGCGCGGUCUGCGCCAAGGAACUUUAACGAAAGAGUGCCUCCGGCCGCCUCGGAAACGGUGUGCGUGCGGGAGGCGAAUCUUGUCUAGAACCAC

............((((((.(((...)))....)))))).......((((((((.((((((....)))))).))))))))..(((((((((..((....))..))))))))).............((((((...))))))..((...((((....((((..((((((.(((((.(((....)))...))).)).))))))...))))...)))).))..

>KT308924 *Betula* *albosinensis* isolate 1 5.8S ribosomal RNA gene and internal transcribed spacer 1, partial sequence; 5.8S ribosomal RNA gene, complete sequence; and internal transcribed spacer 2, partial sequence

UCGAAACCUGCCCAGCAGAACGACCCGUGAACCUGUUGAAACAACUGGGGGUGGGGGGCGAUCUCGCCCCUUGCCCYCGAACGGUAGGGAGACACUYGUGCAUCCCUGCCGAACAACGAACCCCGGCGCGGUCYGCGCCAAGGAACUUUAACGAAAGAGUGCCUCCGGCCGCCUCGGAAACGGUGUGCGUGCGGGAGGUGAAUCUUGUCUAGAACCAU

............((((((.(((...)))....)))))).......(((((((((((((((....)))))))))))))))..(((((((((..((....))..))))))))).............((((((...))))))..((...((((....((((..((((((.(((((.(((....)))...))).)).))))))...))))...)))).))..

>KT308923 *Betula* *utilis* var. *occidentalis* isolate 1 5.8S ribosomal RNA gene and internal transcribed spacer 1, partial sequence; 5.8S ribosomal RNA gene, complete sequence; and internal transcribed spacer 2, partial sequence

UCGAAACCUGCCCAGCAGAACGACCCGUGAACCUGUUGAAACAACUGGGGGUGGGGGGCGAUCUCGCCCCUUGCCCCCGAACGGUAGGGAGACACUUGUGCAUCCCUGCCGAACAACGAACCCCGGCGCGGUCCGCGCCAAGGAACUUUAACGAAAGAGUGCCUCCGGCCGCCUCGGAAACGGUGUGCGUGCGGGAGGUGAAUCUUGUCUAGAACCAU

............((((((.(((...)))....)))))).......(((((((((((((((....)))))))))))))))..(((((((((..((....))..))))))))).............((((((...))))))..((...((((....((((..((((((.(((((.(((....)))...))).)).))))))...))))...)))).))..

>KT308921 *Betula* *delavayi* isolate 2 5.8S ribosomal RNA gene and internal transcribed spacer 1, partial sequence; 5.8S ribosomal RNA gene, complete sequence; and internal transcribed spacer 2, partial sequence

UCGAAACCUGCCCAGCAGAAUGACCCGUGAACCUGUUGAAACAACUGGGGGCGGGGGGCGAUCUCGCCCCGUGCCCCCGAAUGGCAGGGAGACACUUGUGCAUCCCUGCUGAACAACGAACCCCGGCGCGGUCUGUGCCAAGGAAAUUUAACGAAAGAGUGCCUCGGGCCGCCUCGGAAACGGUGUGCGUGCGGGAGGUGAAUCUUGUCUAGAACCAU

............((((((.(((...)))....)))))).......((((((((.((((((....)))))).))))))))..(((((((((..((....))..))))))))).............((((((...))))))..((...((((....((((..(((((..(((((.(((....)))...))).))..)))))...))))...)))).))..

>KT308920 *Betula* *schmidtii* isolate 2 5.8S ribosomal RNA gene and internal transcribed spacer 1, partial sequence; 5.8S ribosomal RNA gene, complete sequence; and internal transcribed spacer 2, partial sequence

UCGAAACCUGCCCAGCAGAAUGACCCGUGAACUUGUUGAAACAACUGGGGGCGGGGGGCGAUCUCGCCCCGUGCCCCCGAACGGUAGGGAGACACUUGUGCAUCCCUGCCGAACAACGAACCCCGGCGCGGUCUGCGCCAAGGAACUUUAACGAAAGAGUGCCUCCAGCCGCCUCGGAAACGGUGUGCGUGCGGGAGGUGAAUCUUGUCUAGAACCAU

............((((((.(((...)))....)))))).......((((((((.((((((....)))))).))))))))..(((((((((..((....))..))))))))).............((((((...))))))..((...((((....((((..((((((.(((((.(((....)))...))).)).))))))...))))...)))).))..

>KT308919 *Betula* *schmidtii* isolate 1 5.8S ribosomal RNA gene and internal transcribed spacer 1, partial sequence; 5.8S ribosomal RNA gene, complete sequence; and internal transcribed spacer 2, partial sequence

UCGAAACCUGCCCAGCAGAAUGACCCGUGAACUUGUUGAAACAACUGGGGGCGGGGGGCGAUCUCGCCCCGUGCCCCCGAACGGUAGGGAGACACUUGUGCAUCCCUGCCGAACAACGAACCCCGGCGCGGUCUGCGCCAAGGAACUUUAACGAAAGAGUGCCUCCAGCCGCCUCGGAAACGGUGUGCGUGCGGGAGGUGAAUCUUGUCUAGAACCAU

............((((((.(((...)))....)))))).......((((((((.((((((....)))))).))))))))..(((((((((..((....))..))))))))).............((((((...))))))..((...((((....((((..((((((.(((((.(((....)))...))).)).))))))...))))...)))).))..

>KT308918 *Betula* *chinensis* isolate 2 5.8S ribosomal RNA gene and internal transcribed spacer 1, partial sequence; 5.8S ribosomal RNA gene, complete sequence; and internal transcribed spacer 2, partial sequence

UCGAAACCUGCCCAGCAGAACGACCCGUGAACAUGUUGAAACAACUGGGGGCGGGGGGCGUUCUCGCCCCGUGCCCCCGAACGGUAGGGAGACACUCGUGCAUCCCUGCCGAACAACGAACCCCGGCGCGGUCUGCGCCAAGGAACUUUAACGAAAGAGUGCCUCCGGCCGCCUCGGAAACGGUGUGCGUGCGGGAGGUGAAUCUUGUCUAGAACCAU

............(((((..(((...))).....))))).......((((((((.((((((....)))))).))))))))..(((((((((..((....))..))))))))).............((((((...))))))..((...((((....((((..((((((.(((((.(((....)))...))).)).))))))...))))...)))).))..

>KT308917 *Betula* *chinensis* isolate 1 5.8S ribosomal RNA gene and internal transcribed spacer 1, partial sequence; 5.8S ribosomal RNA gene, complete sequence; and internal transcribed spacer 2, partial sequence

UCGAAACCUGCCCAGCAGAACGACCCGUGAACAUGUUGAAACAACUGGGGGCGGGGGGCGAUCUCGCCCCGUGCCCCCGAACGGUAGGGAGACACUCGUGCAUCCCUGCCGAACAACGAACCCCGGCGCGGUCUGCGCCAAGGAACUUUAACGAAAGAGUGCCUCCGGCCGCCUCGGAAACGGUGUGCGUGCGGGAGGUGAAUCUUGUCUAGAACCAU

............(((((..(((...))).....))))).......((((((((.((((((....)))))).))))))))..(((((((((..((....))..))))))))).............((((((...))))))..((...((((....((((..((((((.(((((.(((....)))...))).)).))))))...))))...)))).))..

>KT308916 *Betula* *chichibuensis* isolate 2 5.8S ribosomal RNA gene and internal transcribed spacer 1, partial sequence; 5.8S ribosomal RNA gene, complete sequence; and internal transcribed spacer 2, partial sequence

UCGAAACCUGCCCAGCAGAAUGACCCGUGAACCUGUUGAAACAACUGGGGGCGGGGGGCGAUCUCGCCCCGUGCCCCCGAACGGUAGGGAGACACUUGUGCAUCCCUGCUGAACAACGAACCCCGGCGCGGUCUGCGCCAAGGAACUUUAACGAAAGAGUGCCUCCGGCCGCCUCGGAAACGGUGUGCGUGCGGGAGGUGAAUCUUGUCUAGAACCAU

............((((((.(((...)))....)))))).......((((((((.((((((....)))))).))))))))..(((((((((..((....))..))))))))).............((((((...))))))..((...((((....((((..((((((.(((((.(((....)))...))).)).))))))...))))...)))).))..

>KT308915 *Betula* *chichibuensis* isolate 1 5.8S ribosomal RNA gene and internal transcribed spacer 1, partial sequence; 5.8S ribosomal RNA gene, complete sequence; and internal transcribed spacer 2, partial sequence

UCGAAACCUGCCCAGCAGAAUGACCCGUGAACCUGUUGAAACAACUGGGGGCGGGGGGCGAUCUCGCCCCGUGCCCCCGAACGGUAGGGAGACACUUGUGCAUCCCUGCUGAACAACGAACCCCGGCGCGGUCUGCGCCAAGGAACUUUAACGAAAGAGUGCCUCCGGCCGCCUCGGAAACGGUGUGCGUGCGGGAGGUGAAUCUUGUCUAGAACCAU

............((((((.(((...)))....)))))).......((((((((.((((((....)))))).))))))))..(((((((((..((....))..))))))))).............((((((...))))))..((...((((....((((..((((((.(((((.(((....)))...))).)).))))))...))))...)))).))..

>KT308914 *Betula* *calcicola* 5.8S ribosomal RNA gene and internal transcribed spacer 1, partial sequence; 5.8S ribosomal RNA gene, complete sequence; and internal transcribed spacer 2, partial sequence

UCGAAACCUGCCCAGCAGAAUGACCCGUGAACCUGUUGAAACAACUGGGGGCGGGGGGCGAUCUCGCCCCGUGCCCCCGAAUGGCAGGGAGACACUUGUGCAUCCCUGCUGAACAACGAACCCCGGCGCGGUCUGUGCCAAGGAAAUUUAACGAAAGAGUGCCUCUGGCCGCCUCGGAAACGGUGUGCGUGCGGGAGGUGAAUCUUGUCUAGAACCAU

............((((((.(((...)))....)))))).......((((((((.((((((....)))))).))))))))..(((((((((..((....))..))))))))).............((((((...))))))..((...((((....((((..((((((.(((((.(((....)))...))).)).))))))...))))...)))).))..

>KT308913 *Betula* *delavayi* isolate 1 5.8S ribosomal RNA gene and internal transcribed spacer 1, partial sequence; 5.8S ribosomal RNA gene, complete sequence; and internal transcribed spacer 2, partial sequence

UCGAAACCUGCCCAGCAGAAUGACCCGUGAACCUGUUGAAACAACUGGGGGCGGGGGGCGAUCUCGCCCCGUGCCCCCGAAUGGCAGGGAGACACUUGUGCAUCCCUGCUGAACAACGAACCCCGGCGCGGUCUGUGCCAAGGAAAUUUAACGAAAGAGUGCCUCCGGCCGCCUCGGAAACGGUGUGCGUGCGGGAGGUGAAUCUUGUCUAGAACCAU

............((((((.(((...)))....)))))).......((((((((.((((((....)))))).))))))))..(((((((((..((....))..))))))))).............((((((...))))))..((...((((....((((..((((((.(((((.(((....)))...))).)).))))))...))))...)))).))..

>KT308912 *Betula* *bomiensis* isolate 2 5.8S ribosomal RNA gene and internal transcribed spacer 1, partial sequence; 5.8S ribosomal RNA gene, complete sequence; and internal transcribed spacer 2, partial sequence

UCGAAACCUGCCCAGCAGAACGACCCGUGAACCUGUUGAAACAACUGGGGGCGGGGGGCGAUCUCGCCCCGUGCCUCCGAACGGUAGGGAGACACUCGUGCAUCCCUGCUGAACAACGAACCCCGGCGCGGUCUGCGCCAAGGAACUUUAACGAAAGAGUGCCUCCGGCCGCCUCGGAAACGGUGUGCGUGCGGGAGGUGAAUCUUGUCUAGAACCAU

............((((((.(((...)))....)))))).......((((((((.((((((....)))))).))))))))..(((((((((..((....))..))))))))).............((((((...))))))..((...((((....((((..((((((.(((((.(((....)))...))).)).))))))...))))...)))).))..

>KT308911 *Betula* *bomiensis* isolate 1 5.8S ribosomal RNA gene and internal transcribed spacer 1, partial sequence; 5.8S ribosomal RNA gene, complete sequence; and internal transcribed spacer 2, partial sequence

UCGAAACCUGCCCAGCAGAACGACCCGUGAACCUGUUGAAACAACUGGGGGCGGGGGGCGAUCUCGCCCCGUGCCUCCGAACGGUAGGGAGACACUCGUGCAUCCCUGCUGAACAACGAACCCCGGCGCGGUCUGCGCCAAGGAACUUUAACGAAAGAGUGCCUCCGGCCGCCUCGGAAACGGUGUGCGUGCGGGAGGUGAAUCUUGUCUAGAACCAU

............((((((.(((...)))....)))))).......((((((((.((((((....)))))).))))))))..(((((((((..((....))..))))))))).............((((((...))))))..((...((((....((((..((((((.(((((.(((....)))...))).)).))))))...))))...)))).))..

>KT308910 *Betula* *potaninii* isolate 2 5.8S ribosomal RNA gene and internal transcribed spacer 1, partial sequence; 5.8S ribosomal RNA gene, complete sequence; and internal transcribed spacer 2, partial sequence

UCGAAACCUGCCCAGCAGAAUGACCCGUGAACCUGUUGAAACAACUGGGGGCGGGGGGCGAUCUCGCCCCGUGCCCCCGAAUGGCAGGGAGACACUUGUGCAUCCCUGCUGAACAACGAACCCCGGCGCGGUCUGUGCCAAGGAAAUUUAACGAAAGAGUGCCUCUGGCCGCCUCGGAAACGGUGUGCGUGCGGGAGGUGAAUCUUGUCUAGAACCAU

............((((((.(((...)))....)))))).......((((((((.((((((....)))))).))))))))..(((((((((..((....))..))))))))).............((((((...))))))..((...((((....((((..((((((.(((((.(((....)))...))).)).))))))...))))...)))).))..

>KT308909 *Betula* *potaninii* isolate 1 5.8S ribosomal RNA gene and internal transcribed spacer 1, partial sequence; 5.8S ribosomal RNA gene, complete sequence; and internal transcribed spacer 2, partial sequence

UCGAAACCUGCYCAGCAGAAUGACCCGUGAACCUGUUGAAACAACUGGGGGCGGGGGGCGAUCUCGCCCCGUGCCCCCGAAUGGCAGGGAGACACUUGUGCAUCCCUGCUGAACAACGAACCCCGGCGCGGUCUGUGCCAAGGAAAUUUAACGAAAGAGUGCCUCUGGCCGCCUCGGAAACGGUGUGCGUGCGGGAGGUGAAUCUUGUCUAGAACCAU

............((((((.(((...)))....)))))).......((((((((.((((((....)))))).))))))))..(((((((((..((....))..))))))))).............((((((...))))))..((...((((....((((..((((((.(((((.(((....)))...))).)).))))))...))))...)))).))..

>KT308908 *Betula* *corylifolia* isolate 2 5.8S ribosomal RNA gene and internal transcribed spacer 1, partial sequence; 5.8S ribosomal RNA gene, complete sequence; and internal transcribed spacer 2, partial sequence

UCGAAACCUGCCCAGCAGAACGACCCGUGAACCUGUUGAAACAACUGGGGGCGGGGGGCGAUCUCGCCCCGUGCCCCCGAACGGUAGGGAGACACUCGUGCAUCCCUGCCGAACAACGAACCCCGGCGCGGUCUGCGCCAAGGAACUUUAACGAAAGAAUGCCUCCGGCCGCCUCGGAAACGGUGUGCGUGCGGGAGGUGAAUCUUGUCUAGAACCAU

............((((((.(((...)))....)))))).......((((((((.((((((....)))))).))))))))..(((((((((..((....))..))))))))).............((((((...))))))..((...((((....((((..((((((.(((((.(((....)))...))).)).))))))...))))...)))).))..

>KT308907 *Betula* *corylifolia* isolate 1 5.8S ribosomal RNA gene and internal transcribed spacer 1, partial sequence; 5.8S ribosomal RNA gene, complete sequence; and internal transcribed spacer 2, partial sequence

UCGAAACCUGCCCAGCAGAACGACCCGUGAACCUGUUGAAACAACUGGGGGCGGGGGGCGAUCUCGCCCCGUGCCCCCGAACGGUAGGGAGACACUCGUGCAUCCCUGCCGAACAACGAACCCCGGCGCGGUCUGCGCCAAGGAACUUUAACGAAAGAAUGCCUCCGGCCGCCUCGGAAACGGUGUGCGUGCGGGAGGUGAAUCUUGUCUAGAACCAU

............((((((.(((...)))....)))))).......((((((((.((((((....)))))).))))))))..(((((((((..((....))..))))))))).............((((((...))))))..((...((((....((((..((((((.(((((.(((....)))...))).)).))))))...))))...)))).))..

>KT308906 *Betula* *fargesii* 5.8S ribosomal RNA gene and internal transcribed spacer 1, partial sequence; 5.8S ribosomal RNA gene, complete sequence; and internal transcribed spacer 2, partial sequence

UCGAAACCUGCCCAGCAGAACGACCCGUGAACAUGUUGAAACAACUGGGGGCGGGGGGCGAUCUCGCCCCGUGCCCCCGAACGGUAGGGAGACACUCGUGCAUCCCUGCCGAACAACGAACCCCGGCGCGGUCUGCGCCAAGGAACUUUAACGAAAGAGUGCCUCCGGCCGCCUCGGAAACGGUGUGCGUGCGGGAGGUGAAUCUUGUCUAGAACCAU

............(((((..(((...))).....))))).......((((((((.((((((....)))))).))))))))..(((((((((..((....))..))))))))).............((((((...))))))..((...((((....((((..((((((.(((((.(((....)))...))).)).))))))...))))...)))).))..

>KT308905 *Betula* *globispica* isolate 2 5.8S ribosomal RNA gene and internal transcribed spacer 1, partial sequence; 5.8S ribosomal RNA gene, complete sequence; and internal transcribed spacer 2, partial sequence

UCGAAACCUGCCCAGCAGAACGACCCGUGAACAUGUUGAAACAACUGGGGGCGGGGGGCGAUCUCGCCCCGUGCCCCCGAACGGUAGGGAGACACUCGUGCAUCCCUGCCGAACAACGAACCCCGGCGCGGUCUGCGCCAAGGAACUUUAACGAAAGAGUGCCUCCGGCCGCCUCGGAAACGGUGUGCGUGCGGGAGGUGAAUCUUGUCUAGAACCAU

............(((((..(((...))).....))))).......((((((((.((((((....)))))).))))))))..(((((((((..((....))..))))))))).............((((((...))))))..((...((((....((((..((((((.(((((.(((....)))...))).)).))))))...))))...)))).))..

>KT308904 *Betula* *globispica* isolate 1 internal transcribed spacer 1, partial sequence; 5.8S ribosomal RNA gene, complete sequence; and internal transcribed spacer 2, partial sequence

UCGAAACCUGCCCAGCAGAACGACCCGUGAACAUGUUGAAACAACUGGGGGCGGGGGGCGAUCUCGCCCCGUGCCCCCGAACGGUAGGGAGACACUCGUGCAUCCCUGCCGAACAACGAACCCCGGCGCGGUCUGCGCCAAGGAACUUUAACGAAAGAGUGCCUCCGGCCGCCUCGGAAACGGUGUGCGUGCGGGAGGUGAAUCUUGUCUAGAACCAU

............(((((..(((...))).....))))).......((((((((.((((((....)))))).))))))))..(((((((((..((....))..))))))))).............((((((...))))))..((...((((....((((..((((((.(((((.(((....)))...))).)).))))))...))))...)))).))..

>JN247411 *Betula* *pendula* voucher MCA 221 internal transcribed spacer 1, partial sequence; 5.8S ribosomal RNA gene, complete sequence; and internal transcribed spacer 2, partial sequence

UCGAAACCUGCCCAGCAGAACGACCCGUGAACCUGUUGAAACAACUGGGGGUGUGGGGCGAUCUCGCCCCUUGCCCCCGAACGGUAGGGAGACACUUGUGCAUCCCUGCCGAACAACGAACCCCGGCGCGGUCCGCGCCAAGGAACUUUAACGAAAGAGUGCCUCCGGCCGCCUCGGAAACGGUGUGCGUGCGGGAGGUGAAUCUUGUCUAGAACCAU

............((((((.(((...)))....)))))).......((((((((.((((((....)))))).))))))))..(((((((((..((....))..))))))))).............((((((...))))))..((...((((....((((..((((((.(((((.(((....)))...))).)).))))))...))))...)))).))..

>FJ011780 *Betula* *utilis* voucher MacAtrher-Tibet Expedition 452 18S ribosomal RNA gene, partial sequence; internal transcribed spacer 1, 5.8S ribosomal RNA gene, and internal transcribed spacer 2, complete sequence; and 26S ribosomal RNA gene, partial sequence

UCGAAACCUGCCCAGCAGAACGACCCGUGAACCUGUUGAAACAACUGGGGGUGGGGGGCGAUCUCGCCCCUUGCCCUCGAACGGUAGGGAGACACUCGUGCAUCCCUGCCGAACAACGAACCCCGGCGCGGUCUGCGCCAAGGAACUUUAACGAAAGAGUGCCUCCGGCCGCCUCGGAAACGGUGUGCGUGCGGGAGGUGAAUCUUGUCUAGAACCAU

............((((((.(((...)))....)))))).......(((((((((((((((....)))))))))))))))..(((((((((..((....))..))))))))).............((((((...))))))..((...((((....((((..((((((.(((((.(((....)))...))).)).))))))...))))...)))).))..

>FJ011779 *Betula* *schmidtii* voucher Lee s.n. 18S ribosomal RNA gene, partial sequence; internal transcribed spacer 1, 5.8S ribosomal RNA gene, and internal transcribed spacer 2, complete sequence; and 26S ribosomal RNA gene, partial sequence

UCGAAACCUGCCCAGCAGAAUGACCCGUGAACUUGUUGAAACAACUGGGGGCGGGGGGCGAUCUCGCCCCGUGCCCCCGAACGGUAGGGAGACACUUGUGCAUCCCUGCCGAACAACGAACCCCGGCGCGGUCUGCGCCAAGGAACUUUAACGAAAGAGUGCCUCCAGCCGCCUCGGAAACGGUGUGCGUGCGGGAGGUGAAUCUUGUCUAGAACCAU

............((((((.(((...)))....)))))).......((((((((.((((((....)))))).))))))))..(((((((((..((....))..))))))))).............((((((...))))))..((...((((....((((..((((((.(((((.(((....)))...))).)).))))))...))))...)))).))..

>FJ011778 *Betula* *platyphylla* voucher Lee s.n. 18S ribosomal RNA gene, partial sequence; internal transcribed spacer 1, 5.8S ribosomal RNA gene, and internal transcribed spacer 2, complete sequence; and 26S ribosomal RNA gene, partial sequence

UCGAAACCUGCCCAGCAGAACGACCCGUGAACCUGUUGAAACAACUGGGGGUGUGGGGCGAUCUCGCCCCUUGCCCCCGAACGGUAGGGAGACACUUGUGCAUCCCUGCCGAACAACGAACCCCGGCGCGGUCCGCGCCAAGGAACUUUAACGAAAGAGUGCCUCCGGCCGCCUCGGAAACGGUGUGCGUGCGGGAGGUGAAUCUUGUCUAGAACCAU

............((((((.(((...)))....)))))).......((((((((.((((((....)))))).))))))))..(((((((((..((....))..))))))))).............((((((...))))))..((...((((....((((..((((((.(((((.(((....)))...))).)).))))))...))))...)))).))..

>FJ011777 *Betula* *pendula* voucher CS03022 18S ribosomal RNA gene, partial sequence; internal transcribed spacer 1, 5.8S ribosomal RNA gene, and internal transcribed spacer 2, complete sequence; and 26S ribosomal RNA gene, partial sequence

UCGAAACCUGUCCAGCAGAACGACCCGUGAACCUGUUGAAACAACGGGGGGUGUGGGGCGAUCUCGCCCCUUGCCCCCGAACGGUAGGGAGACACUUGUGCAUCCCUGCCGAACAACGAACCGCGGCGCGGUCCGCGCCAAGGAACUUUAACGAAAGAGUGCCUCCGGCCGCCUCGGAAACGGUGUGCGUGCGGGAGGUGAAUCUUGUCUAGAACCAU

............((((((.(((...)))....))))))........(((((((.((((((....)))))).)))))))...(((((((((..((....))..))))))))).............((((((...))))))..((...((((....((((..((((((.(((((.(((....)))...))).)).))))))...))))...)))).))..

>FJ011773 *Betula* *davurica* voucher Tibet218 18S ribosomal RNA gene, partial sequence; internal transcribed spacer 1, 5.8S ribosomal RNA gene, and internal transcribed spacer 2, complete sequence; and 26S ribosomal RNA gene, partial sequence

UCGAAACCUGCCCAGCAGAACGACCCGUGAACCUGUUGAAACAACUGGGGGUGGGGGGCGAUCUCGCCCCUUGCCCUCGAACGGUAGGGAGACACUCGUGCAUCCCUGCCGAACAACGAACCCCGGCGCGGUCUGCGCCAAGGAACUUUAACGAAAGAGUGCCUCCGGCCGCCUCGGAAACGGUGUGCGUGCGGGAGGUGAAUCUUGUCUAGAACCAU

............((((((.(((...)))....)))))).......(((((((((((((((....)))))))))))))))..(((((((((..((....))..))))))))).............((((((...))))))..((...((((....((((..((((((.(((((.(((....)))...))).)).))))))...))))...)))).))..

>FJ011772 *Betula* *davurica* voucher UNA63303V 18S ribosomal RNA gene, partial sequence; internal transcribed spacer 1, 5.8S ribosomal RNA gene, and internal transcribed spacer 2, complete sequence; and 26S ribosomal RNA gene, partial sequence

UCGAAACCUGCCCAGCAGAACGACCCGUGAACCUGUUGAAACAACUGGGGGUGGGGGGCGAUCUCGCCCCUUGCCCCCGAACGGUAGGGAGACACUUGUGCAUCCCUGCCGAACAACGAACCCCGGCGCGGUCUGCGCCAAGGAACUUUAACGAAAGAGUGCCUCCCGCCGCCUCGGAAACGGUGUGCGUGCGGGAGGUGAAUCUUGUCUAGAACCAU

............((((((.(((...)))....)))))).......(((((((((((((((....)))))))))))))))..(((((((((..((....))..))))))))).............((((((...))))))..((...((((....((((..((((((.(((((.(((....)))...))).)).))))))...))))...)))).))..

>FJ011771 *Betula* *davurica* voucher UNA64631H 18S ribosomal RNA gene, partial sequence; internal transcribed spacer 1, 5.8S ribosomal RNA gene, and internal transcribed spacer 2, complete sequence; and 26S ribosomal RNA gene, partial sequence

UCGAAACCUGCCCAGCAGAACGACCCGUGAACCUGUUGAAACAACUGGGGGUGGGGGGCGAUCUCGCCCCUUGCCCCCGAACGGUAGGGAGACACUUGUGCAUCCCUGCCGAACAACGAACCCCGGCGCGGUCUGCGCCAAGGAACUUUAACGAAAGAGUGCCUCCCGCCGCCUCGGAAACGGUGUGCGUGCGGGAGGUGAAUCUUGUCUAGAACCAU

............((((((.(((...)))....)))))).......(((((((((((((((....)))))))))))))))..(((((((((..((....))..))))))))).............((((((...))))))..((...((((....((((..((((((.(((((.(((....)))...))).)).))))))...))))...)))).))..

>FJ011770 *Betula* *davurica* voucher Lee s.n. 18S ribosomal RNA gene, partial sequence; internal transcribed spacer 1, 5.8S ribosomal RNA gene, and internal transcribed spacer 2, complete sequence; and 26S ribosomal RNA gene, partial sequence

UCGAAACCUGCCCAGCAGAACGACCCGUGAACCUGUUGAAACAACUGGGGGUGGGGGGCGAUCUCGCCCCUUGCCCCCGAACGGUAGGGAGACACUUGUGCAUCCCUGCCGAACAACGAACCCCGGCGCGGUGUGCGCCAAGGAACUUUAACGAAAGAGUGCCUCCCGCCGCCUCGGAAACGGUGUGCGUGCGGGAGGUGAAUCUUGUCUAGAACCAU

............((((((.(((...)))....)))))).......(((((((((((((((....)))))))))))))))..(((((((((..((....))..))))))))).............((((((...))))))..((...((((....((((..((((((.(((((.(((....)))...))).)).))))))...))))...)))).))..

>AY763114 *Betula* *alnoides* isolate 3464 internal transcribed spacer 1, partial sequence; 5.8S ribosomal RNA gene, complete sequence; and internal transcribed spacer 2, partial sequence

UCGAAACCUGCCCAGCAGAACGACCCGCGAACCUGUUGAAACAACUGGGGGCGGGGGGCRAUCUCGCCCCGUGCCCCCGAACGGUAGGGAGACACUCGUGCAUCCCUGCCGAACAACGAACCCCGGCGCGGUCUGCGCCAAGGAACUUUAACGAAAGAGUGCCUCCGGCCGCCUCGGAAACGGUGUGCGUGCGGGAGGUGAAUCUUGUCUAGAACCAU

............((((((..((...)).....)))))).......((((((((.(((((......))))).))))))))..(((((((((..((....))..))))))))).............((((((...))))))..((...((((....((((..((((((.(((((.(((....)))...))).)).))))))...))))...)))).))..

>AY763113 *Betula* *luminifera* isolate 2841 internal transcribed spacer 1, partial sequence; 5.8S ribosomal RNA gene, complete sequence; and internal transcribed spacer 2, partial sequence

UCGAAACCUGCCCAGCAGAACGACCCGUGAACCUGUUGAAACAACUGGGGGCAGGGGGCGAUCUCGCCCCGUGCCUCCGAACGGUAGGGAGACACUCGUGCAUCCCUGCCGAACAACGAACCCCGGCGCGGUCUGCGCCAAGGAACUUUAACGAAAGAGUGCCUCCGGCCGUCUCGGAAACGGUGUGCGUGCGGGAGGUGAAUCUUGUCUAGAACCAU

............((((((.(((...)))....)))))).......((((((((.((((((....)))))).))))))))..(((((((((..((....))..))))))))).............((((((...))))))..((...((((....((((..((((((.(((((.(((....)))...))).)).))))))...))))...)))).))..

>AY761134 *Betula* *utilis* isolate 2893 internal transcribed spacer 1, partial sequence; 5.8S ribosomal RNA gene, complete sequence; and internal transcribed spacer 2, partial sequence

UCGAAACCUGCCCAGCAGAACGACCCGUGAACCUGUUGAAACAACUGGGGGUGGGGGGCGAUCUCGCCCCUUGCCCUCGAACGGUAGGGAGACACUCGUGCAUCCCUGCCGAACAACGAACCCCGGCGCGGUCUGCGCCAAGGAACUUUAACGAAAGAGUGCCUCCGGCCGCCUCGGAAACGGUGUGCGUGCGGGAGGUGAAUCUUGUCUAGAACCAU

............((((((.(((...)))....)))))).......(((((((((((((((....)))))))))))))))..(((((((((..((....))..))))))))).............((((((...))))))..((...((((....((((..((((((.(((((.(((....)))...))).)).))))))...))))...)))).))..

>AY761133 *Betula* *schmidtii* isolate 2875 internal transcribed spacer 1, partial sequence; 5.8S ribosomal RNA gene, complete sequence; and internal transcribed spacer 2, partial sequence

UCGAAACCUGCCCAGCAGAAUGACCCGUGAACUUGUUGAAACAACUGGGGGCGGGGGGCGAUCUCGCCCCGUGCCCCCGAACGGUAGGGAGACACUUGUGCAUCCCUGCCGAACAACGAACCCCGGCGCGGUCUGCGCCAAGGAACUUUAACGAAAGAGUGCCUCCAGCCGCCUCGGAAACGGUGUGCGUGCGGGAGGUGAAUCUUGUCUAGAACCAU

............((((((.(((...)))....)))))).......((((((((.((((((....)))))).))))))))..(((((((((..((....))..))))))))).............((((((...))))))..((...((((....((((..((((((.(((((.(((....)))...))).)).))))))...))))...)))).))..

>AY761132 *Betula* *raddeana* isolate 3338 internal transcribed spacer 1, partial sequence; 5.8S ribosomal RNA gene, complete sequence; and internal transcribed spacer 2, partial sequence

UCGAAACCUGCCCAGCAGAACGACCCGUGAACCUGUUGAAACAACUGGGGGUGGGGGGC--UCUCGCCCCUUGCCCCCGAACGGUAGGGAGACACUCGUGCAUCCCUGCCGAACAACGAACCCCGGCGCGGUCUGCGCCAAGGAACUUUAACGAAAGAGUGCCUCCCGCCGCCUCGGAAACGGUGUGCGUGCGGGAGGUGAAUCUUGUCUAGAACCAU

............((((((.(((...)))....)))))).......((((((((((((((--....))))))))))))))..(((((((((..((....))..))))))))).............((((((...))))))..((...((((....((((..((((((.(((((.(((....)))...))).)).))))))...))))...)))).))..

>AY761131 *Betula* *pumila* isolate 3246 internal transcribed spacer 1, partial sequence; 5.8S ribosomal RNA gene, complete sequence; and internal transcribed spacer 2, partial sequence

UCGAAACCUGCCCAGCAGAACGACCCGUGAACUUGUUGAAACAACUGGGGGUGGGGGGC--UCUCGCCCCUUGCCCCCGAACGGUAGGGAGACACUUGUGCAUCCCUGCCGAACAACGAACCCCGGCGCGGUCUGCGCCAAGGAACUUUAACGAAAGAGUGCCUCCGGCCGCCUCGGAAACGGUGUGCGUGCGGGAGGUGAAUCUUGUCUAGAACCAU

............((((((.(((...)))....)))))).......((((((((((((((--....))))))))))))))..(((((((((..((....))..))))))))).............((((((...))))))..((...((((....((((..((((((.(((((.(((....)))...))).)).))))))...))))...)))).))..

>AY761130 *Betula* *pubescens* isolate 2895 internal transcribed spacer 1, partial sequence; 5.8S ribosomal RNA gene, complete sequence; and internal transcribed spacer 2, partial sequence

UCGAAACCUGCCCAGCAGAACGACCCGUGAACCUGUUGAAACAACUGGGGGUGGGGGGCGAUCUCGCCCCUUGCCCCCGAACGGUAGGGAGACACUUGUGCAUCCCUGCCGAACAACGAACCCCGGCGCGGUCCGCGCCAAGGAACUUUAACGAAAGAGUGCCUCCGGCCGCCUCGGAAACGGUGUGCGUGCGGGAGGUGAAUCUUGUCUAGAACCAU

............((((((.(((...)))....)))))).......(((((((((((((((....)))))))))))))))..(((((((((..((....))..))))))))).............((((((...))))))..((...((((....((((..((((((.(((((.(((....)))...))).)).))))))...))))...)))).))..

>AY761129 *Betula* *populifolia* isolate 2890 internal transcribed spacer 1, partial sequence; 5.8S ribosomal RNA gene, complete sequence; and internal transcribed spacer 2, partial sequence

UCGAAACCUGCCCAGCAGAACGACCCGUGAACCUGUUGAAACAACUGGGGGUGGGGGGCGAUCUCGCCCCUUGUCCCCGAACGGUAGGGAGACACUUGUGCAUCCCUGCCGAACAACGAACCCCGGCGCGGUCCGCGCCAAGGAACUUUAACGAAAGAGUGCCUCCGGCCGCCUCGGAAACGGUGUGCGUGCGGGAGGUGAAUCUUGUCUAGAACCAU

............((((((.(((...)))....)))))).......(((((((((((((((....)))))))))))))))..(((((((((..((....))..))))))))).............((((((...))))))..((...((((....((((..((((((.(((((.(((....)))...))).)).))))))...))))...)))).))..

>AY761128 *Betula* *platyphylla* isolate 2934 internal transcribed spacer 1, partial sequence; 5.8S ribosomal RNA gene, complete sequence; and internal transcribed spacer 2, partial sequence

UCGAAACCUGCCCAGCAGAACGACCCGUGAACCUGUUGAAACAACUGGGGGUGUGGGGCGAUCUCGCCCCUUGCCCCCGAACGGUAGGGAGACACUUGUGCAUCCCUGCCGAACAACGAACCCCGGCGCGGUCCGCGCCAAGGAACUUUAACGAAAGAGUGCCUCCGGCCGCCUCGGAAACGGUGUGCGUGCGGGAGGUGAAUCUUGUCUAGAACCAU

............((((((.(((...)))....)))))).......((((((((.((((((....)))))).))))))))..(((((((((..((....))..))))))))).............((((((...))))))..((...((((....((((..((((((.(((((.(((....)))...))).)).))))))...))))...)))).))..

>AY761127 *Betula* *pendula* isolate 2902 internal transcribed spacer 1, partial sequence; 5.8S ribosomal RNA gene, complete sequence; and internal transcribed spacer 2, partial sequence

UCGAAACCUGCCCAGCAGAACGACCCGUGAACCUGUUGAAACAACUGGGGGUGUGGGGCGAUCUCGCCCCUUGCCCCCGAACGGUAGGGAGACACUUGUGCAUCCCUGCCGAACAACGAACCCCGGCGCGGUCCGCGCCAAGGAACUUUAACGAAAGAGUGCCUCCGGCCGCCUCGGAAACGGUGUGCGUGCGGGAGGUGAAUCUUGUCUAGAACCAU

............((((((.(((...)))....)))))).......((((((((.((((((....)))))).))))))))..(((((((((..((....))..))))))))).............((((((...))))))..((...((((....((((..((((((.(((((.(((....)))...))).)).))))))...))))...)))).))..

>AY761126 *Betula* *papyrifera* isolate 2892 internal transcribed spacer 1, partial sequence; 5.8S ribosomal RNA gene, complete sequence; and internal transcribed spacer 2, partial sequence

UCGAAACCUGCCCGGCAGAACGACCCGUGAACCUGUUGAAACAACUGGGGGUGGGGGGCGAUCUCGCCCCUUGCCCCCGAACGGUAGGGAGACACUUGUGCAUCCCUGCCGAACAACGAACCCCGGCGCGGUCUGCGCCAAGGAACUUUAACGAAAGAGUGCCUCCCGCCGCCUCGGAAACGGUGUGCGUGCGGGAGGUGAAUCUUGUCUAGAACCAU

............((((((.(((...)))....)))))).......(((((((((((((((....)))))))))))))))..(((((((((..((....))..))))))))).............((((((...))))))..((...((((....((((..((((((.(((((.(((....)))...))).)).))))))...))))...)))).))..

>AY761125 *Betula* *occidentalis* isolate 2883 internal transcribed spacer 1, partial sequence; 5.8S ribosomal RNA gene, complete sequence; and internal transcribed spacer 2, partial sequence

UCGAAACCUGCCCAGCAGAACGACCCGUGAACCUGUUGAAACAACUGGGGGYGUGGGGCGAUCUCGCCCCUUGCCCCCGAACGGUAGGGAGACACUUGUGCAUCCCUGCCGAACAACGAACCCCGGCGCGGUCUGCGCCAAGGAACUUUAACGAAAGAGUGCCUCCGGCCGCCUCGGAAACGGUGUGCGUGCGGGAGGUGAAUCUUGUCUAGAACCAU

............((((((.(((...)))....)))))).......((((((((.((((((....)))))).))))))))..(((((((((..((....))..))))))))).............((((((...))))))..((...((((....((((..((((((.(((((.(((....)))...))).)).))))))...))))...)))).))..

>AY761124 *Betula* *nigra* isolate 2927 internal transcribed spacer 1, partial sequence; 5.8S ribosomal RNA gene, complete sequence; and internal transcribed spacer 2, partial sequence

UCGAAACCUGCCCAGCAGAACGACCCGUGAACCUGUUGAAACAACUGGGGGUGGGGGGCGAUCUCGCCCCGUGCCUCCGAACGGUAGGGAGACACUUGUGCAUCCCUGCUGAACAACGAACCCCGGCGCGGUCUGCGCCAAGGAACUUUAACGAAAGAGUGCCUCCGGCCGCCUCGGAAACGGUGUGCGUGCAGGAAAACAAUCUUGUCUAGAACCAC

............((((((.(((...)))....)))))).......((((((((.((((((....)))))).))))))))..(((((((((..((....))..))))))))).............((((((...))))))..((...((((....((((.((..(((.(((((.(((....)))...))).)).)))...)).))))...)))).))..

>AY761122 *Betula* *nana* isolate 3337 internal transcribed spacer 1, partial sequence; 5.8S ribosomal RNA gene, complete sequence; and internal transcribed spacer 2, partial sequence

UCGAAACCUGCCCAGCAGAACGACCCGUGAACCUGUUGAAACAACUGGGGGUGUGGGGCGAUCUCGCCCCUUGYCCCCGAACGGUAGGGAGACACUUGUGCAUCCCUGCCGAACAACGAACCCCGGCGCGGUCYGCGCCAAGGAACUUUAACGAAAGAGUGCCUCCGGCCGCCUCGGAAACGGUGUGCGUGCGGGAGGUGAAUCUUGUCUAGAACCAU

............((((((.(((...)))....)))))).......((((((((.((((((....)))))).))))))))..(((((((((..((....))..))))))))).............((((((...))))))..((...((((....((((..((((((.(((((.(((....)))...))).)).))))))...))))...)))).))..

>AY761121 *Betula* *michauxii* isolate 3406 internal transcribed spacer 1, partial sequence; 5.8S ribosomal RNA gene, complete sequence; and internal transcribed spacer 2, partial sequence

UCGAAACCUGCCCAGCAGAACGACCCGUGAACCUGUUGAAACAACUGGGGGCGGGGGGCGAUCUCGCCCCGUGCCCCCGAACGGUAGGGAGACACUCGUGCAUCCCUGCCGAACAACGAACCCCGGCGCGGUCUGCGCCAAGGAACUUUAACGAAAGAGUGCCUCCGGCCGCCUCGGAAACGGUGUGCGUGCGGGAGGUGAAUCUUGUCUAGAACCAU

............((((((.(((...)))....)))))).......((((((((.((((((....)))))).))))))))..(((((((((..((....))..))))))))).............((((((...))))))..((...((((....((((..((((((.(((((.(((....)))...))).)).))))))...))))...)))).))..

>AY761120 *Betula* *medwediewii* isolate 3465 internal transcribed spacer 1, partial sequence; 5.8S ribosomal RNA gene, complete sequence; and internal transcribed spacer 2, partial sequence

UCGAAACCUGCCCAGCAGAACGACCCGUGAACAUGUUGAAACAACUGGGGGCGGGGGGCGAUCUCGCCCCGUGCCCCCGAACGGCAGGGAGACACUCGUGCAUCCCUGCCGAACAACGAACCCCGGCGCGGUCUGCGCCAAGGAACUUUAACGAAAGAGUGCCUCCGGCCGCCUCGGAAACGGUGUGCGUGCGGGAGGCGAAUCUUGUCUAGAACCAU

............(((((..(((...))).....))))).......((((((((.((((((....)))))).))))))))..(((((((((..((....))..))))))))).............((((((...))))))..((...((((....((((..((((((.(((((.(((....)))...))).)).))))))...))))...)))).))..

>AY761119 *Betula* *maximovicziana* isolate 3463 internal transcribed spacer 1, partial sequence; 5.8S ribosomal RNA gene, complete sequence; and internal transcribed spacer 2, partial sequence

UCGAAACCUGCCCAGCAGAACGACCCGUGAACCUGUUGAAACAACUGGGGGUGGGGGGCGAUCUCGCCCCUUGCCCCCGAACGGUAGGGAGACACUCGUGCAUCCCUGCCGAACAACGAACCCCGGCGCGGUCUGCGCCAAGGAACUUUAACGAAAGAGUGCCUCCGGCCGCCUCGGAAACGGUGUGCGUGCGGGAGGUGAAUCUUGUCUAGAACCAU

............((((((.(((...)))....)))))).......(((((((((((((((....)))))))))))))))..(((((((((..((....))..))))))))).............((((((...))))))..((...((((....((((..((((((.(((((.(((....)))...))).)).))))))...))))...)))).))..

>AY761118 *Betula* *maximovicziana* isolate 2932 internal transcribed spacer 1, partial sequence; 5.8S ribosomal RNA gene, complete sequence; and internal transcribed spacer 2, partial sequence

UCGAAACCUGCCCAGCAGAACGACCCGUGAACCUGUUGAAACAACUGGGGGUGGGGGGCGAUCUCGCCCCUUGCCCCCGAACGGUAGGGAGACACUCGUGCAUCCCUGCCGAACAACGAACCCCGGCGCGGUCUGCGCCAAGGAACUUUAACGAAAGAGUGCCUCCGGCCGCCUCGGAAACGGUGUGCGUGCGGGAGGUGAAUCUUGUCUAGAACCAU

............((((((.(((...)))....)))))).......(((((((((((((((....)))))))))))))))..(((((((((..((....))..))))))))).............((((((...))))))..((...((((....((((..((((((.(((((.(((....)))...))).)).))))))...))))...)))).))..

>AY761117 *Betula* *luminifera* isolate 3299 internal transcribed spacer 1, partial sequence; 5.8S ribosomal RNA gene, complete sequence; and internal transcribed spacer 2, partial sequence

UCGAAACCUGCCCAGCAGAACGACCCGUGAACCUGUUGAAACAACUGGGGGCAGGGGGCGAUCUCGCCCCGUGCCCCCGAACGGUAGGGAGACACUCGUGCAUCCCUGCCGAACAACGAACCCCGGCGCGGUCUGCGCCAAGGAACUUUAACGAAAGAGUGCCUCCGGCCGUCUCGGAAACGGUGUGCGUGCGGGAGGUGAAUCUUGUCUAGAACCAU

............((((((.(((...)))....)))))).......((((((((.((((((....)))))).))))))))..(((((((((..((....))..))))))))).............((((((...))))))..((...((((....((((..((((((.(((((.(((....)))...))).)).))))))...))))...)))).))..

>AY761116 *Betula* *luminifera* isolate 2828 internal transcribed spacer 1, partial sequence; 5.8S ribosomal RNA gene, complete sequence; and internal transcribed spacer 2, partial sequence

UCGAAACCUGCCCAGCAGAACGACCCGUGAACCUGUUGAAACAACUGGGGGCAGGGGGCGAUCUCGCCCCGUGCCCCCGAACGGUAGGGAGACACUCGUGCAUCCCUGCCGAACAACGAACCCCGGCGCGGUCUGCGCCAAGGAACUUUAACGAAAGAGUGCCUCCGGCCGUCUCGGAAACGGUGUGCGUGCGGGAGGUGAAUCUUGUCUAGAACCAU

............((((((.(((...)))....)))))).......((((((((.((((((....)))))).))))))))..(((((((((..((....))..))))))))).............((((((...))))))..((...((((....((((..((((((.(((((.(((....)))...))).)).))))))...))))...)))).))..

>AY761115 *Betula* *lenta* isolate 2936 internal transcribed spacer 1, partial sequence; 5.8S ribosomal RNA gene, complete sequence; and internal transcribed spacer 2, partial sequence

UCGAAACCUGCCCAGCAGAACGACCCGUGAACAUGUUGAAACAACUGGGGGCGGGGGGCGAUCUCGCCCCGUGCCCCCGAACGGCAGGGAGACACUCGUGCAUCCCUGCCGAACAACGAACCCCGGCGCGGUCUGCGCCAAGGAACUUUAACGAAAGAGUGCCUCCGGCCGUCUCGGAAACGGUGUGCGUGCGGGAGGCGAAUCUUGUCUAGAACCAU

............(((((..(((...))).....))))).......((((((((.((((((....)))))).))))))))..(((((((((..((....))..))))))))).............((((((...))))))..((...((((....((((..((((((.(((((.(((....)))...))).)).))))))...))))...)))).))..

>AY761114 *Betula* *humilis* isolate 2894 internal transcribed spacer 1, partial sequence; 5.8S ribosomal RNA gene, complete sequence; and internal transcribed spacer 2, partial sequence

UCGAAACCUGCCCAGCAGAACGACCCGUGAACCUGUUGAAACAACUGGGGGUGUGGGGCGAUCUCGCCCCUUGCCCCCGAACGGUAGGGAGACACUUGUGCAUCCCUGCCGAACAACGAACCCCGGCGCGGUCCGCGCCAAGGAACUUUAACGAAAGAGUGCCUCCGGCCGCCUCGGAAACGGUGUGCGUGCGGGAGGUGAAUCUUGUCUAGAACCAU

............((((((.(((...)))....)))))).......((((((((.((((((....)))))).))))))))..(((((((((..((....))..))))))))).............((((((...))))))..((...((((....((((..((((((.(((((.(((....)))...))).)).))))))...))))...)))).))..

>AY761113 *Betula* *grossa* isolate 3459 internal transcribed spacer 1, partial sequence; 5.8S ribosomal RNA gene, complete sequence; and internal transcribed spacer 2, partial sequence

UCGAAACCUGCCCAGCAGAACGACCCGUGAACCUGUUGAAACAACUGGGGGUGGGGGGCGAUCUCGCCCCUUGCCCCCGAACGGUAGGGAGACACUUGUGCAUCCCUGCCGAACAACGAACCCCGGCGCGGUCUGCGCCAAGGAACUUUAACGAAAGAGUGCCUCCGGCCGCCUCGGAAACGGUGUGCGUGCGGGAGGCGAAUCUUGUCUAGAACCAU

............((((((.(((...)))....)))))).......(((((((((((((((....)))))))))))))))..(((((((((..((....))..))))))))).............((((((...))))))..((...((((....((((..((((((.(((((.(((....)))...))).)).))))))...))))...)))).))..

>AY761112 *Betula* *grossa* isolate 2948 internal transcribed spacer 1, partial sequence; 5.8S ribosomal RNA gene, complete sequence; and internal transcribed spacer 2, partial sequence

UCGAAACCUGCCCAGCAGAACGACCCGUGAACCUGUUGAAACAACUGGGGGUGGGGGGCGAUCUCGCCCCUUGCCCCCGAACGGUAGGGAGACACUUGUGCAUCCCUGCCGAACAACGAACCCCGGCGCGGUCUGCGCCAAGGAACUUUAACGAAAGAGUGCCUCCGGCCGCCUCGGAAACGGUGUGCGUGCGGGAGGCGAAUCUUGUCUAGAACCAU

............((((((.(((...)))....)))))).......(((((((((((((((....)))))))))))))))..(((((((((..((....))..))))))))).............((((((...))))))..((...((((....((((..((((((.(((((.(((....)))...))).)).))))))...))))...)))).))..

>AY761111 *Betula* *globispica* isolate 2942 internal transcribed spacer 1, partial sequence; 5.8S ribosomal RNA gene, complete sequence; and internal transcribed spacer 2, partial sequence

UCGAAACCUGCCCAGCAGAACGACCCGUGAACAUGUUGAAACAACUGGGGGCGGGGGGCGAUCUCGCCCCGUGCCCCCGAACGGUAGGGAGACACUCGUGCAUCCCUGCCGAACAACGAACCCCGGCGCGGUCUGCGCCAAGGAACUUUAACGAAAGAGUGCCUCCGGCCGCCUCGGAAACGGUGUGCGUGCGGGAGGUGAAUCUUGUCUAGAACCAU

............(((((..(((...))).....))))).......((((((((.((((((....)))))).))))))))..(((((((((..((....))..))))))))).............((((((...))))))..((...((((....((((..((((((.(((((.(((....)))...))).)).))))))...))))...)))).))..

>AY761110 *Betula* *glandulosa* isolate 3251 internal transcribed spacer 1, partial sequence; 5.8S ribosomal RNA gene, complete sequence; and internal transcribed spacer 2, partial sequence

UCGAAACCUGCCCAGCAGAACGACCCGUGAACAUGUUGAAACAACUGGGGGCGGGGGGCGAUCUCGCCCCGUGCCCCCGAACGGUAGGGAGACACUCGUGCAUCCCUGCCGAACAACGAACCCCGGCGCGGUCUGCGCCAAGGAACUUUAACGAAAGAGUGCCUCCGGCCGCCUCGGAAACGGUGUGCGUGCGGGAGGUGAAUCUUGUCUAGAACCAU

............(((((..(((...))).....))))).......((((((((.((((((....)))))).))))))))..(((((((((..((....))..))))))))).............((((((...))))))..((...((((....((((..((((((.(((((.(((....)))...))).)).))))))...))))...)))).))..

>AY761109 *Betula* *fruticosa* isolate 3339 internal transcribed spacer 1, partial sequence; 5.8S ribosomal RNA gene, complete sequence; and internal transcribed spacer 2, partial sequence

UCGAAACCUGCCCAGCAGAACGACCCGUGAACCUGUUGAAACAACUGGGGGUGGGGGGCKAUCUCGCCCCUUGCCCCCGAACGGUAGGGAGACACUUGUGCAUCCCUGCCGAACAACGAACCCCGGCGCGGUCUGCGCCAAGGAACUUUAACGAAAGAGUGCCUCCCGCCGCCUCGGAAACGGUGUGCGUGCGGGAGGUGAAUCUUGUCUAGAACCAU

............((((((.(((...)))....)))))).......(((((((((((((((....)))))))))))))))..(((((((((..((....))..))))))))).............((((((...))))))..((...((((....((((..((((((.(((((.(((....)))...))).)).))))))...))))...)))).))..

>AY761108 *Betula* *ermanii* isolate 2961 internal transcribed spacer 1, partial sequence; 5.8S ribosomal RNA gene, complete sequence; and internal transcribed spacer 2, partial sequence

UCGAAACCUGCCCAGCAGAACGACCCGUGAACCUGUUGAAACAACUGGGGGUGGGGGGCGAUCUCGCCCCUUGCCCUCGAACGGUAGGGAGACACUCGUGCAUCCCUGCCGAACAACGAACCCCGGCGCGGUCUGCGCCAAGGAACUUUAACGAAAGAGUGCCUCCGGCCGCCUCGGAAACGGUGUGCGUGCGGGAGGUGAAUCUUGUCUAGAACCAU

............((((((.(((...)))....)))))).......(((((((((((((((....)))))))))))))))..(((((((((..((....))..))))))))).............((((((...))))))..((...((((....((((..((((((.(((((.(((....)))...))).)).))))))...))))...)))).))..

>AY761107 *Betula* *delavayi* isolate 3462 internal transcribed spacer 1, partial sequence; 5.8S ribosomal RNA gene, complete sequence; and internal transcribed spacer 2, partial sequence

UCGAAACCUGCCCAGCAGAAUGACCCGUGAACCUGUUGAAACAACUGGGGGCGGGGGGCGAUCUCGCCCCGUGCCCCCGAAUGGCAGGGAGACACUUGUGCAUCCCUGCUGAACAACGAACCCCGGCGCGGUCUGUGCCAAGGAAAUUUAACGAAAGAGUGCCUCUGGCCGCCUCGGAAACGGUGUGCGUGCGGGAGGUGAAUCUUGUCUAGAACCAU

............((((((.(((...)))....)))))).......((((((((.((((((....)))))).))))))))..(((((((((..((....))..))))))))).............((((((...))))))..((...((((....((((..((((((.(((((.(((....)))...))).)).))))))...))))...)))).))..

>AY761106 *Betula* *corylifolia* isolate 3457 internal transcribed spacer 1, partial sequence; 5.8S ribosomal RNA gene, complete sequence; and internal transcribed spacer 2, partial sequence

UCGAAACCUGCCCAGCAGAACGACCCGUGAACCUGUUGAAACAACUGGGGGCGGGGGGCGAUCUCGCCCCGUGCCCCCGAACGGUAGGGAGACACUCGUGCAUCCCUGCCGAACAACGAACCCCGGCGCGGUCUGCGCCAAGGAACUUUAACGAAAGAAUGCCUCCGGCCGCCUCGGAAACGGUGUGCGUGCGGGAGGUGAAUCUUGUCUAGAACCAU

............((((((.(((...)))....)))))).......((((((((.((((((....)))))).))))))))..(((((((((..((....))..))))))))).............((((((...))))))..((...((((....((((..((((((.(((((.(((....)))...))).)).))))))...))))...)))).))..

>AY761105 *Betula* *chinensis* isolate 2903 internal transcribed spacer 1, partial sequence; 5.8S ribosomal RNA gene, complete sequence; and internal transcribed spacer 2, partial sequence

UCGAAACCUGCCCAGCAGAACGACCCGUGAACCUGUUGAAACAACUGGGGGUGGGGGGCGAUCUCGCCCCUUGCCCCCGAACGGUAGGGAGACACUUGUGCAUCCCUGCCGAACAACGAACCCCGGCGCGGUCUGCGCCAAGGAACUUUAACGAAAGAGUGCCUCCCGCCGCCUCGGAAACGGUGUGCGUGCGGGAGGUGAAUCUUGUCUAGAACCAU

............((((((.(((...)))....)))))).......(((((((((((((((....)))))))))))))))..(((((((((..((....))..))))))))).............((((((...))))))..((...((((....((((..((((((.(((((.(((....)))...))).)).))))))...))))...)))).))..

>AY761104 *Betula* *chichibuensis* isolate 2977 internal transcribed spacer 1, partial sequence; 5.8S ribosomal RNA gene, complete sequence; and internal transcribed spacer 2, partial sequence

UCGAAACCUGCCCAGCAGAAUGACCCGUGAACCUGUUGAAACAACUGGGGGCGGGGGGCGAUCUCGCCCCGUGCCCCCGAACGGUAGGGAGACACUUGUGCAUCCCUGCUGAACAACGAACCCCGGCGCGGUCUGCGCCAAGGAACUUUAACGAAAGAGUGCCUCCGGCCGCCUCGGAAACGGUGUGCGUGCGGGAGGUGAAUCUUGUCUAGAACCAU

............((((((.(((...)))....)))))).......((((((((.((((((....)))))).))))))))..(((((((((..((....))..))))))))).............((((((...))))))..((...((((....((((..((((((.(((((.(((....)))...))).)).))))))...))))...)))).))..

>AY761103 *Betula* *calcicola* isolate 3460 internal transcribed spacer 1, partial sequence; 5.8S ribosomal RNA gene, complete sequence; and internal transcribed spacer 2, partial sequence

UCGAAACCUGCCCAGCAGAAUGACCCGUGAACCUGUUGAAACAACUGGGGGCGGGGGGCGAUCUCGCCCCGUGCCCCCGAAUGGCAGGGAGACACUUGUGCAUCCCUGCUGAACAACGAACCCCGGCGCGGUCUGUGCCAAGGAAAUUUAACGAAAGAGUGCCUCYGGCCGCCUCGGAAACGGUGUGCGUGCGGGAGGUGAAUCUUGUCUAGAACCAU

............((((((.(((...)))....)))))).......((((((((.((((((....)))))).))))))))..(((((((((..((....))..))))))))).............((((((...))))))..((...((((....((((..(((((..(((((.(((....)))...))).))..)))))...))))...)))).))..

>AY761102 *Betula* *apoiensis* isolate 3249 internal transcribed spacer 1, partial sequence; 5.8S ribosomal RNA gene, complete sequence; and internal transcribed spacer 2, partial sequence

UCGAAACCUGCCCAGCAGAACGACCCGUGAACCUGUUGAAACAACUGGGGGCGGGGGGCGAUCUCGCCCCUUGCCCUCGAACGGUAGGGAGACACUUGUGCAUCCCUGCCGAACAACGAACCCCGGCGCGGUCUGCGCCAAGGAACUUUAACGAAAGAGUGCCUCCGGCCGCCUCGGAAACGGUGUGCGUGCGGGAGGUGAAUCUUGUCUAGAACCAU

............((((((.(((...)))....)))))).......(((((((((((((((....)))))))))))))))..(((((((((..((....))..))))))))).............((((((...))))))..((...((((....((((..((((((.(((((.(((....)))...))).)).))))))...))))...)))).))..

>AY761101 *Betula* *alnoides* isolate 3352 internal transcribed spacer 1, partial sequence; 5.8S ribosomal RNA gene, complete sequence; and internal transcribed spacer 2, partial sequence

UCGAAACCUGCCCAGCAGAACGACCCGUGAACCUGUUGAAACAACUGGGGGCGGGGGGCGAUCUCGCCCCGUGCCCCCGAACGGUAGGGAGACACUCGUGCAUCCCUGCCGAACAACGAACCCCGGCGCGGUCUGCGCCAAGGAACUUUAACGAAAGAGUGCCUCCGGCCGCCUCGGAAACGGUGUGCGUGCGGGAGGUGAAUCUUGUCUAGAACCAU

............((((((.(((...)))....)))))).......((((((((.((((((....)))))).))))))))..(((((((((..((....))..))))))))).............((((((...))))))..((...((((....((((..((((((.(((((.(((....)))...))).)).))))))...))))...)))).))..

>AY761100 *Betula* *alleghaniensis* isolate 2880 internal transcribed spacer 1, partial sequence; 5.8S ribosomal RNA gene, complete sequence; and internal transcribed spacer 2, partial sequence

UCGAAACCUGCCCAGCAGAACGACCCGUGAACCUGUUGAAACAACUGGGGGCGGGGGGCGAUCUCGCCCCGUGCCCCCGAACGGCAGGGAGACACUCGUGCAUCCCUGCCGAACAACGAACCCCGGCGCGGUCUGCGCCAAGGAACUUUAACGAAAGAGUGCCUCCGGCCGCCUCGGAAACGGUGUGCGUGCGGGAGGCGAAUCUUGUCUAGAACCAC

............((((((.(((...)))....)))))).......((((((((.((((((....)))))).))))))))..(((((((((..((....))..))))))))).............((((((...))))))..((...((((....((((..((((((.(((((.(((....)))...))).)).))))))...))))...)))).))..

>AY761099 *Betula* *albosinensis* isolate 3018 internal transcribed spacer 1, partial sequence; 5.8S ribosomal RNA gene, complete sequence; and internal transcribed spacer 2, partial sequence

UCGAAACCUGCCCAGCAGAACGACCCGUGAACCUGUUGAAACAACUGGGGGUGGGGGGCGAUCUCGCCCCUUGCCCUCGAACGGUAGGGAGACACUCGUGCAUCCCUGCCGAACAACGAACCCCGGCGCGGUCUGCGCCAAGGAACUUUAACGAAAGAGUGCCUCCGGCCGCCUCGGAAACGGUGUGCGUGCGGGAGGUGAAUCUUGUCUAGAACCAU

............((((((.(((...)))....)))))).......(((((((((((((((....)))))))))))))))..(((((((((..((....))..))))))))).............((((((...))))))..((...((((....((((..((((((.(((((.(((....)))...))).)).))))))...))))...)))).))..

>AY352337 *Betula* *costata* internal transcribed spacer 1, partial sequence; 5.8S ribosomal RNA gene, complete sequence; and internal transcribed spacer 2, partial sequence

UCGAAACCUGCCCAGCAGAACGACCCGUGAACCUGUUGAAACAACUGGGGGCGGGGGGCGAUCUCGCCCCGUGCCCCCGAACGGCAGGGAGACACUCGUGCAUCCCUGCCGAACAACGAACCCCGGCGCGGUCUGCGCCAAGGAACUUUAACGAAAGAGUGCCUCCGGCCGCCUCGGAAACGGUGUGCGUGCGGGAGGCGAAUCUUGUCUAGAACCAC

............((((((.(((...)))....)))))).......((((((((.((((((....)))))).))))))))..(((((((((..((....))..))))))))).............((((((...))))))..((...((((....((((..((((((.(((((.(((....)))...))).)).))))))...))))...)))).))..

>AY352336 *Betula* *nana* internal transcribed spacer 1, partial sequence; 5.8S ribosomal RNA gene, complete sequence; and internal transcribed spacer 2, partial sequence

UCGAAACCUGCCCAGCAGAACGACCCGUGAACCUGUUGAAACAACUGGGGGUGKGGGGCGAUCUCGCCCCUUGCCCCCGAACGGUAGGGAGACACUUGUGCAUCCCUGCCGAACAACGAACCCCGGCGCGGUCYGCGCCAAGGAACUUUAACGAAAGAGUGCCUCCGGCCGCCUCGGAAACGGUGUGCGUGCGGGAGGUGAAUCUUGUCUAGAACCAU

............((((((.(((...)))....)))))).......((((((((.((((((....)))))).))))))))..(((((((((..((....))..))))))))).............((((((...))))))..((...((((....((((..((((((.(((((.(((....)))...))).)).))))))...))))...)))).))..

>AY352335 *Betula* *davurica* internal transcribed spacer 1, partial sequence; 5.8S ribosomal RNA gene, complete sequence; and internal transcribed spacer 2, partial sequence

UCGAAACCUGCCCAGCAGAACGACCCGUGAACCUGUUGAAACAACUGGGGGUGGGGGGCGAUCUCGCCCCUUGCCCCCGAACGGUAGGGAGACACUUGUGCAUCCCUGCCGAACAACGAACCCCGGCGCGGUCUGCGCCAAGGAACUUUAACGAAAGAGUGCCUCCCGCCGCCUCGGAAACGGUGUGCGUGCGGGAGGUGAAUCUUGUCUAGAACCAU

............((((((.(((...)))....)))))).......(((((((((((((((....)))))))))))))))..(((((((((..((....))..))))))))).............((((((...))))))..((...((((....((((..((((((.(((((.(((....)))...))).)).))))))...))))...)))).))..

>AY352334 *Betula* *uber* internal transcribed spacer 1, partial sequence; 5.8S ribosomal RNA gene, complete sequence; and internal transcribed spacer 2, partial sequence

UCGAAACCUGCCCAGCAGAACGACCCGUGAACAUGUUGAAACAACUGGGGGCGGGGGGCGAUCUCGCCCCGUGCCCCCGAACGGCAGGGAGACACUCGUGCAUCCCUGCCGAACAACGAACCCCGGCGCGGUCUGCGCCAAGGAACUUUAACGAAAGAGUGCCUCCGGCCGUCUCGGAAACGGUGUGCGUGCGGGAGGCGAAUCUUGUCUAGAACCAU

............(((((..(((...))).....))))).......((((((((.((((((....)))))).))))))))..(((((((((..((....))..))))))))).............((((((...))))))..((...((((....((((..((((((.(((((.(((....)))...))).)).))))))...))))...)))).))..

>AY352333 *Betula* *populifolia* internal transcribed spacer 1, partial sequence; 5.8S ribosomal RNA gene, complete sequence; and internal transcribed spacer 2, partial sequence

UCGAAACCUGCCCAGCAGAACGACCCGUGAACCUGUUGAAACAACUGGGGGNGGGGGGCGAUCUCGCCCCUUGUCCCCGAACGGUAGGGAGACACUUGUGCAUCCCUGCCGAACAACGAACCCCGGCGCGGUCCGCGCCAAGGAACUUUAACGAAAGAGUGCCUCCGGCCGCCUCGGAAACGGUGUGCGUGCGGGAGGUGAAUCUUGUCUAGAACCAU

............((((((.(((...)))....)))))).......((((((.((((((((....)))))))).))))))..(((((((((..((....))..))))))))).............((((((...))))))..((...((((....((((..((((((.(((((.(((....)))...))).)).))))))...))))...)))).))..

>AY352332 *Betula* *pendula* internal transcribed spacer 1, partial sequence; 5.8S ribosomal RNA gene, complete sequence; and internal transcribed spacer 2, partial sequence

UCGAAACCUGCCCAGCAGAACGACCCGUGAACCUGUUGAAACAACUGGGGGUGUGGGGCGAUCUCGCCCCUUGCCCCCGAACGGUAGGGAGACACUUGUGCAUCCCUGCCGAACAACGAACCCCGGCGCGGUCCGCGCCAAGGAACUUUAACGAAAGAGUGCCUCCGGCCGCCUCGGAAACGGUGUGCGUGCGGGAGGUGAAUCUUGUCUAGAACCAU

............((((((.(((...)))....)))))).......((((((((.((((((....)))))).))))))))..(((((((((..((....))..))))))))).............((((((...))))))..((...((((....((((..((((((.(((((.(((....)))...))).)).))))))...))))...)))).))..

>AY352331 *Betula* *nigra* internal transcribed spacer 1, partial sequence; 5.8S ribosomal RNA gene, complete sequence; and internal transcribed spacer 2, partial sequence

UCGAAACCUGCCCAGCAGAACGACCCGUGAACCUGUUGAAACAACUGGGGGUGGGGGGCGAUCUCGCCCCGUGCCUCCGAACGGUAGGGAGACACUUGUGCAUCCCUGCUGAACAACGAACCCCGGCGCGGUCUGCGCCAAGGAACUUUAACGAAAGAGUGCCUCCGGCCGCCUCGGAAACGGUGUGCGUGCAGGAAAACAAUCUUGUCUAGAACCAU

............((((((.(((...)))....)))))).......((((((((.((((((....)))))).))))))))..(((((((((..((....))..))))))))).............((((((...))))))..((...((((....((((.((..(((.(((((.(((....)))...))).)).)))...)).))))...)))).))..

>AY352330 *Betula* *lenta* internal transcribed spacer 1, partial sequence; 5.8S ribosomal RNA gene, complete sequence; and internal transcribed spacer 2, partial sequence

UCGAAACCUGCCCAGCAGAACGACCCGUGAACAUGUUGAAACAACUGGGGGCGGGGGGCGAUCUCGCCCCGUGCCCCCGAACGGCAGGGAGACACUCGUGCAUCCCUGCCGAACAACGAACCCCGGCGCGGUCUGCGCCAAGGAACUUUAACGAAAGAGUGCCUCCGGCCGUCUCGGAAACGGUGUGCGUGCGGGAGGCGAAUCUUGUCUAGAACCAU

............(((((..(((...))).....))))).......((((((((.((((((....)))))).))))))))..(((((((((..((....))..))))))))).............((((((...))))))..((...((((....((((..((((((.(((((.(((....)))...))).)).))))))...))))...)))).))..

>AF432067 *Betula* *papyrifera* internal transcribed spacer 1, partial sequence; 5.8S ribosomal RNA gene, complete sequence; and internal transcribed spacer 2, partial sequence

UCGAAACCUGCCCAGCAGAACGACCCGUGAACCUGUUGAAACAACUGGGGGUGGGGGGCGAUCUCGCCCCUUGCCCCCGAACGGUAGGGAGACACUUGUGCAUCCCUGCCGAACAACGAACCCCGGCGCGGUCUGCGCCAAGGAACUUUAACGAAAGAGUGCCUCCCGCCGCCUCGGAAACGGUGUGCGUGCGGGAGGUGAAUCUUGUCUAGAACCAU

............((((((.(((...)))....)))))).......(((((((((((((((....)))))))))))))))..(((((((((..((....))..))))))))).............((((((...))))))..((...((((....((((..((((((.(((((.(((....)))...))).)).))))))...))))...)))).))..

>DQ397523 *Betula* *occidentalis* internal transcribed spacer 1, partial sequence; 5.8S ribosomal RNA gene, complete sequence; and internal transcribed spacer 2, partial sequence

UCGAAACCUGCCCAGCAGAACGACCCGUGAACCUGUUGAAACAACUGGGGGCGUGGGGCGAUCUCGCCCCUUGCCCCCGAACGGUAGGGAGACACUUGUGCAUCCCUGCCGAACAACGAACCCCGGCGCGGUCUGCGCCAAGGAACUUUAACGAAAGAGUGCCUCCGGCCGCCUCGGAAACGGUGUGCGUGCGGGAGGUGAAUCUUGUCUAGAACCAU

............((((((.(((...)))....)))))).......((((((((.((((((....)))))).))))))))..(((((((((..((....))..))))))))).............((((((...))))))..((...((((....((((..((((((.(((((.(((....)))...))).)).))))))...))))...)))).))..

>MH014808|*Betula*_*borysthenica* KW0064215 type

UCGAAACCUGCCCAGCAGAACGACCCGUGAACCUGUUGAAACAACUGGGGGUGGGGGGCGAUCUCGCCCCUUGCCCCCGAACGGUAGGGAGACACUUGUGCAUCCCUGCCGAACAACGAACCCCGGCGCGGUCCGCGCCAAGGAACUUUAACGAAAGAGUGCCUCCGGCCGCCUCGGAAACGGUGUGCGUGCGGGAGGUGAAUCUUGUCUAGAACCAU

............((((((.(((...)))....)))))).......(((((((((((((((....)))))))))))))))..(((((((((..((....))..))))))))).............((((((...))))))..((...((((....((((..((((((.(((((.(((....)))...))).)).))))))...))))...)))).))..

>MH238480|*Betula* *klokovii* KW006422 type

UCGAAACCUGCCCAGCAGAACGACCCGUGAACCUGUUGAAACAACUGGGGGUGGGGGGCGAUCUCGCCCCUUGCCCCCGAACGGUAGGGAGACACUUGUGCAUCCCUGCCGAACAACGAACCCCGGCGCGGUCCGCGCCAAGGAACUUUAACGAAAGAGUGCCUCCGGCCGCCUCGGAAACGGUGUGCGUGCGGGAGGUGAAUCU-------------

............((((((.(((...)))....)))))).......(((((((((((((((....)))))))))))))))..(((((((((..((....))..))))))))).............((((((...))))))..--------------(((..((((((.(((((.(((....)))...))).)).))))))...)))-------------

>MH014819|*Betula* *klokovii* KW006422 type

UCGAAACCUGCCCAGCAGAACGACCCGUGAACCUGUUGAAACAACUGGGGGUGGGGGGCGAUCUCGCCCCUUGCCCCCGAACGGUAGGGAGACACUUGUGCAUCCCUGCCGAACAACGAACCCCGGCGCGGUCCGCGCCAAGGAACUUUAACGAAAGAGUGCCUCCGGCCGCCUCGGAAACGGUGUGCGUGCGGGAGGUGAAUCU-------------

............((((((.(((...)))....)))))).......(((((((((((((((....)))))))))))))))..(((((((((..((....))..))))))))).............((((((...))))))..--------------(((..((((((.(((((.(((....)))...))).)).))))))...)))-------------

>MH231207|*Betula* *kotulae* KW006422 type

UCGAAACCUGCCCAGCAGAACGACCCGUGAACCUGUUGAAACAACUGGGGGUGUGGGGCGAUCUCGCCCCUUGCCCCCGAACGGUAGGGAGACACUUGUGCAUCCCUGCCGAACAACGAACCCCGGCGCGGUCCGCGCCAAGGAACUUUAACGAAAGAGUGCCUCCGGCCGCCUCGGAAACGGUGUGCGUGCGGGAGGUGAAUCUUGUCUAGAACCAU

............((((((.(((...)))....)))))).......((((((((.((((((....)))))).))))))))..(((((((((..((....))..))))))))).............((((((...))))))..((...((((....((((..((((((.(((((.(((....)))...))).)).))))))...))))...)))).))..

>MH178103|*Betula* *kotulae* KW008349

UCGAAACCUGCCCAGCAGAACGACCCGUGAACCUGUUGAAACAACUGGGGGUGUGGGGCGAUCUCGCCCCUUGCCCCCGAACGGUAGGGAGACACUUGUGCAUCCCUGCCGAACAACGAACCCCGGCGCGGUCCGCGCCAAGGAACUUUAACGAAAGAGUGCCUCCGGCCGCCUCGGAAACGGUGUGCGUGCGGGAGGUGAAUCUUGUCUAGAACCAU

............((((((.(((...)))....)))))).......((((((((.((((((....)))))).))))))))..(((((((((..((....))..))))))))).............((((((...))))))..((...((((....((((..((((((.(((((.(((....)))...))).)).))))))...))))...)))).))..

>MH178104|*Betula* *kotulae* KW06427

UCGAAACCUGCCCAGCAGAACGACCCGUGAACCUGUUGAAACAACUGGGGGUGUGGGGCGAUCUCGCCCCUUGCCCCCGAACGGUAGGGAGACACUUGUGCAUCCCUGCCGAACAACGAACCCCGGCGCGGUCCGCGCCAAGGAACUUUAACGAAAGAGUGCCUCCGGCCGCCUCGGAAACGGUGUGCGUGCGGGAGGUGAAUCUUGUCUAGAACCAU

............((((((.(((...)))....)))))).......((((((((.((((((....)))))).))))))))..(((((((((..((....))..))))))))).............((((((...))))))..((...((((....((((..((((((.(((((.(((....)))...))).)).))))))...))))...)))).))..

>MH178105|*Betula* *kotulae* KW128013

UCGAAACCUGCCCAGCAGAACGACCCGUGAACCUGUUGAAACAACUGGGGGUGUGGGGCGAUCUCGCCCCUUGCCCCCGAACGGUAGGGAGACACUUGUGCAUCCCUGCCGAACAACGAACCCCGGCGCGGUCCGCGCCAAGGAACUUUAACGAAAGAGUGCCUCCGGCCGCCUCGGAAACGGUGUGCGUGCGGGAGGUGAAUCUUGUCUAGAACCAU

............((((((.(((...)))....)))))).......((((((((.((((((....)))))).))))))))..(((((((((..((....))..))))))))).............((((((...))))))..((...((((....((((..((((((.(((((.(((....)))...))).)).))))))...))))...)))).))..

>MH178106|*Betula* *kotulae* KW128014

UCGAAACCUGCCCAGCAGAACGACCCGUGAACCUGUUGAAACAACUGGGGGUGUGGGGCGAUCUCGCCCCUUGCCCCCGAACGGUAGGGAGACACUUGUGCAUCCCUGCCGAACAACGAACCCCGGCGCGGUCCGCGCCAAGGAACUUUAACGAAAGAGUGCCUCCGGCCGCCUCGGAAACGGUGUGCGUGCGGGAGGUGAAUCUUGUCUAGAACCAU

............((((((.(((...)))....)))))).......((((((((.((((((....)))))).))))))))..(((((((((..((....))..))))))))).............((((((...))))))..((...((((....((((..((((((.(((((.(((....)))...))).)).))))))...))))...)))).))..

>MH178107|*Betula* *kotulae* KW128016

UCGAAACCUGCCCAGCAGAACGACCCGUGAACCUGUUGAAACAACUGGGGGUGUGGGGCGAUCUCGCCCCUUGCCCCCGAACGGUAGGGAGACACUUGUGCAUCCCUGCCGAACAACGAACCCCGGCGCGGUCCGCGCCAAGGAACUUUAACGAAAGAGUGCCUCCGGCCGCCUCGGAAACGGUGUGCGUGCGGGAGGUGAAUCUUGUCUAGAACCAU

............((((((.(((...)))....)))))).......((((((((.((((((....)))))).))))))))..(((((((((..((....))..))))))))).............((((((...))))))..((...((((....((((..((((((.(((((.(((....)))...))).)).))))))...))))...)))).))..

>MH178108|*Betula* *kotulae* KW128018

UCGAAACCUGCCCAGCAGAACGACCCGUGAACCUGUUGAAACAACUGGGGGUGUGGGGCGAUCUCGCCCCUUGCCCCCGAACGGUAGGGAGACACUUGUGCAUCCCUGCCGAACAACGAACCCCGGCGCGGUCCGCGCCAAGGAACUUUAACGAAAGAGUGCCUCCGGCCGCCUCGGAAACGGUGUGCGUGCGGGAGGUGAAUCUUGUCUAGAACCAU

............((((((.(((...)))....)))))).......((((((((.((((((....)))))).))))))))..(((((((((..((....))..))))))))).............((((((...))))))..((...((((....((((..((((((.(((((.(((....)))...))).)).))))))...))))...)))).))..

>MH178109|*Betula* *kotulae* KW128022

UCGAAACCUGCCCAGCAGAACGACCCGUGAACCUGUUGAAACAACUGGGGGUGUGGGGCGAUCUCGCCCCUUGCCCCCGAACGGUAGGGAGACACUUGUGCAUCCCUGCCGAACAACGAACCCCGGCGCGGUCCGCGCCAAGGAACUUUAACGAAAGAGUGCCUCCGGCCGCCUCGGAAACGGUGUGCGUGCGGGAGGUGAAUCUUGUCUAGAACCAU

............((((((.(((...)))....)))))).......((((((((.((((((....)))))).))))))))..(((((((((..((....))..))))))))).............((((((...))))))..((...((((....((((..((((((.(((((.(((....)))...))).)).))))))...))))...)))).))..

>MH231208|*Betula* *kotulae* KW000128019

UCGAAACCUGCCCAGCAGAACGACCCGUGAACCUGUUGAAACAACUGGGGGUGUGGGGCGAUCUCGCCCCUUGCCCCCGAACGGUAGGGAGACACUUGUGCAUCCCUGCCGAACAACGAACCCCGGCGCGGUCCGCGCCAAGGAACUUUAACGAAAGAGUGCCUCCGGCCGCCUCGGAAACGGUGUGCGUGCGGGAGGUGAAUCUUGUCUAGAACCAU

............((((((.(((...)))....)))))).......((((((((.((((((....)))))).))))))))..(((((((((..((....))..))))))))).............((((((...))))))..((...((((....((((..((((((.(((((.(((....)))...))).)).))))))...))))...)))).))..

>MH231209|*Betula* *kotulae* KW000128020

UCGAAACCUGCCCAGCAGAACGACCCGUGAACCUGUUGAAACAACUGGGGGUGUGGGGCGAUCUCGCCCCUUGCCCCCGAACGGUAGGGAGACACUUGUGCAUCCCUGCCGAACAACGAACCCCGGCGCGGUCCGCGCCAAGGAACUUUAACGAAAGAGUGCCUCCGGCCGCCUCGGAAACGGUGUGCGUGCGGGAGGUGAAUCUUGUCUAGAACCAU

............((((((.(((...)))....)))))).......((((((((.((((((....)))))).))))))))..(((((((((..((....))..))))))))).............((((((...))))))..((...((((....((((..((((((.(((((.(((....)))...))).)).))))))...))))...)))).))..

>MH231210|*Betula* *kotulae* KW000128023

UCGAAACCUGCCCAGCAGAACGACCCGUGAACCUGUUGAAACAACUGGGGGUGUGGGGCGAUCUCGCCCCUUGCCCCCGAACGGUAGGGAGACACUUGUGCAUCCCUGCCGAACAACGAACCCCGGCGCGGUCCGCGCCAAGGAACUUUAACGAAAGAGUGCCUCCGGCCGCCUCGGAAACGGUGUGCGUGCGGGAGGUGAAUCUUGUCUAGAACCAU

............((((((.(((...)))....)))))).......((((((((.((((((....)))))).))))))))..(((((((((..((....))..))))))))).............((((((...))))))..((...((((....((((..((((((.(((((.(((....)))...))).)).))))))...))))...)))).))..

>MH231213|*Betula* *kotulae* LW006898

UCGAAACCUGCCCAGCAGAACGACCCGUGAACCUGUUGAAACAACUGGGGGUGUGGGGCGAUCUCGCCCCUUGCCCCCGAACGGUAGGGAGACACUUGUGCAUCCCUGCCGAACAACGAACCCCGGCGCGGUCCGCGCCAAGGAACUUUAACGAAAGAGUGCCUCCGGCCGCCUCGGAAACGGUGUGCGUGCGGGAGGUGAAUCUUGUCUAGAACCAU

............((((((.(((...)))....)))))).......((((((((.((((((....)))))).))))))))..(((((((((..((....))..))))))))).............((((((...))))))..((...((((....((((..((((((.(((((.(((....)))...))).)).))))))...))))...)))).))..

>MH231214|*Betula* *kotulae* (live)

UCGAAACCUGCCCAGCAGAACGACCCGUGAACCUGUUGAAACAACUGGGGGUGUGGGGCGAUCUCGCCCCUUGCCCCCGAACGGUAGGGAGACACUUGUGCAUCCCUGCCGAACAACGAACCCCGGCGCGGUCCGCGCCAAGGAACUUUAACGAAAGAGUGCCUCCGGCCGCCUCGGAAACGGUGUGCGUGCGGGAGGUGAAUCUUGUCUAGAACCAU

............((((((.(((...)))....)))))).......((((((((.((((((....)))))).))))))))..(((((((((..((....))..))))))))).............((((((...))))))..((...((((....((((..((((((.(((((.(((....)))...))).)).))))))...))))...)))).))..

>MH238474|*Betula* *kotulae* 031472

UCGAAACCUGCCCAGCAGAACGACCCGUGAACCUGUUGAAACAACUGGGGGUGUGGGGCGAUCUCGCCCCUUGCCCCCGAACGGUAGGGAGACACUUGUGCAUCCCUGCCGAACAACGAACCCCGGCGCGGUCCGCGCCAAGGAACUUUAACGAAAGAGUGCCUCCGGCCGCCUCGGAAACGGUGUGCGUGCGGGAGGUGAAUCU-------------

............((((((.(((...)))....)))))).......((((((((.((((((....)))))).))))))))..(((((((((..((....))..))))))))).............((((((...))))))..--------------(((..((((((.(((((.(((....)))...))).)).))))))...)))-------------

>MH238475|*Betula* *kotulae* LW032758

UCGAAACCUGCCCAGCAGAACGACCCGUGAACCUGUUGAAACAACUGGGGGUGUGGGGCGAUCUCGCCCCUUGCCCCCGAACGGUAGGGAGACACUUGUGCAUCCCUGCCGAACAACGAACCCCGGCGCGGUCCGCGCCAAGGAACUUUAACGAAAGAGUGCCUCCGGCCGCCUCGGAAACGGUGUGCGUGCGGGAGG--------------------

............((((((.(((...)))....)))))).......((((((((.((((((....)))))).))))))))..(((((((((..((....))..))))))))).............((((((...))))))..---------------------((((.(((((.(((....)))...))).)).)))))--------------------

>MH238476|*Betula* *kotulae* LW032763

UCGAAACCUGCCCAGCAGAACGACCCGUGAACCUGUUGAAACAACUGGGGGUGGGGGGCGAUCUCGCCCCUUGCCCCCGAACGGUAGGGAGACACUUGUGCAUCCCUGCCGAACAACGAACCCCGGCGCGGUCCGCGCCAAGGAACUUUAACGAAAGAGUGCCUCCGGCCGCCUCGGAAACGGUGUGCGUGCGGGAGGUGAAUCU-------------

............((((((.(((...)))....)))))).......((((((((.((((((....)))))).))))))))..(((((((((..((....))..))))))))).............((((((...))))))..--------------(((..((((((.(((((.(((....)))...))).)).))))))...)))-------------

MH238477|*Betula* *kotulae* LWS27026

UCGAAACCUGCCCAGCAGAACGACCCGUGAACCUGUUGAAACAACUGGGGGUGUGGGGCGAUCUCGCCCCUUGCCCCCGAACGGUAGGGAGACACUUGUGCAUCCCUGCCGAACAACGAACCCCGGCGCGGUCCGCGCCAAGGAACUUUAACGAAAGAGUGCCUCCGGCCGCCUCGGAAACGGUGUGCGUGCGGGAGGUGAAUCU-------------

............((((((.(((...)))....)))))).......((((((((.((((((....)))))).))))))))..(((((((((..((....))..))))))))).............((((((...))))))..--------------(((..((((((.(((((.(((....)))...))).)).))))))...)))-------------

>MH238478|*Betula* *kotulae* LWS27022

UCGAAACCUGCCCAGCAGAACGACCCGUGAACCUGUUGAAACAACUGGGGGUGUGGGGCGAUCUCGCCCCUUGCCCCCGAACGGUAGGGAGACACUUGUGCAUCCCUGCCGAACAACGAACCCCGGCGCGGUCCGCGCCAAGGAACUUUAACGAAAGAGUGCCUCCGGCCGCCUCGGAAACGGUGUGCGUGCGGGAGGUGAAUCUUGUCUAGA-----

............((((((.(((...)))....)))))).......((((((((.((((((....)))))).))))))))..(((((((((..((....))..))))))))).............((((((...))))))..-----((((....((((..((((((.(((((.(((....)))...))).)).))))))...))))...))))-----

>MH238476|*Betula* *oycowiensis*

UCGAAACCUGCCCAGCAGAACGACCCGUGAACCUGUUGAAACAACUGGGGGUGGGGGGCGAUCUCGCCCCUUGCCCCCGAACGGUAGGGAGACACUUGUGCAUCCCUGCCGAACAACGAACCCCGGCGCGGUCCGCGCCAAGGAACUUUAACGAAAGAGUGCCUCCGGCCGCCUCGGAAACGGUGUGCGUGCGGGAGGUGAAUCU-------------

............((((((.(((...)))....)))))).......(((((((((((((((....)))))))))))))))..(((((((((..((....))..))))))))).............((((((...))))))..--------------(((..((((((.(((((.(((....)))...))).)).))))))...)))-------------

>MH014809|*Betula* *pubescens* ssp. *carpatica*

UCGAAACCUGCCCAGCAGAACGACCCGUGAACCUGUUGAAACAACUGGGGGUGUGGGGCGAUCUCGCCCCUUGCCCCCGAACGGUAGGGAGACACUUGUGCAUCCCUGCCGAACAACGAACCCCGGCGCGGUCCGCGCCAAGGAACUUUAACGAAAGAGUGCCUCCGGCCGCCUCGGAAACGGUGUGCGUGCGGGAGGUGAAUCUUGUCUAGAACCAU

............((((((.(((...)))....)))))).......((((((((.((((((....)))))).))))))))..(((((((((..((....))..))))))))).............((((((...))))))..((...((((....((((..((((((.(((((.(((....)))...))).)).))))))...))))...)))).))..

>MH178101|*Betula* *pubescens* var. *sibakademica* LE01041130(R) type

UCGAAACCUGCCCAGCAGAACGACCCGUGAACCUGUUGAAACAACUGGGGGUGGGGGGCGAUCUCGCCCCUUGCCCCCGAACGGUAGGGAGACACUUGUGCAUCCCUGCCGAACAACGAACCCCGGCGCGGUCCGCGCCAAGGAACUUUAACGAAAGAGUGCCUCCGGCCGCCUCGGAAACGGUGUGCGUGCGGGAGGUGAAUCUUGUCUAGAACCAU

............((((((.(((...)))....)))))).......(((((((((((((((....)))))))))))))))..(((((((((..((....))..))))))))).............((((((...))))))..((...((((....((((..((((((.(((((.(((....)))...))).)).))))))...))))...)))).))..

>MH231206|*Betula* *pubescens* var. *sibakademica* LE01041130(L) type

UCGAAACCUGCCCAGCAGAACGACCCGUGAACCUGUUGAAACAACUGGGGGUGGGGGGCGAUCUCGCCCCUUGCCCCCGAACGGUAGGGAGACACUUGUGCAUCCCUGCCGAACAACGAACCCCGGCGCGGUCCGCGCCAAGGAACUUUAACGAAAGAGUGCCUCCGGCCGCCUCGGAAACGGUGUGCGUGCGGGAGGUGAAUCUUGUCUA-------

............((((((.(((...)))....)))))).......(((((((((((((((....)))))))))))))))..(((((((((..((....))..))))))))).............((((((...))))))..-------((....((((..((((((.(((((.(((....)))...))).)).))))))...))))...))-------

>MH178102|*Betula* *pubescens* var. *sibakademica* KW128024

UCGAAACCUGCCCAGCAGAACGACCCGUGAACCUGUUGAAACAACUGGGGGUGGGGGGCGAUCUCGCCCCUUGCCCCCGAACGGUAGGGAGACACUUGUGCAUCCCUGCCGAACAACGAACCCCGGCGCGGUCCGCGCCAAGGAACUUUAACGAAAGAGUGCCUCCGGCCGCCUCGGAAACGGUGUGCGUGCGGGAGGUGAAUCUUGUCUAGAACCAU

............((((((.(((...)))....)))))).......(((((((((((((((....)))))))))))))))..(((((((((..((....))..))))))))).............((((((...))))))..((...((((....((((..((((((.(((((.(((....)))...))).)).))))))...))))...)))).))..

>MH231212|*Betula* *pubescens* var. *sibakademica* LWKS031322

UCGAAACCUGCCCAGCAGAACGACCCGUGAACCUGUUGAAACAACUGGGGGUGGGGGGCGAUCUCGCCCCUUGCCCCCGAACGGUAGGGAGACACUUGUGCAUCCCUGCCGAACAACGAACCCCGGCGCGGUCCGCGCCAAGGAACUUUAACGAAAGAGUGCCUCCGGCCGCCUCGGAAACGGUGUGCGUGCGGGAGGUGAAUCUUGUCUAGAACCAU

............((((((.(((...)))....)))))).......(((((((((((((((....)))))))))))))))..(((((((((..((....))..))))))))).............((((((...))))))..((...((((....((((..((((((.(((((.(((....)))...))).)).))))))...))))...)))).))..

>MH238471|*Betula* *pubescens* var. sibacademica KW000128012

UCGAAACCUGCCCAGCAGAACGACCCGUGAACCUGUUGAAACAACUGGGGGUGGGGGGCGAUCUCGCCCCUUGCCCCCGAACGGUAGGGAGACACUUGUGCAUCCCUGCCGAACAACGAACCCCGGCGCGGUCCGCGCCAAGGAACUUUAACGAAAGAGUGCCUCCGGCCGCCUCGGAAACGGUGUGCGUGCGGGAGGUGAAUCUUGUCUAG------

............((((((.(((...)))....)))))).......(((((((((((((((....)))))))))))))))..(((((((((..((....))..))))))))).............((((((...))))))..------(((....((((..((((((.(((((.(((....)))...))).)).))))))...))))...)))------

>MH238472|*Betula* *pubescens* var. sibacademica 031473(1)

UCGAAACCUGCCCAGCAGAACGACCCGUGAACCUGUUGAAACAACUGGGGGUGGGGGGCGAUCUCGCCCCUUGCCCCCGAACGGUAGGGAGACACUUGUGCAUCCCUGCCGAACAACGAACCCCGGCGCGGUCCGCGCCAAGGAACUUUAACGAAAGAGUGCCUCCGGCCGCCUCGGAAACGGUGUGCGUGCGGGAGGUGAAUCUUGUCUAG------

............((((((.(((...)))....)))))).......(((((((((((((((....)))))))))))))))..(((((((((..((....))..))))))))).............((((((...))))))..------(((....((((..((((((.(((((.(((....)))...))).)).))))))...))))...)))------

>MH238473|*Betula* *pubescens* var. sibacademica 031473(2)

UCGAAACCUGCCCAGCAGAACGACCCGUGAACCUGUUGAAACAACUGGGGGUGGGGGGCGAUCUCGCCCCUUGCCCCCGAACGGUAGGGAGACACUUGUGCAUCCCUGCCGAACAACGAACCCCGGCGCGGUCCGCGCCAAGGAACUUUAACGAAAGAGUGCCUCCGGCCGCCUCGGAAACGGUGUGCGUGCGGGAGGUGAAUCU-------------

............((((((.(((...)))....)))))).......(((((((((((((((....)))))))))))))))..(((((((((..((....))..))))))))).............((((((...))))))..--------------(((..((((((.(((((.(((....)))...))).)).))))))...)))-------------
