# Supplementary material for: ITS secondary structure reconstruction to resolve taxonomy and phylogeny of the Betula L. genus
Source: PeerJ. 2021 Mar 23;9:e10889. doi: 10.7717/peerj.10889 (PMC7996101; doi:10.7717/peerj.10889)
Supplement: Supplemental Information 3 [file peerj-09-10889-s003.docx]

**Data S3**. ITS2 structural alignment.

>AJ006445 *Betula* *pendula* 18S, 5.8S and 28S rRNA genes and internal transcribed spacers (ITS1 and ITS2)

ACGUCUGCCUGGGUGUCACGCAUCGUUGCCCCCAACCCCAUCUCCUUGCAAAGGGACGAGGGGGCCUGUGGGGCAGAAAUUGGCCUCCCGUGAGCUCAUGCAUGCGGUUGGCCUAAAAGCGAGUCCUCGGCGACGCGCGCCACGACAAUCGGUGGUUGUCAAACCCUCGUGUCCCGUCGUGCGUGCCGCGUCGCUCAUCGUGUGCUCCUUGACCCUGUUGUGUCGCGCUAGCGACGCUUCCAACGCGA

...............((.((.....(((((((((.((((.(((((((....))))).)).))))..)).))))))).....((((..(((((.((....)).)))))..)))).....((((......((((((((((((((((((...(((.((((....)))).)))......)))))).))).)))))))))..))))..................(((((((....)))))))......)).))

>AJ783646 *Betula* *nigra* 18S rRNA gene (partial), 5.8S rRNA gene, 26S rRNA gene (partial), ITS1 and ITS2

ACGUCUGCCUGGGUGUCACGCAUCGUUGCCCCCAACCCCAUCUCCUUGCAAAGGGACGAGAGGGCCAGUGGGGUAGAAAUUGGCCUCCCGUGAGCUCAUGCAUGCGGUUGGCCUAAAAGCGAGUCCUCGGCGACGCGCGCCACGACAAUCGGUGGUUGACAAACCCUCGUGUCCCGUCGUGCGUGCCGCGUUGCUCAUCGUGUGCUCUUUGACCCUGUUGUGUCGCGCUAGCGAUGCUUCCAAUGCGA

...............((.((.....((((((((..(((..(((((((....))))).))..)))...).))))))).....((((..(((((.((....)).)))))..)))).....((((......((((((((((((((((((...(((.((((....)))).)))......)))))).))).)))))))))..))))..................(((((((....)))))))......)).))

>AJ783645 *Betula* *insignis* 18S rRNA gene (partial), 5.8S rRNA gene, 26S rRNA gene (partial), ITS1 and ITS2

ACGUCUGCCUGGGUGUCACGCAUCGUUGCCCCCAACCCCAUCUCCUUGCAAAGGGACGAGGGG-CUUGUGGGGCAGAAAUUGGCCUCCCGUGAGCUUACGCAUGCGGUUGGCCUAAAAGCGAGUCCUCGGCGACGCGCGCCACGACAAUCGGUGGUUGACAAACCCUCGUGUCCCGUCGUGCGUGCCGCGUCGCUCAUCGUGUGCUCUUUGACCCUGCUGCGUCGCGCUAGCGACGCUUCCAACGCGA

...............((.((.....(((((((((..(((.(((((((....))))).)).)))-..)).))))))).....((((..(((((.((....)).)))))..)))).....((((......((((((((((((((((((...(((.((((....)))).)))......)))))).))).)))))))))..))))..................(((((((....)))))))......)).))

>AJ783644 *Betula* *populifolia* 18S rRNA gene (partial), 5.8S rRNA gene, 26S rRNA gene (partial), ITS1 and ITS2

ACGUCUGCCUGGGUGUCACGCAUCGUUGCCCCCAACCCCAUCUCCUUGCAAAGGGACGAGGGGGCCUGUGGGGCAGAAAUUGGCCUCCCGUGAGCUCAUGCAUGCGGUUGGCCUAAAAGCGAGUCCUCGGCGACGCGCGCCACGACAAUCGGUGGUUGUCAAACCCUCGUGUCCCGUCGUGCGUGCCGCGUCGCUCAUCGUGUGCUCCUUGACCCUGCUGCGUCGCGCUAGCGACGCUUCCAACGCGA

...............((.((.....(((((((((.((((.(((((((....))))).)).))))..)).))))))).....((((..(((((.((....)).)))))..)))).....((((......((((((((((((((((((...(((.((((....)))).)))......)))))).))).)))))))))..))))..................(((((((....)))))))......)).))

>AJ783643 *Betula* *humilis* 18S rRNA gene (partial), 5.8S rRNA gene, 26S rRNA gene (partial), ITS1 and ITS2

ACGUCUGCCUGGGUGUCACGCAUCGUUGCCCCCAACCCCAUCUCCUUGCAAAGGGACGAGGGGGCCUGUGGGGCAGAAAUUGGCCUCCCGUGAGCUCAUGCAUGCGGUUGGCCUAAAAGCGAGUCCUCGGCGACGCGCGCCACGACAAUCGGUGGUUGUCAAACCCUCGUGUCCCGUCGUGCGUGCCGCGUCGCUCAUCGUGUGCUCCUUGACCCUGCUGUGUCGCGCUAGCGACGCUUCCAACGCGA

...............((.((.....(((((((((.((((.(((((((....))))).)).))))..)).))))))).....((((..(((((.((....)).)))))..)))).....((((......((((((((((((((((((...(((.((((....)))).)))......)))))).))).)))))))))..))))..................(((((((....)))))))......)).))

>AJ783642 *Betula* *pumila* 18S rRNA gene (partial), 5.8S rRNA gene, 26S rRNA gene (partial), ITS1 and ITS2

ACGUCUGCCUGGGUGUCACGCAUCGUUGCCCCCAACCCCAUCUCCUUGMAAAGGGACGAGGGGGCCUGUGGGGCAGAAAUUGGCCUCCCGUGAGCUCAUGCAUGCGGUUGGCCUAAAAGCGAGUCCUCGGCGACGCGCGCCACGACAAUCGGUGGUUGUCAAACCCUCGUGUCCCGUCGUGCGUGMCGCGUCGCUCAUCGUGUGCUCCUUGACCCUGCUGUGUCGCGCUAGCGACGCUUCCAACGCGA

...............((.((.....(((((((((.((((.(((((((....))))).)).))))..)).))))))).....((((..(((((.((....)).)))))..)))).....((((......((((((((((((((((((...(((.((((....)))).)))......)))))).))).)))))))))..))))..................(((((((....)))))))......)).))

>AJ783641 *Betula* *alnoides* 18S rRNA gene (partial), 5.8S rRNA gene, 26S rRNA gene (partial), ITS1 and ITS2

ACGUCUGCCUGGGUGUCACGCAUCGUUGCCCCCAACCCCAUCUCCUUGYAAAGGGACGAGGGGGCCUGUGGGGCAGAAAUUGGCCUCCCGUGAGCUCAUGCAUGCGGUUGGCCUAAAAGCGAGUCCUCGGCGACGCGCGCCACGACAAUCGGUGGUUGUCAAACCCUCGUGUCCCGUCGUGCGUGMCGCGUCGCUCAUCGUGUGCUCCUUGACCCUGCUGUGUCGCGCUAGCGACGCUUCCAACGCGA

...............((.((.....(((((((((.((((.(((((((....))))).)).))))..)).))))))).....((((..(((((.((....)).)))))..)))).....((((......((((((((((((((((((...(((.((((....)))).)))......)))))).))).)))))))))..))))..................(((((((....)))))))......)).))

>AJ251683 *Betula* *alba* 18S rRNA gene, 5.8S rRNA gene, 25S rRNA gene, internal transcribed spacer 1 (ITS1) and internal transcribed spacer 2 (ITS2)

ACGUCUGCCUGGGUGUCACGCAUCGUUGCCCCCAACCCCAUCUCCUUGCAAAGGGACGAGGGGGCCUGUGGGGCAGAAAUUGGCCUCCCGUGAGCUCAUGCAUGCGGUUGGCCUAAAAGCGAGUCCUCGGCGACGCGCGCCACGACAAUCGGUGGUUGUCAAACCCUCGUGUCCCGUCGUGCGUGCCGCGUCGCUCAUCGUGUGCUCCUUGACCCUGCUGUGUCGCGCUAGCGACGCUUCCAACGCGA

...............((.((.....(((((((((.((((.(((((((....))))).)).))))..)).))))))).....((((..(((((.((....)).)))))..)))).....((((......((((((((((((((((((...(((.((((....)))).)))......)))))).))).)))))))))..))))..................(((((((....)))))))......)).))

>AM503889.2| *Betula* *pendula* ITS1 (partial), 5.8S rRNA gene, ITS2 and 28S rRNA gene (partial)

ACGUCUGCCUGGGUGUCACGCAUCGUUGCCCCCAACCCCAUCUCCUUGCAAAGGGACGAGGGGGCCUGUGGGGCAGAAAUUGGCCUCCCGUGAGCUCAUGCAUGCGGUUGGCCUAAAAGCGAGUCCUCGGCGACGCGCGCCACGACAAUCGGUGGUUGUCAAACCCUCGUGUCCCGUCGUGCGUGCCGCGUCGCUCAUCGUGUGCUCCUUGACCCUGCUGUGUCGCGCUAGCGACGCUUCCAACGCGA

...............((.((.....(((((((((.((((.(((((((....))))).)).))))..)).))))))).....((((..(((((.((....)).)))))..)))).....((((......((((((((((((((((((...(((.((((....)))).)))......)))))).))).)))))))))..))))..................(((((((....)))))))......)).))

>AB243915 *Betula* *apoiensis* genes for ITS1, 5.8S rRNA, ITS2, haplotype:ap17

ACGUCUGCCUGGGUGUCACGCAUCGUUGCCCCCAACCCCAUCUCCUUGAAAAGGGACGAGGGGGCCUGUGGGGCAGAAAUUGGCCUCCCGUGAGCUCAUGCAUGCGGUUGGUCUAAAAGCGAGUCCUCGGCGACGCGCGCCACGACAAUCGGUGGUUGUCAAACCCUCGUGUCCCGUCGUGCGUGACGCGUCGCUCAUCGUGUGCUCCUUGACCCUGCUGUGUCGUGCUAGCGACGCUUCCAACGCGA

...............((.((.....(((((((((.((((.(((((((....))))).)).))))..)).))))))).....((((..(((((.((....)).)))))..)))).....((((......((((((((((((((((((...(((.((((....)))).)))......)))))).))).)))))))))..))))..................(((((((....)))))))......)).))

>AB243914 *Betula* *apoiensis* genes for ITS1, 5.8S rRNA, ITS2, haplotype:ap14

ACGUCUGCCUGGGUGUCACGCAUCGUUGCCCCCAACCCCAUCUCCUUGAAAAGGGACGAGGGGGCCUGUGGGGCAGAAAUUGGCCUCCCGUGAGCUCAUGCAUGCGGUUGGUCUAAAAGCGAGUCCUCGGCGACGCGCGCCACGACAAUCGGUGGUUGACAAACCCUCGUGUCCCGUCGUGUGUGCCGCGUCGCUCAUCGUGUGCUCUUUGACCCUGCUGUGUUGCGCUAGCGACGCUUCCAACGCGA

...............((.((.....(((((((((.((((.(((((((....))))).)).))))..)).))))))).....((((..(((((.((....)).)))))..)))).....((((......((((((((((((((((((...(((.((((....)))).)))......)))))).))).)))))))))..))))..................(((((((....)))))))......)).))

>AB243913 *Betula* *apoiensis* genes for ITS1, 5.8S rRNA, ITS2, haplotype:ap16

ACGUCUGCCUGGGUGUCACGCAUCGUUGCCCCCAACCC-AUCUCCUUGCAAAGGGACGAGGGGGCCUGUGGGGCAGAAAUUGGCCUCCCGUGAGCUCAUGCAUGCGGUUGGUCUAAAAGCGAGUCCUCGGCGACGCGCGCCACGACAAUCGGUGGUUGUCAAACCCUCGUGUCCCGUCGUGCGUGACGCGUCGCUCAUCGUGUGCUCCUUGACCCUGCUGUGUCGUGCUAGCGACGCUUCCAACGCGA

...............((.((.....(((((((((.(((-.(((((((....))))).))..)))..)).))))))).....((((..(((((.((....)).)))))..)))).....((((......((((((((((((((((((...(((.((((....)))).)))......)))))).))).)))))))))..))))..................(((((((....)))))))......)).))

>AB243912 *Betula* *apoiensis* genes for ITS1, 5.8S rRNA, ITS2, haplotype:ap15

ACGUCUGCCUGGGUGUCACGCAUCGUUGCCCCCAACCCCAUCUCCUUGCAAAGGGACGAGGGGGCCUGUGGGGCAGAAAUUGGCCUCCCGUGAGCUCUUGCAUGCGGUUGGCCUAAAAGCGAGUCCUCGGCGACGCGCGCCACGACAAUCGGUGGUUGACAAACCCUCGUGUCCCGUCGUGCGUGCCGUGUCGCUCAUCGUGUGCUCUUUGACCCUGCUGUGUCGCGCUAGCGACGCUUCCAACGCGA

...............((.((.....(((((((((.((((.(((((((....))))).)).))))..)).))))))).....((((..(((((.((....)).)))))..)))).....((((......((((((((((((((((((...(((.((((....)))).)))......)))))).))).)))))))))..))))..................(((((((....)))))))......)).))

>AB243911 *Betula* *apoiensis* genes for ITS1, 5.8S rRNA, ITS2, haplotype:ap12

ACGUCUGCCUGGGUGUCACGCAUCGUUGCCCCCAACCCCAUCUCCUUGAAAAGGGACGAGGGGGCCUGUGGGGCAGAAAUUGGCCUCCCGUGAGCUCAUGCAUGCGGUUGGUCUAAAAGCGAGUCCUCGGCGACGCGCGCCACGACAAUCGGUGGUUGUCAAACCCUCGUGUCCCGUCGUGCGUGACGCGUCGCUCAUCGUGUGCUCCUUGACCCUGCUGUGUCGUGCUAGCGACGCUUCCAACGCGA

...............((.((.....(((((((((.((((.(((((((....))))).)).))))..)).))))))).....((((..(((((.((....)).)))))..)))).....((((......((((((((((((((((((...(((.((((....)))).)))......)))))).))).)))))))))..))))..................(((((((....)))))))......)).))

>AB243910 *Betula* *apoiensis* genes for ITS1, 5.8S rRNA, ITS2, haplotype:ap10

ACGUCUGCCUGGGUGUCACGCAUCGUUGCCCCCAACCCCAUCUCCUUGCAAAGGGACGAGGGGGCCUGUGGGGCAGAAAUUGGCCUCCCGUGAGCUCAUGCAUGCGGUUGGUCUAAAAGCGAGUCCUCGGCGACGCGCGCCACGACAAUCGGUGGUUGUCAAACCCUCGUGUCCCGUCGUGCGUGACGCGUCGCUCAUCGUGUGCUCCUUGACCCUGCUGUGUCGUGCUAGCGACGCUUCCAACGCGA

...............((.((.....(((((((((.((((.(((((((....))))).)).))))..)).))))))).....((((..(((((.((....)).)))))..)))).....((((......((((((((((((((((((...(((.((((....)))).)))......)))))).))).)))))))))..))))..................(((((((....)))))))......)).))

>AB243909 *Betula* *apoiensis* genes for ITS1, 5.8S rRNA, ITS2, haplotype:ap11

ACGUCUGCCUGGGUGUCACGCAUCGUUGCCCCCAACCCCAUCUCCUUGAAAAGGGACGAGGGGGCCUGUGGGGCAGAAAUUGGCCUCCCGUGAGCUCAUGCAUGCGGUUGGUCUAAAAGCGAGUCCUCGGCGACGCGCGCCACGACAAUCGGUGGUUGUCAAACCCUCGUGUCCCGUCGUGCGUGACGCGUCGCUCAUCGUGUGCUCCUUGACCCUGCUGUGUCGUGCUAGCGACGCUUCCAACGCGA

...............((.((.....(((((((((.((((.(((((((....))))).)).))))..)).))))))).....((((..(((((.((....)).)))))..)))).....((((......((((((((((((((((((...(((.((((....)))).)))......)))))).))).)))))))))..))))..................(((((((....)))))))......)).))

>AB243908 *Betula* *apoiensis* genes for ITS1, 5.8S rRNA, ITS2, haplotype:ap13

ACGUCUGCCUGGGUGUCACGCAUCGUUGCCCCCAACCCCAUCUCCUUGCAAAGGGACGAGGGGGCCUGUGGGGCAGAAAUUGGCCUCCCGUGAGCUCAUGCAUGCGGUUGGCCUAAAAGCGAGUCCUCGGCGACGCGCGCCACGACAAUCGGUGGUUGUCAAACCCUCGUGUCCCGUCGUGCGUGACGCGUCGCUCAUCGUGUGCUCCUUGACCCUGCUGUGUCGUGCUAGCGACGCUUCCAACGCGA

...............((.((.....(((((((((.((((.(((((((....))))).)).))))..)).))))))).....((((..(((((.((....)).)))))..)))).....((((......((((((((((((((((((...(((.((((....)))).)))......)))))).))).)))))))))..))))..................(((((((....)))))))......)).))

>AB243907 *Betula* *apoiensis* genes for ITS1, 5.8S rRNA, ITS2, haplotype:ap5

ACGUCUGCCUGGGUGUCACGCAUCGUUGCCCCCAACCCCAUCUCCUUGCAAAGGGACGAGGGGGCCUGUGGGGCAGAAAUUGGCCUCCCGUGAGCUCAUGCAUGCGGUUGGCCUAAAAGCGAGUCCUCGGCGACGCGCGCCACGACAAUCGGUGGUUGACAAACCCUCGUGUCCCGUCGUGCGUGCCGCGUCGCUCAUCGUGUGCUCUUUGACCCUGCUGUGUCGCGCUAGCGACGCUUCCAACGCGA

...............((.((.....(((((((((.((((.(((((((....))))).)).))))..)).))))))).....((((..(((((.((....)).)))))..)))).....((((......((((((((((((((((((...(((.((((....)))).)))......)))))).))).)))))))))..))))..................(((((((....)))))))......)).))

>AB243906 *Betula* *apoiensis* genes for ITS1, 5.8S rRNA, ITS2, haplotype:ap9

ACGUCUGCCUGGGUGUCACGCAUCGUUGCCCCCAACCCCAUCUCCUUGCAAAGGGACGAGGGGGCCUGUGGGGCAGAAAUUGGCCUCCCGUGAGCUCAUGCAUGCGGUUGGCCUAAAAGCGAGUCCUCGGCGACGCGCGCCACGACAAUCGGUGGUUGACAAACCCUCGUGUCCCGUCGUGCGUGCCGCGUCGCUCAUCGUGUGCUCUUUGACCCUGCUGUGUCGCGCUAGCGACGCUUCCAACGCGA

...............((.((.....(((((((((.((((.(((((((....))))).)).))))..)).))))))).....((((..(((((.((....)).)))))..)))).....((((......((((((((((((((((((...(((.((((....)))).)))......)))))).))).)))))))))..))))..................(((((((....)))))))......)).))

>AB243905 *Betula* *apoiensis* genes for ITS1, 5.8S rRNA, ITS2, haplotype:ap8

ACGUCUGCCUGGGUGUCACGCAUCGUUGCCCCCAACCCCAUCUCCUUGCAAAGGGACGAGGGGGCCUGCGGGGCAGAAAUUGGCCUCCCGUGAGCUCAUGCAUGCGGUUGGCCUAAAAGCGAGUCCUCGGCGACGCGCGCCACGACAAUCGGUGGUUGACAAACCCUCGUGUCCCGUCGUGCGUGCCGCGUCGCUCAUCGUGUGCUCUUUGACCCUGCUGUGUCGCGCUAGCGACGCUUCCAACGCGA

...............((.((.....(((((((((.((((.(((((((....))))).)).))))..)).))))))).....((((..(((((.((....)).)))))..)))).....((((......((((((((((((((((((...(((.((((....)))).)))......)))))).))).)))))))))..))))..................(((((((....)))))))......)).))

>gi|83758349|dbj|AB243904 *Betula* *apoiensis* genes for ITS1, 5.8S rRNA, ITS2, haplotype:ap7

ACGUCUGCCUGGGUGUCACGCAUCGUUGCCCCCAACCCCAUCUCCUUGCAAAGGGACGAGGGGGCCUGUGGGGCAGAAAUUGGCCUCCCGUGAGCUCAUGCAUGCGGUUGGCCUAAAAGCGAGUCCUCGGCGACGCGCGCCACGACAAUCGGUGGUUGACAAACCCUCGUGUCCCGUCGUGCGUGCCGCGUCGCUCAUCGUGUGCUCUUUGACCCUGCUGUGUCGCGCUAGCGACGCUUCCAACGCGA

...............((.((.....(((((((((.((((.(((((((....))))).)).))))..)).))))))).....((((..(((((.((....)).)))))..)))).....((((......((((((((((((((((((...(((.((((....)))).)))......)))))).))).)))))))))..))))..................(((((((....)))))))......)).))

>AB243903 *Betula* *apoiensis* genes for ITS1, 5.8S rRNA, ITS2, haplotype:ap6

ACGUCUGCCUGGGUGUCACGCAUCGUUGCCCCCAACCCCAUCUCCUUGCAAAGGGACGAGGGGGCCUGUGGGGCAGAAAUUGGCCUCCCGUGAGCUCAUGCAUGCGGUUGGCCUAAAAGCGAGUCCUCGGCGACGCGCGCCACGACAAUCGGUGGUUGACAAACCCUCGUGUCCCGUCGUGCGUGUCGCGUCGCUCAUCGUGUGCUCUUUGACCCUGCUGUGUCGCGCUAGCGACGCUUCCAACGCGA

...............((.((.....(((((((((.((((.(((((((....))))).)).))))..)).))))))).....((((..(((((.((....)).)))))..)))).....((((......((((((((((((((((((...(((.((((....)))).)))......)))))).))).)))))))))..))))..................(((((((....)))))))......)).))

>AB243902 *Betula* *apoiensis* genes for ITS1, 5.8S rRNA, ITS2, haplotype:ap3

ACGUCUGCCUGGGUGUCACGCAUCGUUGCCCCCAACCCCAUCUCCUUGCAAAGGGACGAGGGGGCCUGUGGGGCAGAAAUUGGCCUCCCGUGAGCUCAUGCAUGCGGUUGGCCUAAAAGCGAGUCCUCGGCGACGCGCGCCACGACAAUCGGUGGUUGACAAACCCUCGUGUCCCGUCGUGCGUGCCGUGUCGCUCAUCGUGUGCUCUUUGACCCUGCUGUGUCGCGCUAGCGACGCUUCCAUUGCGA

...............((.((.....(((((((((.((((.(((((((....))))).)).))))..)).))))))).....((((..(((((.((....)).)))))..)))).....((((......((((((((((((((((((...(((.((((....)))).)))......)))))).))).)))))))))..))))..................(((((((....)))))))......)).))

>AB243901 *Betula* *apoiensis* genes for ITS1, 5.8S rRNA, ITS2, haplotype:ap2

ACGUCUGCCUGGGUGUCACGCAUCGUUGCCCCCAACCCCAUCUCCUUGCAAAGGGACGAGGGGGCCUGUGGGGCAGAAAUUGGCCUCCCGUGAGCUCUUGCAUGCGGUUGGCCUAAAAACGAGUCCUCGGCGACGCGCGCCACGACAAUCGGUGGUUGACAAACCCUCGUGUCCCGUCGUGCGUGCCGUGUCGCUCAUCGUGUGCUCUUUGACCCUGCUGUGUCGCGCUAGCGACGCUUCCAACGCGA

...............((.((.....(((((((((.((((.(((((((....))))).)).))))..)).))))))).....((((..(((((.((....)).)))))..)))).....((((......((((((((((((((((((...(((.((((....)))).)))......)))))).))).)))))))))..))))..................(((((((....)))))))......)).))

>AB243900 *Betula* *apoiensis* genes for ITS1, 5.8S rRNA, ITS2, haplotype:ap1

ACGUCUGCCUGGGUGUCACGCAUCGUUGCCCCCUACCCCAUCUCCUUGCAAAGGGACGAGGGGGCCUGUGGGGCAGAAAUUGGCCUCCCGUGAGCUCAUGCAUGCGGUUGGCCUAAAAGCGAGUCCUCGGCGACGCGCGCCACGACAAUCGGUGGUUGACAAACCCUCGUGUCCCGUCGUGCGUGCCGUGUCGCUCAUCGUGUGCUCUUUGACCCUGCUGUGUCGCGCUAGCGACGCUUCCAACGCGA

...............((.((.....((((((((..((((.(((((((....))))).)).))))...).))))))).....((((..(((((.((....)).)))))..)))).....((((......((((((((((((((((((...(((.((((....)))).)))......)))))).))).)))))))))..))))..................(((((((....)))))))......)).))

>AB243899 *Betula* *apoiensis* genes for ITS1, 5.8S rRNA, ITS2, haplotype:ap4

ACGUCUGCCUGGGUGUCACGCAUCGUUGCCCCCAACCCCAUCUCCUUGCAAAGGGACGAGGGGGCCUGUGGGGCAGAAAUUGGCCUCCCGUGAGCUCAUGCAUGCGGUUGGCCUAAAAGCGAGUCCUCGGCGACGCGCGCCACGACAAUCGGUGGUUGACAAACCCUCGUGUCCCGUCGUGCGUGCCGUGUCGCUCAUCGUGUGCUCUUUGACCCUGCUGUGUCGCGCUAGCGACGCUUCCAACGCGA

...............((.((.....(((((((((.((((.(((((((....))))).)).))))..)).))))))).....((((..(((((.((....)).)))))..)))).....((((......((((((((((((((((((...(((.((((....)))).)))......)))))).))).)))))))))..))))..................(((((((....)))))))......)).))

>AB243898 *Betula* *ovalifolia* genes for ITS1, 5.8S rRNA, ITS2, haplotype:ov3

ACGUCUGCCUGGGUGUCACGCAUCGUUGCCCCCAACCCCAUCUCCUUGAAAAGGGACGAGGGGGCCUGUGGGGCAGAAAUUGGCCUCCCGUGAGCUCAUGCAUGCGGUUGGCCUAAAAGCGAGUCCUCGGCGACGCGCGCCACGACAAUCGGUGGUUGUCAAACCCUCGUGUCCCGUCGUGCGUGACGCGUCGCUCAUCGUGUGCUCCUUGACCCUGCUGUGUCGCGCUAGCGACGCUUCCAACGCGA

...............((.((.....(((((((((.((((.(((((((....))))).)).))))..)).))))))).....((((..(((((.((....)).)))))..)))).....((((......((((((((((((((((((...(((.((((....)))).)))......)))))).))).)))))))))..))))..................(((((((....)))))))......)).))

>AB243897 *Betula* *ovalifolia* genes for ITS1, 5.8S rRNA, ITS2, haplotype:ov2

ACGUCUGCCUGGGUGUCACGCAUCGUUGCCCCCAACCCCAUCUCCUUGCAAAGGGACGAGGGGGCCUGUGGGGCAGAAAUUGGCCUCCCGUGAGCUCAUGCAUGCGGUUGGCCUAAAAGCGAGUCCUCGGCGACGCGCGCCACGACAAUCGGUGGUUGUCAAACCCUCGUGUCCCGUCGUGCGUGCCGCGUCGCUCAUCGUGUGCUCCUUGACCCUGCUGUGUCGCGCUAGCGACGCUUCCAACGCGA

...............((.((.....(((((((((.((((.(((((((....))))).)).))))..)).))))))).....((((..(((((.((....)).)))))..)))).....((((......((((((((((((((((((...(((.((((....)))).)))......)))))).))).)))))))))..))))..................(((((((....)))))))......)).))

>AB243896 *Betula* *ovalifolia* genes for ITS1, 5.8S rRNA, ITS2, haplotype:ov1

ACGUCUGCCUGGGUGUCACGCAUCGUUGCCCCCAACCCCAUCUCCUUGAAAAGGGACGAGGGGGCCUGUGGGGCAGAAAUUGGCCUCCCGUGAGCUCAUGCAUGCGGUUGGUCUAAAAGCGAGUCCUCGGCGACGCGCGCCACGACAAUCGGUGGUUGUCAAACCCUCGUGUCCCGUCGUGCGUGACGCGUCGCUCAUCGUGUGCUCCUUGACCCUGCUGUGUCGUGCUAGCGACGCUUCCAACGCGA

...............((.((.....(((((((((.((((.(((((((....))))).)).))))..)).))))))).....((((..(((((.((....)).)))))..)))).....((((......((((((((((((((((((...(((.((((....)))).)))......)))))).))).)))))))))..))))..................(((((((....)))))))......)).))

>AB243895 *Betula* *middendorffii* genes for ITS1, 5.8S rRNA, ITS2, haplotype:mi

ACGUCUGCCUGGGUGUCACGCAUCGUUGCCCCCAACCCCAUCUCCUUGAAAAGGGACGAGGGGGCCUGUGGGGCAGAAAUUGGCCUCCCGUGAGCUCAUGCAUGCGGUUGGCCUAAAAGCGAGUCCUCGGCGACGCGCGCCACGACAAUCGGUGGUUGUCAAACCCUCGUGUCCCGUCGUGCGUGACGCGUCGCUCAUCGUGUGCUCCUUGACCCUGCUGUGUCGCGCUAGCGACGCUUCCAACGCGA

...............((.((.....(((((((((.((((.(((((((....))))).)).))))..)).))))))).....((((..(((((.((....)).)))))..)))).....((((......((((((((((((((((((...(((.((((....)))).)))......)))))).))).)))))))))..))))..................(((((((....)))))))......)).))

>AB243894 *Betula* *davurica* genes for ITS1, 5.8S rRNA, ITS2, haplotype:da2

ACGUCUGCCUGGGUGUCACGCAUCGUUGCCCCCAACCCCAUCUCCUUGUAAAGGGACGAGGGGGCCCGUGGGGCAGAAAUUGGCCUCCCGUGAGCUCAUGCAUGCGGUUGGCCUAAAAGCGAGUCCUCGGCGACGCGCGCCACGACAAUCGGUGGUUGUCAAACCCUCGUGUCCCGUCGUGCGUGACGCGUCGCUCAUCGUGUGCUCCUUGACCCUGCUGUGUCGCGCUAGCGACGCUUCCAACGCGA

...............((.((.....((((((((..((((.(((((((....))))).)).))))...).))))))).....((((..(((((.((....)).)))))..)))).....((((......((((((((((((((((((...(((.((((....)))).)))......)))))).))).)))))))))..))))..................(((((((....)))))))......)).))

>AB243893 *Betula* *davurica* genes for ITS1, 5.8S rRNA, ITS2, haplotype:da1

ACGUCUGCCUGGGUGUCACGCAUCGUUGCCCCCAACCCCAUCUCCUUGUAAAGGGACGAGGGGGCCCGUGGGGCAGAAAUUGGCCUCCCGUGAGCUCAUGCAUGCGGUUGGCCUAAAAGCGAGUCCUCGGCGACGCGCGCCACGACAAUCGGUGGUUGUCAAACCCUCGUGUCCCGUCGUGCGUGACGCGUCGCUCAUCGUGUGCUCCUUGACCCUGCUGUGUCGCGCUAGCGACGCUUCCAACGCGA

...............((.((.....((((((((..((((.(((((((....))))).)).))))...).))))))).....((((..(((((.((....)).)))))..)))).....((((......((((((((((((((((((...(((.((((....)))).)))......)))))).))).)))))))))..))))..................(((((((....)))))))......)).))

>AB243892 *Betula* *grossa* genes for ITS1, 5.8S rRNA, ITS2, haplotype:gr

ACGUCUGCCUGGGUGUCACGCAUCGUUGCCCCCAACCCCAUCUCCUUGCAAAGGGACGAGGGGGCCUGUGGGGCAGAAAUUGGCCUCCCGUGAGCUCAUGCAUGCGGUUGGCCUAAAAGCGAGUCCUCGGCGACGCGCGCCACGACAAUCGGUGGUUGUCAAACCCUCGUGUCCCGUCGUGCGUGCCGCGUCGCUCAUCGUGUGCUCCUUGACCCUGCUGUGUCGCGCUAGCGACGCUUCCAACGCGA

...............((.((.....(((((((((.((((.(((((((....))))).)).))))..)).))))))).....((((..(((((.((....)).)))))..)))).....((((......((((((((((((((((((...(((.((((....)))).)))......)))))).))).)))))))))..))))..................(((((((....)))))))......)).))

>AB243891 *Betula* *platyphylla* genes for ITS1, 5.8S rRNA, ITS2, haplotype:pl

ACGUCUGCCUGGGUGUCACGCAUCGUUGCCCCCAACCCCAUCUCCUUGCAAAGGGACGAGGGGGCCUGUGGGGCAGAAAUUGGCCUCCCGUGAGCUCAUGCAUGCGGUUGGCCUAAAAGCGAGUCCUCGGCGACGCGCGCCACGACAAUCGGUGGUUGUCAAACCCUCGUGUCCCGUCGUGCGUGCCGCGUCGCUCAUCGUGUGCUCCUUGACCCUGCUGUGUCGCGCUAGCGACGCUUCCAACGCGA

...............((.((.....(((((((((.((((.(((((((....))))).)).))))..)).))))))).....((((..(((((.((....)).)))))..)))).....((((......((((((((((((((((((...(((.((((....)))).)))......)))))).))).)))))))))..))))..................(((((((....)))))))......)).))

>AB243890 *Betula* *maximovicziana* genes for ITS1, 5.8S rRNA, ITS2, haplotype:ma

ACGUCUGCCUGGGUGUCACGCAUCGUUGCCCCCAACCCCAUCUCCUUGCAAAGGGACGAGGGGGCCUGUGGGGCAGAAAUUGGCCUCCCGUGAGCUCAUGCAUGCGGUUGGCCUAAAAGCGAGUCCUCGGCGACGCGCGCCACGACAAUCGGUGGUUGACAAACCCUCGUGUCCCGUCGUGCGUGCCGCGUCGCUCAUCGUGUGCUCUUUGACCCUGCUGUGUCGCGCUAGCGACGCUUCCAACGCGA

...............((.((.....(((((((((.((((.(((((((....))))).)).))))..)).))))))).....((((..(((((.((....)).)))))..)))).....((((......((((((((((((((((((...(((.((((....)))).)))......)))))).))).)))))))))..))))..................(((((((....)))))))......)).))

>AB243889 *Betula* *ermanii* genes for ITS1, 5.8S rRNA, ITS2, haplotype:er4

ACGUCUGCCUGGGUGUCACGCAUCGUUGCCCCCAACCCCAUCUCCUUGCAAAGGGACGAGGGGGCCUGUGGGGCAGAAAUUGGCCUCCCGUGAGCUCAUGCAUGCGGUUGGCCUAAAAGCGAGUCCUCGGCGACGCGCGCCACGACAAUCGGUGGUUGACAAACCCUCGUGUCCCGUCGUGCGUGCCGCGUCGCUCAUCGUGUGCUCUUUGACCCUGCUGUGUCGCGCUAGCGACGCUUCCAACGCGA

...............((.((.....(((((((((.((((.(((((((....))))).)).))))..)).))))))).....((((..(((((.((....)).)))))..)))).....((((......((((((((((((((((((...(((.((((....)))).)))......)))))).))).)))))))))..))))..................(((((((....)))))))......)).))

>AB243888 *Betula* *ermanii* genes for ITS1, 5.8S rRNA, ITS2, haplotype:er3

ACGUCUGCCUGGGUGUCACGCAUCGUUGCCCCCAACCCCAUCUCCUUGCAAAGGGACGAGGGGGCCUGUGGGGCAGAAAUUGGCCUCCCGUGAGCUCAUGCAUGCGGUUGGCCUAAAAGCGAGUCCUCGGCGACGCGCGCCACGACAAUCGGUGGUUGACAAACCCUCGUGUCCCGUCGUGCGUGCCGUGUCGCUCAUCGUGUGCUCUUUGACCCUGCUGUGUCGCGCUAGCGACGCUUCCAACGCGA

...............((.((.....(((((((((.((((.(((((((....))))).)).))))..)).))))))).....((((..(((((.((....)).)))))..)))).....((((......((((((((((((((((((...(((.((((....)))).)))......)))))).))).)))))))))..))))..................(((((((....)))))))......)).))

>AB243887 *Betula* *ermanii* genes for ITS1, 5.8S rRNA, ITS2, haplotype:er2

ACGUCUGCCUGGGUGUCACGCAUCGUUGCCCCCAACCCCAUCUCCUUGCAAAGGGACGAGGGGGCCUGUGGGGCAGAAAUUGGCCUCCCGUGAGCUCAUGCAUGCGGUUGGCCUAAAAGCGAGUCCUCGGCGACGCGCGCCACGACAAUCGGUGGUUGACAAACCCUCGUGUCCCGUCGUGCGUGCCGCGUCGCUCAUCGUGUGCUCUUUGACCCUGCUGUGUCGCGCUAGCGACGCUUCCAACGCGA

...............((.((.....(((((((((.((((.(((((((....))))).)).))))..)).))))))).....((((..(((((.((....)).)))))..)))).....((((......((((((((((((((((((...(((.((((....)))).)))......)))))).))).)))))))))..))))..................(((((((....)))))))......)).))

>AB243886 *Betula* *ermanii* genes for ITS1, 5.8S rRNA, ITS2, haplotype:er1

ACGUCUGCCUGGGUGUCACGCAUCGUUGCCCCCAACCCCAUCUCCUUGCAAAGGGACGAGGGGGCCUGUGGGGCAGAAAUUGGCCUCCCGUGAGCUCAUGCAUGCGGUUGGCCUAAAAGCGAGUCCUCGGCGACGCGCGCCACGACAAUCGGUGGUUGACAAACCCUCGUGUCCCGUCGUGCGUGCCGUGUCGCUCAUCGUGUGCUCUUUGACCCUGCUGUGUCGCGCUAGCGACGCUUCCAACGCGA

...............((.((.....(((((((((.((((.(((((((....))))).)).))))..)).))))))).....((((..(((((.((....)).)))))..)))).....((((......((((((((((((((((((...(((.((((....)))).)))......)))))).))).)))))))))..))))..................(((((((....)))))))......)).))

>AB243885 *Betula* *corylifolia* genes for ITS1, 5.8S rRNA, ITS2, haplotype:co2

ACGUCUGCCUGGGUGUCACGCAUCGUUGCCCCCAACCCCAUCUCCUUGCAAAGGGACGAGGGG-CUUGUGGGGCAGAAAUUGGCCUCCCGUGAGCUUAUGCAUGCGGUUGGCCUAAAAGCGAGUCCUCGGCGACGCGCGCCACGACAAUCGGUGGUUGACAAACCCUCGUGUCCCGUCGUGUGUGCCGCGUCGCUCAUCAUGUGCUCUUUGACCCUGCUGUGUCGCGCUAGCGACGCUUCCAACGCGA

...............((.((.....(((((((((..(((.(((((((....))))).)).)))-..)).))))))).....((((..(((((.((....)).)))))..)))).....(.((......((((((((((((((((((...(((.((((....)))).)))......)))))).))).)))))))))..)).)..................(((((((....)))))))......)).))

>AB243884 *Betula* *corylifolia* genes for ITS1, 5.8S rRNA, ITS2, haplotype:co1

ACGUCUGCCUGGGUGUCACGCAUCGUUGCCCCCAACCCCAUCUCCUUGCAAAGGGACGAGGGG-CUUGUGGGGCAGAAAUUGGCCUCCCGUGAGCUUAUGCAUGCGGUUGGCCUAAAAGCGAGUCCUCGGCGACGCGCGCCACGACAAUCGGUGGUUGACAAACCCUCGUGUCCCGUCGUGUGUGCCGCGUCGCUCAUCGUGUGCUCUUUGACCCUGCUGUGUCGCGCUAGCGACGCUUCCAACGCGA

...............((.((.....(((((((((..(((.(((((((....))))).)).)))-..)).))))))).....((((..(((((.((....)).)))))..)))).....((((......((((((((((((((((((...(((.((((....)))).)))......)))))).))).)))))))))..))))..................(((((((....)))))))......)).))

>AB243883 *Betula* *globispica* genes for ITS1, 5.8S rRNA, ITS2, haplotype:gl

ACGUCUGCCUGGGUGUCACGCAUCGUUGCCCCCAACCCCAUCUCCUUGCAAAGGGACGAGGGG-CUUGUGGGGCAGAAAUUGGCCUCCCGUGAGCUUACGCAUGCGGUUGGCCUAAAAGCGAGUCCUCGGCGACGCGCGCCACGACAAUCGGUGGUUGACAAACCCUCGUGUCCCGUCGUGCGUGCCGCGUCGCUCAUCGUGUGCUCUUUGACCCUGCUGCGUCGCGCUAGCGACGCUUCCAACGCGA

...............((.((.....(((((((((..(((.(((((((....))))).)).)))-..)).))))))).....((((..(((((.((....)).)))))..)))).....((((......((((((((((((((((((...(((.((((....)))).)))......)))))).))).)))))))))..))))..................(((((((....)))))))......)).))

>AB243882 *Betula* *chichibuensis* genes for ITS1, 5.8S rRNA, ITS2, haplotype:ch2

ACGUCUGCCUGGGUGUCACGCAUCGUUGCCCCCAACCCCAUCUCCUUGCAAAGGGACGAAGGGGCCUGUGGGGCAGAAAUUGGCCUCCCGUGAGCUUAUGCAUGCGGUUGGCCUAAAAGUGAGUCCUCGGCGACGCGCGCCACGACAAUCGGUGGUUGACAAACCCUCGUGUCCCGUCGUGCGUGCCGCGUCGCUCAUCGUGUGCUCUUUGACCCUACUGUGUCGUGCUAGCGACGCUUCCAACGCGA

...............((.((.....(((((((((.((((.(((((((....))))).)).))))..)).))))))).....((((..(((((.((....)).)))))..)))).....((((......((((((((((((((((((...(((.((((....)))).)))......)))))).))).)))))))))..))))..................(((((((....)))))))......)).))

>AB243881 *Betula* *chichibuensis* genes for ITS1, 5.8S rRNA, ITS2, haplotype:ch1

ACGUCUGCCUGGGUGUCACGCAUCGUUGCCCCCAACCCCAUCUCCUUGCAAAGGGACGAAGGGGCCUGUGGGGCAGAAAUUGGCCUCCCGUGAGCUUAUGCAUGCGGUUGGCCUAAAAGUGAGUCCUCGGCGACGCGCGCCACGACAAUCGGUGGUUGACAAACCCUCGUGUCCCGUCGUGCGUGCCGCGUCGCUCAUCGUGUGCUCUUUGACCCUACUGUGUCGUGCUAGCGACGCUUCCAACGCGA

...............((.((.....(((((((((.((((.(((((((....))))).)).))))..)).))))))).....((((..(((((.((....)).)))))..)))).....((((......((((((((((((((((((...(((.((((....)))).)))......)))))).))).)))))))))..))))..................(((((((....)))))))......)).))

>AB243880 *Betula* *schmidtii* genes for ITS1, 5.8S rRNA, ITS2, haplotype:sc

ACGUCUGCCUGGGUGUCACGCAUCGUUGCCCCCAACCCCAUCUCCUUGCAAAGGGACGAGGGGGCCUGUGGGGCAGAAAUUGGCCUCCCGUGAGCUUAUGCAUGCGGUUGGCCUAAAAGCGAGUCCUCGGCGACGCGCGCCACGACAAUCGGUGGUUGACAAACCCUCGUGUCCCGUCGUGCGUGCCGUGUCGCUCAUCUUGUGCUCUUUGACCCUGCUGUGUCGCGCUAGCGACGCUUCCAACGCGA

...............((.((.....(((((((((.((((.(((((((....))))).)).))))..)).))))))).....((((..(((((.((....)).)))))..)))).....(.((......((((((((((((((((((...(((.((((....)))).)))......)))))).))).)))))))))..)).)..................(((((((....)))))))......)).))

>KT960303 *Betula* *nana* voucher ZA2012-3214 internal transcribed spacer 2, partial sequence

ACGUCUGCCUGGGUGUCACGCAUCGUUGCCCCCAACCCCAUCUCCUUGCAAAGGGACGAGGGGGCCUGUGGGGCAGAAAUUGGCCUCCCGUGAGCUCAUGCAUGCGGUUGGCCUAAAAGCGAGUCCUCGGCGACGCGCGCCACGACAAUCGGUGGUUGUCAAACCCUCGUGUCCCGUCGUGCGUGCCGCGUCGCUCAUCGUGUGCUCCUUGACCCUGCUGUGUCGCGCUAGCGACGCUUCCAACGCGA

...............((.((.....(((((((((.((((.(((((((....))))).)).))))..)).))))))).....((((..(((((.((....)).)))))..)))).....((((......((((((((((((((((((...(((.((((....)))).)))......)))))).))).)))))))))..))))..................(((((((....)))))))......)).))

>KT960259 *Betula* *nana* voucher ZA2012-3461 internal transcribed spacer 2, partial sequence

ACGUCUGCCUGGGUGUCACGCAUCGUUGCCCCCAACCCCAUCUCCUUGCAAAGGGACGAGGGGGCCUGUGGGGCAGAAAUUGGCCUCCCGUGAGCUCAUGCAUGCGGUUGGCCUAAAAGCGAGUCCUCGGCGACGCGCGCCACGACAAUCGGUGGUUGUCAAACCCUCGUGUCCCGUCGUGCGUGCCGCGUCGCUCAUCGUGUGCUCCUUGACCCUGCUGUGUCGCGCUAGCGACGCUUCCAACGCGA

...............((.((.....(((((((((.((((.(((((((....))))).)).))))..)).))))))).....((((..(((((.((....)).)))))..)))).....((((......((((((((((((((((((...(((.((((....)))).)))......)))))).))).)))))))))..))))..................(((((((....)))))))......)).))

>KT960160 *Betula* *nana* voucher ZA2012-2518 internal transcribed spacer 2, partial sequence

ACGUCUGCCUGGGUGUCACGCAUCGUUGCCCCCAACCCCAUCUCCUUGCAAAGGGACGAGGGGGCCUGUGGGGCAGAAAUUGGCCUCCCGUGAGCUCAUGCAUGCGGUUGGCCUAAAAGCGAGUCCUCGGCGACGCGCGCCACGACAAUCGGUGGUUGUCAAACCCUCGUGUCCCGUCGUGCGUGCCGCGUCGCUCAUCGUGUGCUCCUUGACCCUGCUGUGUCGCGCUAGCGACGCUUCCAACGCGA

...............((.((.....(((((((((.((((.(((((((....))))).)).))))..)).))))))).....((((..(((((.((....)).)))))..)))).....((((......((((((((((((((((((...(((.((((....)))).)))......)))))).))).)))))))))..))))..................(((((((....)))))))......)).))

>JN998979 *Betula* *pumila* voucher 09PROBE-05905 internal transcribed spacer 2, partial sequence

ACGUCUGCCUGGGUGUCACGCAUCGUUGCCCCCAACCCCAUCUCCUUGAAAAGGGACGAGGGGGCCUGUGGGGCAGAAAUUGGCCUCCCGUGAGCUCAUGCAUGCGGUUGGCCUAAAAGCGAGUCCUCGGCGACGCGCGCCACGACAAUCGGUGGUUGUCAAACCCUCGUGUCCCGUCGUGCGUGACGCGUCGCUCAUCGUGUGCUCCUUGACCCUGCUGUGUCGCGCUAGCGACGCUUCCAACGCGA

...............((.((.....(((((((((.((((.(((((((....))))).)).))))..)).))))))).....((((..(((((.((....)).)))))..)))).....((((......((((((((((((((((((...(((.((((....)))).)))......)))))).))).)))))))))..))))..................(((((((....)))))))......)).))

>JN998978 *Betula* *glandulosa* voucher BIOUG<CAN>:09PROBE-05527 internal transcribed spacer 2, partial sequence

ACGUCUGCCUGGGUGUCACGCAUCGUUGCCCCCAACCCCAUCUCCUUGCAAAGGGACGAGGGGGCCUGUGGGGCAGAAAUUGGCCUCCCGUGAGCUCAUGCAUGCGGUUGGCCUAAAAGCGAGUCCUCGGCGACGCGCGCCACGACAAUCGGUGGUUGUCAAACCCUCGUGUCCCGUCGUGCGUGCCGCGUCGCUCAUCGUGUGCUCCUUGACCCUGCUGUGUCGCGCUAGCGACGCUUCCAACGCGA

...............((.((.....(((((((((.((((.(((((((....))))).)).))))..)).))))))).....((((..(((((.((....)).)))))..)))).....((((......((((((((((((((((((...(((.((((....)))).)))......)))))).))).)))))))))..))))..................(((((((....)))))))......)).))

>JN998977 *Betula* *glandulosa* voucher BIOUG<CAN>:09PROBE-05118 internal transcribed spacer 2, partial sequence

ACGUCUGCCUGGGUGUCACGCAUCGUUGCCCCCAACCCCAUCUCCUUGCAAAGGGACGAGGGGGCCUGUGGGGCAGAAAUUGGCCUCCCGUGAGCUCAUGCAUGCGGUUGGCCUAAAAGCGAGUCCUCGGCGACGCGCGCCACGACAAUCGGUGGUUGUCAAACCCUCGUGUCCCGUCGUGCGUGCCGCGUCGCUCAUCGUGUGCUCCUUGACCCUGCUGUGUCGCGCUAGCGACGCUUCCAACGCGA

...............((.((.....(((((((((.((((.(((((((....))))).)).))))..)).))))))).....((((..(((((.((....)).)))))..)))).....((((......((((((((((((((((((...(((.((((....)))).)))......)))))).))).)))))))))..))))..................(((((((....)))))))......)).))

>JN998976 *Betula* *glandulosa* voucher BIOUG<CAN>:09PROBE-05010 internal transcribed spacer 2, partial sequence

ACGUCUGCCUGGGUGUCACGCAUCGUUGCCCCCAACCCCAUCUCCUUGCAAAGGGACGAGGGGGCCUGUGGGGCAGAAAUUGGCCUCCCGUGAGCUCAUGCAUGCGGUUGGCCUAAAAGCGAGUCCUCGGCGACGCGCGCCACGACAAUCGGUGGUUGUCAAACCCUCGUGUCCCGUCGUGCGUGCCGCGUCGCUCAUCGUGUGCUCCUUGACCCUGCUGUGUCGCGCUAGCGACGCUUCCAACGCGA

...............((.((.....(((((((((.((((.(((((((....))))).)).))))..)).))))))).....((((..(((((.((....)).)))))..)))).....((((......((((((((((((((((((...(((.((((....)))).)))......)))))).))).)))))))))..))))..................(((((((....)))))))......)).))

>JN998975 *Betula* *glandulosa* voucher 09PROBE-05279 internal transcribed spacer 2, partial sequence

ACGUCUGCCUGGGUGUCACGCAUCGUUGCCCCCAACCCCAUCUCCUUGAAAAGGGACGAGGGGGCCUGUGGGGCAGAAAUUGGCCUCCCGUGAGCUCAUGCAUGCGGUUGGCCUAAAAGCGAGUCCUCGGCGACGCGCGCCACGACAAUCGGUGGUUGUCAAACCCUCGUGUCCCGUCGUGCGUGACGCGUCGCUCAUCGUGUGCUCCUUGACCCUGCUGUGUCGCGCUAGCGACGCUUCCAACGCGA

...............((.((.....(((((((((.((((.(((((((....))))).)).))))..)).))))))).....((((..(((((.((....)).)))))..)))).....((((......((((((((((((((((((...(((.((((....)))).)))......)))))).))).)))))))))..))))..................(((((((....)))))))......)).))

>KT309028 *Betula* *occidentalis* isolate 2 5.8S ribosomal RNA gene and internal transcribed spacer 1, partial sequence; 5.8S ribosomal RNA gene, complete sequence; and internal transcribed spacer 2, partial sequence

ACGUCUGCCUGGGUGUCACGCAUCGUUGCCCCCAACCCCAUCUCCUUGCAAAGGGACGAGGGGGCCUGUGGGGCAGAAAUUGGCCUCCCGUGAGCUCAUGCAUGCGGUUGGCCUAAAAGCGAGUCCUCGGCGACGCGCGCCACGACAAUCGGUGGUUGUCAAACCCUCGUGUCCCGUCGUGCGUGCCGCGUCGCUCAUCGUGUGCUCCUUGACCCUGCUGUGUCGCGCUAGCGACGCUUCCAACGCG-

................(.((.....(((((((((.((((.(((((((....))))).)).))))..)).))))))).....((((..(((((.((....)).)))))..)))).....((((......((((((((((((((((((...(((.((((....)))).)))......)))))).))).)))))))))..))))..................(((((((....)))))))......)).)-

>KT309027 *Betula* *occidentalis* isolate 1 5.8S ribosomal RNA gene and internal transcribed spacer 1, partial sequence; 5.8S ribosomal RNA gene, complete sequence; and internal transcribed spacer 2, partial sequence

ACGUCUGCCUGGGUGUCACGCAUCGUUGCCCCCAACCCCAUCUCCUUGCAAAGGGACGAGGGGGCCUGUGGGGCAGAAAUUGGCCUCCCGUGAGCUCAUGCAUGCGGUUGGCCUAAAAGCGAGUCCUCGGCGACGCGCGCCACGACAAUCGGUGGUUGUCAAACCCUCGUGUCCCGUCGUGCGUGCCGCGUCGCUCAUCGUGUGCUCCUUGACCCUGCUGUGUCGCGCUAGCGACGCUUCCAACGCG-

................(.((.....(((((((((.((((.(((((((....))))).)).))))..)).))))))).....((((..(((((.((....)).)))))..)))).....((((......((((((((((((((((((...(((.((((....)))).)))......)))))).))).)))))))))..))))..................(((((((....)))))))......)).)-

>KT309026 *Betula* *humilis* isolate 3 5.8S ribosomal RNA gene and internal transcribed spacer 1, partial sequence; 5.8S ribosomal RNA gene, complete sequence; and internal transcribed spacer 2, partial sequence

ACGUCUGCCUGGGUGUCACGCAUCGUUGCCCCCAACCCCAUCUCCUUGCAAAGGGACGAGGGGGCCUGUGGGGCAGAAAUUGGCCUCCCGUGAGCUCAUGCAUGCGGUUGGCCUAAAAGCGAGUCCUCGGCGACGCGCGCCACGACAAUCGGUGGUUGUCAAACCCUCGUGUCCCGUCGUGCGUGCCGCGUCGCUCAUCGUGUGCUCCUUGACCCCGCUGUGUCGCACUAGCGACGCUUCCAACGCG-

................(.((.....(((((((((.((((.(((((((....))))).)).))))..)).))))))).....((((..(((((.((....)).)))))..)))).....((((......((((((((((((((((((...(((.((((....)))).)))......)))))).))).)))))))))..))))..................(((((((....)))))))......)).)-

>KT309025 *Betula* *humilis* isolate 2 5.8S ribosomal RNA gene and internal transcribed spacer 1, partial sequence; 5.8S ribosomal RNA gene, complete sequence; and internal transcribed spacer 2, partial sequence

ACGUCUGCCUGGGUGUCACGCAUCGUUGCCCCCAACCCCAUCUCCUUGCAAAGGGACGAGGGGGCCUGUGGGGCAGAAAUUGGCCUCCCGUGAGCUCAUGCAUGCGGUUGGCCUAAAAGCGAGUCCUCGGCGACGCGCGCCACGACAAUCGGUGGUUGUCAAACCCUCGUGUCCCGUCGUGCGUGCCGCGUCGCUCAUCGUGUGCUCCUUGACCCUGCUGUGUCGCGCUAGCGACGCUUCCAACGCG-

................(.((.....(((((((((.((((.(((((((....))))).)).))))..)).))))))).....((((..(((((.((....)).)))))..)))).....((((......((((((((((((((((((...(((.((((....)))).)))......)))))).))).)))))))))..))))..................(((((((....)))))))......)).)-

>KT309024 *Betula* *humilis* isolate 1 5.8S ribosomal RNA gene and internal transcribed spacer 1, partial sequence; 5.8S ribosomal RNA gene, complete sequence; and internal transcribed spacer 2, partial sequence

ACGUCUGCCUGGGUGUCACGCAUCGUUGCCCCCAACCCCAUCUCCUUGCAAAGGGACGAGGGGGCCUGUGGGGCAGAAAUUGGCCUCCCGUGAGCUCAUGCAUGCGGUUGGCCUAAAAGCGAGUCCUCGGCGACGCGCGCCACGACAAUCGGUGGUUGUCAAACCCUCGUGUCCCGUCGUGCGUGCCGCGUCGCUCAUCGUGUGCUCCUUGACCCCGCUGUGUCGCACUAGCGACGCUUCCAACGCG-

................(.((.....(((((((((.((((.(((((((....))))).)).))))..)).))))))).....((((..(((((.((....)).)))))..)))).....((((......((((((((((((((((((...(((.((((....)))).)))......)))))).))).)))))))))..))))..................(((((((....)))))))......)).)-

>KT309023 *Betula* *ovalifolia* isolate 2 5.8S ribosomal RNA gene and internal transcribed spacer 1, partial sequence; 5.8S ribosomal RNA gene, complete sequence; and internal transcribed spacer 2, partial sequence

ACGUCUGCCUGGGUGUCACGCAUCGUUGCCCCCAACCCCAUCUCCUUGAAAAGGGACGAGGGGGCCUGUGGGGCAGAAAUUGGCCUCCCGUGAGCUCAUGCAUGCGGUUGGUCUAAAAGCGAGUCCUCGGCGACGCGCGCCACGACAAUCGGUGGUUGUCAAACCCUCGUGUCCCGUCGUGCGUGACGCGUCGCUCAUCGUGUGCUCCUUGACCCUGCUGUGUCGUGCUAGCGACGCUUCCAACGCG-

................(.((.....(((((((((.((((.(((((((....))))).)).))))..)).))))))).....((((..(((((.((....)).)))))..)))).....((((......((((((((((((((((((...(((.((((....)))).)))......)))))).))).)))))))))..))))..................(((((((....)))))))......)).)-

>KT309022 *Betula* *ovalifolia* isolate 1 5.8S ribosomal RNA gene and internal transcribed spacer 1, partial sequence; 5.8S ribosomal RNA gene, complete sequence; and internal transcribed spacer 2, partial sequence

ACGUCUGCCUGGGUGUCACGCAUCGUUGCCCCCAACCCCAUCUCCUUGAAAAGGGACGAGGGGGCCUGUGGGGCAGAAAUUGGCCUCCCGUGAGCUCAUGCAUGCGGUUGGUCUAAAAGCGAGUCCUCGGCGACGCGCGCCACGACAAUCGGUGGUUGUCAAACCCUCGUGUCCCGUCGUGCGUGACGCGUCGCUCAUCGUGUGCUCCUUGACCCUGCUGUGUCGUGCUAGCGACGCUUCCAACGCG-

................(.((.....(((((((((.((((.(((((((....))))).)).))))..)).))))))).....((((..(((((.((....)).)))))..)))).....((((......((((((((((((((((((...(((.((((....)))).)))......)))))).))).)))))))))..))))..................(((((((....)))))))......)).)-

>KT309021 *Betula* *pumila* 5.8S ribosomal RNA gene and internal transcribed spacer 1, partial sequence; 5.8S ribosomal RNA gene, complete sequence; and internal transcribed spacer 2, partial sequence

ACGUCUGCCUGGGUGUCACGCAUCGUUGCCCCCAACCCCAUCUCCUUGAAAAGGGACGAGGGGGCCUGUGGGGCAGAAAUUGGCCUCCCGUGAGCUCAUGCAUGCGGUUGGCCUAAAAGCGAGUCCUCGGCGACGCGCGCCACGACAAUCGGUGGUUGUCAAACCCUCGUGUCCCGUCGUGCGUGACGCGUCGCUCAUCGUGUGCUCCUUGACCCUGCUGUGUCGCGCUAGCGACGCUUCCAACGCG-

................(.((.....(((((((((.((((.(((((((....))))).)).))))..)).))))))).....((((..(((((.((....)).)))))..)))).....((((......((((((((((((((((((...(((.((((....)))).)))......)))))).))).)))))))))..))))..................(((((((....)))))))......)).)-

>KT309020 *Betula* *nana* isolate 2 5.8S ribosomal RNA gene and internal transcribed spacer 1, partial sequence; 5.8S ribosomal RNA gene, complete sequence; and internal transcribed spacer 2, partial sequence

ACGUCUGCCUGGGUGUCACGCAUCGUUGCCCCCAACCCCAUCUCCUUGCAAAGGGACGAGGGGGCCUGUGGGGCAGAAAUUGGCCUCCCGUGAGCUCAUGCAUGCGGUUGGCCUAAAAGCGAGUCCUCGGCGACGCGCGCCACGACAAUCGGUGGUUGUCAAACCCUCGUGUCCCGUCGUGCGUGCCGCGUCGCUCAUCGUGUGCUCCUUGACCCUGCUGUGUCGCGCUAGCGACGCUUCCAACGCG-

................(.((.....(((((((((.((((.(((((((....))))).)).))))..)).))))))).....((((..(((((.((....)).)))))..)))).....((((......((((((((((((((((((...(((.((((....)))).)))......)))))).))).)))))))))..))))..................(((((((....)))))))......)).)-

>KT309019 *Betula* *nana* subsp. *exilis* 5.8S ribosomal RNA gene and internal transcribed spacer 1, partial sequence; 5.8S ribosomal RNA gene, complete sequence; and internal transcribed spacer 2, partial sequence

ACGUCUGCCUGGGUGUCACGCAUCGUUGCCCCCAACCCCAUCUCCUUGAAAAGGGACGAGGGGGCCUGUGGGGCAGAAAUUGGCCUCCCGUGAGCUCAUGCAUGCGGUUGGCCUAAAAGCGAGUCCUCGGCGACGCGCGCCACGACAAUCGGUGGUUGUCAAACCCUCGUGUCCCGUCGUGCGUGACGCGUCGCUCAUCGUGUGCUCCUUGACCCUGCUGUGUCGCGCUAGCGACGCUUCCAACGCG-

................(.((.....(((((((((.((((.(((((((....))))).)).))))..)).))))))).....((((..(((((.((....)).)))))..)))).....((((......((((((((((((((((((...(((.((((....)))).)))......)))))).))).)))))))))..))))..................(((((((....)))))))......)).)-

>KT309018 *Betula* *nana* isolate 1 5.8S ribosomal RNA gene and internal transcribed spacer 1, partial sequence; 5.8S ribosomal RNA gene, complete sequence; and internal transcribed spacer 2, partial sequence

ACGUCUGCCUGGGUGUCACGCAUCGUUGCCCCCAACCCCAUCUCCUUGCAAAGGGACGAGGGGGCCUGUGGGGCAGAAAUUGGCCUCCCGUGAGCUCAUGCAUGCGGUUGGCCUAAAAGCGAGUCCUCGGCGACGCGCGCCACGACAAUCGGUGGUUGUCAAACCCUCGUGUCCCGUCGUGCGUGCCGCGUCGCUCAUCGUGUGCUCCUUGACCCUGCUGUGUCGCGCUAGCGACGCUUCCAACGCG-

................(.((.....(((((((((.((((.(((((((....))))).)).))))..)).))))))).....((((..(((((.((....)).)))))..)))).....((((......((((((((((((((((((...(((.((((....)))).)))......)))))).))).)))))))))..))))..................(((((((....)))))))......)).)-

>KT309017 *Betula* *glandulosa* isolate 2 5.8S ribosomal RNA gene and internal transcribed spacer 1, partial sequence; 5.8S ribosomal RNA gene, complete sequence; and internal transcribed spacer 2, partial sequence

ACGUCUGCCUGGGUGUCACGCAUCGUUGCCCCCAACCCCAUCUCCUUGCAAAGGGACGAGGGGGCCUGUGGGGCAGAAAUUGGCCUCCCGUGAGCUCAUGCAUGCGGUUGGCCUAAAAGCGAGUCCUCGGCGACGCGCGCCACGACAAUCGGUGGUUGUCAAACCCUCGUGUCCCGUCGUGCGUGCCGCGUCGCUCAUCGUGUGCUCCUUGACCCUGCUGUGUCGCGCUAGCGACGCUUCCAACGCG-

................(.((.....(((((((((.((((.(((((((....))))).)).))))..)).))))))).....((((..(((((.((....)).)))))..)))).....((((......((((((((((((((((((...(((.((((....)))).)))......)))))).))).)))))))))..))))..................(((((((....)))))))......)).)-

>KT309016 *Betula* *cordifolia* isolate 2 5.8S ribosomal RNA gene and internal transcribed spacer 1, partial sequence; 5.8S ribosomal RNA gene, complete sequence; and internal transcribed spacer 2, partial sequence

ACGUCUGCCUGGGUGUCACGCAUCGUUGCCCCCAACCCCAUCUCCUUGCAAAGGGACGAGGGGGCCUGUGGGGCAGAAAUUGGCCUCCCGUGAGCUCAUGCAUGCGGUUGGCCUAAAAGCGAGUCCUCGGCGACGCGCGCCACGACAAUCGGUGGUUGUCAAACCCUCGUGUCCCGUCGUGCGUGCCGCGUCGCUCAUCGUGUGCUCCUUGACCCUGCUGUGUCGCGCUAGCGACGCUUCCAACGCG-

................(.((.....(((((((((.((((.(((((((....))))).)).))))..)).))))))).....((((..(((((.((....)).)))))..)))).....((((......((((((((((((((((((...(((.((((....)))).)))......)))))).))).)))))))))..))))..................(((((((....)))))))......)).)-

>KT309015 *Betula* *cordifolia* isolate 1 5.8S ribosomal RNA gene and internal transcribed spacer 1, partial sequence; 5.8S ribosomal RNA gene, complete sequence; and internal transcribed spacer 2, partial sequence

ACGUCUGCCUGGGUGUCACGCAUCGUUGCCCCCAACCCCAUCUCCUUGCAAAGGGACGAGGGGGCCUGUGGGGCAGAAAUUGGCCUCCCGUGAGCUCAUGCAUGCGGUUGGCCUAAAAGCGAGUCCUCGGCGACGCGCGCCACGACAAUCGGUGGUUGUCAAACCCUCGUGUCCCGUCGUGCGUGCCGCGUCGCUCAUCGUGUGCUCCUUGACCCUGCUGUGUCGCGCUAGCGACGCUUCCAACGCG-

................(.((.....(((((((((.((((.(((((((....))))).)).))))..)).))))))).....((((..(((((.((....)).)))))..)))).....((((......((((((((((((((((((...(((.((((....)))).)))......)))))).))).)))))))))..))))..................(((((((....)))))))......)).)-

>gb|KT309014 *Betula* *papyrifera* var. *commutata* 5.8S ribosomal RNA gene and internal transcribed spacer 1, partial sequence; 5.8S ribosomal RNA gene, complete sequence; and internal transcribed spacer 2, partial sequence

ACGUCUGCCUGGGUGUCACGCAUCGUUGCCCCCAACCCCAUCUCCUUGCAAAGGGACGAGGGGGCCUGUGGGGCAGAAAUUGGCCUCCCGUGAGCUCAUGCAUGCGGUUGGCCUAAAAGCGAGUCCUCGGCGACGCGCGCCACGACAAUCGGUGGUUGUCAAACCCUCGUGUCCCGUCGUGCGUGGCGCGUCGCUCAUCGUGUGCUCCUUGACCCUGCUGUGUCGCGCUAGCGACGCUUCCAACGCG-

................(.((.....(((((((((.((((.(((((((....))))).)).))))..)).))))))).....((((..(((((.((....)).)))))..)))).....((((......((((((((((((((((((...(((.((((....)))).)))......)))))).))).)))))))))..))))..................(((((((....)))))))......)).)-

>KT309013 *Betula* *papyrifera* isolate 3 5.8S ribosomal RNA gene and internal transcribed spacer 1, partial sequence; 5.8S ribosomal RNA gene, complete sequence; and internal transcribed spacer 2, partial sequence

ACGUCUGCCUGGGUGUCACGCAUCGUUGCCCCCAACCCCAUCUCCUUGCAAAGGGACGAGGGGGCCUGUGGGGCAGAAAUUGGCCUCCCGUGAGCUCAUGCAUGCGGUUGGCCUAAAAGCGAGUCCUCGGCGACGCGCGCCACGACAAUCGGUGGUUGUCAAACCCUCGUGUCCCGUCGUGCGUGCCGCGUCGCUCAUCGUGUGCUCCUUGACCCUGCUGUGUCGCGCUAGCGACGCUUCCAACGCG-

................(.((.....(((((((((.((((.(((((((....))))).)).))))..)).))))))).....((((..(((((.((....)).)))))..)))).....((((......((((((((((((((((((...(((.((((....)))).)))......)))))).))).)))))))))..))))..................(((((((....)))))))......)).)-

>KT309012 *Betula* *papyrifera* isolate 2 5.8S ribosomal RNA gene and internal transcribed spacer 1, partial sequence; 5.8S ribosomal RNA gene, complete sequence; and internal transcribed spacer 2, partial sequence

ACGUCUGCCUGGGUGUCACGCAUCGUUGCCCCCAACCCCAUCUCCUUGUAAAGGGACGAGGGGGCCUGUGGGGCAGAAAUUGGCCUCCCGUGAGCUCAUGCAUGCGGUUGGCCUAAAAGCGAGUCCUCGGCGACGCGCGCCACGACAAUCGGUGGUUGUCAAACCCUCGUGUCCCGUCGUGCGUGACGCGUCGCUCAUCGUGUGCUCCUUGACCCUGCUGUGUCGCGCUAGCGACGCUUCCAACGCG-

................(.((.....(((((((((.((((.(((((((....))))).)).))))..)).))))))).....((((..(((((.((....)).)))))..)))).....((((......((((((((((((((((((...(((.((((....)))).)))......)))))).))).)))))))))..))))..................(((((((....)))))))......)).)-

>KT309011 *Betula* *papyrifera* isolate 1 5.8S ribosomal RNA gene and internal transcribed spacer 1, partial sequence; 5.8S ribosomal RNA gene, complete sequence; and internal transcribed spacer 2, partial sequence

ACGUCUGCCUGGGUGUCACGCAUCGUUGCCCCCAACCCCAUCUCCUUGUAAAGGGACGAGGGGGCCUGUGGGGCAGAAAUUGGCCUCCCGUGAGCUCAUGCAUGCGGUUGGCCUAAAAGCGAGUCCUCGGCGACGCGCGCCACGACAAUCGGUGGUUGUCAAACCCUCGUGUCCCGUCGUGCGUGACGCGUCGCUCAUCGUGUGCUCCUUGACCCUGCUGUGUCGCGCUAGCGACGCUUCCAACGCG-

................(.((.....(((((((((.((((.(((((((....))))).)).))))..)).))))))).....((((..(((((.((....)).)))))..)))).....((((......((((((((((((((((((...(((.((((....)))).)))......)))))).))).)))))))))..))))..................(((((((....)))))))......)).)-

>KT309010 *Betula* *populifolia* isolate 3 5.8S ribosomal RNA gene and internal transcribed spacer 1, partial sequence; 5.8S ribosomal RNA gene, complete sequence; and internal transcribed spacer 2, partial sequence

ACGUCUGCCUGGGUGUCACGCAUCGUUGCCCCCAACCCCAUCUCCUUGCAAAGGGACGAGGGGGCCUGUGGGGCAGAAAUUGGCCUCCCGUGAGCUCAUGCAUGCGGUUGGCCUAAAAGCGAGUCCUCGGCGACGCGCGCCACGACAAUCGGUGGUUGUCAAACCCUCGUGUCCCGUCGUGCGUGCCGCGUCGCUCAUCGUGUGCUCCUUGACCCUGCUGUGUCGCGCUAGCGACGCUUCCAACGCG-

................(.((.....(((((((((.((((.(((((((....))))).)).))))..)).))))))).....((((..(((((.((....)).)))))..)))).....((((......((((((((((((((((((...(((.((((....)))).)))......)))))).))).)))))))))..))))..................(((((((....)))))))......)).)-

>KT309009 *Betula* *populifolia* isolate 2 5.8S ribosomal RNA gene and internal transcribed spacer 1, partial sequence; 5.8S ribosomal RNA gene, complete sequence; and internal transcribed spacer 2, partial sequence

ACGUCUGCCUGGGUGUCACGCAUCGUUGCCCCCAACCCCAUCUCCUUGCAAAGGGACGAGGGGGCCUGUGGGGCAGAAAUUGGCCUCCCGUGAGCUCAUGCAUGCGGUUGGCCUAAAAGCGAGUCCUCGGCGACGCGCGCCACGACAAUCGGUGGUUGUCAAACCCUCGUGUCCCGUCGUGCGUGCCGCGUCGCUCAUCGUGUGCUCCUUGACCCUGCUGUGUCGCGCUAGCGACGCUUCCAACGCG-

................(.((.....(((((((((.((((.(((((((....))))).)).))))..)).))))))).....((((..(((((.((....)).)))))..)))).....((((......((((((((((((((((((...(((.((((....)))).)))......)))))).))).)))))))))..))))..................(((((((....)))))))......)).)-

>KT309008 *Betula* *pendula* subsp. *mandshurica* isolate 5 5.8S ribosomal RNA gene and internal transcribed spacer 1, partial sequence; 5.8S ribosomal RNA gene, complete sequence; and internal transcribed spacer 2, partial sequence

ACGUCUGCCUGGGUGUCACGCAUCGUUGCCCCCAACCCCAUCUCCUUGCAAAGGGACGAGGGGGCCUGUGGGGCAGAAAUUGGCCUCCCGUGAGCUCAUGCAUGCGGUUGGCCUAAAAGCGAGUCCUCGGCGACGCGCGCCACGACAAUCGGUGGUUGUCAAACCCUCGUGUCCCGUCGUGCGUGCCGCGUCGCUCAUCGUGUGCUCCUUGACCCUGCUGUGUCGCGCUAGCGACGCUUCCAACGCG-

................(.((.....(((((((((.((((.(((((((....))))).)).))))..)).))))))).....((((..(((((.((....)).)))))..)))).....((((......((((((((((((((((((...(((.((((....)))).)))......)))))).))).)))))))))..))))..................(((((((....)))))))......)).)-

>KT309007 *Betula* *pendula* subsp. *pendula* isolate 6 5.8S ribosomal RNA gene and internal transcribed spacer 1, partial sequence; 5.8S ribosomal RNA gene, complete sequence; and internal transcribed spacer 2, partial sequence

ACGUCUGCCUGGGUGUCACGCAUCGUUGCCCCCAACCCCAUCUCCUUGCAAAGGGACGAGGGGGCCUGUGGGGCAGAAAUUGGCCUCCCGUGAGCUCAUGCAUGCGGUUGGCCUAAAAGCGAGUCCUCGGCGACGCGCGCCACGACAAUCGGUGGUUGUCAAACCCUCGUGUCCCGUCGUGCGUGCCGCGUCGCUCAUCGUGUGCUCCUUGACCCUGUUGUGUCGCGCUAGCGACGCUUCCAACGCG-

................(.((.....(((((((((.((((.(((((((....))))).)).))))..)).))))))).....((((..(((((.((....)).)))))..)))).....((((......((((((((((((((((((...(((.((((....)))).)))......)))))).))).)))))))))..))))..................(((((((....)))))))......)).)-

>KT309006 *Betula* *pendula* subsp. *pendula* isolate 5 5.8S ribosomal RNA gene and internal transcribed spacer 1, partial sequence; 5.8S ribosomal RNA gene, complete sequence; and internal transcribed spacer 2, partial sequence

ACGUCUGCCUGGGUGUCACGCAUCGUUGCCCCCAACCCCAUCUCCUUGCAAAGGGACGAGGGGGCCUGUGGGGCAGAAAUUGGCCUCCCGUGAGCUCAUGCAUGCGGUUGGCCUAAAAGCGAGUCCUCGGCGACGCGCGCCACGACAAUCGGUGGUUGUCAAACCCUCGUGUCCCGUCGUGCGUGCCGCGUCGCUCAUCGUGUGCUCCUUGACCCUGCUGUGUCGCGCUAGCGACGCUUCCAACGCG-

................(.((.....(((((((((.((((.(((((((....))))).)).))))..)).))))))).....((((..(((((.((....)).)))))..)))).....((((......((((((((((((((((((...(((.((((....)))).)))......)))))).))).)))))))))..))))..................(((((((....)))))))......)).)-

>KT309005 *Betula* *pendula* subsp. *mandshurica* isolate 4 5.8S ribosomal RNA gene and internal transcribed spacer 1, partial sequence; 5.8S ribosomal RNA gene, complete sequence; and internal transcribed spacer 2, partial sequence

ACGUCUGCCUGGGUGUCACGCAUCGUUGCCCCCAACCCCAUCUCCUUGCAAAGGGACGAGGGGGCCUGUGGGGCAGAAAUUGGCCUCCCGUGAGCUCAUGCAUGCGGUUGGCCUAAAAGCGAGUCCUCGGCGACGCGCGCCACGACAAUCGGUGGUUGUCAAACCCUCGUGUCCCGUCGUGCGUGCCGCGUCGCUCAUCGUGUGCUCCUUGACCCUGCUGUGUCGCGCUAGCGACGCUUCCAACGCG-

................(.((.....(((((((((.((((.(((((((....))))).)).))))..)).))))))).....((((..(((((.((....)).)))))..)))).....((((......((((((((((((((((((...(((.((((....)))).)))......)))))).))).)))))))))..))))..................(((((((....)))))))......)).)-

>KT309004 *Betula* *pendula* subsp. *szechuanica* isolate 3 5.8S ribosomal RNA gene and internal transcribed spacer 1, partial sequence; 5.8S ribosomal RNA gene, complete sequence; and internal transcribed spacer 2, partial sequence

ACGUCUGCCUGGGUGUCACGCAUCGUUGCCCCCAACCCCAUCUCCUUGCAAAGGGACGAGGGGGCCUGUGGGGCAGAAAUUGGCCUCCCGUGAGCUCAUGCAUGCGGUUGGCCUAAAAGCGAGUCCUCGGCGACGCGCGCCACGACAAUCGGUGGUUGUCAAACCCUCGUGUCCCGUCGUGCGUGCCGCGUCGCUCAUCGUGUGCUCCUUGACCCUGCUGUGUCGCGCUAGCGACGCUUCCAACGCG-

................(.((.....(((((((((.((((.(((((((....))))).)).))))..)).))))))).....((((..(((((.((....)).)))))..)))).....((((......((((((((((((((((((...(((.((((....)))).)))......)))))).))).)))))))))..))))..................(((((((....)))))))......)).)-

>KT309003 *Betula* *pendula* subsp. *szechuanica* isolate 2 5.8S ribosomal RNA gene and internal transcribed spacer 1, partial sequence; 5.8S ribosomal RNA gene, complete sequence; and internal transcribed spacer 2, partial sequence

ACGUCUGCCUGGGUGUCACGCAUCGUUGCCCCCAACCCCAUCUCCUUGCAAAGGGACGAGGGGGCCUGUGGGGCAGAAAUUGGCCUCCCGUGAGCUCAUGCAUGCGGUUGGCCUAAAAGCGAGUCCUCGGCGACGCGCGCCACGACAAUCGGUGGUUGUCAAACCCUCGUGUCCCGUCGUGCGUGCCGCGUCGCUCAUCGUGUGCUCCUUGACCCUGCUGUGUCGCGCUAGCGACGCUUCCAACGCG-

................(.((.....(((((((((.((((.(((((((....))))).)).))))..)).))))))).....((((..(((((.((....)).)))))..)))).....((((......((((((((((((((((((...(((.((((....)))).)))......)))))).))).)))))))))..))))..................(((((((....)))))))......)).)-

>KT309002 *Betula* *pendula* subsp. *pendula* isolate 4 5.8S ribosomal RNA gene and internal transcribed spacer 1, partial sequence; 5.8S ribosomal RNA gene, complete sequence; and internal transcribed spacer 2, partial sequence

ACGUCUGCCUGGGUGUCACGCAUCGUUGCCCCCAACCCCAUCUCCUUGCAAAGGGACGAGGGGGCCUGUGGGGCAGAAAUUGGCCUCCCGUGAGCUCAUGCAUGCGGUUGGCCUAAAAGCGAGUCCUCGGCGACGCGCGCCACGACAAUCGGUGGUUGUCAAACCCUCGUGUCCCGUCGUGCGUGCCGCGUCGCUCAUCGUGUGCUCCUUGACCCUGUUGUGUCGCGCUAGCGACGCUUCCAACGCG-

................(.((.....(((((((((.((((.(((((((....))))).)).))))..)).))))))).....((((..(((((.((....)).)))))..)))).....((((......((((((((((((((((((...(((.((((....)))).)))......)))))).))).)))))))))..))))..................(((((((....)))))))......)).)-

>KT309001 *Betula* *pendula* subsp. *pendula* isolate 3 5.8S ribosomal RNA gene and internal transcribed spacer 1, partial sequence; 5.8S ribosomal RNA gene, complete sequence; and internal transcribed spacer 2, partial sequence

ACGUCUGCCUGGGUGUCACGCAUCGUUGCCCCCAACCCCAUCUCCUUGCAAAGGGACGAGGGGGCCUGUGGGGCAGAAAUUGGCCUCCCGUGAGCUCAUGCAUGCGGUUGGCCUAAAAGCGAGUCCUCGGCGACGCGCGCCACGACAAUCGGUGGUUGUCAAACCCUCGUGUCCCGUCGUGCGUGCCGCGUCGCUCAUCGUGUGCUCCUUGACCCUGUUGUGUCGCGCUAGCGACGCUUCCAACGCG-

................(.((.....(((((((((.((((.(((((((....))))).)).))))..)).))))))).....((((..(((((.((....)).)))))..)))).....((((......((((((((((((((((((...(((.((((....)))).)))......)))))).))).)))))))))..))))..................(((((((....)))))))......)).)-

>KT309000 *Betula* *pendula* subsp. *pendula* isolate 2 5.8S ribosomal RNA gene and internal transcribed spacer 1, partial sequence; 5.8S ribosomal RNA gene, complete sequence; and internal transcribed spacer 2, partial sequence

ACGUCUGCCUGGGUGUCACGCAUCGUUGCCCCCAACCCCAUCUCCUUGCAAAGGGACGAGGGGGCCUGUGGGGCAGAAAUUGGCCUCCCGUGAGCUCAUGCAUGCGGUUGGCCUAAAAGCGAGUCCUCGGCGACGCGCGCCACGACAAUCGGUGGUUGUCAAACCCUCGUGUCCCGUCGUGCGUGCCGCGUCGCUCAUCGUGUGCUCCUUGACCCUGCUGUGUCGCGCUAGCGACGCUUCCAACGCG-

................(.((.....(((((((((.((((.(((((((....))))).)).))))..)).))))))).....((((..(((((.((....)).)))))..)))).....((((......((((((((((((((((((...(((.((((....)))).)))......)))))).))).)))))))))..))))..................(((((((....)))))))......)).)-

>KT308999 *Betula* *pendula* subsp. *mandshurica* isolate 3 5.8S ribosomal RNA gene and internal transcribed spacer 1, partial sequence; 5.8S ribosomal RNA gene, complete sequence; and internal transcribed spacer 2, partial sequence

ACGUCUGCCUGGGUGUCACGCAUCGUUGCCCCCAACCCCAUCUCCUUGCAAAGGGACGAGGGGGCCUGUGGGGCAGAAAUUGGCCUCCCGUGAGCUCAUGCAUGCGGUUGGCCUAAAAGCGAGUCCUCGGCGACGCGCGCCACGACAAUCGGUGGUUGUCAAACCCUCGUGUCCCGUCGUGCGUGCCGCGUCGCUCAUCGUGUGCUCCUUGACCCUGCUGUGUCGCGCUAGCGACGCUUCCAACGCG-

...............((.((.....(((((((((.((((.(((((((....))))).)).))))..)).))))))).....((((..(((((.((....)).)))))..)))).....((((......((((((((((((((((((...(((.((((....)))).)))......)))))).))).)))))))))..))))..................(((((((....)))))))......)).))

>KT308998 *Betula* *pendula* subsp. *pendula* isolate 1 5.8S ribosomal RNA gene and internal transcribed spacer 1, partial sequence; 5.8S ribosomal RNA gene, complete sequence; and internal transcribed spacer 2, partial sequence

ACGUCUGCCUGGGUGUCACGCAUCGUUGCCCCCAACCCCAUCUCCUUGCAAAGGGACGAGGGGGCCUGUGGGGCAGAAAUUGGCCUCCCGUGAGCUCAUGCAUGCGGUUGGCCUAAAAGCGAGUCCUCGGCGACGCGCGCCACGACAAUCGGUGGUUGUCAAACCCUCGUGUCCCGUCGUGCGUGCCGCGUCGCUCAUCGUGUGCUCCUUGACCCUGUUGUGUCGCGCUAGCGACGCUUCCAACGCG-

................(.((.....(((((((((.((((.(((((((....))))).)).))))..)).))))))).....((((..(((((.((....)).)))))..)))).....((((......((((((((((((((((((...(((.((((....)))).)))......)))))).))).)))))))))..))))..................(((((((....)))))))......)).)-

>KT308997 *Betula* *pendula* subsp. *szechuanica* isolate 1 5.8S ribosomal RNA gene and internal transcribed spacer 1, partial sequence; 5.8S ribosomal RNA gene, complete sequence; and internal transcribed spacer 2, partial sequence

ACGUCUGCCUGGGUGUCACGCAUCGUUGCCCCCAACCCCAUCUCCUUGCAAAGGGACGAGGGGGCCUGUGGGGCAGAAAUUGGCCUCCCGUGAGCUCAUGCAUGCGGUUGGCCUAAAAGCGAGUCCUCGGCGACGCGCGCCACGACAAUCGGUGGUUGUCAAACCCUCGUGUCCCGUCGUGCGUGCCGCGUCGCUCAUCGUGUGCUCCUUGACCCUGUUGUGUCGCGCUAGCGACGCUUCCAACGCG-

................(.((.....(((((((((.((((.(((((((....))))).)).))))..)).))))))).....((((..(((((.((....)).)))))..)))).....((((......((((((((((((((((((...(((.((((....)))).)))......)))))).))).)))))))))..))))..................(((((((....)))))))......)).)-

>KT308996 *Betula* *pendula* subsp. *mandshurica* isolate 2 5.8S ribosomal RNA gene and internal transcribed spacer 1, partial sequence; 5.8S ribosomal RNA gene, complete sequence; and internal transcribed spacer 2, partial sequence

ACGUCUGCCUGGGUGUCACGCAUCGUUGCCCCCAACCCCAUCUCCUUGCAAAGGGACGAGGGGGCCUGUGGGGCAGAAAUUGGCCUCCCGUGAGCUCAUGCAUGCGGUUGGCCUAAAAGCGAGUCCUCGGCGACGCGCGCCACGACAAUCGGUGGUUGUCAAACCCUCGUGUCCCGUCGUGCGUGCCGCGUCGCUCAUCGUGUGCUCCUUGACCCUGCUGUGUCGCGCUAGCGACGCUUCCAACGCG-

................(.((.....(((((((((.((((.(((((((....))))).)).))))..)).))))))).....((((..(((((.((....)).)))))..)))).....((((......((((((((((((((((((...(((.((((....)))).)))......)))))).))).)))))))))..))))..................(((((((....)))))))......)).)-

>KT308995 *Betula* *glandulosa* isolate 1 5.8S ribosomal RNA gene and internal transcribed spacer 1, partial sequence; 5.8S ribosomal RNA gene, complete sequence; and internal transcribed spacer 2, partial sequence

ACGUCUGCCUGGGUGUCACGCAUCGUUGCCCCCAACCCCAUCUCCUUGUAAAGGGACGAGGGGGCCUGUGGGGCAGAAAUUGGCCUCCCGUGAGCUCAUGCAUGCGGUUGGCCUAAAAGCGAGUCCUCGGCGACGCRCGCCACGACAAUCGGUGGUUGUCAAACCCUCGUGUCCCGUCGUGCGUGACGCGUCGCUCAUCGUGUGCUCCUUGACCCUGCUGUGUCGCGCUAGCGACGCUUCCAACGCG-

................(.((.....(((((((((.((((.(((((((....))))).)).))))..)).))))))).....((((..(((((.((....)).)))))..)))).....((((......((((((((((((((((((...(((.((((....)))).)))......)))))).))).)))))))))..))))..................(((((((....)))))))......)).)-

>KT308994 *Betula* *populifolia* isolate 1 5.8S ribosomal RNA gene and internal transcribed spacer 1, partial sequence; 5.8S ribosomal RNA gene, complete sequence; and internal transcribed spacer 2, partial sequence

ACGUCUGCCUGGGUGUCACGCAUCGUUGCCCCCAACCCCAUCUCCUUGCAAAGGGACGAGGGGGCCUGUGGGGCAGAAAUUGGCCUCCCGUGAGCUCAUGCAUGCGGUUGGCCUAAAAGCGAGUCCUCGGCGACGCGCGCCACGACAAUCGGUGGUUGUCAAACCCUCGUGUCCCGUCGUGCGUGCCGCGUCGCUCAUCGUGUGCUCCUUGACCCUGCUGUGUCGCGCUAGCGACGCUUCCAACGCG-

................(.((.....(((((((((.((((.(((((((....))))).)).))))..)).))))))).....((((..(((((.((....)).)))))..)))).....((((......((((((((((((((((((...(((.((((....)))).)))......)))))).))).)))))))))..))))..................(((((((....)))))))......)).)-

>KT308993 *Betula* *obscura* 5.8S ribosomal RNA gene and internal transcribed spacer 1, partial sequence; 5.8S ribosomal RNA gene, complete sequence; and internal transcribed spacer 2, partial sequence

ACGUCUGCCUGGGUGUCACGCAUCGUUGCCCCCAACCCCAUCUCCUUGCAAAGGGACGAGGGGGCCUGUGGGGCAGAAAUUGGCCUCCCGUGAGCUCAUGCAUGCGGUUGGCCUAAAAGCGAGUCCUCGGCGACGCGCGCCACGACAAUCGGUGGUUGUCAAACCCUCGUGUCCCGUCGUGCGUGCCGCGUCGCUCAUCGUGUGCUCCUUGACCCUGUUGUGUCGCGCUAGCGACGCUUCCAACGCG-

................(.((.....(((((((((.((((.(((((((....))))).)).))))..)).))))))).....((((..(((((.((....)).)))))..)))).....((((......((((((((((((((((((...(((.((((....)))).)))......)))))).))).)))))))))..))))..................(((((((....)))))))......)).)-

>KT308992 *Betula* *turkestanica* 5.8S ribosomal RNA gene and internal transcribed spacer 1, partial sequence; 5.8S ribosomal RNA gene, complete sequence; and internal transcribed spacer 2, partial sequence

ACGUCUGCCUGGGUGUCACGCAUCGUUGCCCCCAACCCCAUCUCCUUGCAAAGGGACGAGGGGGCCUGUGGGGCAGAAAUUGGCCUCCCGUGAGCUCAUGCAUGCGGUUGGCCUAAAAGCGAGUCCUCGGCGACGCGCGCCACGACAAUCGGUGGUUGUCAAACCCUCGUGUCCCGUCGUGCGUGCCGCGUCGCUCAUCGUGUGCUCCUUGACCCUGUUGUGUCGCGCUAGCGACGCUUCCAACGCG-

................(.((.....(((((((((.((((.(((((((....))))).)).))))..)).))))))).....((((..(((((.((....)).)))))..)))).....((((......((((((((((((((((((...(((.((((....)))).)))......)))))).))).)))))))))..))))..................(((((((....)))))))......)).)-

>KT308991 *Betula* *pendula* subsp. *mandshurica* isolate 1 5.8S ribosomal RNA gene and internal transcribed spacer 1, partial sequence; 5.8S ribosomal RNA gene, complete sequence; and internal transcribed spacer 2, partial sequence

ACGUCUGCCUGGGUGUCACGCAUCGUUGCCCCCAACCCCAUCUCCUUGCAAAGGGACGAGGGGGCCUGUGGGGCAGAAAUUGGCCUCCCGUGAGCUCAUGCAUGCGGUUGGCCUAAAAGCGAGUCCUCGGCGACGCGCGCCACGACAAUCGGUGGUUGUCAAACCCUCGUGUCCCGUCGUGCGUGCCGCGUCGCUCAUCGUGUGCUCCUUGACCCUGCUGUGUCGCGCUAGCGACGCUUCCAACGCG-

................(.((.....(((((((((.((((.(((((((....))))).)).))))..)).))))))).....((((..(((((.((....)).)))))..)))).....((((......((((((((((((((((((...(((.((((....)))).)))......)))))).))).)))))))))..))))..................(((((((....)))))))......)).)-

>KT308990 *Betula* *pendula* 5.8S ribosomal RNA gene and internal transcribed spacer 1, partial sequence; 5.8S ribosomal RNA gene, complete sequence; and internal transcribed spacer 2, partial sequence

ACGUCUGCCUGGGUGUCACGCAUCGUUGCCCCCAACCCCAUCUCCUUGCAAAGGGACGAGGGGGCCUGUGGGGCAGAAAUUGGCCUCCCGUGAGCUCAUGCAUGCGGUUGGCCUAAAAGCGAGUCCUCGGCGACGCGCGCCACGACAAUCGGUGGUUGUCAAACCCUCGUGUCCCGUCGUGCGUGCCGCGUCGCUCAUCGUGUGCUCCUUGACCCUGCUGUGUCGCGCUAGCGACGCUUCCAACGCG-

................(.((.....(((((((((.((((.(((((((....))))).)).))))..)).))))))).....((((..(((((.((....)).)))))..)))).....((((......((((((((((((((((((...(((.((((....)))).)))......)))))).))).)))))))))..))))..................(((((((....)))))))......)).)-

>KT308989 *Betula* *tianschanica* 5.8S ribosomal RNA gene and internal transcribed spacer 1, partial sequence; 5.8S ribosomal RNA gene, complete sequence; and internal transcribed spacer 2, partial sequence

ACGUCUGCCUGGGUGUCACGCAUCGUUGCCCCCAACCCCAUCUCCUUGAAAAGGGACGAGGGGGCCUGUGGGGCAGAAAUUGGCCUCCCGUGAGCUCAUGCAUGCGGUUGGCCUAAAAGCGAGUCCUCGGCGACGCGCGCCACGACAAUCGGUGGUUGUCAAACCCUCGUGUCCCGUCGUGCGUGACGCGUCGCUCAUCGUGUGCUCCUUGACCCUGCUGUGUCGCGCUAGCGACGCUUCCAACGCG-

................(.((.....(((((((((.((((.(((((((....))))).)).))))..)).))))))).....((((..(((((.((....)).)))))..)))).....((((......((((((((((((((((((...(((.((((....)))).)))......)))))).))).)))))))))..))))..................(((((((....)))))))......)).)-

>KT308988 *Betula × caerulea* isolate 2 5.8S ribosomal RNA gene and internal transcribed spacer 1, partial sequence; 5.8S ribosomal RNA gene, complete sequence; and internal transcribed spacer 2, partial sequence

ACGUCUGCCUGGGUGUCACGCAUCGUUGCCCCCAACCCCAUCUCCUUGUAAAGGGACGAGGGGGCCUGUGGGGCAGAAAUUGGCCUCCCGUGAGCUCAUGCAUGCGGUUGGCCUAAAAGCGAGUCCUCGGCGACGCGCGCCACGACAAUCGGUGGUUGUCAAACCCUCGUGUCCCGUCGUGCGUGACGCGUCGCUCAUCGUGUGCUCCUUGACCCUGCUGUGUCGCGCUAGCGACGCUUCCAACGCG-

................(.((.....(((((((((.((((.(((((((....))))).)).))))..)).))))))).....((((..(((((.((....)).)))))..)))).....((((......((((((((((((((((((...(((.((((....)))).)))......)))))).))).)))))))))..))))..................(((((((....)))))))......)).)-

>KT308987 *Betula × caerulea* isolate 1 5.8S ribosomal RNA gene and internal transcribed spacer 1, partial sequence; 5.8S ribosomal RNA gene, complete sequence; and internal transcribed spacer 2, partial sequence

ACGUCUGCCUGGGUGUCACGCAUCGUUGCCCCCAACCCCAUCUCCUUGCAAAGGGACGAGGGGGCCUGUGGGGCAGAAAUUGGCCUCCCGUGAGCUCAUGCAUGCGGUUGGCCUAAAAGCGAGUCCUCGGCGACGCGCGCCACGACAAUCGGUGGUUGUCAAACCCUCGUGUCCCGUCGUGCGUGCCGCGUCGCUCAUCGUGUGCUCCUUGACCCUGCUGUGUCGCGCUAGCGACGCUUCCAACGCG-

................(.((.....(((((((((.((((.(((((((....))))).)).))))..)).))))))).....((((..(((((.((....)).)))))..)))).....((((......((((((((((((((((((...(((.((((....)))).)))......)))))).))).)))))))))..))))..................(((((((....)))))))......)).)-

>KT308986 *Betula* *middendorffii* 5.8S ribosomal RNA gene and internal transcribed spacer 1, partial sequence; 5.8S ribosomal RNA gene, complete sequence; and internal transcribed spacer 2, partial sequence

ACGUCUGCCUGGGUGUCACGCAUCGUUGCCCCCAACCCCAUCUCCUUGCAAAGGGACGAGGGGGCCUGUGGGGCAGAAAUUGGCCUCCCGUGAGCUCAUGCAUGCGGUUGGCCUAAAAGCGAGUCCUCGGCGACGCGCGCCACGACAAUCGGUGGUUGUCAAACCCUCGUGUCCCGUCGUGCGUGCCGCGUCGCUCAUCGUGUGCUCCUUGACCCUGCUGUGUCGCGCUAGCGACGCUUCCAACGCG-

................(.((.....(((((((((.((((.(((((((....))))).)).))))..)).))))))).....((((..(((((.((....)).)))))..)))).....((((......((((((((((((((((((...(((.((((....)))).)))......)))))).))).)))))))))..))))..................(((((((....)))))))......)).)-

>KT308985 *Betula* *minor* 5.8S ribosomal RNA gene and internal transcribed spacer 1, partial sequence; 5.8S ribosomal RNA gene, complete sequence; and internal transcribed spacer 2, partial sequence

ACGUCUGCCUGGGUGUCACGCAUCGUUGCCCCCAACCCCAUCUCCUUGCAAAGGGACGAGGGGGCCUGUGGGGCAGAAAUUGGCCUCCCGUGAGCUCAUGCAUGCGGUUGGCCUAAAAGCGAGUCCUCGGCGACGCGCGCCACGACAAUCGGUGGUUGUCAAACCCUCGUGUCCCGUCGUGCGUGCCGCGUCGCUCAUCGUGUGCUCCUUGACCCUGCUGUGUCGCGCUAGCGACGCUUCCAACGCG-

................(.((.....(((((((((.((((.(((((((....))))).)).))))..)).))))))).....((((..(((((.((....)).)))))..)))).....((((......((((((((((((((((((...(((.((((....)))).)))......)))))).))).)))))))))..))))..................(((((((....)))))))......)).)-

>KT308984 *Betula* *microphylla* 5.8S ribosomal RNA gene and internal transcribed spacer 1, partial sequence; 5.8S ribosomal RNA gene, complete sequence; and internal transcribed spacer 2, partial sequence

ACGUCUGCCUGGGUGUCACGCAUCGUUGCCCCCAACCCCAUCUCCUUGCAAAGGGACGAGGGGGCCUGUGGGGCAGAAAUUGGCCUCCCGUGAGCUCAUGCAUGCGGUUGGCCUAAAAGCGAGUCCUCGGCGACGCGCGCCACGACAAUCGGUGGUUGUCAAACCCUCGUGUCCCGUCGUGCGUGACGCGUCGCUCAUCGUGUGCUCCUUGACCCUGCUGUGUCGCGCUAGCGACGCUUCCAACGCG-

................(.((.....(((((((((.((((.(((((((....))))).)).))))..)).))))))).....((((..(((((.((....)).)))))..)))).....((((......((((((((((((((((((...(((.((((....)))).)))......)))))).))).)))))))))..))))..................(((((((....)))))))......)).)-

>KT308983 *Betula* *pubescens* var. litiwinowii isolate 2 5.8S ribosomal RNA gene and internal transcribed spacer 1, partial sequence; 5.8S ribosomal RNA gene, complete sequence; and internal transcribed spacer 2, partial sequence

ACGUCUGCCUGGGUGUCACGCAUCGUUGCCCCCAACCCCAUCUCCUUGCAAAGGGACGAGGGGGCCUGUGGGGCAGAAAUUGGCCUCCCGUGAGCUCAUGCAUGCGGUUGGCCUAAAAGCGAGUCCUCGGCGACGCGCGCCACGACAAUCGGUGGUUGUCAAACCCUCGUGUCCCGUCGUGCGUGCCGCGUCGCUCAUCGUGUGCUCCUUGACCCUGCUGCGUCGCGCUAGCGACGCUUCCAACGCG-

................(.((.....(((((((((.((((.(((((((....))))).)).))))..)).))))))).....((((..(((((.((....)).)))))..)))).....((((......((((((((((((((((((...(((.((((....)))).)))......)))))).))).)))))))))..))))..................(((((((....)))))))......)).)-

>KT308982 *Betula* *pubescens* var. *pubescens* isolate 4 5.8S ribosomal RNA gene and internal transcribed spacer 1, partial sequence; 5.8S ribosomal RNA gene, complete sequence; and internal transcribed spacer 2, partial sequence

ACGUCUGCCUGGGUGUCACGCAUCGUUGCCCCCAACCCCAUCUCCUUGCAAAGGGACGAGGGGGCCUGUGGGGCAGAAAUUGGCCUCCCGUGAGCUCAUGCAUGCGGUUGGCCUAAAAGCGAGUCCUCGGCGACGCGCGCCACGACAAUCGGUGGUUGUCAAACCCUCGUGUCCCGUCGUGCGUGCCGCGUCGCUCAUCGUGUGCUCCUUGACCCUGCUGCGUCGCGCUAGCGACGCUUCCAACGCG-

................(.((.....(((((((((.((((.(((((((....))))).)).))))..)).))))))).....((((..(((((.((....)).)))))..)))).....((((......((((((((((((((((((...(((.((((....)))).)))......)))))).))).)))))))))..))))..................(((((((....)))))))......)).)-

>KT308981 *Betula* *pubescens* var. *pubescens* isolate 3 5.8S ribosomal RNA gene and internal transcribed spacer 1, partial sequence; 5.8S ribosomal RNA gene, complete sequence; and internal transcribed spacer 2, partial sequence

ACGUCUGCCUGGGUGUCACGCAUCGUUGCCCCCAACCCCAUCUCCUUGCAAAGGGACGAGGGGGCCUGUGGGGCAGAAAUUGGCCUCCCGUGAGCUCAUGCAUGCGGUUGGCCUAAAAGCGAGUCCUCGGCGACGCGCGCCACGACAAUCGGUGGUUGUCAAACCCUCGUGUCCCGUCGUGCGUGCCGCGUCGCUCAUCGUGUGCUCCUUGACCCUGCUGCGUCGCGCUAGCGACGCUUCCAACGCG-

................(.((.....(((((((((.((((.(((((((....))))).)).))))..)).))))))).....((((..(((((.((....)).)))))..)))).....((((......((((((((((((((((((...(((.((((....)))).)))......)))))).))).)))))))))..))))..................(((((((....)))))))......)).)-

>KT308980 *Betula* *pubescens* var. *pumila* isolate 3 5.8S ribosomal RNA gene and internal transcribed spacer 1, partial sequence; 5.8S ribosomal RNA gene, complete sequence; and internal transcribed spacer 2, partial sequence

ACGUCUGCCUGGGUGUCACGCAUCGUUGCCCCCAACCCCAUCUCCUUGCAAAGGGACGAGGGGGCCUGUGGGGCAGAAAUUGGCCUCCCGUGAGCUCAUGCAUGCGGUUGGCCUAAAAGCGAGUCCUCGGCGACGCGCGCCACGACAAUCGGUGGUUGUCAAACCCUCGUGUCCCGUCGUGCGUGCCGCGUCGCUCAUCGUGUGCUCCUUGACCCUGCUGCGUCGCGCUAGCGACGCUUCCAACGCG-

................(.((.....(((((((((.((((.(((((((....))))).)).))))..)).))))))).....((((..(((((.((....)).)))))..)))).....((((......((((((((((((((((((...(((.((((....)))).)))......)))))).))).)))))))))..))))..................(((((((....)))))))......)).)-

>KT308979 *Betula × utahensis* 5.8S ribosomal RNA gene and internal transcribed spacer 1, partial sequence; 5.8S ribosomal RNA gene, complete sequence; and internal transcribed spacer 2, partial sequence

ACGUCUGCCUGGGUGUCACGCAUCGUUGCCCCCAACCCCAUCUCCUUGYAAAGGGACGAGGGGGCCUGUGGGGCAGAAAUUGGCCUCCCGUGAGCUCAUGCAUGCGGUUGGCCUAAAAGCGAGUCCUCGGCGACGCGCGCCACGACAAUCGGUGGUUGUCAAACCCUCGUGUCCCGUCGUGCGUGMCGCGUCGCUCAUCGUGUGCUCCUUGACCCUGCUGUGUCGCGCUAGCGACGCUUCCAACGCG-

................(.((.....(((((((((.((((.(((((((....))))).)).))))..)).))))))).....((((..(((((.((....)).)))))..)))).....((((......((((((((((((((((((...(((.((((....)))).)))......)))))).))).)))))))))..))))..................(((((((....)))))))......)).)-

>KT308978 *Betula* *michauxii* 5.8S ribosomal RNA gene and internal transcribed spacer 1, partial sequence; 5.8S ribosomal RNA gene, complete sequence; and internal transcribed spacer 2, partial sequence

ACGUCUGCCUGGGUGUCACGCAUCGUUGCCCCCAACCCCAUCUCCUUGCAAAGGGACGAGGGGGCCUGUGGGGCAGAAAUUGGCCUCCCGUGAGCUCAUGCAUGCGGUUGGCCUAAAAGCGAGUUCUCGGCGACGCGCGCCACGACAAUCGGUGGUUGACAAACCCUGGUGUCCCGUCGUGCGUGCCGCGUCGCUCAUCGUGUGCUCUUUGACCCUGUUGUGUCGCGCUAGCGACGCUUCCAACGCG-

................(.((.....(((((((((.((((.(((((((....))))).)).))))..)).))))))).....((((..(((((.((....)).)))))..)))).....((((......((((((((((((((((((...(.(.((((....)))).).)......)))))).))).)))))))))..))))..................(((((((....)))))))......)).)-

>KT308977 *Betula* *pubescens* subsp. *celtiberica* isolate 2 5.8S ribosomal RNA gene and internal transcribed spacer 1, partial sequence; 5.8S ribosomal RNA gene, complete sequence; and internal transcribed spacer 2, partial sequence

ACGUCUGCCUGGGUGUCACGCAUCGUUGCCCCCAACCCCAUCUCCUUGCAAAGGGACGAGGGGGCCUGUGGGGCAGAAAUUGGCCUCCCGUGAGCUCAUGCAUGCGGUUGGCCUAAAAGCGAGUCCUCGGCGACGCGCGCCACGACAAUCGGUGGUUGUCAAACCCUCGUGUCCCGUCGUGCGUGCCGCGUCGCUCAUCGUGUGCUCCUUGACCCUGCUGUGUCGCGCUAGCGACGCUUCCAACGCG-

................(.((.....(((((((((.((((.(((((((....))))).)).))))..)).))))))).....((((..(((((.((....)).)))))..)))).....((((......((((((((((((((((((...(((.((((....)))).)))......)))))).))).)))))))))..))))..................(((((((....)))))))......)).)-

>KT308976 *Betula* *pubescens* var. *pumila* isolate 2 5.8S ribosomal RNA gene and internal transcribed spacer 1, partial sequence; 5.8S ribosomal RNA gene, complete sequence; and internal transcribed spacer 2, partial sequence

ACGUCUGCCUGGGUGUCACGCAUCGUUGCCCCCAACCCCAUCUCCUUGCAAAGGGACGAGGGGGCCUGUGGGGCAGAAAUUGGCCUCCCGUGAGCUCAUGCAUGCGGUUGGCCUAAAAGCGAGUCCUCGGCGACGCGCGCCACGACAAUCGGUGGUUGUCAAACCCUCGUGUCCCGUCGUGCGUGCCGCGUCGCUCAUCGUGUGCUCCUUGACCCUGCUGCGUCGCGCUAGCGACGCUUCCAACGCG-

................(.((.....(((((((((.((((.(((((((....))))).)).))))..)).))))))).....((((..(((((.((....)).)))))..)))).....((((......((((((((((((((((((...(((.((((....)))).)))......)))))).))).)))))))))..))))..................(((((((....)))))))......)).)-

>KT308975 *Betula* *pubescens* var. fragans isolate 2 5.8S ribosomal RNA gene and internal transcribed spacer 1, partial sequence; 5.8S ribosomal RNA gene, complete sequence; and internal transcribed spacer 2, partial sequence

ACGUCUGCCUGGGUGUCACGCAUCGUUGCCCCCAACCCCAUCUCCUUGCAAAGGGACGAGGGGGCCUGUGGGGCAGAAAUUGGCCUCCCGUGAGCUCAUGCAUGCGGUUGGCCUAAAAGCGAGUCCUCGGCGACGCGCGCCACGACAAUCGGUGGUUGUCAAACCCUCGUGUCCCGUCGUGCGUGCCGCGUCGCUCAUCGUGUGCUCCUUGACCCUGCUGCGUCGCGCUAGCGACGCUUCCAACGCG-

................(.((.....(((((((((.((((.(((((((....))))).)).))))..)).))))))).....((((..(((((.((....)).)))))..)))).....((((......((((((((((((((((((...(((.((((....)))).)))......)))))).))).)))))))))..))))..................(((((((....)))))))......)).)-

>KT308974 *Betula* *pubescens* var. fragans isolate 1 5.8S ribosomal RNA gene and internal transcribed spacer 1, partial sequence; 5.8S ribosomal RNA gene, complete sequence; and internal transcribed spacer 2, partial sequence

ACGUCUGCCUGGGUGUCACGCAUCGUUGCCCCCAACCCCAUCUCCUUGCAAAGGGACGAGGGGGCCUGUGGGGCAGAAAUUGGCCUCCCGUGAGCUCAUGCAUGCGGUUGGCCUAAAAGCGAGUCCUCGGCGACGCGCGCCACGACAAUCGGUGGUUGUCAAACCCUCGUGUCCCGUCGUGCGUGCCGCGUCGCUCAUCGUGUGCUCCUUGACCCUGCUGCGUCGCGCUAGCGACGCUUCCAACGCG-

................(.((.....(((((((((.((((.(((((((....))))).)).))))..)).))))))).....((((..(((((.((....)).)))))..)))).....((((......((((((((((((((((((...(((.((((....)))).)))......)))))).))).)))))))))..))))..................(((((((....)))))))......)).)-

>KT308973 *Betula* *pubescens* var. *pumila* isolate 1 5.8S ribosomal RNA gene and internal transcribed spacer 1, partial sequence; 5.8S ribosomal RNA gene, complete sequence; and internal transcribed spacer 2, partial sequence

ACGUCUGCCUGGGUGUCACGCAUCGUUGCCCCCAACCCCAUCUCCUUGCAAAGGGACGAGGGGGCCUGUGGGGCAGAAAUUGGCCUCCCGUGAGCUCAUGCAUGCGGUUGGCCUAAAAGCGAGUCCUCGGCGACGCGCGCCACGACAAUCGGUGGUUGUCAAACCCUCGUGUCCCGUCGUGCGUGCCGCGUCGCUCAUCGUGUGCUCCUUGACCCUGCUGCGUCGCGCUAGCGACGCUUCCAACGCG-

................(.((.....(((((((((.((((.(((((((....))))).)).))))..)).))))))).....((((..(((((.((....)).)))))..)))).....((((......((((((((((((((((((...(((.((((....)))).)))......)))))).))).)))))))))..))))..................(((((((....)))))))......)).)-

>KT308972 *Betula* *pubescens* subsp. *celtiberica* isolate 1 5.8S ribosomal RNA gene and internal transcribed spacer 1, partial sequence; 5.8S ribosomal RNA gene, complete sequence; and internal transcribed spacer 2, partial sequence

ACGUCUGCCUGGGUGUCACGCAUCGUUGCCCCCAACCCCAUCUCCUUGCAAAGGGACGAGGGGGCCUGUGGGGCAGAAAUUGGCCUCCCGUGAGCUCAUGCAUGCGGUUGGCCUAAAAGCGAGUCCUCGGCGACGCGCGCCACGACAAUCGGUGGUUGUCAAACCCUCGUGUCCCGUCGUGCGUGCCGCGUCGCUCAUCGUGUGCUCCUUGACCCUGUUGUGUCGCGCUAGCGACGCUUCCAACGCG-

................(.((.....(((((((((.((((.(((((((....))))).)).))))..)).))))))).....((((..(((((.((....)).)))))..)))).....((((......((((((((((((((((((...(((.((((....)))).)))......)))))).))).)))))))))..))))..................(((((((....)))))))......)).)-

>KT308971 *Betula* *pubescens* var. litiwinowii isolate 1 5.8S ribosomal RNA gene and internal transcribed spacer 1, partial sequence; 5.8S ribosomal RNA gene, complete sequence; and internal transcribed spacer 2, partial sequence

ACGUCUGCCUGGGUGUCACGCAUCGUUGCCCCCAACCCCAUCUCCUUGCAAAGGGACGAGGGGGCCUGUGGGGCAGAAAUUGGCCUCCCGUGAGCUCAUGCAUGCGGUUGGCCUAAAAGCGAGUCCUCGGCGACGCGCGCCACGACAAUCGGUGGUUGUCAAACCCUCGUGUCCCGUCGUGCGUGCCGCGUCGCUCAUCGUGUGCUCCUUGACCCUGCUGCGUCGCGCUAGCGACGCUUCCAACGCG-

................(.((.....(((((((((.((((.(((((((....))))).)).))))..)).))))))).....((((..(((((.((....)).)))))..)))).....((((......((((((((((((((((((...(((.((((....)))).)))......)))))).))).)))))))))..))))..................(((((((....)))))))......)).)-

>KT308970 *Betula* *pubescens* var. *pubescens* isolate 2 5.8S ribosomal RNA gene and internal transcribed spacer 1, partial sequence; 5.8S ribosomal RNA gene, complete sequence; and internal transcribed spacer 2, partial sequence

ACGUCUGCCUGGGUGUCACGCAUCGUUGCCCCCAACCCCAUCUCCUUGCAAAGGGACGAGGGGGCCUGUGGGGCAGAAAUUGGCCUCCCGUGAGCUCAUGCAUGCGGUUGGCCUAAAAGCGAGUCCUCGGCGACGCGCGCCACGACAAUCGGUGGUUGUCAAACCCUCGUGUCCCGUCGUGCGUGCCGCGUCGCUCAUCGUGUGCUCCUUGACCCUGCUGUGUCGCGCUAGCGACGCUUCCAACGCG-

................(.((.....(((((((((.((((.(((((((....))))).)).))))..)).))))))).....((((..(((((.((....)).)))))..)))).....((((......((((((((((((((((((...(((.((((....)))).)))......)))))).))).)))))))))..))))..................(((((((....)))))))......)).)-

>KT308969 *Betula* *pubescens* var. *pubescens* isolate 1 5.8S ribosomal RNA gene and internal transcribed spacer 1, partial sequence; 5.8S ribosomal RNA gene, complete sequence; and internal transcribed spacer 2, partial sequence

ACGUCUGCCUGGGUGUCACGCAUCGUUGCCCCCAACCCCAUCUCCUUGCAAAGGGACGAGGGGGCCUGUGGGGCAGAAAUUGGCCUCCCGUGAGCUCAUGCAUGCGGUUGGCCUAAAAGCGAGUCCUCGGCGACGCGCGCCACGACAAUCGGUGGUUGUCAAACCCUCGUGUCCCGUCGUGCGUGCCGCGUCGCUCAUCGUGUGCUCCUUGACCCUGCUGUGUCGCGCUAGCGACGCUUCCAACGCG-

................(.((.....(((((((((.((((.(((((((....))))).)).))))..)).))))))).....((((..(((((.((....)).)))))..)))).....((((......((((((((((((((((((...(((.((((....)))).)))......)))))).))).)))))))))..))))..................(((((((....)))))))......)).)-

>KT308968 *Betula* *browicziana* 5.8S ribosomal RNA gene and internal transcribed spacer 1, partial sequence; 5.8S ribosomal RNA gene, complete sequence; and internal transcribed spacer 2, partial sequence

ACGUCUGCCUGGGUGUCACGCAUCGUUGCCCCCAACCCCAUCUCCUUGCAAAGGGACGAGGGGGCCUGUGGGGCAGAAAUUGGCCUCCCGUGAGCUCAUGCAUGCGGUUGGCCUAAAAGCGAGUCCUCGGCGACGCGCGCCACGACAAUCGGUGGUUGUCAAACCCUCGUGUCCCGUCGUGCGUGCCGCGUCGCUCAUCGUGUGCUCCUUGACCCUGCUGUGUCGCGCUAGCGACGCUUCCAACGCG-

................(.((.....(((((((((.((((.(((((((....))))).)).))))..)).))))))).....((((..(((((.((....)).)))))..)))).....((((......((((((((((((((((((...(((.((((....)))).)))......)))))).))).)))))))))..))))..................(((((((....)))))))......)).)-

>KT308967 *Betula* *halophila* 5.8S ribosomal RNA gene and internal transcribed spacer 1, partial sequence; 5.8S ribosomal RNA gene, complete sequence; and internal transcribed spacer 2, partial sequence

ACGUCUGCCUGGGUGUCACGCAUCGUUGCCCCCAACCCCAUCUCCUUGMAAAGGGACGAGGGGGCCUGUGGGGCAGAAAUUGGCCUCCCGUGAGCUCAUGCAUGCGGUUGGCCUAAAAGCGAGUCCUCGGCGACGCGCGCCACGACAAUCGGUGGUUGUCAAACCCUCGUGUCCCGUCGUGCGUGMCGCGUCGCUCAUCGUGUGCUCCUUGACCCUGCUGUGUCGCGCUAGCGACGCUUCCAACGCG-

................(.((.....(((((((((.((((.(((((((....))))).)).))))..)).))))))).....((((..(((((.((....)).)))))..)))).....((((......((((((((((((((((((...(((.((((....)))).)))......)))))).))).)))))))))..))))..................(((((((....)))))))......)).)-

>KT308966 *Betula* *raddeana* 5.8S ribosomal RNA gene and internal transcribed spacer 1, partial sequence; 5.8S ribosomal RNA gene, complete sequence; and internal transcribed spacer 2, partial sequence

ACGUCUGCCUGGGUGUCACGCAUCGUUGCCCCCAACCCCAUCUCCUUGAAAAGGGACGAGGGGGCCUGUGGGGCAGAAAUUGGCCUCCCGUGAGCUCAUGCAUGCGGUUGGCCUAAAAGCGAGUCCUCGGCGACGCGCGCCACGACAAUCGGUGGUUGUCAAACCCUCGUGUCCCGUCGUGCGUGACGCGUCGCUCAUCGUGUGCUCCUUGACCCUGCUGUGUCGCGCUAGCGACGCUUCCAACGCG-

................(.((.....(((((((((.((((.(((((((....))))).)).))))..)).))))))).....((((..(((((.((....)).)))))..)))).....((((......((((((((((((((((((...(((.((((....)))).)))......)))))).))).)))))))))..))))..................(((((((....)))))))......)).)-

>KT308965 *Betula* *nigra* isolate 2 5.8S ribosomal RNA gene and internal transcribed spacer 1, partial sequence; 5.8S ribosomal RNA gene, complete sequence; and internal transcribed spacer 2, partial sequence

ACGUCUGCCUGGGUGUCACGCAUCGUUGCCCCCAACCCCAUCUCCUUGCAAAGGGACGAGAGGGCCAGUGGGGUAGAAAUUGGCCUCCCGUGAGCUCAUGCAUGCGGUUGGCCUAAAAGCGAGUCCUCGGCGACGCGCGCCACGACAAUCGGUGGUUGACAAACCCUCGUGUCCCGUCGUGCGUGCCGCGUUGCUCAUCGUGUGCUCUUUGACCCUGUUGUGUCGCGCUAGCGAUGCUUCCAAUGCG-

................(.((.....((((((((..(((..(((((((....))))).))..)))...).))))))).....((((..(((((.((....)).)))))..)))).....((((......((((((((((((((((((...(((.((((....)))).)))......)))))).))).)))))))))..))))..................(((((((....)))))))......)).)-

>KT308964 *Betula* *nigra* isolate 1 5.8S ribosomal RNA gene and internal transcribed spacer 1, partial sequence; 5.8S ribosomal RNA gene, complete sequence; and internal transcribed spacer 2, partial sequence

ACGUCUGCCUGGGUGUCACGCAUUGUUGCCCCCAACCCCAUCUCCUUGCAAAGGGACGAGGGGGCCAGUGGGGUAGAAAUUGGCCUCCCGUGAGCUCAUGCAUGCGGUUGGCCUAAAAGCGAGUCCUCGGCGACGCGCGCCACGACAAUCGGUGGUUGACAAACCCUCGUGUCCCGUCGUGCGUGCCGCGUUGCUCAUCGUGUGCUCUUUGACCCUGUUGUGUCGCGCUAGCGAUGCUUCCAAUGCG-

................(.((.....((((((((..((((.(((((((....))))).)).))))...).))))))).....((((..(((((.((....)).)))))..)))).....((((......((((((((((((((((((...(((.((((....)))).)))......)))))).))).)))))))))..))))..................(((((((....)))))))......)).)-

>KT308963 *Betula* *dahurica* isolate 2 5.8S ribosomal RNA gene and internal transcribed spacer 1, partial sequence; 5.8S ribosomal RNA gene, complete sequence; and internal transcribed spacer 2, partial sequence

ACGUCUGCCUGGGUGUCACGCAUCGUUGCCCCCAACCCCAUCUCCUUGUAAAGGGACGAGGGGGCCCGUGGGGCAGAAAUUGGCCUCCCGUGAGCUCAUGCAUGCGGUUGGCCUAAAAGCGAGUCCUCGGCGACGCGCGCCACGACAAUCGGUGGUUGUCAAACCCUCGUGUCCCGUCGUGCGUGACGCGUCGCUCAUCGUGUGCUCCUUGACCCUGCUGUGUCGCGCUAGCGACGCUUCCAACGCG-

................(.((.....((((((((..((((.(((((((....))))).)).))))...).))))))).....((((..(((((.((....)).)))))..)))).....((((......((((((((((((((((((...(((.((((....)))).)))......)))))).))).)))))))))..))))..................(((((((....)))))))......)).)-

>KT308962 *Betula* *dahurica* isolate 1 5.8S ribosomal RNA gene and internal transcribed spacer 1, partial sequence; 5.8S ribosomal RNA gene, complete sequence; and internal transcribed spacer 2, partial sequence

ACGUCUGCCUGGGUGUCACGCAUCGUUGCCCCCAACCCCAUCUCCUUGUAAAGGGACGAGGGGGCCCGUGGGGCAGAAAUUGGCCUCCCGUGAGCUCAUGCAUGCGGUUGGCCUAAAAGCGAGUCCUCGGCGACGCGCGCCACGACAAUCGGUGGUUGUCAAACCCUCGUGUCCCGUCGUGCGUGACGCGUCGCUCAUCGUGUGCUCCUUGACCCUGCUGUGUCGCGCUAGCGACGCUUCCAACGCG-

................(.((.....((((((((..((((.(((((((....))))).)).))))...).))))))).....((((..(((((.((....)).)))))..)))).....((((......((((((((((((((((((...(((.((((....)))).)))......)))))).))).)))))))))..))))..................(((((((....)))))))......)).)-

>KT308961 *Betula* *ashburneri* isolate 3 5.8S ribosomal RNA gene and internal transcribed spacer 1, partial sequence; 5.8S ribosomal RNA gene, complete sequence; and internal transcribed spacer 2, partial sequence

ACGUCUGCCUGGGUGUCACGCAUCGUUGCCCCCAACCCCAUCUCCUUGCAAAGGGACGAGGGGGCCAGUGGGGCAGAAAUUGGCCUCCCGUGAGCUCAUGCAUGCGGUUGGCCUAAAAGCGAGUCCUCGGCGACGCGCGCCACGACAAUCGGUGGUUGACAAACCCUCGUGUCCCGUCGUGCGUGCCGCGUCGCUCAUCGUGUGCUCUUUGACCCUGUUGUGUCGCGCUAGCGACGCUUCCAACGCG-

................(.((.....((((((((..((((.(((((((....))))).)).))))...).))))))).....((((..(((((.((....)).)))))..)))).....((((......((((((((((((((((((...(((.((((....)))).)))......)))))).))).)))))))))..))))..................(((((((....)))))))......)).)-

>KT308960 *Betula* *lanata* isolate 2 5.8S ribosomal RNA gene and internal transcribed spacer 1, partial sequence; 5.8S ribosomal RNA gene, complete sequence; and internal transcribed spacer 2, partial sequence

ACGUCUGCCUGGGUGUCACGCAUCGUUGCCCCCAACCCCAUCUCCUUGCAAAGGGACGAGGGGGCCUGUGGGGCAGAAAUUGGCCUCCCGUGAGCUCAUGCAUGCGGUUGGCCUAAAAGCGAGUCCUCGGCGACGCGCGCCACGACAAUCGGUGGUUGACAAACCCUCGUGUCCCGUCGUGCGUGCCGUGUCGCUCAUCGUGUGCUCUUUGACCCUGCUGUGUCGCGCUAGCGACGCUUCCAACGCG-

................(.((.....(((((((((.((((.(((((((....))))).)).))))..)).))))))).....((((..(((((.((....)).)))))..)))).....((((......((((((((((((((((((...(((.((((....)))).)))......)))))).))).)))))))))..))))..................(((((((....)))))))......)).)-

>KT308959 *Betula* *lanata* isolate 1 5.8S ribosomal RNA gene and internal transcribed spacer 1, partial sequence; 5.8S ribosomal RNA gene, complete sequence; and internal transcribed spacer 2, partial sequence

ACGUCUGCCUGGGUGUCACGCAUCGUUGCCCCCAACCCCAUCUCCUUGCAAAGGGACGAGGGGGCCUGUGGGGCAGAAAUUGGCCUCCCGUGAGCUCAUGCAUGCGGUUGGCCUAAAAGCGAGUCCUCGGCGACGCGCGCCACGACAAUCGGUGGUUGACAAACCCUCGUGUCCCGUCGUGCGUGCCGUGUCGCUCAUCGUGUGCUCUUUGACCCUGCUGUGUCGCGCUAGCGACGCUUCCAACGCG-

................(.((.....(((((((((.((((.(((((((....))))).)).))))..)).))))))).....((((..(((((.((....)).)))))..)))).....((((......((((((((((((((((((...(((.((((....)))).)))......)))))).))).)))))))))..))))..................(((((((....)))))))......)).)-

>KT308958 *Betula* *costata* 5.8S ribosomal RNA gene and internal transcribed spacer 1, partial sequence; 5.8S ribosomal RNA gene, complete sequence; and internal transcribed spacer 2, partial sequence

ACGUCUGCCUGGGUGUCACGCAUCGUUGCCCCCAACCCCAUCUCCUUGCAAAGGGACGAGGGGGCCAGUGGGGCAGAAAUUGGCCUCCCGUGAGCUCAUGCAUGCGGUUGGCCUAAAAGCGAGUCCUCGGCGACGCGCGCCACGACAAUCGGUGGUUGACAAACCCUCGUGUCCCGUYGUGCGUGCCGCGUCGCUCAUCGUGUGCUCUUUGACCCUGCUGUGUCGYGCUAGCGACGCUUCCAACGCG-

................(.((.....((((((((..((((.(((((((....))))).)).))))...).))))))).....((((..(((((.((....)).)))))..)))).....((((......((((((((((((((((((...(((.((((....)))).)))......)))))).))).)))))))))..))))..................(((((((....)))))))......)).)-

>KT308957 *Betula* *ermanii* isolate 2 5.8S ribosomal RNA gene and internal transcribed spacer 1, partial sequence; 5.8S ribosomal RNA gene, complete sequence; and internal transcribed spacer 2, partial sequence

ACGUCUGCCUGGGUGUCACGCAUCGUUGCCCCCAACCCCAUCUCCUUGCAAAGGGACGAGGGGGCCUGUGGGGCAGAAAUUGGCCUCCCGUGAGCUCAUGCAUGCGGUUGGCCUAAAAGCGAGUCCUCGGCGACGCGCGCCACGACAAUCGGUGGUUGACAAACCCUCGUGUCCCGUCGUGCGUGCCGYGUCGCUCAUCGUGUGCUCUUUGACCCUGCUGUGUYGCGCUAGCGACGCUUCCAACGCG-

................(.((.....(((((((((.((((.(((((((....))))).)).))))..)).))))))).....((((..(((((.((....)).)))))..)))).....((((......((((((((((((((((((...(((.((((....)))).)))......)))))).))).)))))))))..))))..................(((((((....)))))))......)).)-

>KT308956 *Betula* *ermanii* isolate 1 5.8S ribosomal RNA gene and internal transcribed spacer 1, partial sequence; 5.8S ribosomal RNA gene, complete sequence; and internal transcribed spacer 2, partial sequence

ACGUCUGCCUGGGUGUCACGCAUCGUUGCCCCCAACCCCAUCUCCUUGCAAAGGGACGAGGGGGCCUGUGGGGCAGAAAUUGGCCUCCCGUGAGCUCAUGCAUGCGGUUGGCCUAAAAGCGAGUCCUCGGCGACGCGCGCCACGACAAUCGGUGGUUGACAAACCCUCGUGUCCCGUCGUGCGUGCCGCGUCGCUCAUCGUGUGCUCUUUGACCCUGCUGUGUYGCGCUAGCGACGCUUCCAACGCG-

................(.((.....(((((((((.((((.(((((((....))))).)).))))..)).))))))).....((((..(((((.((....)).)))))..)))).....((((......((((((((((((((((((...(((.((((....)))).)))......)))))).))).)))))))))..))))..................(((((((....)))))))......)).)-

>KT308955 *Betula* *utilis* var. *prattii* 5.8S ribosomal RNA gene and internal transcribed spacer 1, partial sequence; 5.8S ribosomal RNA gene, complete sequence; and internal transcribed spacer 2, partial sequence

ACGUCUGCCUGGGUGUCACGCAUCGUUGCCCCCAACCCCAUCUCCUUGCAAAGGGACGAGGGGGCCUGUGGGGCAGAAAUUGGCCUCCCGUGAGCUCAUGCAUGCGGUUGGCCUAAAAGCGAGUCCUCGGCGACGCGCGCCACGACAAUCGGUGGUUGACAAACCCUCGUGUCCCGUCGUGCGUGCCGCGUCGCUCAUCGUGUGCUCUUUGACCCUGCUGUGUCGCGCUAGCGACGCUUCCAACGCG-

................(.((.....(((((((((.((((.(((((((....))))).)).))))..)).))))))).....((((..(((((.((....)).)))))..)))).....((((......((((((((((((((((((...(((.((((....)))).)))......)))))).))).)))))))))..))))..................(((((((....)))))))......)).)-

>KT308954 *Betula* *albosinensis* isolate 2 5.8S ribosomal RNA gene and internal transcribed spacer 1, partial sequence; 5.8S ribosomal RNA gene, complete sequence; and internal transcribed spacer 2, partial sequence

ACGUCUGCCUGGGUGUCACGCAUCGUUGCCCCCAACCCCAUCUCCUUGCAAAGGGACGAGGGGGCCUGUGGGGCAGAAAUUGGCCUCCCGUGAGCUCAUGCAUGCGGUUGGCCUAAAAGCGAGUCCUCGGCGACGCGCGCCACGACAAUCGGUGGUUGACAAACCCUCGUGUCCCGUCGUGCGUGCCGCGUCGCUCAUCGUGUGCUCUUUGACCCUGCUGUGUCGCGCUAGCGACGCUUCCAACGCG-

................(.((.....(((((((((.((((.(((((((....))))).)).))))..)).))))))).....((((..(((((.((....)).)))))..)))).....((((......((((((((((((((((((...(((.((((....)))).)))......)))))).))).)))))))))..))))..................(((((((....)))))))......)).)-

>KT308953 *Betula* *ashburneri* isolate 2 5.8S ribosomal RNA gene and internal transcribed spacer 1, partial sequence; 5.8S ribosomal RNA gene, complete sequence; and internal transcribed spacer 2, partial sequence

ACGUCUGCCUGGGUGUCACGCAUCGUUGCCCCCAACCCCAUCUCCUUGCAAAGGGACGAGGGGGCCAGUGGGGCAGAAAUUGGCCUCCCGUGAGCUCAUGCAUGCGGUUGGCCUAAAAGCGAGUCCUCGGCGACGCGCGCCACGACAAUCGGUGGUUGACAAACCCUCGUGUCCCGUCGUGCGUGCCGCGUCGCUCAUCGUGUGCUCUUUGACCCUGUUGUGUCGCGCUAGCGACGCUUCCAACGCG-

................(.((.....((((((((..((((.(((((((....))))).)).))))...).))))))).....((((..(((((.((....)).)))))..)))).....((((......((((((((((((((((((...(((.((((....)))).)))......)))))).))).)))))))))..))))..................(((((((....)))))))......)).)-

>KT308952 *Betula* *ashburneri* isolate 1 5.8S ribosomal RNA gene and internal transcribed spacer 1, partial sequence; 5.8S ribosomal RNA gene, complete sequence; and internal transcribed spacer 2, partial sequence

ACGUCUGCCUGGGUGUCACGCAUCGUUGCCCCCAACCCCAUCUCCUUGCAAAGGGACGAGGGGGCCAGUGGGGCAGAAAUUGGCCUCCCGUGAGCUCAUGCAUGCGGUUGGCCUAAAAGCGAGUCCUCGGCGACGCGCGCCACGACAAUCGGUGGUUGACAAACCCUCGUGUCCCGUCGUGCGUGCCGCGUCGCUCAUCGUGUGCUCUUUGACCCUGUUGUGUCGCGCUAGCGACGCUUCCAACGCG-

................(.((.....((((((((..((((.(((((((....))))).)).))))...).))))))).....((((..(((((.((....)).)))))..)))).....((((......((((((((((((((((((...(((.((((....)))).)))......)))))).))).)))))))))..))))..................(((((((....)))))))......)).)-

>KT308951 *Betula* *utilis* var. jacquemontii 5.8S ribosomal RNA gene and internal transcribed spacer 1, partial sequence; 5.8S ribosomal RNA gene, complete sequence; and internal transcribed spacer 2, partial sequence

ACGUCUGCCUGGGUGUCACGCAUCGUUGCCCCCAACCCCAUCUCCUUGCAAAGGGACGAGGGGGCCAGUGGGGCAGAAAUUGGCCUCCCGUGAGCUCAUGCAUGCGGUUGGCCUAAAAGCGAGUCCUCGGCGACGCGCGCCACGACAAUCGGUGGUUGACAAACCCUCGUGUCCCGUCGUGCGUGCCGCGUCGCUCAUCGUGUGCUCUUUGACCCUGUUGUGUCGCGCUAGCGACGCUUCCAACGCG-

................(.((.....((((((((..((((.(((((((....))))).)).))))...).))))))).....((((..(((((.((....)).)))))..)))).....((((......((((((((((((((((((...(((.((((....)))).)))......)))))).))).)))))))))..))))..................(((((((....)))))))......)).)-

>KT308950 *Betula* *utilis* var. *occidentalis* isolate 2 5.8S ribosomal RNA gene and internal transcribed spacer 1, partial sequence; 5.8S ribosomal RNA gene, complete sequence; and internal transcribed spacer 2, partial sequence

ACGUCUGCCUGGGUGUCACGCAUCGUUGCCCCCAACCCCAUCUCCUUGCAAAGGGACGAGGGGGCCWGUGGGGCAGAAAUUGGCCUCCCGUGAGCUCAUGCAUGCGGUUGGCCUAAAAGCGAGUCCUCGGCGAYGCGCGCCACGACAAUCGGUGGUUGACAAACCCUCGUGUCCCGUCGUGCGUGCCGCGUCGCUCAUUGUGUGCUCUUUGACCCUGUUGUGUCGCGCUAGCGACGCUUCCAACGCG-

................(.((.....((((((((..((((.(((((((....))))).)).))))...).))))))).....((((..(((((.((....)).)))))..)))).....((((......((((((((((((((((((...(((.((((....)))).)))......)))))).))).)))))))))..))))..................(((((((....)))))))......)).)-

>KT308949 *Betula* *utilis* isolate 2 5.8S ribosomal RNA gene and internal transcribed spacer 1, partial sequence; 5.8S ribosomal RNA gene, complete sequence; and internal transcribed spacer 2, partial sequence

ACGUCUGCCUGGGUGUCACGCAUCGUUGCCCCCAACCCCAUCUCCUUGCAAAGGGACGAGGGGGCCAGUGGGGCAGAAAUUGGCCUCCCGUGAGCUCAUGCAUGCGGUUGGCCUAAAAGCGAGUCCUCGGCGACGCACGCCACGACAAUCGGUGGUUGACAAACCCUCGUGUCCCGUCGUGCGUGCCGCGUCGCUCAUCGUGUGCUCUUUGACCCUGUUGUGUCGCGCUAGCGACGCUUCCAACGCG-

................(.((.....((((((((..((((.(((((((....))))).)).))))...).))))))).....((((..(((((.((....)).)))))..)))).....((((......((((((((.(((((((((...(((.((((....)))).)))......)))))).)))..))))))))..))))..................(((((((....)))))))......)).)-

>KT308948 *Betula* *utilis* isolate 1 5.8S ribosomal RNA gene and internal transcribed spacer 1, partial sequence; 5.8S ribosomal RNA gene, complete sequence; and internal transcribed spacer 2, partial sequence

ACGUCUGCCUGGGUGUCACGCAUCGUUGCCCCCAACCCCAUCUCCUUGCAAAGGGACGAGGGGGCCAGUGGGGCAGAAAUUGGCCUCCCGUGAGCUCAUGCAUGCGGUUGGCCUAAAAGCGAGUCCUCGGCGACGCGCGCCACGACAAUCGGUGGUUGACAAACCCUCGUGUCCCGUCGUGCGUGCCGCGUCGCUCAUCGUGUGCUCUUUGACCCUGUUGUGUCGCGCUAGCGACGCUUCCAACGCG-

................(.((.....((((((((..((((.(((((((....))))).)).))))...).))))))).....((((..(((((.((....)).)))))..)))).....((((......((((((((((((((((((...(((.((((....)))).)))......)))))).))).)))))))))..))))..................(((((((....)))))))......)).)-

>KT308947 *Betula* *albosinensis* var. septentrionalis 5.8S ribosomal RNA gene and internal transcribed spacer 1, partial sequence; 5.8S ribosomal RNA gene, complete sequence; and internal transcribed spacer 2, partial sequence

ACGUCUGCCUGGGUGUCACGCAUCGUUGCCCCCAACCCCAUCUCCUUGCAAAGGGACGAGGGGGCCUGUGGGGCAGAAAUUGGCCUCCCGUGAGCUCAUGCAUGCGGUUGGCCUAAAAGYGAGUCCUCGGCGACGCGCGCCACGACAAUCGGUGGUUGACAAACCCUCGUGUCCCGUCGUGCGUGCCGCGUCGCUCAUCGUGUGCUCUUUGACCCUGCUGUGUCGCGCUAGCGACGCUUCCAACGCG-

................(.((.....(((((((((.((((.(((((((....))))).)).))))..)).))))))).....((((..(((((.((....)).)))))..)))).....((((......((((((((((((((((((...(((.((((....)))).)))......)))))).))).)))))))))..))))..................(((((((....)))))))......)).)-

>KT308946 *Betula* *maximovicziana* isolate 2 5.8S ribosomal RNA gene and internal transcribed spacer 1, partial sequence; 5.8S ribosomal RNA gene, complete sequence; and internal transcribed spacer 2, partial sequence

ACGUCUGCCUGGGUGUCACGCAUCGUUGCCCCCAACCCCAUCUCCUUGCAAAGGGACGAGGGGGCCUGUGGGGCAGAAAUUGGCCUCCCGUGAGCUCAUGCAUGCGGUUGGCCUAAAAGCGAGUCCUCGGCGACGCGCGCCACGACAAUCGGUGGUUGACAAACCCUCGUGUCCCGUCGUGCGUGCCGCGUCGCUCAUCGUGUGCUCUUUGACCCUGCUGUGUCGCGCUAGCGACGCUUCCAACGCG-

................(.((.....(((((((((.((((.(((((((....))))).)).))))..)).))))))).....((((..(((((.((....)).)))))..)))).....((((......((((((((((((((((((...(((.((((....)))).)))......)))))).))).)))))))))..))))..................(((((((....)))))))......)).)-

>KT308945 *Betula* *maximovicziana* isolate 1 5.8S ribosomal RNA gene and internal transcribed spacer 1, partial sequence; 5.8S ribosomal RNA gene, complete sequence; and internal transcribed spacer 2, partial sequence

ACGUCUGCCUGGGUGUCACGCAUCGUUGCCCCCAACCCCAUCUCCUUGCAAAGGGACGAGGGGGCCUGUGGGGCAGAAAUUGGCCUCCCGUGAGCUCAUGCAUGCGGUUGGCCUAAAAGCGAGUCCUCGGCGACGCGCGCCACGACAAUCGGUGGUUGACAAACCCUCGUGUCCCGUCGUGCGUGCCGCGUCGCUCAUCGUGUGCUCUUUGACCCUGCUGUGUCGCGCUAGCGACGCUUCCAACGCG-

................(.((.....(((((((((.((((.(((((((....))))).)).))))..)).))))))).....((((..(((((.((....)).)))))..)))).....((((......((((((((((((((((((...(((.((((....)))).)))......)))))).))).)))))))))..))))..................(((((((....)))))))......)).)-

>KT308944 *Betula* *luminifera* isolate 3 5.8S ribosomal RNA gene and internal transcribed spacer 1, partial sequence; 5.8S ribosomal RNA gene, complete sequence; and internal transcribed spacer 2, partial sequence

ACGUCUGCCUGGGUGUCACGCAUCGUUGCCCCCAACCCCAUCUCCUUGCAAAGGGACGAGGGGGCCAGUGGGGCAGAAAUUGGCCUCCCGUGAGCUCAUGCAUGCGGUUGGCCUAAAAGCGAGUCCUCGGCGACGCGCGCCACGACAAUCGGUGGUUGACAAACCCUCGUGUCCCGUCGUGCGUGCCGCGUUGCUCAUCGUGUGCUCUUUGACCCUGUUGUGUCGCGCUAGCGAUGCUUCCAACGCG-

................(.((.....((((((((..((((.(((((((....))))).)).))))...).))))))).....((((..(((((.((....)).)))))..)))).....((((......((((((((((((((((((...(((.((((....)))).)))......)))))).))).)))))))))..))))..................(((((((....)))))))......)).)-

>KT308943 *Betula* *luminifera* isolate 2 5.8S ribosomal RNA gene and internal transcribed spacer 1, partial sequence; 5.8S ribosomal RNA gene, complete sequence; and internal transcribed spacer 2, partial sequence

ACGUCUGCCUGGGUGUCACGCAUCGUUGCCCCCAACCCCAUCUCCUUGCAAAGGGACGAGGGGGCCARUGGGGCAGAAAUUGGCCUCCCGUGAGCUCAUGCAUGCGGUUGGCCUAAAAGCGAGUCCUCGGCGACGCGCGCCACGACAAUCGGUGGUUGACAAACCCUCGUGUCCCGUCGUGCGUGCCGCGUUGCUCAUCGUGUGCUCUUUGACCCUGCUGUGUCGCGCUAGCGACGCUUCCAACGCG-

................(.((.....((((((((..((((.(((((((....))))).)).))))...).))))))).....((((..(((((.((....)).)))))..)))).....((((......((((((((((((((((((...(((.((((....)))).)))......)))))).))).)))))))))..))))..................(((((((....)))))))......)).)-

>KT308942 *Betula* *hainanensis* 5.8S ribosomal RNA gene and internal transcribed spacer 1, partial sequence; 5.8S ribosomal RNA gene, complete sequence; and internal transcribed spacer 2, partial sequence

ACGUCUGCCUGGGUGUCACGCAUCGUUGCCCCCAACCCCAUCUCCUUGCAAAGGGACGAGGGGGCCAAUGGGGCAGAAAUUGGCCUCCCGUGAGCUCAUGCAUGCGGUUGGCCUAAAAGCGAGUCCUCGGCGACGCGCGCCACGACAAUCGGUGGUUGACAAACCCUCGUGUCCCGUCGUGCGUGCCGCGUUGCUCAUCGUGUGCUCUUUGACCCUGCUGUGUCGCGCUAGCGACGCUUCCAACGCG-

................(.((.....(((((((...((((.(((((((....))))).)).)))).....))))))).....((((..(((((.((....)).)))))..)))).....((((......((((((((((((((((((...(((.((((....)))).)))......)))))).))).)))))))))..))))..................(((((((....)))))))......)).)-

>KT308941 *Betula* *cylindrostachya* 5.8S ribosomal RNA gene and internal transcribed spacer 1, partial sequence; 5.8S ribosomal RNA gene, complete sequence; and internal transcribed spacer 2, partial sequence

ACGUCUGCCUGGGUGUCACGCAUCGUUGCCCCCAACCCCAUCUCCUUGCAAAGGGACGAGGGGGCCAAUGGGGCAGAAAUUGGCCUCCCGUGAGCUCAUGCAUGCGGUUGGCCUAAAAGCGAGUCCUCGGCGACGCGCGCCACGACAAUCGGUGGUUGACAAACCCUCGUGUCCCGUCGUGCGUGCCGCGUUGCUCAUCGUGUGCUCUUUGACCCUGYUGUGUCGCGCUAGCGACGCUUCCAACGCG-

................(.((.....(((((((...((((.(((((((....))))).)).)))).....))))))).....((((..(((((.((....)).)))))..)))).....((((......((((((((((((((((((...(((.((((....)))).)))......)))))).))).)))))))))..))))..................(((((((....)))))))......)).)-

>KT308940 *Betula* *alnoides* 5.8S ribosomal RNA gene and internal transcribed spacer 1, partial sequence; 5.8S ribosomal RNA gene, complete sequence; and internal transcribed spacer 2, partial sequence

ACGUCUGCCUGGGUGUCACGCAUCGUUGCCCCCAACCCCAUCUCCUUGCAAAGGGACGAGGGGGCCAGUGGGGCAGAAAUUGGCCUCCCGUGUGCUCAUGCGUGCGGUUGGCCUAAAAGCGAGUCCUCGGCGACGCGCGCCACGACAAUCGGUGGUUGACAAACCCUCGUGUCCCGUCGUGCGUGCCGUGUUGCUCAUCGUGUGCUCUUUGACCCUGCUGUGUCGCGCUAGCGAUGCUUCCAAUGCG-

................(.((.....((((((((..((((.(((((((....))))).)).))))...).))))))).....((((..(((((.((....)).)))))..)))).....((((......((((((((((((((((((...(((.((((....)))).)))......)))))).))).)))))))))..))))..................(((((((....)))))))......)).)-

>KT308939 *Betula* *luminifera* isolate 1 5.8S ribosomal RNA gene and internal transcribed spacer 1, partial sequence; 5.8S ribosomal RNA gene, complete sequence; and internal transcribed spacer 2, partial sequence

ACGUCUGCCUGGGUGUCACGCAUCGUUGCCCCCAACCCCAUCUCCUUUCAAAGGGACGAGGGGGCCAGUGGGGCAGAAAUUGGCCUCCCGUGAGCUCAUGCAUGCGGUUGGCCUAAAAGCGAGUCCUCGGCGACGCGCGCCACGACAAUCGGUGGUUGACAAACCCUCGUGUCCCGUCGUGCGUGCCGCGUUGCUCAUCGUGUGCUCUUUGACCCUGUUGUGUCGCGAUAGCGACGCUUCCAACGCG-

................(.((.....((((((((..((((.(((((((....))))).)).))))...).))))))).....((((..(((((.((....)).)))))..)))).....((((......((((((((((((((((((...(((.((((....)))).)))......)))))).))).)))))))))..))))..................(((((((....)))))))......)).)-

>KT308938 *Betula* *lenta* f. *uber* isolate 2 5.8S ribosomal RNA gene and internal transcribed spacer 1, partial sequence; 5.8S ribosomal RNA gene, complete sequence; and internal transcribed spacer 2, partial sequence

ACGUCUGCCUGGGUGUCACGCAUCGUUGCCCCCAACCCCAUCUCCUUGCAAAGGGACGAGGGGGCCUGUGGGGCAGAAAUUGGCCUCCCGUGAGCUCAUGCAUGCGGUUGGCCUAAAAGCGAGUCCUCGGCGACGCGCGCCACGACAAUCGGUGGUUGACAAACCCUCGUGUCCCGUCGUGCGUGCCGCGUCGCUCAUCGUGUGCUCUUUGACCCUGCUGUGUCGCGCUAGCGACGCUUCCAACGCG-

................(.((.....(((((((((.((((.(((((((....))))).)).))))..)).))))))).....((((..(((((.((....)).)))))..)))).....((((......((((((((((((((((((...(((.((((....)))).)))......)))))).))).)))))))))..))))..................(((((((....)))))))......)).)-

>KT308937 *Betula* *lenta* f. *uber* isolate 1 5.8S ribosomal RNA gene and internal transcribed spacer 1, partial sequence; 5.8S ribosomal RNA gene, complete sequence; and internal transcribed spacer 2, partial sequence

ACGUCUGCCUGGGUGUCACGCAUCGUUGCCCCCAACCCCAUCUCCUUGCAAAGGGACGAGGGGGCCUGUGGGGCAGAAAUUGGCCUCCCGUGAGCUCAUGCAUGCGGUUGGCCUAAAAGCGAGUCCUCGGCGACGCGCGCCACGACAAUCGGUGGUUGACAAACCCUCGUGUCCCGUCGUGCGUGCCGCGUCGCUCAUCGUGUGCUCUUUGACCCUGCUGUGUCGCGCUAGCGACGCUUCCAACGCG-

................(.((.....(((((((((.((((.(((((((....))))).)).))))..)).))))))).....((((..(((((.((....)).)))))..)))).....((((......((((((((((((((((((...(((.((((....)))).)))......)))))).))).)))))))))..))))..................(((((((....)))))))......)).)-

>KT308936 *Betula* *lenta* 5.8S ribosomal RNA gene and internal transcribed spacer 1, partial sequence; 5.8S ribosomal RNA gene, complete sequence; and internal transcribed spacer 2, partial sequence

ACGUCUGCCUGGGUGUCACGCAUCGUUGCCCCCAACCCCAUCUCCUUGCAAAGGGACGAGGGGGCCUGUGGGGCAGAAAUUGGCCUCCCGUGAGCUCAUGCAUGCGGUUGGCCUAAAAGCGAGUCCUCGGCGACGCGCGCCACGACAAUCGGUGGUUGACAAACCCUCGUGUCCCGUCGUGCGUGCCGCGUCGCUCAUCGUGUGCUCUUUGACCCUGCUGUGUCGCGCUAGCGACGCUUCCAACGCG-

................(.((.....(((((((((.((((.(((((((....))))).)).))))..)).))))))).....((((..(((((.((....)).)))))..)))).....((((......((((((((((((((((((...(((.((((....)))).)))......)))))).))).)))))))))..))))..................(((((((....)))))))......)).)-

>KT308935 *Betula* *grossa* isolate 2 5.8S ribosomal RNA gene and internal transcribed spacer 1, partial sequence; 5.8S ribosomal RNA gene, complete sequence; and internal transcribed spacer 2, partial sequence

ACGUCUGCCUGGGUGUCACGCAUCGUUGCCCCCAACCCCAUCUCCUUGCAAAGGGACGAGGGGGCCUGUGGGGCAGAAAUUGGCCUCCCGUGAGCUCAUGCAUGCGGUUGGCCUAAAAGCGAGUCCUCGGCGACGCGCGCCACGACAAUCGGUGGUUGUCAAACCCUCGUGUCCCGUCGUGCGUGCCGCGUCGCUCAUCGUGUGCUCCUUGACCCUGCUGUGUCGCGCUAGCGACGCUUCCAACGCG-

................(.((.....(((((((((.((((.(((((((....))))).)).))))..)).))))))).....((((..(((((.((....)).)))))..)))).....((((......((((((((((((((((((...(((.((((....)))).)))......)))))).))).)))))))))..))))..................(((((((....)))))))......)).)-

>KT308934 *Betula* *grossa* isolate 1 5.8S ribosomal RNA gene and internal transcribed spacer 1, partial sequence; 5.8S ribosomal RNA gene, complete sequence; and internal transcribed spacer 2, partial sequence

ACGUCUGCCUGGGUGUCACGCAUCGUUGCCCCCAACCCCAUCUCCUUGCAAAGGGACGAGGGGGCCUGUGGGGCAGAAAUUGGCCUCCCGUGAGCUCAUGCAUGCGGUUGGCCUAAAAGCGAGUCCUCGGCGACGCGCGCCACGACAAUCGGUGGUUGUCAAACCCUCGUGUCCCGUCGUGCGUGCCGCGUCGCUCAUCGUGUGCUCCUUGACCCUGCUGUGUCGCGCUAGCGACGCUUCCAACGCG-

................(.((.....(((((((((.((((.(((((((....))))).)).))))..)).))))))).....((((..(((((.((....)).)))))..)))).....((((......((((((((((((((((((...(((.((((....)))).)))......)))))).))).)))))))))..))))..................(((((((....)))))))......)).)-

>KT308933 *Betula* *megrelica* isolate 2 5.8S ribosomal RNA gene and internal transcribed spacer 1, partial sequence; 5.8S ribosomal RNA gene, complete sequence; and internal transcribed spacer 2, partial sequence

ACGUCUGCCUGGGUGUCACGCAUCGUUGCCCCCAACCCCAUCUCCUUGCAAAGGGACGAGGGGGCCUGUGGGGCAGAAAUUGGCCUCCCGUGAGCUCAUGCAUGCGGUUGGCCUAAAAGCGAGUCCUCGGCGACGCGCGCCACGACAAUCGGUGGUUGACAAACCCUCGUGUCCCGUCGUGCGUGCCGCGUCGCUCAUCGUGUGCUCUUUGACCCUGCUGUGUCGCGCUAGCGACGCUUCCAACGCG-

................(.((.....(((((((((.((((.(((((((....))))).)).))))..)).))))))).....((((..(((((.((....)).)))))..)))).....((((......((((((((((((((((((...(((.((((....)))).)))......)))))).))).)))))))))..))))..................(((((((....)))))))......)).)-

>KT308932 *Betula* *megrelica* isolate 1 5.8S ribosomal RNA gene and internal transcribed spacer 1, partial sequence; 5.8S ribosomal RNA gene, complete sequence; and internal transcribed spacer 2, partial sequence

ACGUCUGCCUGGGUGUCACGCAUCGUUGCCCCCAACCCCAUCUCCUUGCAAAGGGACGAGGGGGCCUGUGGGGCAGAAAUUGGCCUCCCGUGAGCUCAUGCAUGCGGUUGGCCUAAAAGCGAGUCCUCGGCGACGCGCGCCACGACAAUCGGUGGUUGACAAACCCUCGUGUCCCGUCGUGCGUGCCGCGUCGCUCAUCGUGUGCUCUUUGACCCUGCUGUGUCGCGCUAGCGACGCUUCCAACGCG-

................(.((.....(((((((((.((((.(((((((....))))).)).))))..)).))))))).....((((..(((((.((....)).)))))..)))).....((((......((((((((((((((((((...(((.((((....)))).)))......)))))).))).)))))))))..))))..................(((((((....)))))))......)).)-

>KT308931 *Betula* *medwediewii* isolate 2 5.8S ribosomal RNA gene and internal transcribed spacer 1, partial sequence; 5.8S ribosomal RNA gene, complete sequence; and internal transcribed spacer 2, partial sequence

ACGUCUGCCUGGGUGUCACGCAUCGUUGCCCCCAACCCCAUCUCCUUGCAAAGGGACGAGGGGGCCUGUGGGGCAGAAAUUGGCCUCCCGUGAGCUCAUGCAUGCGGUUGGCCUAAAAGCGAGUCCUCGGCGACGCGCGCCACGACAAUCGGUGGUUGACAAACCCUCGUGUCCCGUCGUGCGUGCCGCGUCGCUCAUCGUGUGCUCUUUGACCCUGCUGUGUCGCGCUAGCGACGCUUCCAACGCG-

................(.((.....(((((((((.((((.(((((((....))))).)).))))..)).))))))).....((((..(((((.((....)).)))))..)))).....((((......((((((((((((((((((...(((.((((....)))).)))......)))))).))).)))))))))..))))..................(((((((....)))))))......)).)-

>KT308930 *Betula* *medwediewii* isolate 1 5.8S ribosomal RNA gene and internal transcribed spacer 1, partial sequence; 5.8S ribosomal RNA gene, complete sequence; and internal transcribed spacer 2, partial sequence

ACGUCUGCCUGGGUGUCACGCAUCGUUGCCCCCAACCCCAUCUCCUUGCAAAGGGACGAGGGGGCCUGUGGGGCAGAAAUUGGCCUCCCGUGAGCUCAUGCAUGCGGUUGGCCUAAAAGCGAGUCCUCGGCGACGCGCGCCACGACAAUCGGUGGUUGACAAACCCUCGUGUCCCGUCGUGCGUGCCGCGUCGCUCAUCGUGUGCUCUUUGACCCUGCUGUGUCGCGCUAGCGACGCUUCCAACGCG-

................(.((.....(((((((((.((((.(((((((....))))).)).))))..)).))))))).....((((..(((((.((....)).)))))..)))).....((((......((((((((((((((((((...(((.((((....)))).)))......)))))).))).)))))))))..))))..................(((((((....)))))))......)).)-

>KT308929 *Betula* *insignis* subsp. *fansipanensis* 5.8S ribosomal RNA gene and internal transcribed spacer 1, partial sequence; 5.8S ribosomal RNA gene, complete sequence; and internal transcribed spacer 2, partial sequence

ACGUCUGCCUGGGUGUCACGCAUCGUUGCCCCCAACCCCAUCUCCUUGCAAAGGGAUGAGGGGGCCUGUGGGGCAGAAAUUGGCCUCCCGUGAGCUCAUGCAUGCGGUUGGCCUAAAAGCGAGUCCUCGGCGACGCGCGCCACGACAAUCGGUGGUUGACAAACCCUCGUGUCCCGUCGUGCGUGCCGCGUCGCUCAUCGGGUGCUCUUUGACCCUGCUGUGUCGCUCUAGCGACGCUUCCAACGCG-

................(.((.....(((((((((.((((.(((((((....))))).)).))))..)).))))))).....((((..(((((.((....)).)))))..))))......(((......((((((((((((((((((...(((.((((....)))).)))......)))))).))).)))))))))..)))...................(((((((....)))))))......)).)-

>KT308928 *Betula* *insignis* isolate 2 5.8S ribosomal RNA gene and internal transcribed spacer 1, partial sequence; 5.8S ribosomal RNA gene, complete sequence; and internal transcribed spacer 2, partial sequence

ACGUCUGCCUGGGUGUCACGCAUCGUUGCCCCCAACCCCAUCUCCUUGCAAAGGGAUGAGGGGGCCUGUGGGGCAGAAAUUGGCCUCCCGUGAGCUCAUGCAUGCGGUUGGCCUAAAAGCGAGUCCUCGGCGACGCGCGCCACGACAAUCGGUGGUUGACAAACCCUCGUGUCCCGUCGUGCGUGCCGCGUCGCUCAUCGGGUGCUCUUUGACCCUGCUGUGUCGCUCUAGCGACGCUUCCAACGCG-

................(.((.....(((((((((.((((.(((((((....))))).)).))))..)).))))))).....((((..(((((.((....)).)))))..))))......(((......((((((((((((((((((...(((.((((....)))).)))......)))))).))).)))))))))..)))...................(((((((....)))))))......)).)-

>KT308927 *Betula* *insignis* isolate 1 5.8S ribosomal RNA gene and internal transcribed spacer 1, partial sequence; 5.8S ribosomal RNA gene, complete sequence; and internal transcribed spacer 2, partial sequence

ACGUCUGCCUGGGUGUCACGCAUCGUUGCCCCCAACCCCNUCUCCUUGCAAAGGGAUGAGGGGGCCUGUGGGGCAGAAAUUGGCCUCCCGUGAGCUCAUGCAUGCGGUUGGCCUAAAAGCGAGUCCUCGGCGACGCGCGCCACGACAAUCGGUGGUUGACAAACCCUCGUGUCCCGUCGUGCGUGCCGCGUCGCUCAUCGNGUGCUCUUUGACCCUGCUGUGUCGCUCUAGCGACGCUUCCAACGCG-

................(.((.....(((((((((.((((.(((((((....))))).)).))))..)).))))))).....((((..(((((.((....)).)))))..)))).....((((......((((((((((((((((((...(((.((((....)))).)))......)))))).))).)))))))))..))))..................(((((((....)))))))......)).)-

>KT308926 *Betula* *murrayana* 5.8S ribosomal RNA gene and internal transcribed spacer 1, partial sequence; 5.8S ribosomal RNA gene, complete sequence; and internal transcribed spacer 2, partial sequence

ACGUCUGCCUGGGUGUCACGCAUCGUUGCCCCCAACCCCAUCUCCUUGCAAAGGGACGAGGGGGCCUGUGGGGCAGAAAUUGGCCUCCCGUGAGCUCAUGCAUGCGGUUGGCCUAAAAGCGAGUCCUCGGCGACGCGCGCCACGACAAUCGGUGGUUGACAAACCCUCGUGUCCCGUCGUGCGUGCCGCGUCGCCCAUCGUGUGCUCUUUGACCCUGCUGUGUCGCGCCAGCGACGCUUCCAACGCG-

................(.((.....(((((((((.((((.(((((((....))))).)).))))..)).))))))).....((((..(((((.((....)).)))))..)))).....((((......((((((((((((((((((...(((.((((....)))).)))......)))))).))).)))))))))..))))..................(((((((....)))))))......)).)-

>KT308925 *Betula* *alleghaniensis* 5.8S ribosomal RNA gene and internal transcribed spacer 1, partial sequence; 5.8S ribosomal RNA gene, complete sequence; and internal transcribed spacer 2, partial sequence

ACGUCUGCCUGGGUGUCACGCAUCGUUGCCCCCAACCCCAUCUCCUUGCAAAGGGACGAGGGGGCCUGUGGGGCAGAAAUUGGCCUCCCGUGAGCUCAUGCAUGCGGUUGGCCUAAAAGCGAGUCCUCGGCGACGCGCGCCACGACAAUCGGUGGUUGACAAACCCUCGUGUCCCGUCGUGCGUGCCGCGUCGCUCAUCGUGUGCUCUUUGACCCUGCUGUGUCGCGCCAGCGACGCUUCCAACGCG-

................(.((.....(((((((((.((((.(((((((....))))).)).))))..)).))))))).....((((..(((((.((....)).)))))..)))).....((((......((((((((((((((((((...(((.((((....)))).)))......)))))).))).)))))))))..))))..................(((((((....)))))))......)).)-

>KT308924 *Betula* *albosinensis* isolate 1 5.8S ribosomal RNA gene and internal transcribed spacer 1, partial sequence; 5.8S ribosomal RNA gene, complete sequence; and internal transcribed spacer 2, partial sequence

ACGUCUGCCUGGGUGUCACGCAUCGUUGCCCCCAACCCCAUCUCCUUGCAAAGGGACGAGGGGGCCUGUGGGGCAGAAAUUGGCCUCCCGUGAGCUCAUGCAUGCGGUUGGCCUAAAAGCGAGUCCUCGGCGACGCGCGCCACGACAAUCGGUGGUUGWCAAACCCUCGUGUCCCGUCGUGCGUGCCGCGUCGCUCAUCGUGUGCUCNUUGACCCUGCUGUGUCGCGCUAGCGACGCUUCCAACGCG-

................(.((.....(((((((((.((((.(((((((....))))).)).))))..)).))))))).....((((..(((((.((....)).)))))..)))).....((((......((((((((((((((((((...(((.((((....)))).)))......)))))).))).)))))))))..))))..................(((((((....)))))))......)).)-

>KT308923 *Betula* *utilis* var. *occidentalis* isolate 1 5.8S ribosomal RNA gene and internal transcribed spacer 1, partial sequence; 5.8S ribosomal RNA gene, complete sequence; and internal transcribed spacer 2, partial sequence

ACGUCUGCCUGGGUGUCACGCAUCGUUGCCCCCAACCCCAUCUCCUUGCAAAGGGACGAGGGGGCCUGUGGGGCAGAAAUUGGCCUCCCGUGAGCUCAUGCAUGCGGUUGGCCUAAAAGCGAGUCCUCGGCGACGCGCGCCACGACAAUCGGUGGUUGUCAAACCCUCGUGUCCCGUCGUGCGUGCCGCGUCGCUCAUCGUGUGCUCCUUGACCCUGCUGUGUCGCGCUAGCGACGCUUCCAACGCG-

................(.((.....(((((((((.((((.(((((((....))))).)).))))..)).))))))).....((((..(((((.((....)).)))))..)))).....((((......((((((((((((((((((...(((.((((....)))).)))......)))))).))).)))))))))..))))..................(((((((....)))))))......)).)-

>KT308922 *Betula* *delavayi* isolate 3 5.8S ribosomal RNA gene and internal transcribed spacer 1, partial sequence; 5.8S ribosomal RNA gene, complete sequence; and internal transcribed spacer 2, partial sequence

ACGUCUGCCUGGGUGUCACGCAUCGUUGCCCCCAACCCCAUCUCCUUKCAAAGGGACGAGGGGGCCAGUGGGGCAGAAAUUGGCCUCCCGUGAGCUCAUGCAUGCGGUUGGCCUAAAAGCGAGUCCUCGGCGACGCGCGCCACGACAAUCGGUGGUUGACAAACCCUCGUGUCCCGUCGUGCGUGCCGCGUUGCUCAUCGUGUGCUCUUUGACCCUGUUGUGUCGCGAUAGCGACGCUUCCAACGCG-

................(.((.....((((((((..((((.(((((((....))))).)).))))...).))))))).....((((..(((((.((....)).)))))..)))).....((((......((((((((((((((((((...(((.((((....)))).)))......)))))).))).)))))))))..))))..................(((((((....)))))))......)).)-

>KT308921 *Betula* *delavayi* isolate 2 5.8S ribosomal RNA gene and internal transcribed spacer 1, partial sequence; 5.8S ribosomal RNA gene, complete sequence; and internal transcribed spacer 2, partial sequence

ACGUCUGCCUGGGUGUCACGCAUCGUUGCCCCCAACCCCAUCUCCUUGCAAAGGGACGAAGGGGCCUGUGGGGCAGAAAUUGGCCUCCCGUGAGCUUAUGCAUGCGGUUGGCCUAAAAGCGAGUCCUCGGCGACGCGUGCCACGACAAUCGGUGGUUGACAAACCCUCGUGUCCCGUCGUGCGUGCCGCGUCGCUCAUCGUGUGCUCUUUGACCCUGCUGUGUCGUGCUAGCGACGCUUCCAACGCG-

................(.((.....(((((((((.((((.(((((((....))))).)).))))..)).))))))).....((((..(((((.((....)).)))))..)))).....((((......((((((((((((((((((...(((.((((....)))).)))......)))))).))).)))))))))..))))..................(((((((....)))))))......)).)-

>KT308920 *Betula* *schmidtii* isolate 2 5.8S ribosomal RNA gene and internal transcribed spacer 1, partial sequence; 5.8S ribosomal RNA gene, complete sequence; and internal transcribed spacer 2, partial sequence

ACGUCUGCCUGGGUGUCACGCAUCGUUGCCCCCAACCCCAUCUCCUUGCAAAGGGACGAGGGGGCCUGUGGGGCAGAAAUUGGCCUCCCGUGAGCUUAUGCAUGCGGUUGGCCUAAAAGCGAGUCCUCGGCGACGCGCGCCACGACAAUCGGUGGUUGACAAACCCUCGUGUCCCGUCGUGCGUGCCGUGUCGCUCAUCUUGUGCUCUUUGACCCUGCUGUGUCGCGCUAGCGACGCUUCCAACGCG-

................(.((.....(((((((((.((((.(((((((....))))).)).))))..)).))))))).....((((..(((((.((....)).)))))..)))).....(.((......((((((((((((((((((...(((.((((....)))).)))......)))))).))).)))))))))..)).)..................(((((((....)))))))......)).)-

>KT308919 *Betula* *schmidtii* isolate 1 5.8S ribosomal RNA gene and internal transcribed spacer 1, partial sequence; 5.8S ribosomal RNA gene, complete sequence; and internal transcribed spacer 2, partial sequence

ACGUCUGCCUGGGUGUCACGCAUCGUUGCCCCCAACCCCAUCUCCUUGCAAAGGGACGAGGGGGCCUGUGGGGCAGAAAUUGGCCUCCCGUGAGCUUAUGCAUGCGGUUGGCCUAAAAGCGAGUCCUCGGCGACGCGCGCCACGACAAUCGGUGGUUGACAAACCCUCGUGUCCCGUCGUGCGUGCCGUGUCGCUCAUCUUGUGCUCUUUGACCCUGCUGUGUCGCGCUAGCGACGCUUCCAACGCG-

................(.((.....(((((((((.((((.(((((((....))))).)).))))..)).))))))).....((((..(((((.((....)).)))))..)))).....(.((......((((((((((((((((((...(((.((((....)))).)))......)))))).))).)))))))))..)).)..................(((((((....)))))))......)).)-

>KT308918 *Betula* *chinensis* isolate 2 5.8S ribosomal RNA gene and internal transcribed spacer 1, partial sequence; 5.8S ribosomal RNA gene, complete sequence; and internal transcribed spacer 2, partial sequence

ACGUCUGCCUGGGUGUCACGCAUCGUUGCCCCCAACCCCAUCUCCUUGCAAAGGGACGAGGGG-CUUGUGGGGCAGAAAUUGGCCUCCCGUGAGCUUACGCAUGCGGUUGGCCUAAAAGCGAGUCCUCGGCGACGCGCGCCACGACAAUCGGUGGUUGACAAACCCUCGUGUCCCGUCGUGCGUGCCGCGUCGCUCAUCGUGUGCUCUUUGACCCUGCUGCGUCGCGCUAGCGACGCUUCCAACGCG-

................(.((.....(((((((((..(((.(((((((....))))).)).)))-..)).))))))).....((((..(((((.((....)).)))))..)))).....((((......((((((((((((((((((...(((.((((....)))).)))......)))))).))).)))))))))..))))..................(((((((....)))))))......)).)-

>KT308917 *Betula* *chinensis* isolate 1 5.8S ribosomal RNA gene and internal transcribed spacer 1, partial sequence; 5.8S ribosomal RNA gene, complete sequence; and internal transcribed spacer 2, partial sequence

ACGUCUGCCUGGGUGUCACGCAUCGUUGCCCCCAACCCCAUCUCCUUGCAAAGGGACGAGGGG-CUUGUGGGGCAGAAAUUGGCCUCCCGUGAGCUUACGCAUGCGGUUGGCCUAAAAGCGAGUCCUCGGCGACGCGCGCCACGACAAUCGGUGGUUGACAAACCCUCGUGUCCCGUCGUGCGUGCCGCGUCGCUCAUCGUGUGCUCUUUGACCCUGCUGCGUCGCGCUAGCGACGCUUCCAACGCG-

................(.((.....(((((((((..(((.(((((((....))))).)).)))-..)).))))))).....((((..(((((.((....)).)))))..)))).....((((......((((((((((((((((((...(((.((((....)))).)))......)))))).))).)))))))))..))))..................(((((((....)))))))......)).)-

>KT308916 *Betula* *chichibuensis* isolate 2 5.8S ribosomal RNA gene and internal transcribed spacer 1, partial sequence; 5.8S ribosomal RNA gene, complete sequence; and internal transcribed spacer 2, partial sequence

ACGUCUGCCUGGGUGUCACGCAUCGUUGCCCCCAACCCCAUCUCCUUGCAAAGGGACGAAGGGGCCUGUGGGGCAGAAAUUGGCCUCCCGUGAGCUUAUGCAUGCGGUUGGCCUAAAAGUGAGUCCUCGGCGACGCGCGCCACGACAAUCGGUGGUUGACAAACCCUCGUGUCCCGUCGUGCGUGCCGCGUCGCUCAUCGUGUGCUCUUUGACCCUACUGUGUCGUGCUAGCGACGCUUCCAACGCG-

................(.((.....(((((((((.((((.(((((((....))))).)).))))..)).))))))).....((((..(((((.((....)).)))))..)))).....((((......((((((((((((((((((...(((.((((....)))).)))......)))))).))).)))))))))..))))..................(((((((....)))))))......)).)-

>KT308915 *Betula* *chichibuensis* isolate 1 5.8S ribosomal RNA gene and internal transcribed spacer 1, partial sequence; 5.8S ribosomal RNA gene, complete sequence; and internal transcribed spacer 2, partial sequence

ACGUCUGCCUGGGUGUCACGCAUCGUUGCCCCCAACCCCAUCUCCUUGCAAAGGGACGAAGGGGCCUGUGGGGCAGAAAUUGGCCUCCCGUGAGCUUAUGCAUGCGGUUGGCCUAAAAGUGAGUCCUCGGCGACGCGCGCCACGACAAUCGGUGGUUGACAAACCCUCGUGUCCCGUCGUGCGUGCCGCGUCGCUCAUCGUGUGCUCUUUGACCCUACUGUGUCGUGCUAGCGACGCUUCCAACGCG-

................(.((.....(((((((((.((((.(((((((....))))).)).))))..)).))))))).....((((..(((((.((....)).)))))..)))).....((((......((((((((((((((((((...(((.((((....)))).)))......)))))).))).)))))))))..))))..................(((((((....)))))))......)).)-

>KT308914 *Betula* *calcicola* 5.8S ribosomal RNA gene and internal transcribed spacer 1, partial sequence; 5.8S ribosomal RNA gene, complete sequence; and internal transcribed spacer 2, partial sequence

ACGUCUGCCUGGGUGUCACGCAUCGUUGCCCCCAACCCCAUCUCCUUGCAAAGGGACGAAGGGGCCUGUGGGGCAGAAAUUGGCCUCCCGUGAGCUUAUGCAUGCGGUUGGCCUAAAAGCGAGUCCUCGGCGACGCGUGCCACGACAAUCGGUGGUUGACAAACCCUCGUGUCCCGUCGUGCGUGCCGCGUCGCUCAUCGUGUGCUCUUUGACCCUGCUGUGUCGUGCUAGCGACGCUUCCAACGCG-

................(.((.....(((((((((.((((.(((((((....))))).)).))))..)).))))))).....((((..(((((.((....)).)))))..)))).....((((......((((((((((((((((((...(((.((((....)))).)))......)))))).))).)))))))))..))))..................(((((((....)))))))......)).)-

>KT308913 *Betula* *delavayi* isolate 1 5.8S ribosomal RNA gene and internal transcribed spacer 1, partial sequence; 5.8S ribosomal RNA gene, complete sequence; and internal transcribed spacer 2, partial sequence

ACGUCUGCCUGGGUGUCACGCAUCGUUGCCCCCAACCCCAUCUCCUUGCAAAGGGACGAAGGGGCCUGUGGGGCAGAAAUUGGCCUCCCGUGAGCUUAUGCAUGCGGUUGGCCUAAAAGCGAGUCCUCGGCGACGCGUGCCACGACAAUCGGUGGUUGACAAACCCUCGUGUCCCGUCGUGCGUGCCGCGUCGCUCAUCGUGUGCUCUUUGACCCUGCUGUGUCGUGCUAGCGACGCUUCCAACGCG-

................(.((.....(((((((((.((((.(((((((....))))).)).))))..)).))))))).....((((..(((((.((....)).)))))..)))).....((((......((((((((((((((((((...(((.((((....)))).)))......)))))).))).)))))))))..))))..................(((((((....)))))))......)).)-

>KT308912 *Betula* *bomiensis* isolate 2 5.8S ribosomal RNA gene and internal transcribed spacer 1, partial sequence; 5.8S ribosomal RNA gene, complete sequence; and internal transcribed spacer 2, partial sequence

ACGUCUGCCUGGGUGUCACGCAUCGUUGCCCCCAACCCCAUCUCCUUGCAAAGGGACGAGGGGGCCAGUGGGGCAGAAAUUGGCCUCCCGUGAGCUCAUGCAUGCGGUUGGCCUAAAAGCGAGUCCUCGGCGACGCGCGCCACGACAAUCGGUGGUUGACAAACCCUCGUGUCCCGUCGUGCGUGCCGCGUUGCUCAUCGUGUGCUCUUUGACCCUGCUGCGUCGCGCUAGCGACGCUUCCAACGCG-

................(.((.....((((((((..((((.(((((((....))))).)).))))...).))))))).....((((..(((((.((....)).)))))..)))).....((((......((((((((((((((((((...(((.((((....)))).)))......)))))).))).)))))))))..))))..................(((((((....)))))))......)).)-

>KT308911 *Betula* *bomiensis* isolate 1 5.8S ribosomal RNA gene and internal transcribed spacer 1, partial sequence; 5.8S ribosomal RNA gene, complete sequence; and internal transcribed spacer 2, partial sequence

ACGUCUGCCUGGGUGUCACGCAUCGUUGCCCCCAACCCCAUCUCCUUGCAAAGGGACGAGGGGGCCAGUGGGGCAGAAAUUGGCCUCCCGUGAGCUCAUGCAUGCGGUUGGCCUAAAAGCGAGUCCUCGGCGACGCGCGCCACGACAAUCGGUGGUUGACAAACCCUCGUGUCCCGUCGUGCGUGCCGCGUUGCUCAUCGUGUGCUCUUUGACCCUGCUGCGUCGCGCUAGCGACGCUUCCAACGCG-

................(.((.....((((((((..((((.(((((((....))))).)).))))...).))))))).....((((..(((((.((....)).)))))..)))).....((((......((((((((((((((((((...(((.((((....)))).)))......)))))).))).)))))))))..))))..................(((((((....)))))))......)).)-

>KT308910 *Betula* *potaninii* isolate 2 5.8S ribosomal RNA gene and internal transcribed spacer 1, partial sequence; 5.8S ribosomal RNA gene, complete sequence; and internal transcribed spacer 2, partial sequence

ACGUCUGCCUGGGUGUCACGCAUCGUUGCCCCCAACCCCAUCUCCUUGCAAAGGGACGAAGGGGCCUGUGGGGCAGAAAUUGGCCUCCCGUGAGCUUAUGCAUGCGGUUGGCCUAAAAGCGAGUCCUCGGCGACGCGUGCCACGACAAUCGGUGGUUGACAAACCCUCGUGUCCCGUCGUGCGUGCCGCGUCGCUCAUCGUGUGCUCUUUGACCCUGCUGUGUCGUGCUAGCGACGCUUCCAACGCG-

................(.((.....(((((((((.((((.(((((((....))))).)).))))..)).))))))).....((((..(((((.((....)).)))))..)))).....((((......((((((((((((((((((...(((.((((....)))).)))......)))))).))).)))))))))..))))..................(((((((....)))))))......)).)-

>KT308909 *Betula* *potaninii* isolate 1 5.8S ribosomal RNA gene and internal transcribed spacer 1, partial sequence; 5.8S ribosomal RNA gene, complete sequence; and internal transcribed spacer 2, partial sequence

ACGUCUGCCUGGGUGUCACGCAUCGUUGCCCCCAACCCCAUCUCCUUGCAAAGGGACGAAGGGGCCUGUGGGGCAGAAAUUGGCCUCCCGUGAGCUUAUGCAUGCGGUUGGCCUAAAAGCGAGUCCUCGGCGACGCGUGCCACGACAAUCGGUGGUUGACAAACCCUCGUGUCCCGUCGUGCGUGCCGCGUCGCUCAUCGUGUGCUCUUUGACCCUGCUGUGUCGUGCUAGCGACGCUUCCAACGCG-

................(.((.....(((((((((.((((.(((((((....))))).)).))))..)).))))))).....((((..(((((.((....)).)))))..)))).....((((......((((((((((((((((((...(((.((((....)))).)))......)))))).))).)))))))))..))))..................(((((((....)))))))......)).)-

>KT308908 *Betula* *corylifolia* isolate 2 5.8S ribosomal RNA gene and internal transcribed spacer 1, partial sequence; 5.8S ribosomal RNA gene, complete sequence; and internal transcribed spacer 2, partial sequence

ACGUCUGCCUGGGUGUCACGCAUCGUUGCCCCCAACCCCAUCUCCUUGCAAAGGGACGAGGGG-CUUGUGGGGCAGAAAUUGGCCUCCCGUGAGCUUAUGCAUGCGGUUGGCCUAAAAGCGAGUCCUCGGCGACGCGCGCCACGACAAUCGGUGGUUGACAAACCCUCGUGUCCCGUCGUGUGUGCCGCGUCGCUCAUCGUGUGCUCUUUGACCCUGCUGUGUCGCGCUAGCGACGCUUCCAACGCG-

................(.((.....(((((((((..(((.(((((((....))))).)).)))-..)).))))))).....((((..(((((.((....)).)))))..)))).....((((......((((((((((((((((((...(((.((((....)))).)))......)))))).))).)))))))))..))))..................(((((((....)))))))......)).)-

>KT308907 *Betula* *corylifolia* isolate 1 5.8S ribosomal RNA gene and internal transcribed spacer 1, partial sequence; 5.8S ribosomal RNA gene, complete sequence; and internal transcribed spacer 2, partial sequence

ACGUCUGCCUGGGUGUCACGCAUCGUUGCCCCCAACCCCAUCUCCUUGCAAAGGGACGAGGGG-CUUGUGGGGCAGAAAUUGGCCUCCCGUGAGCUUAUGCAUGCGGUUGGCCUAAAAGCGAGUCCUCGGCGACGCGCGCCACGACAAUCGGUGGUUGACAAACCCUCGUGUCCCGUCGUGUGUGCCGCGUCGCUCAUCGUGUGCUCUUUGACCCUGCUGUGUCGCGCUAGCGACGCUUCCAACGCG-

................(.((.....(((((((((..(((.(((((((....))))).)).)))-..)).))))))).....((((..(((((.((....)).)))))..)))).....((((......((((((((((((((((((...(((.((((....)))).)))......)))))).))).)))))))))..))))..................(((((((....)))))))......)).)-

>KT308906 *Betula* *fargesii* 5.8S ribosomal RNA gene and internal transcribed spacer 1, partial sequence; 5.8S ribosomal RNA gene, complete sequence; and internal transcribed spacer 2, partial sequence

ACGUCUGCCUGGGUGUCACGCAUCGUUGCCCCCAACCCCAUCUCCUUGCAAAGGGACGAGGGG-CUUGUGGGGCAGAAAUUGGCCUCCCGUGAGCUUACGCAUGCGGUUGGCCUAAAAGCGAGUCCUCGGCGACGCGCGCCACGACAAUCGGUGGUUGACAAACCCUCGUGUCCCGUCGUGCGUGCCGCGUCGCUCAUCGUGUGCUCUUUGACCCUGCUGCGUCGCGCUAGCGACGCUUCCAACGCG-

................(.((.....(((((((((..(((.(((((((....))))).)).)))-..)).))))))).....((((..(((((.((....)).)))))..)))).....((((......((((((((((((((((((...(((.((((....)))).)))......)))))).))).)))))))))..))))..................(((((((....)))))))......)).)-

>KT308905 *Betula* *globispica* isolate 2 5.8S ribosomal RNA gene and internal transcribed spacer 1, partial sequence; 5.8S ribosomal RNA gene, complete sequence; and internal transcribed spacer 2, partial sequence

ACGUCUGCCUGGGUGUCACGCAUCGUUGCCCCCAACCCCAUCUCCUUGCAAAGGGACGAGGGGGCUUGUGGGGCAGAAAUUGGCCUCCCGUGAGCUUACGCAUGCGGUUGGCCUAAAAGCGAGUCCUCGGCGACGCGCGCCACGACAAUCGGUGGUUGACAAACCCUCGUGUCCCGUCGUGCGUGCCGCGUCGCUCAUCGUGUGCUCUUUGACCCUGCUGCGUCGCGCUAGCGACGCUUCCAACGCG-

................(.((.....(((((((((.((((.(((((((....))))).)).))))..)).))))))).....((((..(((((.((....)).)))))..)))).....((((......((((((((((((((((((...(((.((((....)))).)))......)))))).))).)))))))))..))))..................(((((((....)))))))......)).)-

>KT308904 *Betula* *globispica* isolate 1 internal transcribed spacer 1, partial sequence; 5.8S ribosomal RNA gene, complete sequence; and internal transcribed spacer 2, partial sequence

ACGUCUGCCUGGGUGUCACGCAUCGUUGCCCCCAACCCCAUCUCCUUGCAAAGGGACGAGGGG-CUUGUGGGGCAGAAAUUGGCCUCCCGUGAGCUUACGCAUGCGGUUGGCCUAAAAGCGAGUCCUCGGCGACGCGCGCCACGACAAUCGGUGGUUGACAAACCCUCGUGUCCCGUCGUGCGUGCCGCGUCGCUCAUCGUGUGCUCUUUGACCCUGCUGCGUCGCGCUAGCGACGCUUCCAACGCG-

................(.((.....(((((((((..(((.(((((((....))))).)).)))-..)).))))))).....((((..(((((.((....)).)))))..)))).....((((......((((((((((((((((((...(((.((((....)))).)))......)))))).))).)))))))))..))))..................(((((((....)))))))......)).)-

>JN247411 *Betula* *pendula* voucher MCA 221 internal transcribed spacer 1, partial sequence; 5.8S ribosomal RNA gene, complete sequence; and internal transcribed spacer 2, partial sequence

ACGUCUGCCUGGGUGUCACGCAUCGUUGCCCCCAACCCCAUCUCCUUGCAAAGGGACGAGGGGGCCUGUGGGGCAGAAAUUGGCCUCCCGUGAGCUCAUGCAUGCGGUUGGCCUAAAAGCGAGUCCUCGGCGACGCGCGCCACGACAAUCGGUGGUUGUCAAACCCUCGUGUCCCGUCGUGCGUGCCGCGUCGCUCAUCGUGUGCUCCUUGACCCUGCUGUGUCGCGCUAGCGACGCUUCCAACGCGA

...............((.((.....(((((((((.((((.(((((((....))))).)).))))..)).))))))).....((((..(((((.((....)).)))))..)))).....((((......((((((((((((((((((...(((.((((....)))).)))......)))))).))).)))))))))..))))..................(((((((....)))))))......)).))

>FJ011780 *Betula* *utilis* voucher MacAtrher-Tibet Expedition 452 18S ribosomal RNA gene, partial sequence; internal transcribed spacer 1, 5.8S ribosomal RNA gene, and internal transcribed spacer 2, complete sequence; and 26S ribosomal RNA gene, partial sequence

ACGUCUGCCUGGGUGUCACGCAUCGUUGCCCCCAACCCCAUCUCCUUGCAAAGGGACGAGGGGGCCAGUGGGGCAGAAAUUGGCCUCCCGUGAGCUCAUGCAUGCGGUUGGCCUAAAAGCGAGUCCUCGGCGACGCGCGCCACGACAAUCGGUGGUUGACAAACCCUCGUGUCCCGUCGUGCGUGCCGCGUCGCUCAUCGUGUGCUCUUUGACCCUGUUGUGUCGCGCUAGCGACGCUUCCAACGCGA

...............((.((.....((((((((..((((.(((((((....))))).)).))))...).))))))).....((((..(((((.((....)).)))))..)))).....((((......((((((((((((((((((...(((.((((....)))).)))......)))))).))).)))))))))..))))..................(((((((....)))))))......)).))

>FJ011779 *Betula* *schmidtii* voucher Lee s.n. 18S ribosomal RNA gene, partial sequence; internal transcribed spacer 1, 5.8S ribosomal RNA gene, and internal transcribed spacer 2, complete sequence; and 26S ribosomal RNA gene, partial sequence

ACGUCUGCCUGGGUGUCACGCAUCGUUGCCCCCAACCCCAUCUCCUUGCAAAGGGACGAGGGGGCCUGUGGGGCAGAAAUUGGCCUCCCGUGAGCUUAUGCAUGCGGUUGGCCUAAAAGCGAGUCCUCGGCGACGCGCGCCACGACAAUCGGUGGUUGACAAACCCUCGUGUCCCGUCGUGCGUGCCGUGUCGCUCAUCUUGUGCUCUUUGACCCUGCUGUGUCGCGCUAGCGACGCUUCCAACGCGA

...............((.((.....(((((((((.((((.(((((((....))))).)).))))..)).))))))).....((((..(((((.((....)).)))))..)))).....(.((......((((((((((((((((((...(((.((((....)))).)))......)))))).))).)))))))))..)).)..................(((((((....)))))))......)).))

>FJ011778 *Betula* *platyphylla* voucher Lee s.n. 18S ribosomal RNA gene, partial sequence; internal transcribed spacer 1, 5.8S ribosomal RNA gene, and internal transcribed spacer 2, complete sequence; and 26S ribosomal RNA gene, partial sequence

ACGUCUGCCUGGGUGUCACGCAUCGUUGCCCCCAACCCCAUCUCCUUGCAAAGGGACGAGGGGGCCUGUGGGGCAGAAAUUGGCCUCCCGUGAGCUCAUGCAUGCGGUUGGCCUAAAAGCGAGUCCUCGGCGACGCGCGCCACGACAAUCGGUGGUUGUCAAACCCUCGUGUCCCGUCGUGCGUGCCGCGUCGCUCAUCGUGUGCUCCUUGACCCUGCUGUGUCGCGCUAGCGACGCUUCCAACGCGA

...............((.((.....(((((((((.((((.(((((((....))))).)).))))..)).))))))).....((((..(((((.((....)).)))))..)))).....((((......((((((((((((((((((...(((.((((....)))).)))......)))))).))).)))))))))..))))..................(((((((....)))))))......)).))

>FJ011777 *Betula* *pendula* voucher CS03022 18S ribosomal RNA gene, partial sequence; internal transcribed spacer 1, 5.8S ribosomal RNA gene, and internal transcribed spacer 2, complete sequence; and 26S ribosomal RNA gene, partial sequence

ACGUCUGCCUGGGUGUCACGCAUCGUUGCCCCCAACCCCAUCUCCUUGCAAAGGGACGAGGGGGCCUGUGGGGCAGAAAUUGGCCUCCCGUGAGCUCAUGCAUGCGGUUGGCCUAAAAGCGAGUCCUCGGCGACGCGCGCCACGACAAUCGGUGGUUGUCAAACCCUCGUGUCCCGUCGUGCGUGCCGCGUCGCUCAUCGUGUGCUCCUUGACCCUGUUGUGUCGCGCUAGCGACGCUUCCAACGCGA

...............((.((.....(((((((((.((((.(((((((....))))).)).))))..)).))))))).....((((..(((((.((....)).)))))..)))).....((((......((((((((((((((((((...(((.((((....)))).)))......)))))).))).)))))))))..))))..................(((((((....)))))))......)).))

>FJ011776 *Betula* *papyrifera* voucher CS78048 internal transcribed spacer 1, partial sequence; 5.8S ribosomal RNA gene and internal transcribed spacer 2, complete sequence; and 26S ribosomal RNA gene, partial sequence

ACGUCUGCGUGGGUGUCACGCAUCGUUGCCCCCAACCCCAUCUCCUUGUAAAGGGACGAGGGGGCCUGUGGGGCAGAAAUUGGCCUCCCGUGAGCUCAUGCAUGCGGUUGGCCUAAAAGCGAGUCCUCGGCGACGCGCGCCACGACAAUCGGUGGUUGUCAAACCCUCGUGUCCCGUCGUGCGUGACGCGUCGCUCAUCGUGUGCUCCUUGACCCUGCUGUGUCGCGCUAGCGACGCUUCCAACGCGA

...............((.((.....(((((((((.((((.(((((((....))))).)).))))..)).))))))).....((((..(((((.((....)).)))))..)))).....((((......((((((((((((((((((...(((.((((....)))).)))......)))))).))).)))))))))..))))..................(((((((....)))))))......)).))

>FJ011775 *Betula* *lenta* voucher CS89165 internal transcribed spacer 1, partial sequence; 5.8S ribosomal RNA gene and internal transcribed spacer 2, complete sequence; and 26S ribosomal RNA gene, partial sequence

ACGUCUGCCUGGGUGUCACGCAUCGUUGCCCCCAACCCCAUCUCCUUGCAAAGGGACGAGGGGGCCUGUGGGGCAGAAAUUGGCCUCCCGUGAGCUCAUGCAUGCGGUUGGCCUAAAAGCGAGUCCUCGGCGACGCGCGCCGCGACAAUCGGUGGUUGACAAACCCUCGUGUCCCGUCGUGCGUGCCGCGUCGCUCAUCGUGUGCUCUUUGACCCUGCUGUGUCGCGCCAGCGACGCUUCCAACGCGA

...............((.((.....(((((((((.((((.(((((((....))))).)).))))..)).))))))).....((((..(((((.((....)).)))))..)))).....((((......((((((((((((((((((...(((.((((....)))).)))......)))))).))).)))))))))..))))..................(((((((....)))))))......)).))

>FJ011774 *Betula* *glandulosa* voucher CS78027 internal transcribed spacer 1, partial sequence; 5.8S ribosomal RNA gene and internal transcribed spacer 2, complete sequence; and 26S ribosomal RNA gene, partial sequence

ACGUCUGCCUGGGUGUCACGCAUCGUUGCCCCCAACCCCAUCUCCUUGCAAAGGGACGAGGGGGCCUGUGGGGCAGAAAUUGGCCUCCCGUGAGCUCAUGCAUGCGGUUGGCCUAAAAGCGAGUCCUCGGCGACGCGCGCCACGACAAUCGGUGGUUGUCAAACCCUCGUGUCCCGUCGUGCGUGCCGCGUCGCUCAUCGUGUGCUCCUUGACCCUGCUGUGUCGCGCUAGCGACGCUUCCAACGCGA

...............((.((.....(((((((((.((((.(((((((....))))).)).))))..)).))))))).....((((..(((((.((....)).)))))..)))).....((((......((((((((((((((((((...(((.((((....)))).)))......)))))).))).)))))))))..))))..................(((((((....)))))))......)).))

>FJ011773 *Betula* *davurica* voucher Tibet218 18S ribosomal RNA gene, partial sequence; internal transcribed spacer 1, 5.8S ribosomal RNA gene, and internal transcribed spacer 2, complete sequence; and 26S ribosomal RNA gene, partial sequence

ACGUCUGCCUGGGUGUCACGCAUCGUUGCCCCCAACCCCAUCUCCUUGCAAAGGGACGAGGGGGCCAGUGGGGCAGAAAUUGGCCUCCCGUGAGCUCAUGCAUGCGGUUGGCCUAAAAGCGAGUCCUCGGCGACGCGCGCCACGACAAUCGGUGGUUGACAAACCCUCGUGUCCCGUCGUGCGUGCCGCGUCGCUCAUCGUGUGCUCUUUGACCCUGUUGUGUCGCGCUAGCGACGCUUCCAACGCGA

...............((.((.....((((((((..((((.(((((((....))))).)).))))...).))))))).....((((..(((((.((....)).)))))..)))).....((((......((((((((((((((((((...(((.((((....)))).)))......)))))).))).)))))))))..))))..................(((((((....)))))))......)).))

>FJ011772 *Betula* *davurica* voucher UNA63303V 18S ribosomal RNA gene, partial sequence; internal transcribed spacer 1, 5.8S ribosomal RNA gene, and internal transcribed spacer 2, complete sequence; and 26S ribosomal RNA gene, partial sequence

ACGUCUGCCUGGGUGUCACGCAUCGUUGCCCCCAACCCCAUCUCCUUGUAAAGGGACGAGGGGGCCCGUGGGGCAGAAAUUGGCCUCCCGUGAGCUCAUGCAUGCGGUUGGCCUAAAAGCGAGUCCUCGGCGACGCGCGCCACGACAAUCGGUGGUUGUCAAACCCUCGUGUCCCGUCGUGCGUGACGCGUCGCUCAUCGUGUGCUCCUUGACCCUGCUGUGUCGCGCUAGCGACGCUUCCAACGCGA

...............((.((.....((((((((..((((.(((((((....))))).)).))))...).))))))).....((((..(((((.((....)).)))))..)))).....((((......((((((((((((((((((...(((.((((....)))).)))......)))))).))).)))))))))..))))..................(((((((....)))))))......)).))

>FJ011771 *Betula* *davurica* voucher UNA64631H 18S ribosomal RNA gene, partial sequence; internal transcribed spacer 1, 5.8S ribosomal RNA gene, and internal transcribed spacer 2, complete sequence; and 26S ribosomal RNA gene, partial sequence

ACGUCUGCCUGGGUGUCACGCAUCGUUGCCCCCAACCCCAUCUCCUUGUAAAGGGACGAGGGGGCCCGUGGGGCAGAAAUUGGCCUCCCGUGAGCUCAUGCAUGCGGUUGGCCUAAAAGCGAGUCCUCGGCGACGCGCGCCACGACAAUCGGUGGUUGUCAAACCCUCGUGUCCCGUCGUGCGUGACGCGUCGCUCAUCGUGUGCUCCUUGACCCUGCUGUGUCGCGCUAGCGACGCUUCCAACGCGA

...............((.((.....((((((((..((((.(((((((....))))).)).))))...).))))))).....((((..(((((.((....)).)))))..)))).....((((......((((((((((((((((((...(((.((((....)))).)))......)))))).))).)))))))))..))))..................(((((((....)))))))......)).))

>FJ011770 *Betula* *davurica* voucher Lee s.n. 18S ribosomal RNA gene, partial sequence; internal transcribed spacer 1, 5.8S ribosomal RNA gene, and internal transcribed spacer 2, complete sequence; and 26S ribosomal RNA gene, partial sequence

ACGUCUGCCUGGGUGUCACGCAUCGUUGCCCCCAACCCCAUCUCCUUGUAAAGGGACGAGGGGGCCCGUGGGGCAGAAAUUGGCCUCCCGUGAGCUCAUGCAUGCGGUUGGCCUAAAAGCGAGUCCUCGGCGACGCGCGCCACGACAAUCGGUGGUUGUCAAACCCUCGUGUCCCCUCGUGCGUGACGCGUCGUUCAUCGUGUGCUCCUUGACCCUGCUGUGUCGCGCUAGCGACGCUUCCAACGCGA

...............((.((.....((((((((..((((.(((((((....))))).)).))))...).))))))).....((((..(((((.((....)).)))))..)))).....((((......(((((((((((((((((....(((.((((....)))).))).......))))).))).)))))))))..))))..................(((((((....)))))))......)).))

>FJ011769 *Betula* *alnoides* voucher Wen6405 internal transcribed spacer 1, partial sequence; 5.8S ribosomal RNA gene and internal transcribed spacer 2, complete sequence; and 26S ribosomal RNA gene, partial sequence

ACGUCUGCCUGGGUGUCACGCAUCGUUGCCCCCAACCCCAUCUCCUUGCAAAGGGACGAGGGGGCCAAUGGGGCAGAAAUUGGCCUCCCGUGAGCUCAUGCAUGCGGUUGGCCUAAAAGCGAGUCCUCGGCGACGCGCGCCACGACAAUCGGUGGUUGACAAACCCUCGUGUCCCGUCGUGCGUGCCGCGUUGCUCAUCGUGUGCUCUUUGACCCUGCUGUGUCGCGCUAGCGACGCUUCCAACGCGA

...............((.((.....(((((((...((((.(((((((....))))).)).)))).....))))))).....((((..(((((.((....)).)))))..)))).....((((......((((((((((((((((((...(((.((((....)))).)))......)))))).))).)))))))))..))))..................(((((((....)))))))......)).))

>AY763114 *Betula* *alnoides* isolate 3464 internal transcribed spacer 1, partial sequence; 5.8S ribosomal RNA gene, complete sequence; and internal transcribed spacer 2, partial sequence

ACGUCUGCCUGGGUGUCACGCAUCGUUGCCCCCAACCCCAUCUCCUUGCAAAGGGACGAGGGGGCCAGUGGGGCAGAAAUUGGCCUCCCGUGAGCUCAUGCAUGCGGUUGGCCUAAAAGCGAGUCCUCGGCGACGCGCGCCACGACAAUCGGUGGUUGACAAACCCUCGUGUCCCGUCGUGCGUGCCGCGUUGCUCAUCGUGUGCUCUUUGACCCUGYUGUGUCGCGCUAGCGAUGCUUCCAACGCG-

................(.((.....((((((((..((((.(((((((....))))).)).))))...).))))))).....((((..(((((.((....)).)))))..)))).....((((......((((((((((((((((((...(((.((((....)))).)))......)))))).))).)))))))))..))))..................(((((((....)))))))......)).)-

>AY763113 *Betula* *luminifera* isolate 2841 internal transcribed spacer 1, partial sequence; 5.8S ribosomal RNA gene, complete sequence; and internal transcribed spacer 2, partial sequence

ACGUCUGCCUGGGUGUCACGCAUCGUUGCCCCCAACCCCAUCUCCUUGCAAAGGGACGAGGGGGCCAAUGGGGCAGAAAUUGGCCUCCCGUGAGCUCAUGCAUGCGGUUGGCCUAAAAGCGAGUCCUCGGCGACGCGCGCCACGACAAUCGGUGGUUGACAAACCCUCGUGUCCCGUCGUGCGUGCCGCGUUGCUCAUCGUGUGCUCUUUGACCCUGCUGUGUCGCGCUAGCGACGCUUCCAACGCG-

................(.((.....(((((((...((((.(((((((....))))).)).)))).....))))))).....((((..(((((.((....)).)))))..)))).....((((......((((((((((((((((((...(((.((((....)))).)))......)))))).))).)))))))))..))))..................(((((((....)))))))......)).)-

>AY761134 *Betula* *utilis* isolate 2893 internal transcribed spacer 1, partial sequence; 5.8S ribosomal RNA gene, complete sequence; and internal transcribed spacer 2, partial sequence

ACGUCUGCCUGGGUGUCACGCAUCGUUGCCCCCAACCCCAUCUCCUUGCAAAGGGACGAGGGGGCCAGUGGGGCAGAAAUUGGCCUCCCGUGAGCUCAUGCAUGCGGUUGGCCUAAAAGCGAGUCCU-GGCGACGCGCGCCACGACAAUCGGUGGUUGACAAACCCUCGUGUCCCGUCGUGCGUGCCGCGUCGCUCAGCGUGUGCUCUUUGACCCUGCUGUGUCGCGCUAGCGACGCUUCCAACGCG-

................(.((.....((((((((..((((.(((((((....))))).)).))))...).))))))).....((((..(((((.((....)).)))))..)))).....(((......-((((((((((((((((((...(((.((((....)))).)))......)))))).))).)))))))))...)))..................(((((((....)))))))......)).)-

>AY761133 *Betula* *schmidtii* isolate 2875 internal transcribed spacer 1, partial sequence; 5.8S ribosomal RNA gene, complete sequence; and internal transcribed spacer 2, partial sequence

ACGUCUGCCUGGGUGUCACGCAUCGUUGCCCCCAACCCCAUCUCCUUGCAAAGGGACGAGGGGGCCUGUGGGGCAGAAAUUGGCCUCCCGUGAGCUUAUGCAUGCGGUUGGCCUAAAAGCGAGUCCUCGGCGACGCGCGCCACGACAAUCGGUGGUUGACAAACCCUCGUGUCCCGUCGUGCGUGCCGUGUCGCUCAUCUUGUGCUCUUUGACCCUGCUGUGUCGCGCUAGCGACGCUUCCAACGCG-

................(.((.....(((((((((.((((.(((((((....))))).)).))))..)).))))))).....((((..(((((.((....)).)))))..)))).....(.((......((((((((((((((((((...(((.((((....)))).)))......)))))).))).)))))))))..)).)..................(((((((....)))))))......)).)-

>AY761132 *Betula* *raddeana* isolate 3338 internal transcribed spacer 1, partial sequence; 5.8S ribosomal RNA gene, complete sequence; and internal transcribed spacer 2, partial sequence

ACGUCUGCCUGGGUGUCACGCAUCGUUGCCCCCAACCCCAUCUCCUUGAAAAGGGACGAGGGGGCCUGUGGGGCAGAAAUUGGCCUCCCGUGAGCUCAUGCAUGCGGUUGGCCUAAAAGCGAGUCCUCGGCGACGCGCGCCACGACAAUCGGUGGUUGUCAAACCCUCGUGUCCCGUCGUGCGUGACGCGUCGCUCAUCGUGUGCUCCUUGACCCUGCUGUGUCGCGCUAGCGACGCUUCCAACGCG-

................(.((.....(((((((((.((((.(((((((....))))).)).))))..)).))))))).....((((..(((((.((....)).)))))..)))).....((((......((((((((((((((((((...(((.((((....)))).)))......)))))).))).)))))))))..))))..................(((((((....)))))))......)).)-

>AY761131 *Betula* *pumila* isolate 3246 internal transcribed spacer 1, partial sequence; 5.8S ribosomal RNA gene, complete sequence; and internal transcribed spacer 2, partial sequence

ACGUCUGCCUGGGUGUCACGCAUCGUUGCCCCCAACCCCAUCUCCUUGAAAAGGGACGAGGGGGCCUGUGGGGCAGAAAUUGGCCUCCCGUGAGCUCAUGCAUGCGGUUGGCCUAAAAGCGAGUCCUCGGCGACGCGCGCCACGACAAUCGGUGGUUGUCAAACCCUCGUGUCCCGUCGUGCGUGACGCGUCGCUCAUCGUGUGCUCCUUGACCCUGCUGUGUCGCGCUAGCGACGCUUCCAACGCG-

................(.((.....(((((((((.((((.(((((((....))))).)).))))..)).))))))).....((((..(((((.((....)).)))))..)))).....((((......((((((((((((((((((...(((.((((....)))).)))......)))))).))).)))))))))..))))..................(((((((....)))))))......)).)-

>AY761130 *Betula* *pubescens* isolate 2895 internal transcribed spacer 1, partial sequence; 5.8S ribosomal RNA gene, complete sequence; and internal transcribed spacer 2, partial sequence

ACGUCUGCCUGGGUGUCACGCAUCGUUGCCCCCAACCCCAUCUCCUUGCAAAGGGACGAGGGGGCCUGUGGGGCAGAAAUUGGCCUCCCGUGAGCUCAUGCAUGCGGUUGGCCUAAAAGCGAGUCCU-GGCGACGCGCGCCACGACAAUCGGUGGUUGUCAAACCCUCGUGUCCCGUCGUGCGUGCCGCGUCGCUCAUCGUGUGCUCCUUGACCCUGCUGCGUCGCGCUAGCGACGCUUCCAACGCG-

................(.((.....(((((((((.((((.(((((((....))))).)).))))..)).))))))).....((((..(((((.((....)).)))))..)))).....((((.....-((((((((((((((((((...(((.((((....)))).)))......)))))).))).)))))))))..))))..................(((((((....)))))))......)).)-

>AY761129 *Betula* *populifolia* isolate 2890 internal transcribed spacer 1, partial sequence; 5.8S ribosomal RNA gene, complete sequence; and internal transcribed spacer 2, partial sequence

ACGUCUGCCUGGGUGUCACGCAUCGUUGCCCCCAACCCCAUCUCCUUGCAAAGGGACGAGGGGGCCUGUGGGGCAGAAAUUGGCCUCCCGUGAGCUCAUGCAUGCGGUUGGCCUAAAAGCGAGUCCU-GGCGACGCGCGCCACGACAAUCGGUGGUUGUCAAACCCUCGUGUCCCGUCGUGCGUGCCGCGUCGCUCAUCGUGUGCUCCUUGACCCUGCUGUGUCGCGCUAGCGACGCUUCCAACGCG-

................(.((.....(((((((((.((((.(((((((....))))).)).))))..)).))))))).....((((..(((((.((....)).)))))..)))).....((((.....-((((((((((((((((((...(((.((((....)))).)))......)))))).))).)))))))))..))))..................(((((((....)))))))......)).)-

>AY761128 *Betula* *platyphylla* isolate 2934 internal transcribed spacer 1, partial sequence; 5.8S ribosomal RNA gene, complete sequence; and internal transcribed spacer 2, partial sequence

ACGUCUGCCUGGGUGUCACGCAUCGUUGCCCCCAACCCCAUCUCCUUGCAAAGGGACGAGGGGGCCUGUGGGGCAGAAAUUGGCCUCCCGUGAGCUCAUGCAUGCGGUUGGCCUAAAAGCGAGUCCU-GGCGACGCGCGCCACGACAAUCGGUGGUUGUCAAACCCUCGUGUCCCGUCGUGCGUGCCGCGUCGCUCAUCGUGUGCUCCUUGACCCUGCUGUGUCGCGCUAGCGACGCUUCCAACGCG-

................(.((.....(((((((((.((((.(((((((....))))).)).))))..)).))))))).....((((..(((((.((....)).)))))..)))).....((((.....-((((((((((((((((((...(((.((((....)))).)))......)))))).))).)))))))))..))))..................(((((((....)))))))......)).)-

>AY761127 *Betula* *pendula* isolate 2902 internal transcribed spacer 1, partial sequence; 5.8S ribosomal RNA gene, complete sequence; and internal transcribed spacer 2, partial sequence

ACGUCUGCCUGGGUGUCACGCAUCGUUGCCCCCAACCCCAUCUCCUUGNAAAGGGACGAGGGGGCCUGUGGGGCAGAAAUUGGCCUCCCGUGAGCUCAUGCAUGCGGUUGGCCUAAAAGCGAGUCCU-GGCGACGCGCGCCACGACAAUCGGUGGUUGUCAAACCCUCGUGUCCCGUCGUGCGUGCCGCGUCGCUCAUCGUGUGCUCCUUGACCCUGCUGUGUCGCGCUAGCGACGCUUCCAACGCG-

................(.((.....(((((((((.((((.(((((((....))))).)).))))..)).))))))).....((((..(((((.((....)).)))))..)))).....((((.....-((((((((((((((((((...(((.((((....)))).)))......)))))).))).)))))))))..))))..................(((((((....)))))))......)).)-

>AY761126 *Betula* *papyrifera* isolate 2892 internal transcribed spacer 1, partial sequence; 5.8S ribosomal RNA gene, complete sequence; and internal transcribed spacer 2, partial sequence

ACGUCUGCCUGGGUGUCACGCAUCGUUGCCCCCAACCCCAUCUCCUUGUAAAGGGACGAGGGGGCCUGUGGGGCAGAAAUUGGCCUCCCGUGAGCUCAUGCAUGCGGUUGGCCUAAAAGCGAGUCCUCGGCGACGCGCGCCACGACAAUCGGUGGUUGUCAAACCCUCGUGUCCCGUCGUGCGUGACGCGUCGCUCAUCGUGUGCUCCUUGACCCUGCUGUGUCGCGCUAGCGACGCUUCCAACGCG-

................(.((.....(((((((((.((((.(((((((....))))).)).))))..)).))))))).....((((..(((((.((....)).)))))..)))).....((((......((((((((((((((((((...(((.((((....)))).)))......)))))).))).)))))))))..))))..................(((((((....)))))))......)).)-

>AY761125 *Betula* *occidentalis* isolate 2883 internal transcribed spacer 1, partial sequence; 5.8S ribosomal RNA gene, complete sequence; and internal transcribed spacer 2, partial sequence

ACGUCUGCCUGGGUGUCACGCAUCGUUGCCCCCAACCCCAUCUCCUUGCAAAGGGACGAGGGGGCCUGUGGGGCAGAAAUUGGCCUCCCGUGAGCUCAUGCAUGCGGUUGGCCUAAAAGCGAGUCCUCGGCGACGCGCGCCACGACAAUCGGUGGUUGUCAAACCCUCGUGUCCCGUCGUGCGUGCCGCGUCGCUCAUCGUGUGCUCCUUGACCCUGCUGUGUCGCGCUAGCGACGCUUCCAACGCG-

................(.((.....(((((((((.((((.(((((((....))))).)).))))..)).))))))).....((((..(((((.((....)).)))))..)))).....((((......((((((((((((((((((...(((.((((....)))).)))......)))))).))).)))))))))..))))..................(((((((....)))))))......)).)-

>AY761124 *Betula* *nigra* isolate 2927 internal transcribed spacer 1, partial sequence; 5.8S ribosomal RNA gene, complete sequence; and internal transcribed spacer 2, partial sequence

ACGUCUGCCUGGGUGUCACGCAUCGUUGCCCCCAACCCCAUCUCCUUGCAAAGGGACGAGAGGGCCAGUGGGGUAGAAAUUGGCCUCCCGUGAGCUCAUGCAUGCGGUUGGCCUAAAAGCGAGUCCUCGGCGACGCGCGCCACGACAAUCGGUGGUUGACAAACCCUCGUGUCCCGUCGUGCGUGCCGCGUUGCUCAUCGUGUGCUCUUUGACCCUGUUGUGUCGCGCUAGCGAUGCUUCCAAUGCG-

................(.((.....((((((((..(((..(((((((....))))).))..)))...).))))))).....((((..(((((.((....)).)))))..)))).....((((......((((((((((((((((((...(((.((((....)))).)))......)))))).))).)))))))))..))))..................(((((((....)))))))......)).)-

>AY761123 *Betula* neoalaskana isolate 3466 internal transcribed spacer 1, partial sequence; 5.8S ribosomal RNA gene, complete sequence; and internal transcribed spacer 2, partial sequence

ACGUCUGCCUGGGUGUCACGCAUCGUUGCCCCCAACCCCAUCUCCUUGCAAAGGGACGAGGGGGCCUGUGGGGCAGAAAUUGGCCUCCCGUGAGCUCAUGCAUGCGGUUGGCCUAAAAGCGAGUCCUCGGCGACGCGCGCCACGACAAUCGGUGGUUGUCAAACCCUCGUGUCCCGUCGUGCGUGCCGCGUCGCUCAUCGUGUGCUCCUUGACCCUGYUGUGUCGCGCUAGCGACGCUUCCAACGCG-

................(.((.....(((((((((.((((.(((((((....))))).)).))))..)).))))))).....((((..(((((.((....)).)))))..)))).....((((......((((((((((((((((((...(((.((((....)))).)))......)))))).))).)))))))))..))))..................(((((((....)))))))......)).)-

>AY761122 *Betula* *nana* isolate 3337 internal transcribed spacer 1, partial sequence; 5.8S ribosomal RNA gene, complete sequence; and internal transcribed spacer 2, partial sequence

ACGUCUGCCUGGGUGUCACGCAUCGUUGCCCCCAACCCCAUCUCCUUGCAAAGGGACGAGGGGGCCUGUGGGGCAGAAAUUGGCCUCCCGUGAGCUCAUGCAUGCGGUUGGCCUAAAAGCGAGUCCUCGGCGACGCGCGCCACGACAAUCGGUGGUUGUCAAACCCUCGUGUCCCGUCGUGCGUGCCGCGUCGCUCAUCGUGUGCUCCUUGACCCUGCUGUGUCGCGCUAGCGACGCUUC-AACGCG-

................(.((.....(((((((((.((((.(((((((....))))).)).))))..)).))))))).....((((..(((((.((....)).)))))..)))).....((((......((((((((((((((((((...(((.((((....)))).)))......)))))).))).)))))))))..))))..................(((((((....)))))))...-..)).)-

>AY761121 *Betula* *michauxii* isolate 3406 internal transcribed spacer 1, partial sequence; 5.8S ribosomal RNA gene, complete sequence; and internal transcribed spacer 2, partial sequence

ACGUCUGCCUGGGUGUCACGCAUCGUUGCCCCCAACCCCAUCUCCUUGCAAAGGGACGAGGGGGCCUGUGGGGCAGAAAUUGGCCUCCCGUGAGCUCAUGCAUGCGGUUGGCCUAAAAGCGAGUUCUCGGCGACGCGCGCCACGACAAUCGGUGGUUGACAAACCCUGGUGUCCCGUCGUGCGUGCCGCGUCGCUCAUCGUGUGCUCUUUGACCCUGUUGUGUCGCGCUAGCGACGCUUCCAACGCG-

................(.((.....(((((((((.((((.(((((((....))))).)).))))..)).))))))).....((((..(((((.((....)).)))))..)))).....((((......((((((((((((((((((...(.(.((((....)))).).)......)))))).))).)))))))))..))))..................(((((((....)))))))......)).)-

>AY761120 *Betula* *medwediewii* isolate 3465 internal transcribed spacer 1, partial sequence; 5.8S ribosomal RNA gene, complete sequence; and internal transcribed spacer 2, partial sequence

ACGUCUGCCUGGGUGUCACGCAUCGUUGCCCCCAACCCCAUCUCCUUGCAAAGGGACGAGGGGGCCUGUGGGGCAGAAAUUGGCCUCCCGUGAGCUCAUGCAUGCGGUUGGCCUAAAAGCGAGUCCUCGGCGACGCGCGCCACGACAAUCGGUGGUUGACAAACCCUCGUGUCCCGUCGUGCGUGCCGCGUCGCUCAUCGUGUGCUCUUUGACCCUGCUGUGUCGCGCUAGCGACGCUUCCAACGCG-

................(.((.....(((((((((.((((.(((((((....))))).)).))))..)).))))))).....((((..(((((.((....)).)))))..)))).....((((......((((((((((((((((((...(((.((((....)))).)))......)))))).))).)))))))))..))))..................(((((((....)))))))......)).)-

>AY761119 *Betula* *maximovicziana* isolate 3463 internal transcribed spacer 1, partial sequence; 5.8S ribosomal RNA gene, complete sequence; and internal transcribed spacer 2, partial sequence

ACGUCUGCCUGGGUGUCACGCAUCGUUGCCCCCAACCCCAUCUCCUUGCAAAGGGACGAGGGGGCCUGUGGGGCAGAAAUUGGCCUCCCGUGAGCUCAUGCAUGCGGUUGGCCUAAA-GCGAGUCCUCGGCGACGCGCGCCACGACA-UCGGUGGUUGACAAACCCUCGUGUCCCGUCGUGCGUGCCGCGUCGCUCAUCGUGUGCUCUUUGACCCUGCUGUGUCGCGCUAGCGACGCUUCCAACGCG-

................(.((.....(((((((((.((((.(((((((....))))).)).))))..)).))))))).....((((..(((((.((....)).)))))..))))....-((((......((((((((((((((((((.-.(((.((((....)))).)))......)))))).))).)))))))))..))))..................(((((((....)))))))......)).)-

>AY761118 *Betula* *maximovicziana* isolate 2932 internal transcribed spacer 1, partial sequence; 5.8S ribosomal RNA gene, complete sequence; and internal transcribed spacer 2, partial sequence

ACGUCUGCCUGGGUGUCACGCAUCGUUGCCCCCAACCCCAUCUCCUUGCAAAGGGACGAGGGGGCCUGUGGGGCAGAAAUUGGCCUCCCGUGAGCUCAUGCAUGCGGUUGGCCUAAAAGCGAGUCCUCGGCGACGCGCGCCACGACAAUCGGUGGUUGACAAACCCUCGUGUCCCGUCGUGCGUGCCGCGUCGCUCAUCGUGUGCUCUUUGACCCUGCUGUGUCGCGCUAGCGACGCUUCCAACGCG-

................(.((.....(((((((((.((((.(((((((....))))).)).))))..)).))))))).....((((..(((((.((....)).)))))..)))).....((((......((((((((((((((((((...(((.((((....)))).)))......)))))).))).)))))))))..))))..................(((((((....)))))))......)).)-

>AY761117 *Betula* *luminifera* isolate 3299 internal transcribed spacer 1, partial sequence; 5.8S ribosomal RNA gene, complete sequence; and internal transcribed spacer 2, partial sequence

ACGUCUGCCUGGGUGUCACGCAUCGUUGCCCCCAACCCCAUCUCCUUGCAAAGGGACGAGGGGGCCAAUGGGGCAGAAAUUGGCCUCCCGUGAGCUCAUGCAUGCGGUUGGCCUAAAAGCGAGUCCUCGGCGACGCGCGCCACGACAAUCGGUGGUUGACAAACCCUCGUGUCCCGUCGUGCGUGCCGCGUUGCUCAUCGUGUGCUCUUUGACCCUGCUGUGUCGCGCUAGCGACGCUUCCAACGCG-

................(.((.....(((((((...((((.(((((((....))))).)).)))).....))))))).....((((..(((((.((....)).)))))..)))).....((((......((((((((((((((((((...(((.((((....)))).)))......)))))).))).)))))))))..))))..................(((((((....)))))))......)).)-

>AY761116 *Betula* *luminifera* isolate 2828 internal transcribed spacer 1, partial sequence; 5.8S ribosomal RNA gene, complete sequence; and internal transcribed spacer 2, partial sequence

ACGUCUGCCUGGGUGUCACGCAUCGUUGCCCCCAACCCCAUCUCCUUGCAAAGGGACGAGGGGGCCAAUGGGGCAGAAAUUGGCCUCCCGUGAGCUCAUGCAUGCGGUUGGCCUAAAAGCGAGUCCUCGGCGACGCGCGCCACGACAAUCGGUGGUUGACAAACCCUCGUGUCCCGUCGUGCGUGCCGCGUUGCUCAUCGUGUGCUCUUUGACCCUGCUGUGUCGCGCUAGCGACRCUUCCAACGCG-

................(.((.....(((((((...((((.(((((((....))))).)).)))).....))))))).....((((..(((((.((....)).)))))..)))).....((((......((((((((((((((((((...(((.((((....)))).)))......)))))).))).)))))))))..))))..................(((((((....)))))))......)).)-

>AY761115 *Betula* *lenta* isolate 2936 internal transcribed spacer 1, partial sequence; 5.8S ribosomal RNA gene, complete sequence; and internal transcribed spacer 2, partial sequence

ACGUCUGCCUGGGUGUCACGCAUCGUUGCCCCCAACCCCAUCUCCUUGCAAAGGGACGAGGGGGCCUGUGGGGCAGAAAUUGGCCUCCCGUGAGCUCAUGCAUGCGGUUGGCCUAAAAGCGAGUCCUCGGCGACGCGCGCCACGACAAUCGGUGGUUGACAAACCCUCGUGUCCCGUCGUGCGUGCCGCGUCGCUCAUCGUGUGCUCUUUGACCCUGCUGUGUCGCGCUAGCGACGCUUCCAACGCG-

................(.((.....(((((((((.((((.(((((((....))))).)).))))..)).))))))).....((((..(((((.((....)).)))))..)))).....((((......((((((((((((((((((...(((.((((....)))).)))......)))))).))).)))))))))..))))..................(((((((....)))))))......)).)-

>AY761114 *Betula* *humilis* isolate 2894 internal transcribed spacer 1, partial sequence; 5.8S ribosomal RNA gene, complete sequence; and internal transcribed spacer 2, partial sequence

ACGUCUGCCUGGGUGUCACGCAUCGUUGCCCCCAACCCCAUCUCCUUGCAAAGGGACGAGGGGGCCUGUGGGGCAGAAAUUGGCCUCCCGUGAGCUCAUGCAUGCGGUUGGCCUAAAAGCGAGUCCU-GGCGACGCGCGCCACGACAAUCGGUGGUUGUCAAACCCUCGUGUCCCGUCGUGCGUGCCGCGUCGCUCAUCGUGUGCUCCUUGACCCUGCUGUGUCGCGCUAGCGACGCUUCCAACGCG-

................(.((.....(((((((((.((((.(((((((....))))).)).))))..)).))))))).....((((..(((((.((....)).)))))..)))).....((((.....-((((((((((((((((((...(((.((((....)))).)))......)))))).))).)))))))))..))))..................(((((((....)))))))......)).)-

>AY761113 *Betula* *grossa* isolate 3459 internal transcribed spacer 1, partial sequence; 5.8S ribosomal RNA gene, complete sequence; and internal transcribed spacer 2, partial sequence

ACGUCUGCCUGGGUGUCACGCAUCGUUGCCCCCAACCCCAUCUCCUUGCAAAGGGACGAGGGGGCCUGUGGGGCAGAAAUUGGCCUCCCGUGAGCUCAUGCAUGCGGUUGGCCUAAAAGCGAGUCCUCGGCGACGCGCGCCACGACAAUCGGUGGUUGUCAAACCCUCGUGUCCCGUCGUGCGUGCCGCGUCGCUCAUCGUGUGCUCCUUGACCCUGCUGUGUCGCGCUAGCGACGCUUCCAACGCG-

................(.((.....(((((((((.((((.(((((((....))))).)).))))..)).))))))).....((((..(((((.((....)).)))))..)))).....((((......((((((((((((((((((...(((.((((....)))).)))......)))))).))).)))))))))..))))..................(((((((....)))))))......)).)-

>AY761112 *Betula* *grossa* isolate 2948 internal transcribed spacer 1, partial sequence; 5.8S ribosomal RNA gene, complete sequence; and internal transcribed spacer 2, partial sequence

ACGUCUGCCUGGGUGUCACGCAUCGUUGCCCCCAACCCCAUCUCCUUGCAAAGGGACGAGGGGGCCUGUGGGGCAGAAAUUGGCCUCCCGUGAGCUCAUGCAUGCGGUUGGCCUAAAAGCGAGUCCUCGGCGACGCGCGCCACGACAAUCGGUGGUUGUCAAACCCUCGUGUCCCGUCGUGCGUGCCGCGUCGCUCAUCGUGUGCUCCUUGACCCUGCUGUGUCGCGCUAGCGACGCUUCCAACGCG-

................(.((.....(((((((((.((((.(((((((....))))).)).))))..)).))))))).....((((..(((((.((....)).)))))..)))).....((((......((((((((((((((((((...(((.((((....)))).)))......)))))).))).)))))))))..))))..................(((((((....)))))))......)).)-

>AY761111 *Betula* *globispica* isolate 2942 internal transcribed spacer 1, partial sequence; 5.8S ribosomal RNA gene, complete sequence; and internal transcribed spacer 2, partial sequence

ACGUCUGCCUGGGUGUCACGCAUCGUUGCCCCCAACCCCAUCUCCUUGCAAAGGGACGAGGGG-CUUGUGGGGCAGAAAUUGGCCUCCCGUGAGCUUACGCAUGCGGUUGGCCUAAAAGCGAGUCCUCGGCGACGCGCGCCACGACAAUCGGUGGUUGACAAACCCUCGUGUCCCGUCGUGCGUGCCGCGUCGCUCAUCGUGUGCUCUUUGACCCUGCUGCGUCGCGCUAGCGACGCUUCCAACGCG-

................(.((.....(((((((((..(((.(((((((....))))).)).)))-..)).))))))).....((((..(((((.((....)).)))))..)))).....((((......((((((((((((((((((...(((.((((....)))).)))......)))))).))).)))))))))..))))..................(((((((....)))))))......)).)-

>AY761110 *Betula* *glandulosa* isolate 3251 internal transcribed spacer 1, partial sequence; 5.8S ribosomal RNA gene, complete sequence; and internal transcribed spacer 2, partial sequence

ACGUCUGCCUGGGUGUCACGCAUCGUUGCCCCCAACCCCAUCUCCUUGCAAAGGGACGAGGGG-CUUGUGGGGCAGAAAUUGGCCUCCCGUGAGCUUACGCAUGCGGUUGGCCUAAAAGCGAGUCCUCGGCGACGCGCGCCACGGCAAUCGGUGGUUGACAAACCUUCGUGUCCCGUCGUGCGUGCCGCGUCGCUCAUCGUGUGCUCUUUGACCCUGCUGCGUCGCGCUAGCGACGCUUCCAACGCG-

................(.((.....(((((((((..(((.(((((((....))))).)).)))-..)).))))))).....((((..(((((.((....)).)))))..)))).....((((......((((((((((((((((((...(((.((((....)))).)))......)))))).))).)))))))))..))))..................(((((((....)))))))......)).)-

>AY761109 *Betula* *fruticosa* isolate 3339 internal transcribed spacer 1, partial sequence; 5.8S ribosomal RNA gene, complete sequence; and internal transcribed spacer 2, partial sequence

ACGUCUGCCUGGGUGUCACGCAUCGUUGCCCCCAACCCCAUCUCCUUGCAAAGGGACGAGGGGGCCUGUGGGGCAGAAAUUGGCCUCCCGUGAGCUCAUGCAUGCGGUUGGCCUAAAAGCGAGUCCUCGGCGACGCGCGCCACGACAAUCGGUGGUUGUCAAACCCUCGUGUCCCGUCGUGCGUGACGCGUCGCUCAUCGUGUGCUCCUUGACCCUGCUGUGUCGCGCUAGCGACGCUUCCAACGCG-

................(.((.....(((((((((.((((.(((((((....))))).)).))))..)).))))))).....((((..(((((.((....)).)))))..)))).....((((......((((((((((((((((((...(((.((((....)))).)))......)))))).))).)))))))))..))))..................(((((((....)))))))......)).)-

>AY761108 *Betula* *ermanii* isolate 2961 internal transcribed spacer 1, partial sequence; 5.8S ribosomal RNA gene, complete sequence; and internal transcribed spacer 2, partial sequence

ACGUCUGCCUGGGUGUCACGCAUCGUUGCCCCCAACCCCAUCUCCUUGCAAAGGGACGAGGGGGCCUGUGGGGCAGAAAUUGGCCUCCCGUGAGCUCAUGCAUGCGGUUGGCCUAAAAGCGAGUCCUCGGCGACGCGCGCCACGACAAUCGGUGGUUGACAAACCCUCGUGUCCCGUCGUGCGUGCCGUGUCGCUCAUCGUGUGCUCUUUGACCCUGCUGUGUCGCGCUAGCGACGCUUCCAACGCG-

................(.((.....(((((((((.((((.(((((((....))))).)).))))..)).))))))).....((((..(((((.((....)).)))))..)))).....((((......((((((((((((((((((...(((.((((....)))).)))......)))))).))).)))))))))..))))..................(((((((....)))))))......)).)-

>AY761107 *Betula* *delavayi* isolate 3462 internal transcribed spacer 1, partial sequence; 5.8S ribosomal RNA gene, complete sequence; and internal transcribed spacer 2, partial sequence

ACGUCUGCCUGGGUGUCACGCAUCGUUGCCCCCAACCCCAUCUCCUUGCAAAGGGACGAAGGGGCCUGUGGGGCAGAAAUUGGCCUCCCGUGAGCUUAUGCAUGCGGUUGGCCUAAAAGCGAGUCCUCGGCGACGCGUGCCACGACAAUCGGUGGUUGACAAACCCUCGUGUCCCGUCGUGCGUGCCGCGUCGCUCAUCGUGUGCUCUUUGACCCUGCUGUGUCGUGCUAGCGACGCUUCCAACGCG-

................(.((.....(((((((((.((((.(((((((....))))).)).))))..)).))))))).....((((..(((((.((....)).)))))..)))).....((((......((((((((((((((((((...(((.((((....)))).)))......)))))).))).)))))))))..))))..................(((((((....)))))))......)).)-

>AY761106 *Betula* *corylifolia* isolate 3457 internal transcribed spacer 1, partial sequence; 5.8S ribosomal RNA gene, complete sequence; and internal transcribed spacer 2, partial sequence

ACGUCUGCCUGGGUGUCACGCAUCGUUGCCCCCAACCCCAUCUCCUUGCAAAGGGACGAGGGG-CUUGUGGGGCAGAAAUUGGCCUCCCGUGAGCUUAUGCAUGCGGUUGGCCUAAAAGCGAGUCCUCGGCGACGCGCGCCACGACAAUCGGUGGUUGACAAACCCUCGUGUCCCGUCGUGUGUGCCGCGUCGCUCAUCRUGUGCUCUUUGACCCUGCUGUGUCGCGCUAGCGACGCUUCCAACGCG-

................(.((.....(((((((((..(((.(((((((....))))).)).)))-..)).))))))).....((((..(((((.((....)).)))))..)))).....((((......((((((((((((((((((...(((.((((....)))).)))......)))))).))).)))))))))..))))..................(((((((....)))))))......)).)-

>AY761105 *Betula* *chinensis* isolate 2903 internal transcribed spacer 1, partial sequence; 5.8S ribosomal RNA gene, complete sequence; and internal transcribed spacer 2, partial sequence

ACGUCUGCCUGGGUGUCACGCAUCGUUGCCCCCAACCCCAUCUCCUUGUAAAGGGACGAGGGGGCCCGUGGGGCAGAAAUUGGCCUCCCGUGAGCUCAUGCAUGCGGUUGGCCUAAAAGCGAGUCCUCGGCGACGCGCGCCACGACAAUCGGUGGUUGUCAAACCCUCGUGUCCCGUCGUGCGUGACGCGUCGCUCAUCGUGUGCUCCUUGACCCUGCUGUGUCGCGCUAGCGACGCUUCCAACGCG-

................(.((.....((((((((..((((.(((((((....))))).)).))))...).))))))).....((((..(((((.((....)).)))))..)))).....((((......((((((((((((((((((...(((.((((....)))).)))......)))))).))).)))))))))..))))..................(((((((....)))))))......)).)-

>AY761104 *Betula* *chichibuensis* isolate 2977 internal transcribed spacer 1, partial sequence; 5.8S ribosomal RNA gene, complete sequence; and internal transcribed spacer 2, partial sequence

ACGUCUGCCUGGGUGUCACGCAUCGUUGCCCCCAACCCCAUCUCCUUGCAAAGGGACGAAGGGGCCUGUGGGGCAGAAAUUGGCCUCCCGUGAGCUUAUGCAUGCGGUUGGCCUAAAAGUGAGUCCUCGGCGACGCGCGCCACGACAAUCGGUGGUUGACAAACCCUCGUGUCCCGUCGUGCGUGCCGCGUCGCUCAUCGUGUGCUCUUUGACCCUACUGUGUCGUGCUAGCGACGCUUCCAACGCG-

................(.((.....(((((((((.((((.(((((((....))))).)).))))..)).))))))).....((((..(((((.((....)).)))))..)))).....((((......((((((((((((((((((...(((.((((....)))).)))......)))))).))).)))))))))..))))..................(((((((....)))))))......)).)-

>AY761103 *Betula* *calcicola* isolate 3460 internal transcribed spacer 1, partial sequence; 5.8S ribosomal RNA gene, complete sequence; and internal transcribed spacer 2, partial sequence

ACGUCUGCCUGGGUGUCACGCAUCGUUGCCCCCAACCCCAUCUCCUUGCAAAGGGACGAAGGGGCCUGUGGGGCAGAAAUUGGCCUCCCGUGAGCUUAUGCAUGCGGUUGGCCUAAAAGCGAGUCCUCGGCGACGCGUGCCACGACAAUCGGUGGUUGACAAACCCUCGUGUCCCGUCGUGCGUGCCGCAUCGCUCAUCGUGUGCUCUUUGACCCUGCUGUGUCGUGCUAGCGACGCUUCCAACGCG-

................(.((.....(((((((((.((((.(((((((....))))).)).))))..)).))))))).....((((..(((((.((....)).)))))..)))).....((((......(((((.((((((((((((...(((.((((....)))).)))......)))))).))).))).)))))..))))..................(((((((....)))))))......)).)-

>AY761102 *Betula* *apoiensis* isolate 3249 internal transcribed spacer 1, partial sequence; 5.8S ribosomal RNA gene, complete sequence; and internal transcribed spacer 2, partial sequence

ACGUCUGCCUGGGUGUCACGCAUCGUUGCCCCCAACCCCAUCUCCUUGCAAAGGGACGAGGGGGCCUGUGGGGCAGAAAUUGGCCUCCCGUGAGCUCAUGCAUGCGGUUGGCCUAAAAGCGAGUCCUCGGCGACGCGCGCCACGACAAUCGGUGGUUGUCAAACCCUCGUGUCCCGUCGUGCGUGMCGCGUCGCUCAUCGUGUGCUCCUUGACCCUGCUGUGUCGCGCUAGCGACGCUUCCAACGCG-

................(.((.....(((((((((.((((.(((((((....))))).)).))))..)).))))))).....((((..(((((.((....)).)))))..)))).....((((......((((((((((((((((((...(((.((((....)))).)))......)))))).))).)))))))))..))))..................(((((((....)))))))......)).)-

>AY761101 *Betula* *alnoides* isolate 3352 internal transcribed spacer 1, partial sequence; 5.8S ribosomal RNA gene, complete sequence; and internal transcribed spacer 2, partial sequence

ACGUCUGCCUGGGUGUCACGCAUCGUUGCCCCCAACCCCAUCUCCUUGCAAAGGGACGAGGGGGCCAGUGGGGCAGAAAUUGGCCUCCCGUGAGCUCAUGCAUGCGGUUGGCCUAAAAGCGAGUCCUCGGCGACGCGCGCCACGACAAUCGGUGGUUGACAAACCCUCGUGUCCCGUCGUGCGUGCCGCGUUGCUCAUCGUGUGCUCUUUGACCCUGCUGUGUCGCGCUAGCGAUGCUUCCAACGCG-

................(.((.....((((((((..((((.(((((((....))))).)).))))...).))))))).....((((..(((((.((....)).)))))..)))).....((((......((((((((((((((((((...(((.((((....)))).)))......)))))).))).)))))))))..))))..................(((((((....)))))))......)).)-

>AY761100 *Betula* *alleghaniensis* isolate 2880 internal transcribed spacer 1, partial sequence; 5.8S ribosomal RNA gene, complete sequence; and internal transcribed spacer 2, partial sequence

ACGUCUGCCUGGGUGUCACGCAUCGUUGCCCCCAACCCCAUCUCCUUGCAAAGGGACGAGGGGGCCUGUGGGGCAGAAAUUGGCCUCCCGUGAGCUCAUGCAUGCGGUUGGCCUAAAAGCGAGUCCUCGGCGACGCGCGCCACGACAAUCGGUGGUUGACAAACCCUCGUGUCCCGUCGUGCGUGCCGCGUCGCUCAUCGUGUGCUCUUUGACCCUGCUGUGUCGCGCCAGCGACGCUUCCAACGCG-

................(.((.....(((((((((.((((.(((((((....))))).)).))))..)).))))))).....((((..(((((.((....)).)))))..)))).....((((......((((((((((((((((((...(((.((((....)))).)))......)))))).))).)))))))))..))))..................(((((((....)))))))......)).)-

>AY761099 *Betula* *albosinensis* isolate 3018 internal transcribed spacer 1, partial sequence; 5.8S ribosomal RNA gene, complete sequence; and internal transcribed spacer 2, partial sequence

ACGUCUGCCUGGGUGUCACGCAUCGUUGCCCCCAACCCCAUCUCCUUGCAAAGGGACGAGGGGGCCAGUGGGGCAGAAAUUGGCCUCCCGUGAGCUCAUGCAUGCGGUUGGCCUAAAAGCGAGUCCUCGGCGACGCACGCCACGACAAUCGGUGGUUGACAAACCCUCGUGUCCCGUCGUGCGUGCCGCGUCGCUCAUCGUGUGCUCUUUGACCCUGUUGUGUCGCGCUAGCGACGCUUCCAACGCG-

................(.((.....((((((((..((((.(((((((....))))).)).))))...).))))))).....((((..(((((.((....)).)))))..)))).....((((......((((((((.(((((((((...(((.((((....)))).)))......)))))).)))..))))))))..))))..................(((((((....)))))))......)).)-

>AY352337 *Betula* *costata* internal transcribed spacer 1, partial sequence; 5.8S ribosomal RNA gene, complete sequence; and internal transcribed spacer 2, partial sequence

ACGUCUGCCUGGGUGUCACGCAUCGUUGCCCCCAACCCCAUCUCCUUGCAAAGGGACGAGGGGGCCUGUGGGGCAGAAAUUGGCCUCCCGUGAGCUCAUGCAUGCGGUUGGCCUAAAAGCGAGUCCUCGGCGACGCGCGCCACGACAAUCGGUGGUUGACAAACCCUCGUGUCCCGUCGUGCGUGCCGCGUCGCYCAUCGUGUGCUCUUUGACCCUGCUGUGUCGCGCCAGCGACGCUUCCAACGCG-

................(.((.....(((((((((.((((.(((((((....))))).)).))))..)).))))))).....((((..(((((.((....)).)))))..)))).....((((......((((((((((((((((((...(((.((((....)))).)))......)))))).))).)))))))))..))))..................(((((((....)))))))......)).)-

>AY352336 *Betula* *nana* internal transcribed spacer 1, partial sequence; 5.8S ribosomal RNA gene, complete sequence; and internal transcribed spacer 2, partial sequence

ACGUCUGCCUGGGUGUCACGCAUCGUUGCCCCCAACCCCAUCUCCUUGCAAAGGGACGAGGGGGCCUGUGGGGCAGAAAUUGGCCUCCCGUGAGCUCAUGCAUGCGGUUGGCCUAAAAGCGAGUCCUCGGCGACGCGCGCCACGACAAUCGGUGGUUGUCAAACCCUCGUGUCCCGUCGUGCGUGCCGCGUCGCUCAUCGUGUGCUCCUUGACCCUGCUGUGUCGCGCUAGCGACGCUUCCAACGCG-

................(.((.....(((((((((.((((.(((((((....))))).)).))))..)).))))))).....((((..(((((.((....)).)))))..)))).....((((......((((((((((((((((((...(((.((((....)))).)))......)))))).))).)))))))))..))))..................(((((((....)))))))......)).)-

>AY352332 *Betula* *pendula* internal transcribed spacer 1, partial sequence; 5.8S ribosomal RNA gene, complete sequence; and internal transcribed spacer 2, partial sequence

ACGUCUGCCUGGGUGUCACGCAUCGUUGCCCCCAACCCCAUCUCCUUGCAAAGGGACGAGGGGGCCUGUGGGGCAGAAAUUGGCCUCCCGUGAGCUCAUGCAUGCGGUUGGCCUAAAAGCGAGUCCUCGGCGACGCGCGCCACGACAAUCGGUGGUUGUCAAACCCUCGUGUCCCGUCGUGCGUGCCGCGUCGCUCAUCGUGUGCUCCUUGACCCUGUUGUGUCGCGCUAGCGACGCUUCCAACGCG-

................(.((.....(((((((((.((((.(((((((....))))).)).))))..)).))))))).....((((..(((((.((....)).)))))..)))).....((((......((((((((((((((((((...(((.((((....)))).)))......)))))).))).)))))))))..))))..................(((((((....)))))))......)).)-

>AY352331 *Betula* *nigra* internal transcribed spacer 1, partial sequence; 5.8S ribosomal RNA gene, complete sequence; and internal transcribed spacer 2, partial sequence

ACGUCUGCCUGGGUGUCACGCAUCGUUGCCCCCAACCCCAUCUCCUUGCAAAGGGACGAGAGGGCCAGUGGGGUAGAAAUUGGCCUCCCGUGAGCUCAUGCAUGCGGUUGGCCUAAAAGCGAGUCCUCGGCGACGCGCGCCACGACAAUCGGUGGUUGACAAACCCUCGUGUCCCGUCGUGCGUGCCGCGUUGCUCAUCGUGUGCUCUUUGACCCUGUUGUGUCGCGCUAGCGAUGCUUCCAAUGCG-

................(.((.....((((((((..(((..(((((((....))))).))..)))...).))))))).....((((..(((((.((....)).)))))..)))).....((((......((((((((((((((((((...(((.((((....)))).)))......)))))).))).)))))))))..))))..................(((((((....)))))))......)).)-

>AY352330 *Betula* *lenta* internal transcribed spacer 1, partial sequence; 5.8S ribosomal RNA gene, complete sequence; and internal transcribed spacer 2, partial sequence

ACGUCUGCCUGGGUGUCACGCAUCGUUGCCCCCAACCCCAUCUCCUUGCAAAGGGACGAGGGGGCCUGUGGGGCAGAAAUUGGCCUCCCGUGAGCUCAUGCAUGCGGUUGGCCUAAAAGCGAGUCCUCGGCGACGCGCGCCACGACAAUCGGUGGUUGACAAACCCUCGUGUCCCGUCGUGCGUGCCGCGUCGCUCAUCGUGUGCUCUUUGACCCUGCUGUGUCGCGCUAGCGACGCUUCCAACGCG-

................(.((.....(((((((((.((((.(((((((....))))).)).))))..)).))))))).....((((..(((((.((....)).)))))..)))).....((((......((((((((((((((((((...(((.((((....)))).)))......)))))).))).)))))))))..))))..................(((((((....)))))))......)).)-

>AF432067 *Betula* *papyrifera* internal transcribed spacer 1, partial sequence; 5.8S ribosomal RNA gene, complete sequence; and internal transcribed spacer 2, partial sequence

ACGUCUGCCUGGGUGUCACGCAUCGUUGCCCCCAACCCCAUCUCCUUGUAAAGGGACGAGGGGGCCUGUGGGGCAGAAAUUGGCCUCCCGUGAGCUCAUGCAUGCGGUUGGCCUAAAAGCGAGUCCUCGGCGACGCACGCCACGACAAUCGGUGGUUGUCAAACCCUCGUGUCCCGUCGUGCGUGACGCGUCGCUCAUCGUGUGCUCCUUGACCCUGCUGUGUCGCGCUAGCGACGCUUCCAACGCGA

...............((.((.....(((((((((.((((.(((((((....))))).)).))))..)).))))))).....((((..(((((.((....)).)))))..)))).....((((......((((((((.(((((((((...(((.((((....)))).)))......)))))).)))..))))))))..))))..................(((((((....)))))))......)).))

>DQ397523 *Betula* *occidentalis* internal transcribed spacer 1, partial sequence; 5.8S ribosomal RNA gene, complete sequence; and internal transcribed spacer 2, partial sequence

ACGUCUGCCUGGGUGUCACGCAUCGUUGCCCCCAACCCCAUCUCCUUGCAAAGGGACGAGGGGGCCUGUGGGGCAGAAAUUGGCCUCCCGUGAGCUCAUGCAUGCGGUUGGCCUAAAAGCGAGUCCUCGGCGACGCGCGCCACGACAAUCGGUGGUUGUCAAACCCUCGUGUCCCGUCGUGCGUGCCGCGUCGCUCAUCGUGUGCUCCUUGACCCUGCUGUGUCGCGCUAGCGACGCUUCCAACGCGA

...............((.((.....(((((((((.((((.(((((((....))))).)).))))..)).))))))).....((((..(((((.((....)).)))))..)))).....((((......((((((((((((((((((...(((.((((....)))).)))......)))))).))).)))))))))..))))..................(((((((....)))))))......)).))

>LC382209.1 *Betula* *platyphylla* var. japonica Yuki Shiotani 63 genes for ITS1, 5.8S rRNA, ITS2, partial and complete sequence

ACGUCUGCCUGGGUGUCACGCAUCGUUGCCCCCAACCCCAUCUCCUUGCAAAGGGACGAGGGGGCCUGUGGGGCAGAAAUUGGCCUCCCGUGAGCUCAUGCAUGCGGUUGGCCUAAAAGCGAGUCCUCGGCGACGCGCGCCACGACAAUCGGUGGUUGUCAAACCCUCGUGUCCCGUCGUGCGUGCCGCGUCGCUCAUCGUGUGCUCCUUGACCCUGCUGUGUCGCGCUAGCGACGCUUCCAACGCG-

................(.((.....(((((((((.((((.(((((((....))))).)).))))..)).))))))).....((((..(((((.((....)).)))))..)))).....((((......((((((((((((((((((...(((.((((....)))).)))......)))))).))).)))))))))..))))..................(((((((....)))))))......)).)-

>LC382208.1 *Betula* *platyphylla* var. japonica Yuki Shiotani 62 genes for ITS1, 5.8S rRNA, ITS2, partial and complete sequence

ACGUCUGCCUGGGUGUCACGCAUCGUUGCCCCCAACCCCAUCUCCUUGCAAAGGGACGAGGGGGCCUGUGGGGCAGAAAUUGGCCUCCCGUGAGCUCAUGCAUGCGGUUGGCCUAAAAGCGAGUCCUCGGCGACGCGCGCCACGACAAUCGGUGGUUGUCAAACCCUCGUGUCCCGUCGUGCGUGCCGCGUCGCUCAUCGUGUGCUCCUUGACCCUGCUGUGUCGCGCUAGCGACGCUUCCAACGCG-

................(.((.....(((((((((.((((.(((((((....))))).)).))))..)).))))))).....((((..(((((.((....)).)))))..)))).....((((......((((((((((((((((((...(((.((((....)))).)))......)))))).))).)))))))))..))))..................(((((((....)))))))......)).)-

>LC382207.1 *Betula* *ermanii* Yuki Shiotani er2 genes for ITS1, 5.8S rRNA, ITS2, partial and complete sequence

ACGUCUGCCUGGGUGUCACGCAUCGUUGCCCCCAACCCCAUCUCCUUGCAAAGGGACGAGGGGGCCUGUGGGGCAGAAAUUGGCCUCCCGUGAGCUCAUGCAUGCGGUUGGCCUAAAAGCGAGUCCUCGGCGACGCGCGCCACGACAAUCGGUGGUUGACAAACCCUCGUGUCCCGUCGUGCGUGCCGYGUCGCUCAUCGUGUGCUCUUUGACCCUGCUGUGUCGCGCUAGCGACGCUUCCAACGCG-

................(.((.....(((((((((.((((.(((((((....))))).)).))))..)).))))))).....((((..(((((.((....)).)))))..)))).....((((......((((((((((((((((((...(((.((((....)))).)))......)))))).))).)))))))))..))))..................(((((((....)))))))......)).)-

>LC382206.1 *Betula* *ermanii* Yuki Shiotani er1 genes for ITS1, 5.8S rRNA, ITS2, partial and complete sequence

ACGUCUGCCUGGGUGUCACGCAUCGUUGCCCCCAACCCCAUCUCCUUGCAAAGGGACGAGGGGGCCUGUGGGGCAGAAAUUGGCCUCCCGUGAGCUCAUGCAUGCGGUUGGCCUAAAAGCGAGUCCUCGGCGACGCGCGCCACGACAAUCGGUGGUUGACAAACCCUCGUGUCCCGUCGUGCGUGCCGYGUCGCUCAUCGUGUGCUCUUUGACCCUGCUGUGUCGCGCUAGCGACGCUUCCAACGCG-

................(.((.....(((((((((.((((.(((((((....))))).)).))))..)).))))))).....((((..(((((.((....)).)))))..)))).....((((......((((((((((((((((((...(((.((((....)))).)))......)))))).))).)))))))))..))))..................(((((((....)))))))......)).)-

>LC382205.1 *Betula* *ovalifolia* Yuki Shiotani 77 genes for ITS1, 5.8S rRNA, ITS2, partial and complete sequence

ACGUCUGCCUGGGUGUCACGCAUCGUUGCCCCCAACCCCAUCUCCUUGAAAAGGGACGAGGGGGCCUGUGGGGCAGAAAUUGGCCUCCCGUGAGCUCAUGCAUGCGGUUGGUCUAAAAGCGAGUCCUCGGCGACGCGCGCCACGACAAUCGGUGGUUGUCAAACCCUCGUGUCCCGUCGUGCGUGACGCGUCGCUCAUCGUGUGCUCCUUGACCCUGCUGUGUCGUGCUAGCGACGCUUCCAACGCG-

................(.((.....(((((((((.((((.(((((((....))))).)).))))..)).))))))).....((((..(((((.((....)).)))))..)))).....((((......((((((((((((((((((...(((.((((....)))).)))......)))))).))).)))))))))..))))..................(((((((....)))))))......)).)-

>LC382204.1 *Betula* *ovalifolia* Yuki Shiotani 41 genes for ITS1, 5.8S rRNA, ITS2, partial and complete sequence

ACGUCUGCCUGGGUGUCACGCAUCGUUGCCCCCAACCCCAUCUCCUUGAAAAGGGACGAGGGGGCCUGUGGGGCAGAAAUUGGCCUCCCGUGAGCUCAUGCAUGCGGUUGGUCUAAAAGCGAGUCCUCGGCGACGCGCGCCACGACAAUCGGUGGUUGUCAAACCCUCGUGUCCCGUCGUGCGUGACGCGUCGCUCAUCGUGUGCUCCUUGACCCUGCUGUGUCGUGCUAGCGACGCUUCCAACGCG-

................(.((.....(((((((((.((((.(((((((....))))).)).))))..)).))))))).....((((..(((((.((....)).)))))..)))).....((((......((((((((((((((((((...(((.((((....)))).)))......)))))).))).)))))))))..))))..................(((((((....)))))))......)).)-

>LC382025.1 *Betula* *ovalifolia × Betula* *ermanii* Yuki Shiotani 54 genes for ITS1, 5.8S rRNA, ITS2, partial and complete sequence, clone: C2

ACGUCUGCCUGGGUGUCACGCAUCGUUGCCCCCAACCCCAUCUCCUUGMAAAGGGACGAGGGGGCCUGUGGGGCAGAAAUUGGCCUCCCGUGAGCUCAUGCAUGCGGUUGGYCUAAAAGCGAGUCCUCGGCGACGCGCGCCACGACAAUCGGUGGUUGWCAAACCCUCGUGUCCCGUCGUGCGUGMCGYGUCGCUCAUCGUGUGCUCYUUGACCCUGCUGUGUCGYGCUAGCGACGCUUCCAACGCG-

................(.((.....(((((((((.((((.(((((((....))))).)).))))..)).))))))).....((((..(((((.((....)).)))))..)))).....((((......((((((((((((((((((...(((.((((....)))).)))......)))))).))).)))))))))..))))..................(((((((....)))))))......)).)-

>LC382024.1 *Betula* *ovalifolia × Betula* *ermanii* Yuki Shiotani 53 genes for ITS1, 5.8S rRNA, ITS2, partial and complete sequence, clone: C1

ACGUCUGCCUGGGUGUCACGCAUCGUUGCCCCCAACCCCAUCUCCUUGMAAAGGGACGAGGGGGCCUGUGGGGCAGAAAUUGGCCUCCCGUGAGCUCAUGCAUGCGGUUGGYCUAAAAGCGAGUCCUCGGCGACGCGCGCCACGACAAUCGGUGGUUGWCAAACCCUCGUGUCCCGUCGUGCGUGMCGYGUCGCUCAUCGUGUGCUCYUUGACCCUGCUGUGUCGYGCUAGCGACGCUUCCAACGCG-

................(.((.....(((((((((.((((.(((((((....))))).)).))))..)).))))))).....((((..(((((.((....)).)))))..)))).....((((......((((((((((((((((((...(((.((((....)))).)))......)))))).))).)))))))))..))))..................(((((((....)))))))......)).)-

>MG237810.1 *Betula* *minor* voucher CCDB-18297-G08 5.8S ribosomal RNA gene, partial sequence; internal transcribed spacer 2, complete sequence; and large subunit ribosomal RNA gene, partial sequence

ACGUCUGCCUGGGUGUCACGCAUCGUUGCCCCCAACCCCAUCUCCUUGCAAAGGGACGAGGGGGCCUGUGGGGCAGAAAUUGGCCUCCCGUGAGCUCAUGCAUGCGGUUGGCCUAAAAGCGAGUCCUCGGCGACGCGCGCCACGACAAUCGGUGGUUGUCAAACCCUCGUGUCCCGUCGUGCGUGCCGCGUCGCUCAUCGUGUGCUCCUUGACCCUGCUGUGUCGCGCUAGCGACGCUUCCAACGCGA

...............((.((.....(((((((((.((((.(((((((....))))).)).))))..)).))))))).....((((..(((((.((....)).)))))..)))).....((((......((((((((((((((((((...(((.((((....)))).)))......)))))).))).)))))))))..))))..................(((((((....)))))))......)).))

>MG237745.1 *Betula* *populifolia* voucher MT00179824 5.8S ribosomal RNA gene, partial sequence; internal transcribed spacer 2, complete sequence; and large subunit ribosomal RNA gene, partial sequence

ACGUCUGCCUGGGUGUCACGCAUCGUUGCCCCCAACCCCAUCUCCUUGCAAAGGGACGAGGGGGCCUGUGGGGCAGAAAUUGGCCUCCCGUGAGCUCAUGCAUGCGGUUGGCCUAAAAGCGAGUCCUCGGCGACGCGCGCCACGACAAUCGGUGGUUGUCAAACCCUCGUGUCCCGUCGUGCGUGCCGCGUCGCUCAUCGUGUGCUCCUUGACCCUGCUGUGUCGCGCUAGCGACGCUUCCAACGCGA

...............((.((.....(((((((((.((((.(((((((....))))).)).))))..)).))))))).....((((..(((((.((....)).)))))..)))).....((((......((((((((((((((((((...(((.((((....)))).)))......)))))).))).)))))))))..))))..................(((((((....)))))))......)).))

>MG237590.1 *Betula* *occidentalis* voucher CCDB-18325-E2 5.8S ribosomal RNA gene, partial sequence; internal transcribed spacer 2, complete sequence; and large subunit ribosomal RNA gene, partial sequence

ACGUCUGCCUGGGUGUCACGCAUCGUUGCCCCCAACCCCAUCUCCUUGCAAAGGGACGAGGGGGCCUGUGGGGCAGAAAUUGGCCUCCCGUGAGCUCAUGCAUGCGGUUGGCCUAAAAGCGAGUCCUCGGCGACGCGCGCCACGACAAUCGGUGGUUGUCAAACCCUCGUGUCCCGUCGUGCGUGCCGCGUCGCUCAUCGUGUGCUCCUUGACCCUGCUGUGUCGCGCUAGCGACGCUUCCAACGCGA

...............((.((.....(((((((((.((((.(((((((....))))).)).))))..)).))))))).....((((..(((((.((....)).)))))..)))).....((((......((((((((((((((((((...(((.((((....)))).)))......)))))).))).)))))))))..))))..................(((((((....)))))))......)).))

>MG237484.1 *Betula* neoalaskana voucher CCDB-18325-H2 5.8S ribosomal RNA gene, partial sequence; internal transcribed spacer 2, complete sequence; and large subunit ribosomal RNA gene, partial sequence

ACGUCUGCCUGGGUGUCACGCAUCGUUGCCCCCAACCCCAUCUCCUUGCAAAGGGACGAGGGGGCCUGUGGGGCAGAAAUUGGCCUCCCGUGAGCUCAUGCAUGCGGUUGGCCUAAAAGCGAGUCCUCGGCGACGCGCGCCACGACAAUCGGUGGUUGUCAAACCCUCGUGUCCCGUCGUGCGUGCCGCGUCGCUCAUCGUGUGCUCCUUGACCCUGCUGUGUCGCGCUAGCGACGCUUCCAACGCGA

...............((.((.....(((((((((.((((.(((((((....))))).)).))))..)).))))))).....((((..(((((.((....)).)))))..)))).....((((......((((((((((((((((((...(((.((((....)))).)))......)))))).))).)))))))))..))))..................(((((((....)))))))......)).))

>MG237429.1 *Betula* *nana* voucher CCDB-18325-F1 5.8S ribosomal RNA gene, partial sequence; internal transcribed spacer 2, complete sequence; and large subunit ribosomal RNA gene, partial sequence

ACGUCUGCCUGGGUGUCACGCAUCGUUGCCCCCAACCCCAUCUCCUUGCAAAGGGACGAGGGGGCCUGUGGGGCAGAAAUUGGCCUCCCGUGAGCUCAUGCAUGCGGUUGGCCUAAAAGCGAGUCCUCGGCGACGCGCGCCACGACAAUCGGUGGUUGUCAAACCCUCGUGUCCCGUCGUGCGUGCCGCGUCGCUCAUCGUGUGCUCCUUGACCCUGCUGUGUCGCGCUAGCGACGCUUCCAACGCGA

...............((.((.....(((((((((.((((.(((((((....))))).)).))))..)).))))))).....((((..(((((.((....)).)))))..)))).....((((......((((((((((((((((((...(((.((((....)))).)))......)))))).))).)))))))))..))))..................(((((((....)))))))......)).))

>MG237283.1 *Betula* *occidentalis* voucher CCDB-18325-F2 5.8S ribosomal RNA gene, partial sequence; internal transcribed spacer 2, complete sequence; and large subunit ribosomal RNA gene, partial sequence

ACGUCUGCCUGGGUGUCACGCAUCGUUGCCCCCAACCCCAUCUCCUUGCAAAGGGACGAGGGGGCCUGUGGGGCAGAAAUUGGCCUCCCGUGAGCUCAUGCAUGCGGUUGGCCUAAAAGCGAGUCCUCGGCGACGCGCGCCACGACAAUCGGUGGUUGUCAAACCCUCGUGUCCCGUCGUGCGUGCCGCGUCGCUCAUCGUGUGCUCCUUGACCCUGCUGUGUCGCGCUAGCGACGCUUCCAACGCGA

...............((.((.....(((((((((.((((.(((((((....))))).)).))))..)).))))))).....((((..(((((.((....)).)))))..)))).....((((......((((((((((((((((((...(((.((((....)))).)))......)))))).))).)))))))))..))))..................(((((((....)))))))......)).))

>MG237175.1 *Betula* *lenta* voucher CCDB-18297-B07 5.8S ribosomal RNA gene, partial sequence; internal transcribed spacer 2, complete sequence; and large subunit ribosomal RNA gene, partial sequence

ACGUCUGCCUGGGUGUCACGCAUCGUUGCCCCCAACCCCAUCUCCUUGCAAAGGGACGAGGGGGCCUGUGGGGCAGAAAUUGGCCUCCCGUGAGCUCAUGCAUGCGGUUGGCCUAAAAGCGAGUCCUCGGCGACGCGCGCCACGACAAUCGGUGGUUGACAAACCCUCGUGUCCCGUCGUGCGUGCCGCGUCGCUCAUCGUGUGCUCUUUGACCCUGCUGUGUCGCGCCAGCGACGCUUCCAACGCGA

...............((.((.....(((((((((.((((.(((((((....))))).)).))))..)).))))))).....((((..(((((.((....)).)))))..)))).....((((......((((((((((((((((((...(((.((((....)))).)))......)))))).))).)))))))))..))))..................(((((((....)))))))......)).))

>MG236939.1 *Betula* *papyrifera* voucher CCDB-18325-B2 5.8S ribosomal RNA gene, partial sequence; internal transcribed spacer 2, complete sequence; and large subunit ribosomal RNA gene, partial sequence

ACGUCUGCCUGGGUGUCACGCAUCGUUGCCCCCAACCCCAUCUCCUUGUAAAGGGACGAGGGGGCCUGUGGGGCAGAAAUUGGCCUCCCGUGAGCUCAUGCAUGCGGUUGGCCUAAAAGCGAGUCCUCGGCGACGCACGCCACGACAAUCGGUGGUUGUCAAACCCUCGUGUCCCGUCGUGCGUGACGCGUCGCUCAUCGUGUGCUCCUUGACCCUGCUGUGUCGCGCUAGCGACGCUUCCAACGCGA

...............((.((.....(((((((((.((((.(((((((....))))).)).))))..)).))))))).....((((..(((((.((....)).)))))..)))).....((((......((((((((.(((((((((...(((.((((....)))).)))......)))))).)))..))))))))..))))..................(((((((....)))))))......)).))

>MG236820.1 *Betula* kenaica voucher CCDB-18325-D1 5.8S ribosomal RNA gene, partial sequence; internal transcribed spacer 2, complete sequence; and large subunit ribosomal RNA gene, partial sequence

ACGUCUGCCUGGGUGUCACGCAUCGUUGCCCCCAACCCCAUCUCCUUGCAAAGGGACGAGGGGGCCUGUGGGGCAGAAAUUGGCCUCCCGUGAGCUCAUGCAUGCGGUUGGCCUAAAAGCGAGUCCUCGGCGACGCGCGCCACGACAAUCGGUGGUUGUCAAACCCUCGUGUCCCGUCGUGCGUGCCGCGUCGCUCAUCGUGUGCUCCUUGACCCUGUUGUGUCGCGCUAGCGACGCUUCCAACGCGA

...............((.((.....(((((((((.((((.(((((((....))))).)).))))..)).))))))).....((((..(((((.((....)).)))))..)))).....((((......((((((((((((((((((...(((.((((....)))).)))......)))))).))).)))))))))..))))..................(((((((....)))))))......)).))

>MG236767.1 *Betula* *glandulosa* voucher CCDB-18325-B1 5.8S ribosomal RNA gene, partial sequence; internal transcribed spacer 2, complete sequence; and large subunit ribosomal RNA gene, partial sequence

ACGUCUGCCUGGGUGUCACGCAUCGUUGCCCCCAACCCCAUCUCCUUGCAAAGGGACGAGGGGGCCUGUGGGGCAGAAAUUGGCCUCCCGUGAGCUCAUGCAUGCGGUUGGCCUAAAAGCGAGUCCUCGGCGACGCGCGCCACGACAAUCGGUGGUUGUCAAACCCUCGUGUCCCGUCGUGCGUGCCGCGUCGCUCAUCGUGUGCUCCUUGACCCUGCUGUGUCGCGCUAGCGACGCUUCCAACGCGA

...............((.((.....(((((((((.((((.(((((((....))))).)).))))..)).))))))).....((((..(((((.((....)).)))))..)))).....((((......((((((((((((((((((...(((.((((....)))).)))......)))))).))).)))))))))..))))..................(((((((....)))))))......)).))

>MG236556.1 *Betula* *occidentalis* voucher CCDB-18325-D2 5.8S ribosomal RNA gene, partial sequence; internal transcribed spacer 2, complete sequence; and large subunit ribosomal RNA gene, partial sequence

ACGUCUGCCUGGGUGUCACGCAUCGUUGCCCCCAACCCCAUCUCCUUGCAAAGGGACGAGGGGGCCUGUGGGGCAGAAAUUGGCCUCCCGUGAGCUCAUGCAUGCGGUUGGCCUAAAAGCGAGUCCUCGGCGACGCGCGCCACGACAAUCGGUGGUUGUCAAACCCUCGUGUCCCGUCGUGCGUGCCGCGUCGCUCAUCGUGUGCUCCUUGACCCUGCUGUGUCGCGCUAGCGACGCUUCCAACGCGA

...............((.((.....(((((((((.((((.(((((((....))))).)).))))..)).))))))).....((((..(((((.((....)).)))))..)))).....((((......((((((((((((((((((...(((.((((....)))).)))......)))))).))).)))))))))..))))..................(((((((....)))))))......)).))

>MG236141.1 *Betula* neoalaskana voucher CCDB-18325-G2 5.8S ribosomal RNA gene, partial sequence; internal transcribed spacer 2, complete sequence; and large subunit ribosomal RNA gene, partial sequence

ACGUCUGCCUGGGUGUCACGCAUCGUUGCCCCCAACCCCAUCUCCUUGUAAAGGGACGAGGGGGCCUGUGGGGCAGAAAUUGGCCUCCCGUGAGCUCAUGCAUGCGGUUGGCCUAAAAGCGAGUCCUCGGCGACGCGCGCCACGACAAUCGGUGGUUGUCAAACCCUCGUGUCCCGUCGUGCGUGACGCGUCGCUCAUCGUGUGCUCCUUGACCCUGCUGUGUCGCGCUAGCGACGCUUCCAACGCGA

...............((.((.....(((((((((.((((.(((((((....))))).)).))))..)).))))))).....((((..(((((.((....)).)))))..)))).....((((......((((((((((((((((((...(((.((((....)))).)))......)))))).))).)))))))))..))))..................(((((((....)))))))......)).))

>MG235907.1 *Betula* *nana* voucher CCDB-18325-E1 5.8S ribosomal RNA gene, partial sequence; internal transcribed spacer 2, complete sequence; and large subunit ribosomal RNA gene, partial sequence

ACGUCUGCCUGGGUGUCACGCAUCGUUGCCCCCAACCCCAUCUCCUUGCAAAGGGACGAGGGGGCCUGUGGGGCAGAAAUUGGCCUCCCGUGAGCUCAUGCAUGCGGUUGGCCUAAAAGCGAGUCCUCGGCGACGCGUGCCACGACAAUCGGUGGUUGUCAAACCCUCGUGUCCCGUCGUGCGUGCCGCGUCGCUCAUCGUGUGCUCCUUGACCCUGCUGUGUCGCGCUAGCGACGCUUCCAACGCGA

...............((.((.....(((((((((.((((.(((((((....))))).)).))))..)).))))))).....((((..(((((.((....)).)))))..)))).....((((......((((((((((((((((((...(((.((((....)))).)))......)))))).))).)))))))))..))))..................(((((((....)))))))......)).))

>MG235784.1 *Betula* *alleghaniensis* voucher CCDB-18325-G1 5.8S ribosomal RNA gene, partial sequence; internal transcribed spacer 2, complete sequence; and large subunit ribosomal RNA gene, partial sequence

ACGUCUGCCUGGGUGUCACGCAUCGUUGCCCCCAACCCCAUCUCCUUGCAAAGGGACGAGGGGGCCUGUGGGGCAGAAAUUGGCCUCCCGUGAGCUCAUGCAUGCGGUUGGCCUAAAAGCGAGUCCUCGGCGACGCGCGCCACGACAAUCGGUGGUUGACAAACCCUCGUGUCCCGUCGUGCGUGCCGCGUCGCUCAUCGUGUGCUCUUUGACCCUGCUGUGUCGCGCCAGCGACGCUUCCAACGCGA

...............((.((.....(((((((((.((((.(((((((....))))).)).))))..)).))))))).....((((..(((((.((....)).)))))..)))).....((((......((((((((((((((((((...(((.((((....)))).)))......)))))).))).)))))))))..))))..................(((((((....)))))))......)).))

>MG235525.1 *Betula* *cordifolia* voucher CCDB-18297-A07 5.8S ribosomal RNA gene, partial sequence; internal transcribed spacer 2, complete sequence; and large subunit ribosomal RNA gene, partial sequence

ACGUCUGCCUGGGUGUCACGCAUCGUUGCCCCCAACCCCAUCUCCUUGCAAAGGGACGAGGGGGCCUGUGGGGCAGAAAUUGGCCUCCCGUGAGCUCAUGCAUGCGGUUGGCCUAAAAGCGAGUCCUCGGCGACGCGCGCCACGACAAUCGGUGGUUGUCAAACCCUCGUGUCCCGUCGUGCGUGCCGCGUCGCUCAUCGUGUGCUCCUUGACCCUGCUGUGUCGCGCUAGCGACGCUUCCAACGCGA

...............((.((.....(((((((((.((((.(((((((....))))).)).))))..)).))))))).....((((..(((((.((....)).)))))..)))).....((((......((((((((((((((((((...(((.((((....)))).)))......)))))).))).)))))))))..))))..................(((((((....)))))))......)).))

>MG235191.1 *Betula* neoalaskana voucher CCDB-18325-A1 5.8S ribosomal RNA gene, partial sequence; internal transcribed spacer 2, complete sequence; and large subunit ribosomal RNA gene, partial sequence

ACGUCUGCCUGGGUGUCACGCAUCGUUGCCCCCAACCCCAUCUCCUUGUAAAGGGACGAGGGGGCCUGUGGGGCAGAAAUUGGCCUCCCGUGAGCUCAUGCAUGCGGUUGGCCUAAAAGCGAGUCCUCGGCGACGCGCGCCACGACAAUCGGUGGUUGUCAAACCCUCGUGUCCCGUCGUGCGUGACGCGUCGCUCAUCGUGUGCUCCUUGACCCUGCUGUGUCGCGCUAGCGACGCUUCCAACGCGA

...............((.((.....(((((((((.((((.(((((((....))))).)).))))..)).))))))).....((((..(((((.((....)).)))))..)))).....((((......((((((((((((((((((...(((.((((....)))).)))......)))))).))).)))))))))..))))..................(((((((....)))))))......)).))

>MG234781.1 *Betula* *michauxii* voucher CCDB-18297-D07 5.8S ribosomal RNA gene, partial sequence; internal transcribed spacer 2, complete sequence; and large subunit ribosomal RNA gene, partial sequence

ACGUCUGCCUGGGUGUCACGCAUCGUUGCCCCCAACCCCAUCUCCUUGCAAAGGGACGAGGGGGCCUGUGGGGCAGAAAUUGGCCUCCCGUGAGCUCAUGCAUGCGGUUGGCCUAAAAGCGAGUUCUCGGCGACGCGCGCCACGACAAUCGGUGGUUGACAAACCCUGGUGUCCCGUCGUGCGUGCCGCGUCGCUCAUCGUGUGCUCUUUGACCCUGUUGUGUCGCGCUAGCGACGCUUCCAACGCGA

...............((.((.....(((((((((.((((.(((((((....))))).)).))))..)).))))))).....((((..(((((.((....)).)))))..)))).....((((......((((((((((((((((((...(((.((((....)))).)))......)))))).))).)))))))))..))))..................(((((((....)))))))......)).))

>MG234627.1 *Betula* kenaica voucher CCDB-18325-C1 5.8S ribosomal RNA gene, partial sequence; internal transcribed spacer 2, complete sequence; and large subunit ribosomal RNA gene, partial sequence

ACGUCUGCCUGGGUGUCACGCAUCGUUGCCCCCAACCCCAUCUCCUUGCAAAGGGACGAGGGGGCCUGUGGGGCAGAAAUUGGCCUCCCGUGAGCUCAUGCAUGCGGUUGGCCUAAAAGCGAGUCCUCGGCGACGCGCGCCACGACAAUCGGUGGUUGUCAAACCCUCGUGUCCCGUCGUGCGUGCCGCGUCGCUCAUCGUGUGCUCCUUGACCCUGCUGUGUCGCGCUAGCGACGCUUCCAACGCGA

...............((.((.....(((((((((.((((.(((((((....))))).)).))))..)).))))))).....((((..(((((.((....)).)))))..)))).....((((......((((((((((((((((((...(((.((((....)))).)))......)))))).))).)))))))))..))))..................(((((((....)))))))......)).))

>MG234564.1 *Betula* *lenta* voucher CCDB-18297-C07 5.8S ribosomal RNA gene, partial sequence; internal transcribed spacer 2, complete sequence; and large subunit ribosomal RNA gene, partial sequence

ACGUCUGCCUGGGUGUCACGCAUCGUUGCCCCCAACCCCAUCUCCUUGCAAAGGGACGAGGGGGCCUGUGGGGCAGAAAUUGGCCUCCCGUGAGCUCAUGCAUGCGGUUGGCCUAAAAGCGAGUCCUCGGCGACGCGCGCCACGACAAUCGGUGGUUGACAAACCCUCGUGUCCCGUCGUGCGUGCCGCGUCGCUCAUCGUGUGCUCUUUGACCCUGCUGUGUCGCGCCAGCGACGCUUCCAACGCGA

...............((.((.....(((((((((.((((.(((((((....))))).)).))))..)).))))))).....((((..(((((.((....)).)))))..)))).....((((......((((((((((((((((((...(((.((((....)))).)))......)))))).))).)))))))))..))))..................(((((((....)))))))......)).))

>MH042914|*Betula* *atrata* 031470

ACGUCUGCCUGGGUGUCACGCAUCGUUGCCCCCAACCCCAUCUCCUUGCAAAGGGACGAGGGGGCCUGUGGGGCAGAAAUUGGCCUCCCGUGAGCUCAUGCAUGCGGUUGGCCUAAAAGCGAGUCCUCGGCGACGCGCGCCACGACAAUCGGUGGUUGUCAAACCCUCGUGUCCCGUCGUGCGUGCCGCGUCGCUCAUCGUGUGCUCCUUGACCCUGCUGUGUCGCGCUAGCGACGCUUCCAACGCGA

...............((.((.....(((((((((.((((.(((((((....))))).)).))))..)).))))))).....((((..(((((.((....)).)))))..)))).....((((......((((((((((((((((((...(((.((((....)))).)))......)))))).))).)))))))))..))))..................(((((((....)))))))......)).))

>MH014808|*Betula*_*borysthenica* KW0064215 type

ACGUCUGCCUGGGUGUCACGCAUCGUUGCCCCCAACCCCAUCUCCUUGCAAAGGGACGAGGGGGCCUGUGGGGCAGAAAUUGGCCUCCCGUGAGCUCAUGCAUGCGGUUGGCCUAAAAGCGAGUCCUCGGCGACGCGCGCCACGACAAUCGGUGGUUGUCAAACCCUCGUGUCCCGUCGUGCGUGCCGCGUCGCUCCUCGUGUGCUCCUUGACCCUGCUGCGUCGCGCUAGCGACGCUUCCAACGCGA

...............((.((.....(((((((((.((((.(((((((....))))).)).))))..)).))))))).....((((..(((((.((....)).)))))..)))).....((((......((((((((((((((((((...(((.((((....)))).)))......)))))).))).)))))))))..))))..................(((((((....)))))))......)).))

>MH300135|*Betula* *klokovii* KW128023

ACGUCUGCCUGGGUGUCACGCAUCGUUGCCCCCAACCCCAUCUCCUUGCAAAGGGACGAGGGGGCCUGUGGGGCAGAAAUUGGCCUCCCGUGAGCUCAUGCAUGCGGUUGGCCUAAAAGCGAGUCCUCGGCGACGCGCGCCACGACAAUCGGUGGUUGUCAAACCCUCGUGUCCCGUCGUGCGUGCCGCGUCGCUCAUCGUGUGCUCCUUGACCCUGCUGUGUCGCGCUAGCGACGCUUCCAACGCGA

...............((.((.....(((((((((.((((.(((((((....))))).)).))))..)).))))))).....((((..(((((.((....)).)))))..)))).....((((......((((((((((((((((((...(((.((((....)))).)))......)))))).))).)))))))))..))))..................(((((((....)))))))......)).))

>MH042917|*Betula* *kotulae* KW006422 type

ACGUCUGCCUGGGUGUCACGCAUCGUUGCCCCCAACCCCAUCUCCUUGCAAAGGGACGAGGGGGCCUGUGGGGCAGAAAUUGGCCUCCCGUGAGCUCAUGCAUGCGGUUGGCCUAAAAGCGAGUCCUCGGCGACGCGCGCCACGACAAUCGGUGGUUGUCAAACCCUCGUGUCCCGUCGUGCGUGCCGCGUCGCUCAUCGUGUGCUCCUUGACCCUGCUGUGUCGCGCUAGCGACGCUUCCAACGCGA

...............((.((.....(((((((((.((((.(((((((....))))).)).))))..)).))))))).....((((..(((((.((....)).)))))..)))).....((((......((((((((((((((((((...(((.((((....)))).)))......)))))).))).)))))))))..))))..................(((((((....)))))))......)).))

>MH042912|*Betula* *kotulae* KW008349

ACGUCUGCCUGGGUGUCACGCAUCGUUGCCCCCAACCCCAUCUCCUUGCAAAGGGACGAGGGGGCCUGUGGGGCAGAAAUUGGCCUCCCGUGAGCUCAUGCAUGCGGUUGGCCUAAAAGCGAGUCCUCGGCGACGCGCGCCACGACAAUCGGUGGUUGUCAAACCCUCGUGUCCCGUCGUGCGUGCCGCGUCGCUCAUCGUGUGCUCCUUGACCCUGCUGUGUCGCGCUAGCGACGCUUCCAACGCGA

...............((.((.....(((((((((.((((.(((((((....))))).)).))))..)).))))))).....((((..(((((.((....)).)))))..)))).....((((......((((((((((((((((((...(((.((((....)))).)))......)))))).))).)))))))))..))))..................(((((((....)))))))......)).))

>MH178103|*Betula* *kotulae* KW008349

ACGUCUGCCUGGGUGUCACGCAUCGUUGCCCCCAACCCCAUCUCCUUGCAAAGGGACGAGGGGGCCUGUGGGGCAGAAAUUGGCCUCCCGUGAGCUCAUGCAUGCGGUUGGCCUAAAAGCGAGUCCUCGGCGACGCGCGCCACGACAAUCGGUGGUUGUCAAACCCUCGUGUCCCGUCGUGCGUGCCGCGUCGCUCAUCGUGUGCUCCUUGACCCUGCUGUGUCGCGCUAGCGACGCUUCCAACGCGA

...............((.((.....(((((((((.((((.(((((((....))))).)).))))..)).))))))).....((((..(((((.((....)).)))))..)))).....((((......((((((((((((((((((...(((.((((....)))).)))......)))))).))).)))))))))..))))..................(((((((....)))))))......)).))

>MH042913|*Betula* *kotulae* KW128013

ACGUCUGCCUGGGUGUCACGCAUCGUUGCCCCCAACCCCAUCUCCUUGCAAAGGGACGAGGGGGCCUGUGGGGCAGAAAUUGGCCUCCCGUGAGCUCAUGCAUGCGGUUGGCCUAAAAGCGAGUCCUCGGCGACGCGCGCCACGACAAUCGGUGGUUGUCAAACCCUCGUGUCCCGUCGUGCGUGCCGCGUCGCUCAUCGUGUGCUCCUUGACCCUGCUGUGUCGCGCUAGCGACGCUUCCAACGCGA

...............((.((.....(((((((((.((((.(((((((....))))).)).))))..)).))))))).....((((..(((((.((....)).)))))..)))).....((((......((((((((((((((((((...(((.((((....)))).)))......)))))).))).)))))))))..))))..................(((((((....)))))))......)).))

>MH178105|*Betula* *kotulae* KW128013

ACGUCUGCCUGGGUGUCACGCAUCGUUGCCCCCAACCCCAUCUCCUUGCAAAGGGACGAGGGGGCCUGUGGGGCAGAAAUUGGCCUCCCGUGAGCUCAUGCAUGCGGUUGGCCUAAAAGCGAGUCCUCGGCGACGCGCGCCACGACAAUCGGUGGUUGUCAAACCCUCGUGUCCCGUCGUGCGUGCCGCGUCGCUCAUCGUGUGCUCCUUGACCCUGCUGUGUCGCGCUAGCGACGCUUCCAACGCGA

...............((.((.....(((((((((.((((.(((((((....))))).)).))))..)).))))))).....((((..(((((.((....)).)))))..)))).....((((......((((((((((((((((((...(((.((((....)))).)))......)))))).))).)))))))))..))))..................(((((((....)))))))......)).))

>MH042916|*Betula* *kotulae* LW006898

ACGUCUGCCUGGGUGUCACGCAUCGUUGCCCCCAACCCCAUCUCCUUGCAAAGGGACGAGGGGGCCKGUGGGGCAGAAAUUGGCCUCCCGUGAGCUCAUGCAUGCGGUUGGCCUAAAAGCGAGUCCUCGGCGACGCGCGCCACGACAAUCGGUGGUUGUCAAACCCUCGUGUCCCGUCGUGCGUGCCGCGUCGCUCAUCGUGUGCUCCUUGACCCUGCUGUGUCGCGCUAGCGACGCUUCCAACGCGA

...............((.((.....(((((((((.((((.(((((((....))))).)).))))..)).))))))).....((((..(((((.((....)).)))))..)))).....((((......((((((((((((((((((...(((.((((....)))).)))......)))))).))).)))))))))..))))..................(((((((....)))))))......)).))

>MH178104|*Betula* *kotulae* KW06427

ACGUCUGCCUGGGUGUCACGCAUCGUUGCCCCCAACCCCAUCUCCUUGCAAAGGGACGAGGGGGCCUGUGGGGCAGAAAUUGGCCUCCCGUGAGCUCAUGCAUGCGGUUGGCCUAAAAGCGAGUCCUCGGCGACGCGCGCCACGACAAUCGGUGGUUGUCAAACCCUCGUGUCCCGUCGUGCGUGCCGCGUCGCUCAUCGUGUGCUCCUUGACCCUGCUGUGUCGCGCUAGCGACGCUUCCAACGCGA

...............((.((.....(((((((((.((((.(((((((....))))).)).))))..)).))))))).....((((..(((((.((....)).)))))..)))).....((((......((((((((((((((((((...(((.((((....)))).)))......)))))).))).)))))))))..))))..................(((((((....)))))))......)).))

>MH178106|*Betula* *kotulae* KW128014

ACGUCUGCCUGGGUGUCACGCAUCGUUGCCCCCAACCCCAUCUCCUUGCAAAGGGACGAGGGGGCCUGUGGGGCAGAAAUUGGCCUCCCGUGAGCUCAUGCAUGCGGUUGGCCUAAAAGCGAGUCCUCGGCGACGCGCGCCACGACAAUCGGUGGUUGUCAAACCCUCGUGUCCCGUCGUGCGUGCCGCGUCGCUCAUCGUGUGCUCCUUGACCCUGCUGUGUCGCGCUAGCGACGCUUCCAACGCGA

...............((.((.....(((((((((.((((.(((((((....))))).)).))))..)).))))))).....((((..(((((.((....)).)))))..)))).....((((......((((((((((((((((((...(((.((((....)))).)))......)))))).))).)))))))))..))))..................(((((((....)))))))......)).))

>MH178107|*Betula* *kotulae* KW128016

ACGUCUGCCUGGGUGUCACGCAUCGUUGCCCCCAACCCCAUCUCCUUGCAAAGGGACGAGGGGGCCUGUGGGGCAGAAAUUGGCCUCCCGUGAGCUCAUGCAUGCGGUUGGCCUAAAAGCGAGUCCUCGGCGACGCGCGCCACGACAAUCGGUGGUUGUCAAACCCUCGUGUCCCGUCGUGCGUGCCGCGUCGCUCAUCGUGUGCUCCUUGACCCUGCUGUGUCGCGCUAGCGACGCUUCCAACGCGA

...............((.((.....(((((((((.((((.(((((((....))))).)).))))..)).))))))).....((((..(((((.((....)).)))))..)))).....((((......((((((((((((((((((...(((.((((....)))).)))......)))))).))).)))))))))..))))..................(((((((....)))))))......)).))

>MH178108|*Betula* *kotulae* KW128018

ACGUCUGCCUGGGUGUCACGCAUCGUUGCCCCCAACCCCAUCUCCUUGCAAAGGGACGAGGGGGCCUGUGGGGCAGAAAUUGGCCUCCCGUGAGCUCAUGCAUGCGGUUGGCCUAAAAGCGAGUCCUCGGCGACGCGCGCCACGACAAUCGGUGGUUGUCAAACCCUCGUGUCCCGUCGUGCGUGCCGCGUCGCUCAUCGUGUGCUCCUUGACCCUGCUGUGUCGCGCUAGCGACGCUUCCAACGCGA

...............((.((.....(((((((((.((((.(((((((....))))).)).))))..)).))))))).....((((..(((((.((....)).)))))..)))).....((((......((((((((((((((((((...(((.((((....)))).)))......)))))).))).)))))))))..))))..................(((((((....)))))))......)).))

>MH178109|*Betula* *kotulae* KW128022

ACGUCUGCCUGGGUGUCACGCAUCGUUGCCCCCAACCCCAUCUCCUUGCAAAGGGACGAGGGGGCCUGUGGGGCAGAAAUUGGCCUCCCGUGAGCUCAUGCAUGCGGUUGGCCUAAAAGCGAGUCCUCGGCGACGCGCGCCACGACAAUCGGUGGUUGUCAAACCCUCGUGUCCCGUCGUGCGUGCCGCGUCGCUCAUCGUGUGCUCCUUGACCCUGUUGUGUCGCGCUAGCGACGCUUCCAACGCGA

...............((.((.....(((((((((.((((.(((((((....))))).)).))))..)).))))))).....((((..(((((.((....)).)))))..)))).....((((......((((((((((((((((((...(((.((((....)))).)))......)))))).))).)))))))))..))))..................(((((((....)))))))......)).))

>MH042918|*Betula* *kotulae* (live)

ACGUCUGCCUGGGUGUCACGCAUCGUUGCCCCCAACCCCAUCUCCUUGCAAAGGGACGAGGGGGCCUGUGGGGCAGAAAUUGGCCUCCCGUGAGCUCAUGCAUGCGGUUGGCCUAAAAGCGAGUCCUCGGCGACGCGCGCCACGACAAUCGGUGGUUGUCAAACCCUCGUGUCCCGUCGUGCGUGCCGCGUCGCUCAUCGUGUGCUCCUUGACCCUGCUGUGUCGCGCUAGCGACGCUUCCAACGCGA

...............((.((.....(((((((((.((((.(((((((....))))).)).))))..)).))))))).....((((..(((((.((....)).)))))..)))).....((((......((((((((((((((((((...(((.((((....)))).)))......)))))).))).)))))))))..))))..................(((((((....)))))))......)).))

>MH042919|*Betula* *kotulae* (live)

ACGUCUGCCUGGGUGUCACGCAUCGUUGCCCCCAACCCCAUCUCCUUGCAAAGGGACGAGGGGGCCUGUGGGGCAGAAAUUGGCCUCCCGUGAGCUCAUGCAUGCGGUUGGCCUAAAAGCGAGUCCUCGGCGACGCGCGCCACGACAAUCGGUGGUUGUCAAACCCUCGUGUCCCGUCGUGCGUGCCGCGUCGCUCAUCGUGUGCUCCUUGACCCUGCUGUGUCGCGCUAGCGACGCUUCCAACGCGA

...............((.((.....(((((((((.((((.(((((((....))))).)).))))..)).))))))).....((((..(((((.((....)).)))))..)))).....((((......((((((((((((((((((...(((.((((....)))).)))......)))))).))).)))))))))..))))..................(((((((....)))))))......)).))

>MH014809| *Betula* *pubescens* ssp. *carpatica*

ACGUCUGCCUGGGUGUCACGCAUCGUUGCCCCCAACCCCAUCUCCUUGCAAAGGGACGAGGGGGCCUGUGGGGCAGAAAUUGGCCUCCCGUGAGCUCAUGCAUGCGGUUGGCCUAAAAGCGAGUCCUCGGCGACGCGCGCCACGACAAUCGGUGGUUGUCAAACCCUCGUGUCCCGUCGUGCGUGCCGCGUCGCUCAUCGUGUGCUCCUUGACCCUGCUGUGUCGCGCUAGCGACGCUUCCAACGCGA

...............((.((.....(((((((((.((((.(((((((....))))).)).))))..)).))))))).....((((..(((((.((....)).)))))..)))).....((((......((((((((((((((((((...(((.((((....)))).)))......)))))).))).)))))))))..))))..................(((((((....)))))))......)).))

>MH178101| *Betula* *pubescens* var. *sibakademica* LE01041130(R) type

ACGUCUGCCUGGGUGUCACGCAUCGUUGCCCCCAACCCCAUCUCCUUGCAAAGGGACGAGGGGGCCUGUGGGGCAGAAAUUGGCCUCCCGUGAGCUCAUGCAUGCGGUUGGCCUAAAAGCGAGUCCUCGGCGACGCGCGCCACGACAAUCGGUGGUUGUCAAACCCUCGUGUCCCGUCGUGCGUGCCGCGUCGCUCAUCGUGUGCUCCUUGACCCUGCUGCGUCGCGCUAGCGACGCUUCCAACGCGA

...............((.((.....(((((((((.((((.(((((((....))))).)).))))..)).))))))).....((((..(((((.((....)).)))))..)))).....((((......((((((((((((((((((...(((.((((....)))).)))......)))))).))).)))))))))..))))..................(((((((....)))))))......)).))

>MH178102|*Betula* *pubescens* var. *sibakademica* KW128024

ACGUCUGCCUGGGUGUCACGCAUCGUUGCCCCCAACCCCAUCUCCUUGCAAAGGGACGAGGGGGCCUGUGGGGCAGAAAUUGGCCUCCCGUGAGCUCAUGCAUGCGGUUGGCCUAAAAGCGAGUCCUCGGCGACGCGCGCCACGACAAUCGGUGGUUGUCAAACCCUCGUGUCCCGUCGUGCGUGCCGCGUCGCUCAUCGUGUGCUCCUUGACCCUGCUGCGUCGCGCUAGCGACGCUUCCAACGCGA

...............((.((.....(((((((((.((((.(((((((....))))).)).))))..)).))))))).....((((..(((((.((....)).)))))..)))).....((((......((((((((((((((((((...(((.((((....)))).)))......)))))).))).)))))))))..))))..................(((((((....)))))))......)).))

>MH042911|*Betula* *pubescens* var. *sibakademica* KW128024

ACGUCUGCCUGGGUGUCACGCAUCGUUGCCCCCAACCCCAUCUCCUUGCAAAGGGACGAGGGGGCCUGUGGGGCAGAAAUUGGCCUCCCGUGAGCUCAUGCAUGCGGUUGGCCUAAAAGCGAGUCCUCGGCGACGCGCGCCACGACAAUCGGUGGUUGUCAAACCCUCGUGUCCCGUCGUGCGUGCCGCGUCGCUCAUCGUGUGCUCCUUGACCCUGCUGCGUCGCGCUAGCGACGCUUCCAACGCGA

...............((.((.....(((((((((.((((.(((((((....))))).)).))))..)).))))))).....((((..(((((.((....)).)))))..)))).....((((......((((((((((((((((((...(((.((((....)))).)))......)))))).))).)))))))))..))))..................(((((((....)))))))......)).))

>MH042915|*Betula* *pubescens* var. *sibakademica* LWKS031322

ACGUCUGCCUGGGUGUCACGCAUCGUUGCCCCCAACCCCAUCUCCUUGCAAAGGGACGAGGGGGCCUGUGGGGCAGAAAUUGGCCUCCCGUGAGCUCAUGCAUGCGGUUGGCCUAAAAGCGAGUCCUCGGCGACGCGCGCCACGACAAUCGGUGGUUGUCAAACCCUCGUGUCCCGUCGUGCGUGCCGCGUCGCUCAUCGUGUGCUCCUUGACCCUGCUGCGUCGCGCUAGCGACGCUUCCAACGCGA

...............((.((.....(((((((((.((((.(((((((....))))).)).))))..)).))))))).....((((..(((((.((....)).)))))..)))).....((((......((((((((((((((((((...(((.((((....)))).)))......)))))).))).)))))))))..))))..................(((((((....)))))))......)).))
