# Supplementary material for: ITS secondary structure reconstruction to resolve taxonomy and phylogeny of the Betula L. genus
Source: PeerJ. 2021 Mar 23;9:e10889. doi: 10.7717/peerj.10889 (PMC7996101; doi:10.7717/peerj.10889)
Supplement: Supplemental Information 4 [file peerj-09-10889-s004.docx]

**Data S4.** Distribution of different structure variants in ITS1.

**ITS1 additional loop HA**
Structural variant 1 (A)

CCRGYAGAAYGACCCGUGAACYUGUUGA
.((((((.(((...)))....)))))).


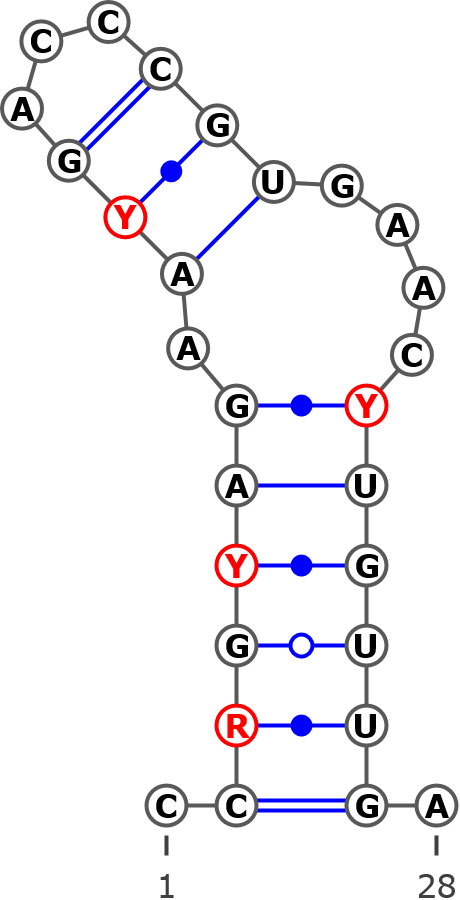


AB243915 *Betula apoiensis*, haplotype:ap17

AB243914 *Betula apoiensis*, haplotype:ap14

AB243913 *Betula apoiensis*, haplotype:ap16

AB243898 *Betula ovalifolia*, haplotype:ov3

AB243897 *Betula ovalifolia*, haplotype:ov2

AB243895 *Betula middendorffii*, haplotype:mi

KT309023 *Betula ovalifolia* isolate 2

KT309022 *Betula ovalifolia* isolate 1

KT309021 *Betula pumila*

KT308989 *Betula tianschanica*

KT308966 *Betula raddeana*

AY761132 *Betula raddeana* isolate 3338

AY761131 *Betula pumila* isolate 3246

KT309019 *Betula nana* subsp. *exilis*

AJ783644 *Betula populifolia*

AJ783643 *Betula humilis*

AJ783642 *Betula pumila*

AJ783641 *Betula alnoides*

AB243911 *Betula apoiensis*, haplotype:ap12

AB243910 *Betula apoiensis*, haplotype:ap10

AB243909 *Betula apoiensis*, haplotype:ap11

AJ006445 *Betula pendula*

AJ783646 *Betula nigra*

AJ783645 *Betula insignis*

AJ251683 *Betula alba*

AM503889.2| *Betula pendula*

AB243907 *Betula apoiensis*, haplotype:ap5

AB243906 *Betula apoiensis*, haplotype:ap9

AB243905 *Betula apoiensis*, haplotype:ap8

AB243904 *Betula apoiensis*, haplotype:ap7

AB243903 *Betula apoiensis*, haplotype:ap6

AB243902 *Betula apoiensis*, haplotype:ap3

AB243901 *Betula apoiensis*, haplotype:ap2

AB243900 *Betula apoiensis*, haplotype:ap1

AB243899 *Betula apoiensis*, haplotype:ap4

AB243894 *Betula davurica*, haplotype:da2

AB243893 *Betula davurica*, haplotype:da1

AB243892 *Betula grossa*, haplotype:gr

AB243891 *Betula platyphylla*, haplotype:pl

AB243890 *Betula maximovicziana*, haplotype:ma

AB243889 *Betula ermanii*, haplotype:er4

AB243888 *Betula ermanii*, haplotype:er3

AB243887 *Betula ermanii*, haplotype:er2

AB243886 *Betula ermanii*, haplotype:er1

AB243885 *Betula corylifolia*, haplotype:co2

AB243884 *Betula corylifolia*, haplotype:co1

AB243882 *Betula chichibuensis*, haplotype:ch2

AB243881 *Betula chichibuensis*, haplotype:ch1

AB243880 *Betula schmidtii*, haplotype:sc

KT309028 *Betula occidentalis* isolate 2

KT309027 *Betula occidentalis* isolate 1

KT309026 *Betula humilis* isolate 3

KT309025 *Betula humilis* isolate 2

KT309024 *Betula humilis* isolate 1

KT309020 *Betula nana* isolate 2

KT309018 *Betula nana* isolate 1

KT309017 *Betula glandulosa* isolate 2

KT309016 *Betula cordifolia* isolate 2

KT309015 *Betula cordifolia* isolate 1

KT309014 *Betula papyrifera* var. *commutata*

KT309013 *Betula papyrifera* isolate 3

KT309012 *Betula papyrifera* isolate 2

KT309011 *Betula papyrifera* isolate 1

KT309010 *Betula populifolia* isolate 3

KT309009 *Betula populifolia* isolate 2

KT309008 *Betula pendula* subsp. *mandshurica* isolate 5

KT309007 *Betula pendula* subsp. *pendula* isolate 6

KT309006 *Betula pendula* subsp. *pendula* isolate 5

KT309005 *Betula pendula* subsp. *mandshurica* isolate 4

KT309004 *Betula pendula* subsp. *szechuanica* isolate 3

KT309003 *Betula pendula* subsp. *szechuanica* isolate 2

KT309002 *Betula pendula* subsp. *pendula* isolate 4

KT309001 *Betula* *pendula* subsp. *pendula* isolate 3

KT309000 *Betula* *pendula* subsp. *pendula* isolate 2

KT308999 *Betula* *pendula* subsp. *mandshurica* isolate 3

KT308998 *Betula* *pendula* subsp. *pendula* isolate 1

KT308997 *Betula* *pendula* subsp. *szechuanica* isolate 1

KT308996 *Betula* *pendula* subsp. *mandshurica* isolate 2

KT308995 *Betula* *glandulosa* isolate 1

KT308994 *Betula* *populifolia* isolate 1

KT308993 *Betula* *obscura*

KT308992 *Betula* *turkestanica*

KT308991 *Betula* *pendula*

KT308990 *Betula* *pendula* subsp. *mandshurica* isolate 1

KT308988 *Betula × caerulea* isolate 2

KT308987 *Betula × caerulea* isolate 1

KT308986 *Betula* *middendorffii*

KT308985 *Betula* *minor*

KT308984 *Betula* *microphylla*

KT308983 *Betula* *pubescens* var. *litwinowii* isolate 2

KT308982 *Betula* *pubescens* var. *pubescens* isolate 4

KT308981 *Betula* *pubescens* var. *pubescens* isolate 3

KT308980 *Betula* *pubescens* var. *pumila* isolate 3

KT308979 *Betula × utahensis*

KT308978 *Betula* *michauxii*

KT308977 *Betula* *pubescens* subsp. *celtiberica* isolate 2

KT308976 *Betula* *pubescens* var. *pumila* isolate 2

KT308975 *Betula* *pubescens* var. fragans isolate 2

KT308974 *Betula* *pubescens* var. fragans isolate 1

KT308973 *Betula* *pubescens* var. *pumila* isolate 1

KT308972 *Betula* *pubescens* subsp. *celtiberica* isolate 1

KT308971 *Betula* *pubescens* var. litiwinowii isolate 1

KT308970 *Betula* *pubescens* var. *pubescens* isolate 2

KT308969 *Betula* *pubescens* var. *pubescens* isolate 1

KT308968 *Betula* *browicziana*

KT308967 *Betula* *halophila*

KT308965 *Betula* *nigra* isolate 2

KT308964 *Betula* *nigra* isolate 1

KT308963 *Betula* *dahurica* isolate 2

KT308962 *Betula* *dahurica* isolate 1

KT308961 *Betula* *ashburneri* isolate 3

KT308960 *Betula* *lanata* isolate 2

KT308959 *Betula* *lanata* isolate 1

KT308958 *Betula* *costata*

KT308957 *Betula* *ermanii* isolate 2

KT308956 *Betula* *ermanii* isolate 1

KT308955 *Betula* *utilis* var. *prattii*

KT308954 *Betula* *albosinensis* isolate 2

KT308953 *Betula* *ashburneri* isolate 2

KT308952 *Betula* *ashburneri* isolate 1

KT308951 *Betula* *utilis* var. jacquemontii

KT308950 *Betula* *utilis* var. *occidentalis* isolate 2

KT308949 *Betula* *utilis* isolate 2

KT308948 *Betula* *utilis* isolate 1

KT308947 *Betula* *albosinensis* var. septentrionalis

KT308946 *Betula* *maximovicziana* isolate 2

KT308945 *Betula* *maximovicziana* isolate 1

KT308943 *Betula* *luminifera* isolate 2

KT308942 *Betula* *hainanensis*

KT308941 *Betula* *cylindrostachya*

KT308940 *Betula* *alnoides*

KT308938 *Betula* *lenta* f. *uber* isolate 2

KT308937 *Betula* *lenta* f. *uber* isolate 1

KT308936 *Betula* *lenta*

KT308935 *Betula* *grossa* isolate 2

KT308934 *Betula* *grossa* isolate 1

KT308933 *Betula* *megrelica* isolate 2

KT308932 *Betula* *megrelica* isolate 1

KT308931 *Betula* *medwediewii* isolate 2

KT308930 *Betula* *medwediewii* isolate 1

KT308929 *Betula* *insignis* subsp. *fansipanensis*

KT308928 *Betula* *insignis* isolate 2

KT308927 *Betula* *insignis* isolate 1

KT308926 *Betula* *murrayana*

KT308925 *Betula* *alleghaniensis*

KT308924 *Betula* *albosinensis* isolate 1

KT308923 *Betula* *utilis* var. *occidentalis*

KT308921 *Betula* *delavayi* isolate 2

KT308920 *Betula* *schmidtii* isolate 2

KT308919 *Betula* *schmidtii* isolate 1

KT308916 *Betula* *chichibuensis* isolate 2

KT308915 *Betula* *chichibuensis* isolate 1

KT308914 *Betula* *calcicola*

KT308913 *Betula* *delavayi* isolate 1

KT308912 *Betula* *bomiensis* isolate 2

KT308911 *Betula* *bomiensis* isolate 1

KT308910 *Betula* *potaninii* isolate 2

KT308909 *Betula* *potaninii* isolate 1

KT308908 *Betula* *corylifolia* isolate 2

KT308907 *Betula* *corylifolia* isolate 1

JN247411 *Betula* *pendula* voucher MCA 221

FJ011780 *Betula* *utilis* voucher MacAtrher-Tibet Expedition452

FJ011779 *Betula* *schmidtii* Lee s.n.

FJ011778 *Betula* *platyphylla* Lee s.n.

FJ011777 *Betula* *pendula* voucher CS03022

FJ011773 *Betula* *davurica* voucher Tibet218

FJ011772 *Betula* *davurica* voucher UNA63303V

FJ011771 *Betula* *davurica* voucher UNA64631H

FJ011770 *Betula* *davurica* voucher Lee s.n.

AY763113 *Betula* *luminifera* isolate 2841

AY761134 *Betula* *utilis* isolate 2893

AY761133 *Betula* *schmidtii* isolate 2875

AY761130 *Betula* *pubescens* isolate 2895

AY761129 *Betula* *populifolia* isolate 2890

AY761128 *Betula* *platyphylla* isolate 2934

AY761127 *Betula* *pendula* isolate 2902

AY761126 *Betula* *papyrifera* isolate 2892

AY761125 *Betula* *occidentalis* isolate 2883

AY761124 *Betula* *nigra* isolate 2927

AY761122 *Betula* *nana* isolate 3337

AY761121 *Betula* *michauxii* isolate 3406

AY761119 *Betula* *maximovicziana* isolate 3463

AY761118 *Betula* *maximovicziana* isolate 2932

AY761117 *Betula* *luminifera* isolate 3299

AY761116 *Betula* *luminifera* isolate 2828

AY761114 *Betula* *humilis* isolate 2894

AY761113 *Betula* *grossa* isolate 3459

AY761112 *Betula* *grossa* isolate 2948

AY761109 *Betula* *fruticosa* isolate 3339

AY761108 *Betula* *ermanii* isolate 2961

AY761107 *Betula* *delavayi* isolate 3462

AY761106 *Betula* *corylifolia* isolate 3457

AY761105 *Betula* *chinensis* isolate 2903

AY761104 *Betula* *chichibuensis* isolate 2977

AY761103 *Betula* *calcicola* isolate 3460

AY761102 *Betula* *apoiensis* isolate 3249

AY761101 *Betula* *alnoides* isolate 3352

AY761100 *Betula* *alleghaniensis* isolate 2880

AY761099 *Betula* *albosinensis* isolate 3018

AY352337 *Betula* *costata*

AY352336 *Betula* *nana*

AY352335 *Betula* *davurica*

AY352333 *Betula* *populifolia*

AY352332 *Betula* *pendula*

AY352331 *Betula* *nigra*

AF432067 *Betula* *papyrifera*

DQ397523 *Betula* *occidentalis*

MH231211 *Betula* *atrata*

MH014808 *Betula* *borysthenica* (KW0061415, holotype)

MH238480 *Betula* *klokovii* (KW006422, holotype)

MH231207 *Betula* *kotulae* (KW006426, holotype)

MH178103 *Betula* *kotulae* (KW008349)

MH178104 *Betula* *kotulae* (KW06427)

MH178105 *Betula* *kotulae* (KW128013)

MH178106 *Betula* *kotulae* (KW128014)

MH178107 *Betula* *kotulae* (KW128016)

MH178108 *Betula* *kotulae* (KW128018)

MH231208 *Betula* *kotulae* (KW128019)

MH231209 *Betula* *kotulae* (KW128020)

MH178109 *Betula* *kotulae* (KW128022)

MH231210 *Betula* *kotulae* (KW128023)

MH238474 *Betula* *kotulae*

MH238475 *Betula* *kotulae* (LW032758)

MH231213 *Betula* *kotulae* (LW006898)

MH238478 *Betula* *kotulae* (LWS27022)

MH238477 *Betula* *kotulae* (LWS27026)

MH231214 *Betula* *kotulae*

MH238476 *Betula* *oycowiensis* (LWKS031322)

MH014809 *Betula* *pubescens* ssp. *carpatica*

MH231206 *Betula* *pubescens* var. *sibakademica* (LE01041130, type)

MH238471 *Betula* *pubescens* var. *sibakademica* (KW128012)

MH178102 *Betula* *pubescens* var. *sibakademica* (KW128024)

MH238472 *Betula* *pubescens* var. *sibakademica*

MH238473 *Betula* *pubescens* var. *sibakademica*

MH231212 *Betula* *pubescens* var. *sibakademica* (LWKS031322)

Structural variant 2 (B)

CCAGCAGAACGACCCGUGAACAUGUUGA
.(((((..(((...))).....))))).


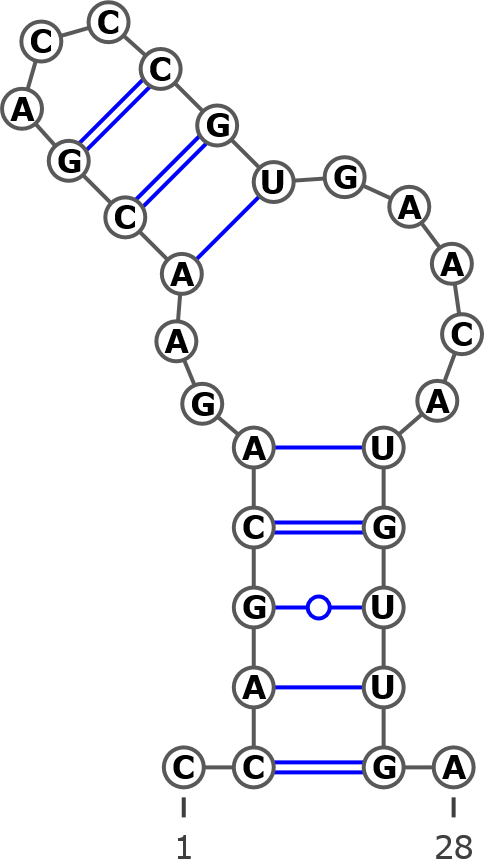


AB243883 *Betula* *globispica*, haplotype:gl

KT308918 *Betula* *chinensis* isolate 2

KT308917 *Betula* *chinensis* isolate 1

KT308906 *Betula* *fargesii*

KT308905 *Betula* *globispica* isolate 2

KT308904 *Betula* *globispica* isolate 1

AY761120 *Betula* *medwediewii* isolate 3465

AY761111 *Betula* *globispica* isolate 2942

AY761110 *Betula* *glandulosa* isolate 3251

AY761115 *Betula* *lenta* isolate 2936

AY352334 *Betula* *uber*

AY352330 *Betula* *lenta*

Structural variant 3 (C)

CCAGCAGAACGACCCGCGAACCUGUUGA
.((((((..((...)).....)))))).


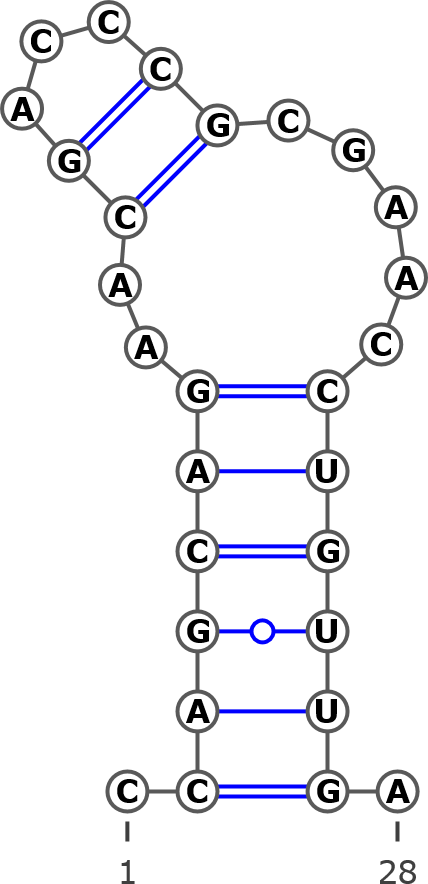


KT308944 *Betula* *luminifera* isolate 3

AY763114 *Betula* *alnoides* isolate 3464

Structural variant 4 (D)

CAGCAGAACGACCCGUGAACCUGUUS
.(((((.(((...)))....))))).


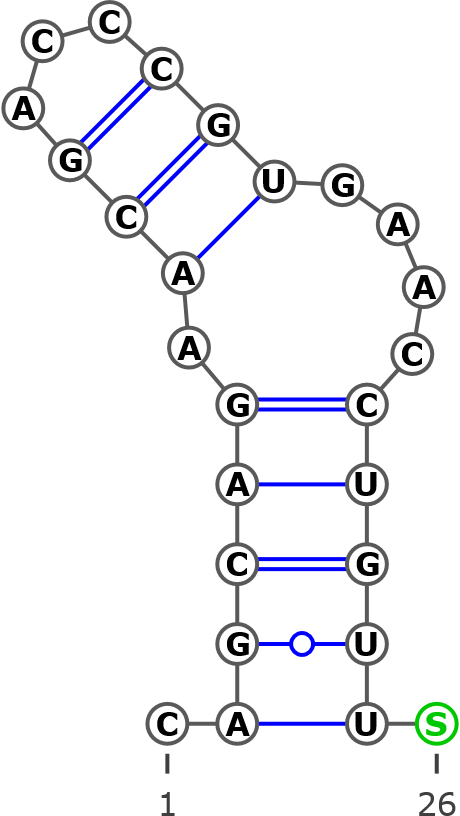


AB243912 *Betula* *apoiensis*, haplotype:ap15

AB243908 *Betula* *apoiensis*, haplotype:ap13

**Helix 1**

Structural variant 1 (A)

CUGGGGGYGGGGGGCGAUCUYGCCCCUUGYCCYCGA
.(((((((((((((((....))))))))))))))).


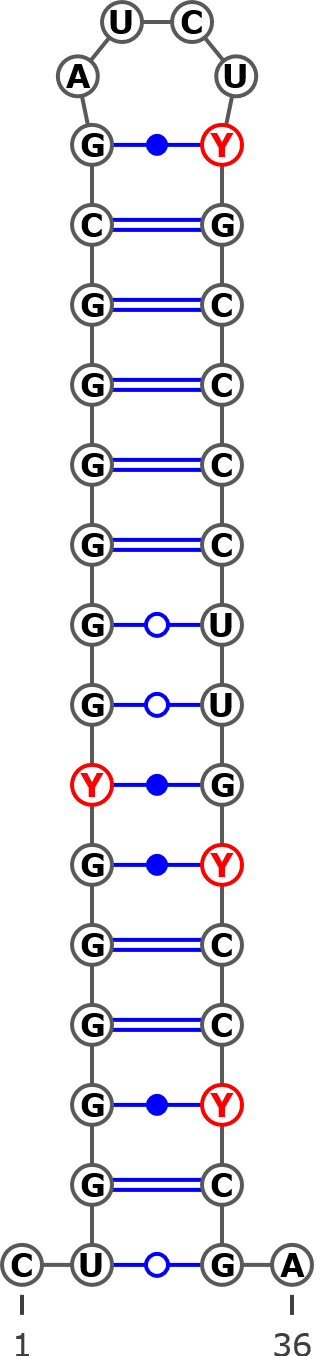


AJ783644 *Betula* *populifolia*

AJ783643 *Betula* *humilis*

AJ783642 *Betula* *pumila*

AJ783641 *Betula* *alnoides*

AB243911 *Betula* *apoiensis*, haplotype:ap12

AB243910 *Betula* *apoiensis*, haplotype:ap10

AB243909 *Betula* *apoiensis*, haplotype:ap11

AB243908 *Betula* *apoiensis*, haplotype:ap13

AB243906 *Betula* *apoiensis*, haplotype:ap9

AB243905 *Betula* *apoiensis*, haplotype:ap8

AB243904 *Betula* *apoiensis*, haplotype:ap7

AB243903 *Betula* *apoiensis*, haplotype:ap6

AB243902 *Betula* *apoiensis*, haplotype:ap3

AB243901 *Betula* *apoiensis*, haplotype:ap2

AB243900 *Betula* *apoiensis*, haplotype:ap1

AB243893 *Betula* *davurica*, haplotype:da1

AB243892 *Betula* *grossa*, haplotype:gr

AB243890 *Betula* *maximovicziana*, haplotype:ma

AB243888 *Betula* *ermanii*, haplotype:er3

AB243887 *Betula* *ermanii*, haplotype:er2

AB243886 *Betula* *ermanii*, haplotype:er1

KT309027 *Betula* *occidentalis* isolate 1

KT309026 *Betula* *humilis* isolate 3

KT309025 *Betula* *humilis* isolate 2

KT309024 *Betula* *humilis* isolate 1

KT309020 *Betula* *nana* isolate 2

KT309018 *Betula* *nana* isolate 1

KT309017 *Betula* *glandulosa* isolate 2

KT309016 *Betula* *cordifolia* isolate 2

KT309015 *Betula* *cordifolia* isolate 1

KT309014 *Betula* *papyrifera* var. *commutata*

KT309012 *Betula* *papyrifera* isolate 2

KT309011 *Betula* *papyrifera* isolate 1

KT309010 *Betula* *populifolia* isolate 3

KT309009 *Betula* *populifolia* isolate 2

KT308995 *Betula* *glandulosa* isolate 1

KT308994 *Betula* *populifolia* isolate 1

KT308988 *Betula × caerulea* isolate 2

KT308987 *Betula × caerulea* isolate 1

KT308986 *Betula* *middendorffii*

KT308985 *Betula* *minor*

KT308984 *Betula* *microphylla*

KT308983 *Betula* *pubescens* var. *litwinovii* isolate 2

KT308982 *Betula* *pubescens* var. *pubescens* isolate 4

KT308981 *Betula* *pubescens* var. *pubescens* isolate 3

KT308980 *Betula* *pubescens* var. *pumila* isolate 3

KT308979 *Betula × utahensis*

KT308977 *Betula* *pubescens* subsp. *celtiberica* isolate 2

KT308976 *Betula* *pubescens* var. *pumila* isolate 2

KT308975 *Betula* *pubescens* var. fragans isolate 2

KT308974 *Betula* *pubescens* var. fragans isolate 1

KT308973 *Betula* *pubescens* var. *pumila* isolate 1

KT308972 *Betula* *pubescens* subsp. *celtiberica* isolate 1

KT308971 *Betula* *pubescens* var. *litwinowii* isolate 1

KT308970 *Betula* *pubescens* var. *pubescens* isolate 2

KT308969 *Betula* *pubescens* var. *pubescens* isolate 1

KT308967 *Betula* *halophila*

KT308963 *Betula* *dahurica* isolate 2

KT308962 *Betula* *dahurica* isolate 1

KT308961 *Betula* *ashburneri* isolate 3

KT308960 *Betula* *lanata* isolate 2

KT308959 *Betula* *lanata* isolate 1

KT308955 *Betula* *utilis* var. *prattii*

KT308954 *Betula* *albosinensis* isolate 2

KT308953 *Betula* *ashburneri* isolate 2

KT308952 *Betula* *ashburneri* isolate 1

KT308951 *Betula* *utilis* var. jacquemontii

KT308950 *Betula* *utilis* var. *occidentalis* isolate 2

KT308949 *Betula* *utilis* isolate 2

KT308948 *Betula* *utilis* isolate 1

KT308947 *Betula* *albosinensis* var. septentrionalis

KT308946 *Betula* *maximovicziana* isolate 2

KT308945 *Betula* *maximovicziana* isolate 1

KT308936 *Betula* *lenta*

KT308935 *Betula* *grossa* isolate 2

KT308934 *Betula* *grossa* isolate 1

KT308924 *Betula* *albosinensis* isolate 1

KT308923 *Betula* *utilis* var. *occidentalis* isolate 1

FJ011780 *Betula* *utilis* voucher MacAtrher-Tibet Expedition 452

FJ011773 *Betula* *davurica* voucher Tibet218

FJ011772 *Betula* *davurica* voucher UNA63303V

FJ011771 *Betula* *davurica* voucher UNA64631H

FJ011770 *Betula* *davurica* voucher Lee s.n.

AY761134 *Betula* *utilis* isolate 2893

AY761130 *Betula* *pubescens* isolate 2895

AY761129 *Betula* *populifolia* isolate 2890

AY761126 *Betula* *papyrifera* isolate 2892

AY761119 *Betula* *maximovicziana* isolate 3463

AY761118 *Betula* *maximovicziana* isolate 2932

AY761113 *Betula* *grossa* isolate 3459

AY761112 *Betula* *grossa* isolate 2948

AY761109 *Betula* *fruticosa* isolate 3339

AY761108 *Betula* *ermanii* isolate 3339

AY761106 *Betula* *corylifolia* isolate 3457

AY761105 *Betula* *chinensis* isolate 2903

AY761102 *Betula* *apoiensis* isolate 3249

AY761099 *Betula* *albosinensis* isolate 3018

AY352337 *Betula* *costata*

AY352335 *Betula* *davurica*

AF432067 *Betula* *papyrifera*

MH014808 *Betula* *borysthenica* (KW0061415, holotype)

MH238480 *Betula* *klokovii* (KW006422, holotype)

MH238476| *Betula* *oycowiensis* (LW032763)

MH231206 *Betula* *pubescens* var. *sibakademica* (LE01041130, type)

MH238471 *Betula* *pubescens* var. *sibakademica* (KW128012)

MH178102 *Betula* *pubescens* var. *sibakademica* (KW128024)

MH238472 *Betula* *pubescens* var. *sibakademica*

MH238473 *Betula* *pubescens* var. *sibakademica*

MH231212 *Betula* *pubescens* var. *sibakademica* (LWKS031322)

Structural variant 2 (B)

CUGGGGGYRKGGGGCGWUCUCGCCCCKUGYCYYCGA
.((((((((.((((((....)))))).)))))))).


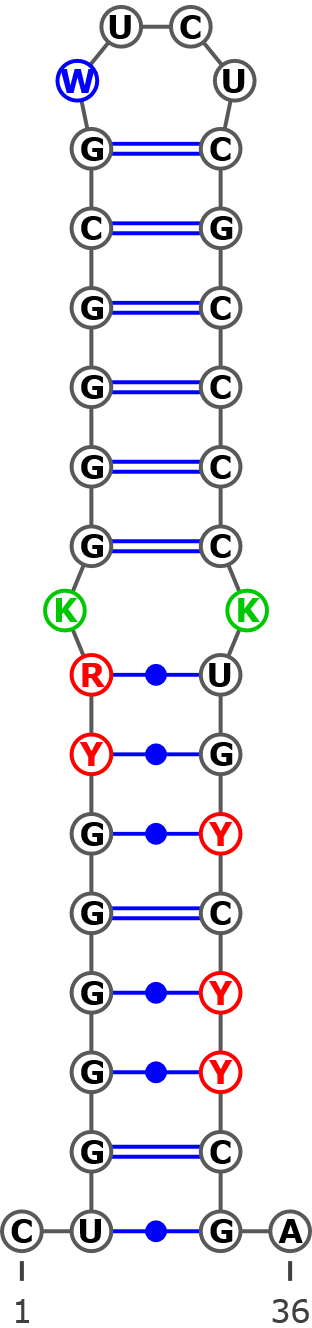


MH014809 *Betula* *pubescens* ssp. *carpatica*

AJ006445 *Betula* *pendula*

AJ783646 *Betula* *nigra*

AJ783645 *Betula* *insignis*

AJ251683 *Betula* *alba*

AM503889.2 *Betula* *pendula*

AB243907 *Betula* *apoiensis*, haplotype:ap5

AB243899 *Betula* *apoiensis*, haplotype:ap4

AB243889 *Betula* *ermanii*, haplotype:er4

AB243891 *Betula* *platyphylla*, haplotype:pl

AB243885 *Betula* *corylifolia*, haplotype:co2

AB243884 *Betula* *corylifolia*, haplotype:co1

AB243883 *Betula* *globispica*, haplotype:gl

AB243882 *Betula* *chichibuensis*, haplotype:ch2

AB243881 *Betula* *chichibuensis*, haplotype:ch1

AB243880 *Betula* *schmidtii*, haplotype:sc

KT309028 *Betula* *occidentalis* isolate 2

KT309013 *Betula* *papyrifera* isolate 3

KT309008 *Betula* *pendula* subsp. *mandshurica* isolate 5

KT309007 *Betula* *pendula* subsp. *pendula* isolate 6

KT309006 *Betula* *pendula* subsp. *pendula* isolate 5

KT309005 *Betula* *pendula* subsp. *mandshurica* isolate 4

KT309004 *Betula* *pendula* subsp. *szechuanica* isolate 3

KT309003 *Betula* *pendula* subsp. *szechuanica* isolate 2

KT309002 *Betula* *pendula* subsp. *pendula* isolate 4

KT309001 *Betula* *pendula* subsp. *pendula* isolate 3

KT309000 *Betula* *pendula* subsp. *pendula* isolate 2

KT308999 *Betula* *pendula* subsp. *mandshurica* isolate 3

KT308998 *Betula* *pendula* subsp. *pendula* isolate 1

KT308997 *Betula* *pendula* subsp. *szechuanica* isolate 1

KT308996 *Betula* *pendula* subsp. *mandshurica* isolate 2

KT308993 *Betula* *obscura*

KT308992 *Betula* *turkestanica*

KT308991 *Betula* *pendula* subsp. *mandshurica* isolate 1

KT308990 *Betula* *pendula*

KT308978 *Betula* *michauxii*

KT308968 *Betula* *browicziana*

KT308965 *Betula* *nigra* isolate 2

KT308964 *Betula* *nigra* isolate 1

KT308958 *Betula* *costata*

KT308957 *Betula* *ermanii* isolate 2

KT308956 *Betula* *ermanii* isolate 1

KT308944 *Betula* *luminifera* isolate 3

KT308943 *Betula* *luminifera* isolate 2

KT308942 *Betula* *hainanensis*

KT308941 *Betula* *cylindrostachya*

KT308940 *Betula* *alnoides*

KT308938 *Betula* *lenta* f. *uber* isolate 2

KT308937 *Betula* *lenta* f. *uber* isolate 1

KT308933 *Betula* *megrelica* isolate 2

KT308932 *Betula* *megrelica* isolate 1

KT308931 *Betula* *medwediewii* isolate 2

KT308930 *Betula* *medwediewii* isolate 1

KT308929 *Betula* *insignis* subsp. *fansipanensis*

KT308928 *Betula* *insignis* isolate 2

KT308927 *Betula* *insignis* isolate 1

KT308926 *Betula* *murrayana*

KT308925 *Betula* *alleghaniensis*

KT308921 *Betula* *delavayi* isolate 2

KT308920 *Betula* *schmidtii* isolate 2

KT308919 *Betula* *schmidtii* isolate 1

KT308918 *Betula* *chinensis* isolate 2

KT308917 *Betula* *chinensis* isolate 1

KT308916 *Betula* *chichibuensis* isolate 2

KT308915 *Betula* *chichibuensis* isolate 1

KT308914 *Betula* *calcicola*

KT308913 *Betula* *delavayi* isolate 1

KT308912 *Betula* *bomiensis* isolate 2

KT308911 *Betula* *bomiensis* isolate 1

KT308910 *Betula* *potaninii* isolate 2

KT308909 *Betula* *potaninii* isolate 1

KT308908 *Betula* *corylifolia* isolate 2

KT308907 *Betula* *corylifolia* isolate 1

KT308906 *Betula* *fargesii*

KT308905 *Betula* *globispica* isolate 2

KT308904 *Betula* *globispica* isolate 1

JN247411 *Betula* *pendula* voucher MCA 221

FJ011779 *Betula* *schmidtii* voucher Lee s.n.

FJ011778 *Betula* *platyphylla* voucher Lee s.n.

AY763113 *Betula* *luminifera* isolate 2841

AY761133 *Betula* *schmidtii* isolate 2875

AY761128 *Betula* *platyphylla* isolate 2934

AY761127 *Betula* *pendula* isolate 2902

AY761125 *Betula* *occidentalis* isolate 2883

AY761124 *Betula* *nigra* isolate 2927

AY761122 *Betula* *nana* isolate 3337

AY761121 *Betula* *michauxii* isolate 3406

AY761120 *Betula* *medwediewii* isolate 3465

AY761117 *Betula* *luminifera* isolate 3299

AY761116 *Betula* *luminifera* isolate 2828

AY761115 *Betula* *lenta* isolate 2936

AY761114 *Betula* *humilis* isolate 2894

AY761111 *Betula* *globispica* isolate 2942

AY761110 *Betula* *glandulosa* isolate 3251

AY761107 *Betula* *delavayi* isolate 3462

AY761104 *Betula* *chichibuensis* isolate 2977

AY761103 *Betula* *calcicola* isolate 3460

AY761101 *Betula* *alnoides* isolate 3352

AY761100 *Betula* *alleghaniensis* isolate 2880

AY352336 *Betula* *nana*

AY352334 *Betula* *uber*

AY352332 *Betula* *pendula*

AY352331 *Betula* *nigra*

AY352330 *Betula* *lenta*

DQ397523 *Betula* *occidentalis*

MH231211 *Betula* *atrata*

MH231207 *Betula* *kotulae* (KW006426, holotype)

MH178103 *Betula* *kotulae* (KW008349)

MH178104 *Betula* *kotulae* (KW06427)

MH231207 |*Betula* *kotulae*

MH178105 *Betula* *kotulae* (KW128013)

MH178106 *Betula* *kotulae* (KW128014)

MH178107 *Betula* *kotulae* (KW128016)

MH178108 *Betula* *kotulae* (KW128018)

MH231208 *Betula* *kotulae* (KW128019)

MH231209 *Betula* *kotulae* (KW128020)

MH178109 *Betula* *kotulae* (KW128022)

MH231210 *Betula* *kotulae* (KW128023)

MH238474 *Betula* *kotulae*

MH238475 *Betula* *kotulae* (LW032758)

MH231213 *Betula* *kotulae* (LW006898)

MH238478 *Betula* *kotulae* (LWS27022)

MH238477 *Betula* *kotulae* (LWS27026)

MH231214 *Betula* *kotulae*

Structural variant 3 (C)

YUGGGGGYGGGGGGCUCUCGCCCCUUGCCCCCGA
.((((((((((((((....)))))))))))))).


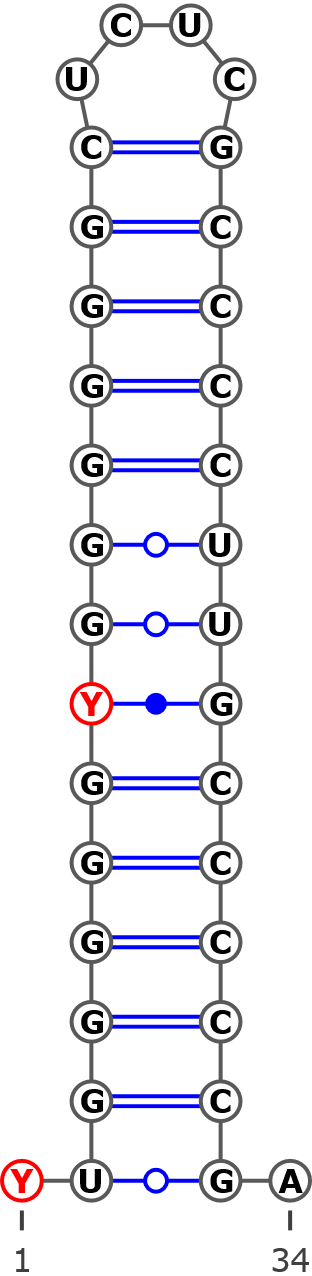


AB243915 *Betula* *apoiensis*, haplotype:ap17

AB243914 *Betula* *apoiensis*, haplotype:ap14

AB243913 *Betula* *apoiensis*, haplotype:ap16

AB243912 *Betula* *apoiensis*, haplotype:ap15

AB243898 *Betula* *ovalifolia*, haplotype:ov3

AB243897 *Betula* *ovalifolia*, haplotype:ov2

AB243895 *Betula* *middendorffii*, haplotype:mi

KT309023 *Betula* *ovalifolia* isolate 2

KT309022 *Betula* *ovalifolia* isolate 1

KT309021 *Betula* *pumila*

KT309019 *Betula* *nana* subsp. *exilis*

KT308989 *Betula* *tianschanica*

KT308966 *Betula* *raddeana*

AY761132 *Betula* *raddeana* isolate 3338

AY761131 *Betula* *pumila* isolate 3246

Structural variant 4 (D)

CUGGGGGUGGGGGGCGAUCUCACCCCUUGCCCCCGA
.(((((((((((((.(....).))))))))))))).


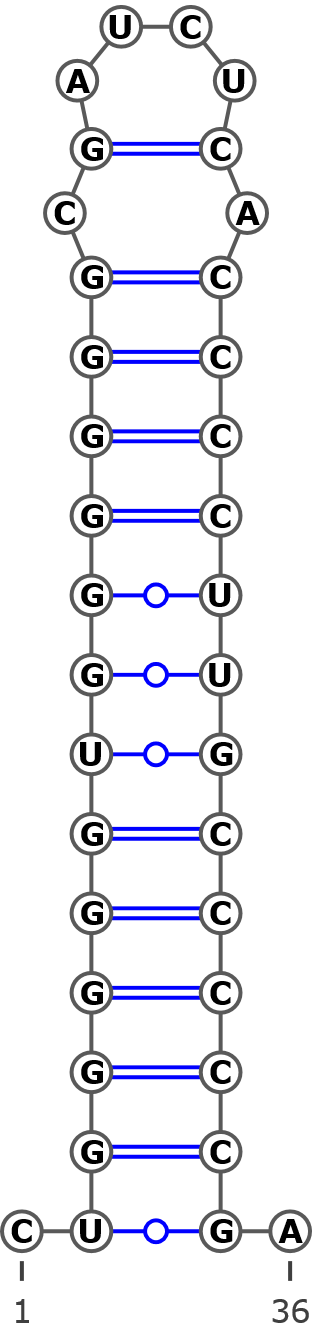


AB243894 *Betula* *davurica*, haplotype:da2

Structural variant 5 (E)

GGGGGGUGUGGGGCGAUCUCGCCCCUUGCCCCCG
.(((((((.((((((....)))))).))))))).


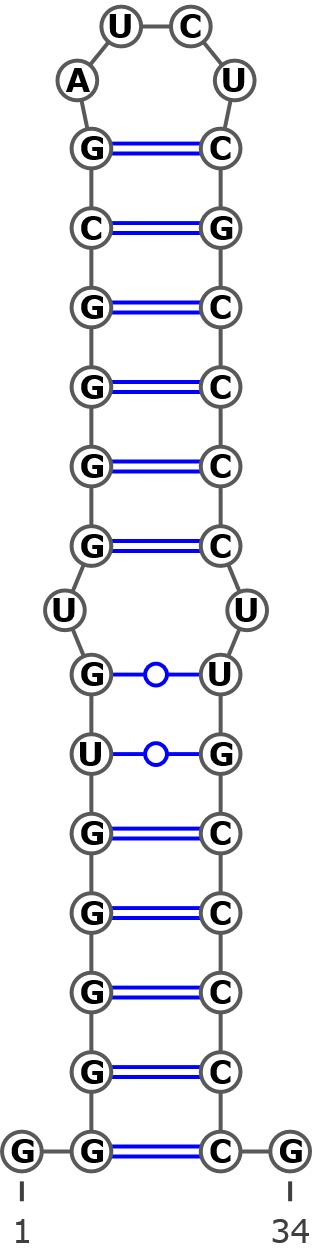


FJ011777 *Betula* *pendula* voucher CS03022

Structural variant 6 (F) optional

CUGGGGGNGGGGGGCGAUCUCGCCCCUUGUCCCCGA
.((((((.((((((((....)))))))).)))))).


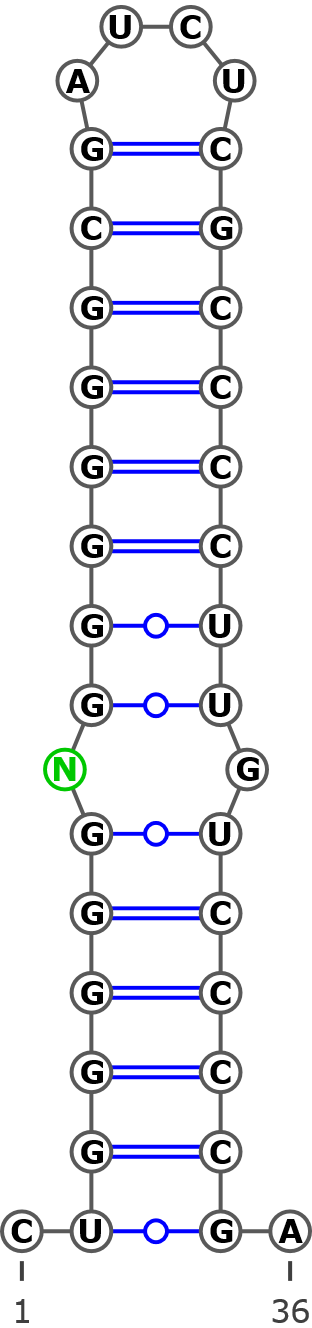


AY352333 *Betula* *populifolia*

Structural variant 7 (G) optional

CUGGGGGCGGGGGGCRAUCUCGCCCCGUGCCCCCGA
.((((((((.(((((......))))).)))))))).


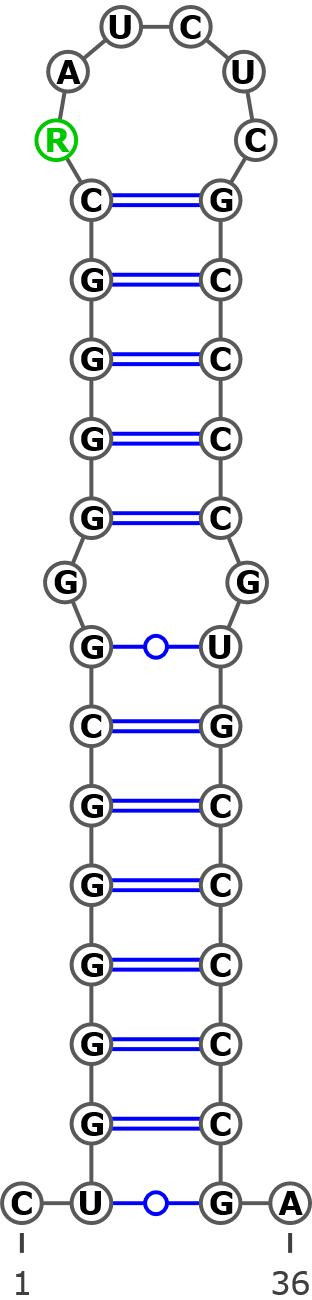


AY763114 *Betula* *alnoides*

**Helix 2**

Structural variant 1 (A)

AYGGYAGGGAGACACUYGUGCAUCCCUGCYGA
.(((((((((..((....))..))))))))).


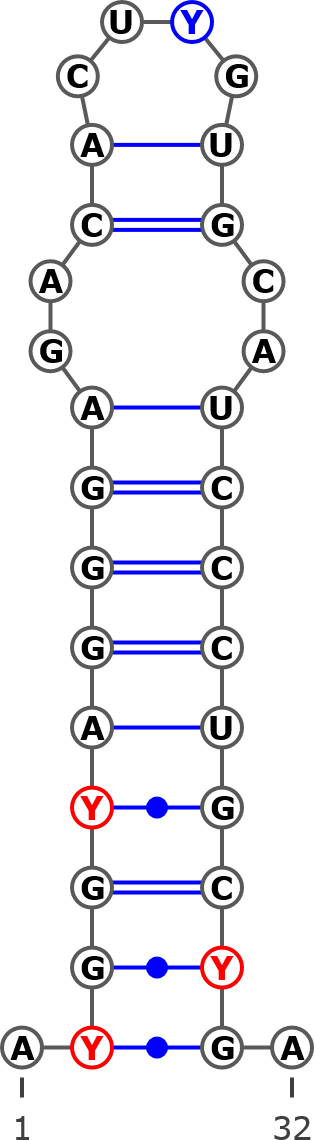


AB243915 *Betula* *apoiensis*, haplotype:ap17

AB243914 *Betula* *apoiensis*, haplotype:ap14

AB243913 *Betula* *apoiensis*, haplotype:ap16

AB243912 *Betula* *apoiensis*, haplotype:ap15

AB243898 *Betula* *ovalifolia*, haplotype:ov3

AB243897 *Betula* *ovalifolia*, haplotype:ov2

AB243895 *Betula* *middendorffii*, haplotype:mi

KT309023 *Betula* *ovalifolia* isolate 2

KT309022 *Betula* *ovalifolia* isolate 1

KT309021 *Betula* *pumila*

KT308989 *Betula* *tianschanica*

KT308966 *Betula* *raddeana*

AY761132 *Betula* *raddeana* isolate 3338

AY761131 *Betula* *pumila* isolate 3246

KT309019 *Betula* *nana* subsp. *exilis*

AB243894 *Betula* *davurica*, haplotype:da2

AB243883 *Betula* *globispica*, haplotype:gl

KT308918 *Betula* *chinensis* isolate 2

KT308917 *Betula* *chinensis* isolate 1

KT308906 *Betula* *fargesii*

KT308905 *Betula* *globispica* isolate 2

KT308904 *Betula* *globispica* isolate 1

AY761120 *Betula* *medwediewii* isolate 3465

AY761111 *Betula* *globispica* isolate 2942

AY761110 *Betula* *glandulosa* isolate 3251

AY761115 *Betula* *lenta* isolate 2936

AY352334 *Betula* *uber*

AY352330 *Betula* *lenta*

AJ006445 *Betula* *pendula*

AJ783646 *Betula* *nigra*

AJ783645 *Betula* *insignis*

AJ251683 *Betula* *alba*

AM503889.2| *Betula* *pendula*

AB243907 *Betula* *apoiensis*, haplotype:ap5

AB243899 *Betula* *apoiensis*, haplotype:ap4

AB243891 *Betula* *platyphylla*, haplotype:pl

AB243889 *Betula* *ermanii*, haplotype:er4

AB243885 *Betula* *corylifolia*, haplotype:co2

AB243884 *Betula* *corylifolia*, haplotype:co1

AB243882 *Betula* *chichibuensis*, haplotype:ch2

AB243881 *Betula* *chichibuensis*, haplotype:ch1

AB243880 *Betula* *schmidtii*, haplotype:sc

KT309028 *Betula* *occidentalis* isolate 2

KT309013 *Betula* *papyrifera* isolate 3

KT309008 *Betula* *pendula* subsp. *mandshurica* isolate 5

KT309007 *Betula* *pendula* subsp. *pendula* isolate 6

KT309006 *Betula* *pendula* subsp. *pendula* isolate 5

KT309005 *Betula* *pendula* subsp. *mandshurica* isolate 4

KT309004 *Betula* *pendula* subsp. *szechuanica* isolate 3

KT309003 *Betula* *pendula* subsp. *szechuanica* isolate 2

KT309002 *Betula* *pendula* subsp. *pendula* isolate 4

KT309001 *Betula* *pendula* subsp. *pendula* isolate 3

KT309000 *Betula* *pendula* subsp. *pendula* isolate 2

KT308999 *Betula* *pendula* subsp. *mandshurica* isolate 3

KT308998 *Betula* *pendula* subsp. *pendula* isolate 1

KT308997 *Betula* *pendula* subsp. *szechuanica* isolate 1

KT308996 *Betula* *pendula* subsp. *mandshurica* isolate 2

KT308993 *Betula* *obscura*

KT308992 *Betula* *turkestanica*

KT308991 *Betula* *pendula* subsp. *mandshurica* isolate 1

KT308990 *Betula* *pendula*

KT308978 *Betula* *michauxii*

KT308968 *Betula* *browicziana*

KT308965 *Betula* *nigra* isolate 2

KT308964 *Betula* *nigra* isolate 1

KT308958 *Betula* *costata*

KT308957 *Betula* *ermanii* isolate 2

KT308956 *Betula* *ermanii* isolate 1

KT308944 *Betula* *luminifera* isolate 3

KT308943 *Betula* *luminifera* isolate 2

KT308942 *Betula* *hainanensis*

KT308941 *Betula* *cylindrostachya*

KT308940 *Betula* *alnoides*

KT308938 *Betula* *lenta* f. *uber* isolate 2

KT308937 *Betula* *lenta* f. *uber* isolate 1

KT308933 *Betula* *megrelica* isolate 2

KT308932 *Betula* *megrelica* isolate 1

KT308931 *Betula* *medwediewii* isolate 2

KT308930 *Betula* *medwediewii* isolate 1

KT308926 *Betula* *murrayana*

KT308925 *Betula* *alleghaniensis*

KT308921 *Betula* *delavayi* isolate 2

KT308920 *Betula* *schmidtii* isolate 2

KT308919 *Betula* *schmidtii* isolate 1

KT308916 *Betula* *chichibuensis* isolate 2

KT308915 *Betula* *chichibuensis* isolate 1

KT308914 *Betula* *calcicola*

KT308913 *Betula* *delavayi* isolate 1

KT308912 *Betula* *bomiensis* isolate 2

KT308911 *Betula* *bomiensis* isolate 1

KT308910 *Betula* *potaninii* isolate 2

KT308909 *Betula* *potaninii* isolate 1

KT308908 *Betula* *corylifolia* isolate 2

KT308907 *Betula* *corylifolia* isolate 1

JN247411 *Betula* *pendula* voucher MCA 221

FJ011779 *Betula* *schmidtii* voucher Lee s.n.

FJ011778 *Betula* *platyphylla* voucher Lee s.n.

FJ011777 *Betula* *pendula* voucher CS03022

AY763113 *Betula* *luminifera* isolate 2841

AY761133 *Betula* *schmidtii* isolate 2875

AY761128 *Betula* *platyphylla* isolate 2934

AY761127 *Betula* *pendula* isolate 2902

AY761125 *Betula* *occidentalis* isolate 2883

AY761124 *Betula* *nigra* isolate 2927

AY761122 *Betula* *nana* isolate 3337

AY761121 *Betula* *michauxii* isolate 3406

AY761117 *Betula* *luminifera* isolate 3299

AY761116 *Betula* *luminifera* isolate 2828

AY761114 *Betula* *humilis* isolate 2894

AY761107 *Betula* *delavayi* isolate 3462

AY761104 *Betula* *chichibuensis* isolate 2977

AY761103 *Betula* *calcicola* isolate 3460

AY761101 *Betula* *alnoides* isolate 3352

AY761100 *Betula* *alleghaniensis* isolate 2880

AY352336 *Betula* *nana*

AY352332 *Betula* *pendula*

AY352331 *Betula* *nigra*

DQ397523 *Betula* *occidentalis*

AY763114 *Betula* *alnoides*

AF432067 *Betula* *papyrifera*

AJ783644 *Betula* *populifolia*

AJ783643 *Betula* *humilis*

AJ783642 *Betula* *pumila*

AJ783641 *Betula* *alnoides* isolate 3464

AB243911 *Betula* *apoiensis*, haplotype:ap12

AB243910 *Betula* *apoiensis*, haplotype:ap10

AB243909 *Betula* *apoiensis*, haplotype:ap11

AB243908 *Betula* *apoiensis*, haplotype:ap13

AB243906 *Betula* *apoiensis*, haplotype:ap9

AB243905 *Betula* *apoiensis*, haplotype:ap8

AB243904 *Betula* *apoiensis*, haplotype:ap7

AB243903 *Betula* *apoiensis*, haplotype:ap6

AB243902 *Betula* *apoiensis*, haplotype:ap3

AB243901 *Betula* *apoiensis*, haplotype:ap2

AB243900 *Betula* *apoiensis*, haplotype:ap1

AB243893 *Betula* *davurica*, haplotype:da1

AB243892 *Betula* *grossa*, haplotype:gr

AB243890 *Betula* *maximovicziana*, haplotype:ma

AB243888 *Betula* *ermanii*, haplotype:er3

AB243887 *Betula* *ermanii*, haplotype:er2

AB243886 *Betula* *ermanii*, haplotype:er1

KT309027 *Betula* *occidentalis* isolate 1

KT309026 *Betula* *humilis* isolate 3

KT309025 *Betula* *humilis* isolate 2

KT309024 *Betula* *humilis* isolate 1

KT309020 *Betula* *nana* isolate 2

KT309018 *Betula* *nana* isolate 1

KT309017 *Betula* *glandulosa* isolate 2

KT309016 *Betula* *cordifolia* isolate 2

KT309015 *Betula* *cordifolia* isolate 1

KT309014 *Betula* *papyrifera* var. *commutata*

KT309012 *Betula* *papyrifera* isolate 2

KT309011 *Betula* *papyrifera* isolate 1

KT309010 *Betula* *populifolia* isolate 3

KT309009 *Betula* *populifolia* isolate 2

KT308995 *Betula* *glandulosa* isolate 1

KT308994 *Betula* *populifolia* isolate 1

KT308988 *Betula × caerulea* isolate 2

KT308987 *Betula × caerulea* isolate 1

KT308986 *Betula* *middendorffii*

KT308985 *Betula* *minor*

KT308984 *Betula* *microphylla*

KT308983 *Betula* *pubescens* var. litiwinowii isolate 2

KT308982 *Betula* *pubescens* var. *pubescens* isolate 4

KT308981 *Betula* *pubescens* var. *pubescens* isolate 3

KT308980 *Betula* *pubescens* var. *pumila* isolate 3

KT308979 *Betula × utahensis*

KT308977 *Betula* *pubescens* subsp. *celtiberica* isolate 2

KT308976 *Betula* *pubescens* var. *pumila* isolate 2

KT308975 *Betula* *pubescens* var. fragans isolate 2

KT308974 *Betula* *pubescens* var. fragans isolate 1

KT308973 *Betula* *pubescens* var. *pumila* isolate 1

KT308972 *Betula* *pubescens* subsp. *celtiberica* isolate 1

KT308971 *Betula* *pubescens* var. litiwinowii isolate 1

KT308970 *Betula* *pubescens* var. *pubescens* isolate 2

KT308969 *Betula* *pubescens* var. *pubescens* isolate 1

KT308967 *Betula* *halophila*

KT308963 *Betula* *dahurica* isolate 2

KT308962 *Betula* *dahurica* isolate 1

KT308961 *Betula* *ashburneri* isolate 3

KT308960 *Betula* *lanata* isolate 2

KT308959 *Betula* *lanata* isolate 1

KT308955 *Betula* *utilis* var. *prattii*

KT308954 *Betula* *albosinensis* isolate 2

KT308953 *Betula* *ashburneri* isolate 2

KT308952 *Betula* *ashburneri* isolate 1

KT308951 *Betula* *utilis* var. jacquemontii

KT308950 *Betula* *utilis* var. *occidentalis* isolate 2

KT308949 *Betula* *utilis* isolate 2

KT308948 *Betula* *utilis* isolate 1

KT308947 *Betula* *albosinensis* var. septentrionalis

KT308946 *Betula* *maximovicziana* isolate 2

KT308945 *Betula* *maximovicziana* isolate 1

KT308936 *Betula* *lenta*

KT308935 *Betula* *grossa* isolate 2

KT308934 *Betula* *grossa* isolate 1

KT308924 *Betula* *albosinensis* isolate 1

KT308923 *Betula* *utilis* var. *occidentalis* isolate 1

FJ011780 *Betula* *utilis* voucher MacAtrher-Tibet Expedition 452

FJ011773 *Betula* *davurica* voucher Tibet218

FJ011772 *Betula* *davurica* voucher UNA63303V

FJ011771 *Betula* *davurica* voucher UNA64631H

FJ011770 *Betula* *davurica* voucher Lee s.n

AY761134 *Betula* *utilis* isolate 2893

AY761130 *Betula* *pubescens* isolate 2895

AY761129 *Betula* *populifolia* isolate 2890

AY761126 *Betula* *papyrifera* isolate 2892

AY761119 *Betula* *maximovicziana* isolate 3463

AY761118 *Betula* *maximovicziana* isolate 2932

AY761113 *Betula* *grossa* isolate 3459

AY761112 *Betula* *grossa* isolate 2948

AY761109 *Betula* *fruticosa* isolate 3339

AY761108 *Betula* *ermanii* isolate 2961

AY761106 *Betula* *corylifolia* isolate 3457

AY761105 *Betula* *chinensis* isolate 2903

AY761102 *Betula* *apoiensis* isolate 3249

AY761099 *Betula* *albosinensis* isolate 3018

AY352337 *Betula* *costata*

AY352335 *Betula* *davurica*

AY352333 *Betula* *populifolia*

MH231211 *Betula* *atrata*

MH014808 *Betula* *borysthenica* (KW0061415, holotype)

MH238480 *Betula* *klokovii* (KW006422, holotype)

MH231207 *Betula* *kotulae* (KW006426, holotype)

MH178103 *Betula* *kotulae* (KW008349)

MH178104 *Betula* *kotulae* (KW06427)

MH178105 *Betula* *kotulae* (KW128013)

MH178106 *Betula* *kotulae* (KW128014)

MH178107 *Betula* *kotulae* (KW128016)

MH178108 *Betula* *kotulae* (KW128018)

MH231208 *Betula* *kotulae* (KW128019)

MH231209 *Betula* *kotulae* (KW128020)

MH178109 *Betula* *kotulae* (KW128022)

MH231210 *Betula* *kotulae* (KW128023)

MH238474 *Betula* *kotulae*

MH238475 *Betula* *kotulae* (LW032758)

MH231213 *Betula* *kotulae* (LW006898)

MH238478 *Betula* *kotulae* (LWS27022)

MH238477 *Betula* *kotulae* (LWS27026)

MH231214 *Betula* *kotulae*

MH238476 *Betula* *oycowiensis* (LWKS031322)

MH014809 *Betula* *pubescens* ssp. *carpatica*

MH231206 *Betula* *pubescens* var. *sibakademica* (LE01041130, type)

MH238471 *Betula* *pubescens* var. *sibakademica* (KW128012)

MH178102 *Betula* *pubescens* var. *sibakademica* (KW128024)

MH238472 *Betula* *pubescens* var. *sibakademica*

MH238473 *Betula* *pubescens* var. *sibakademica*

MH231212 *Betula* *pubescens* var. *sibakademica* (LWKS031322)

Structural variant 2 (B)

CGGCAGGGAGACACUCGUGCAUCCCUGCCU
.((((((((..((....))..)))))))).


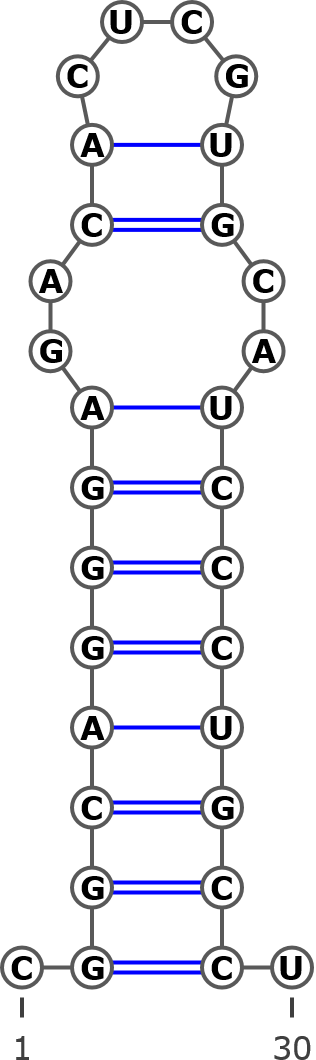


KT308929 *Betula* *insignis* subsp. *fansipanensis*

KT308928 *Betula* *insignis* isolate 2

KT308927 *Betula* *insignis* isolate 1

**Helix 3**

CGGCGCGGUSYGYGCCA
.((((((...)))))).


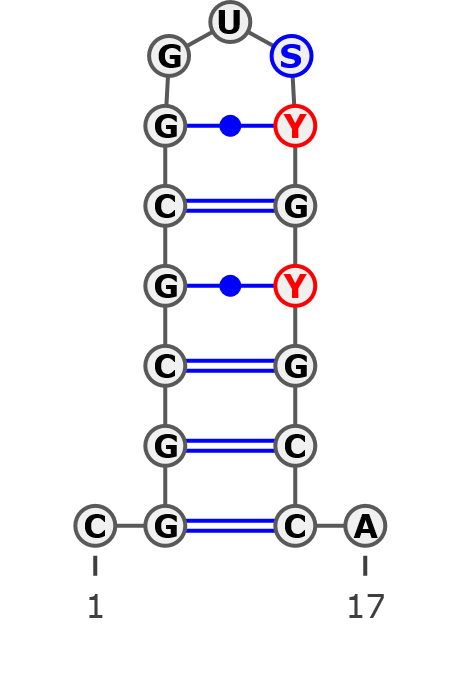


Structurally identical for all analyzed sequences

**Helix 4**

Variant 1 (A)

AGGAAMUUUAACGAAAGAVUGCCUCYNGCCGYCUCGGAAACGGUGUGCGUGCGGGRGGYGAAUCUUGUCUAGAACCA
.((...((((....((((..((((((.(((((.(((....)))...))).)).))))))...))))...)))).)).


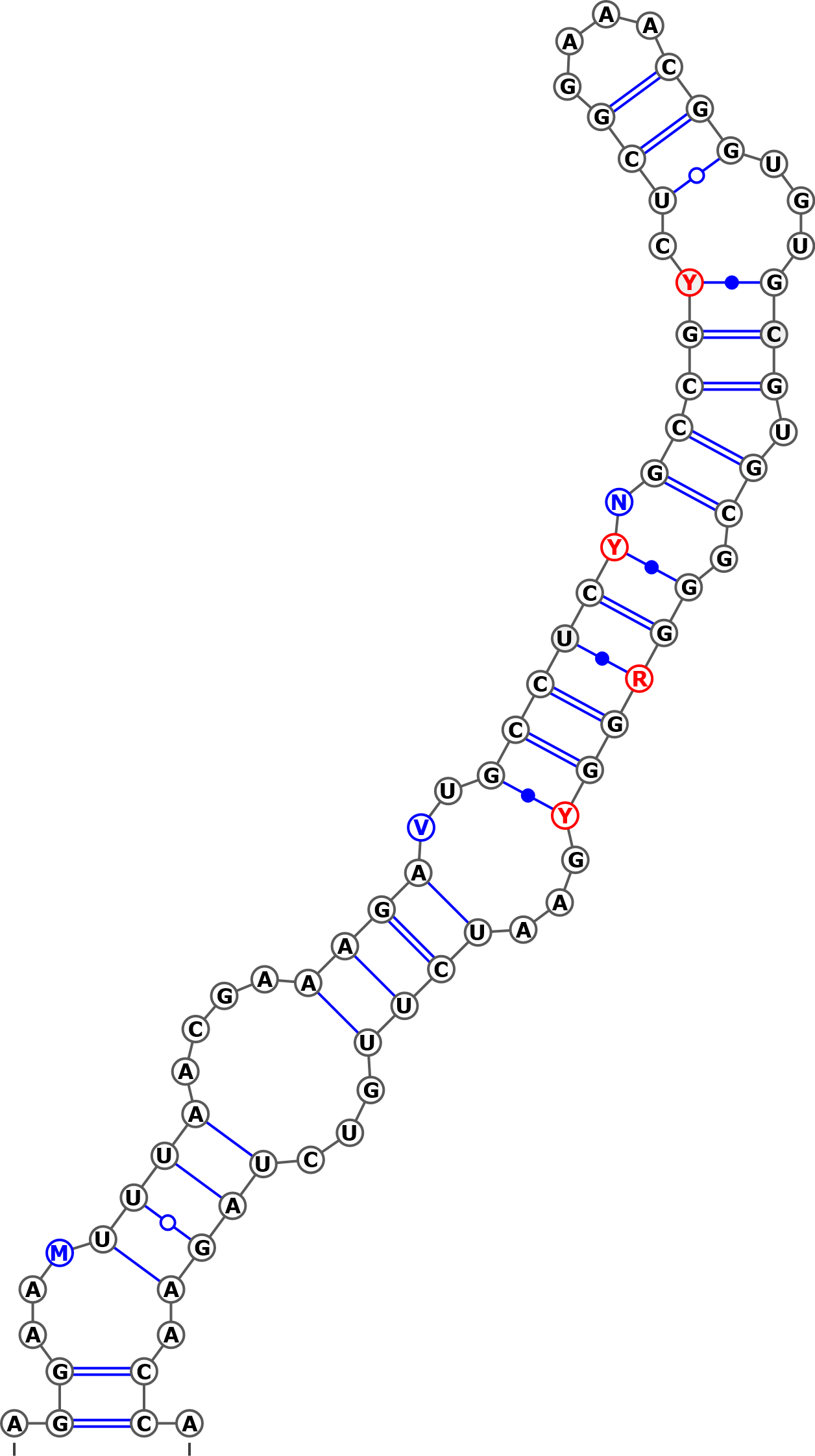


AB243915 *Betula* *apoiensis*, haplotype:ap17

AB243914 *Betula* *apoiensis*, haplotype:ap14

AB243913 *Betula* *apoiensis*, haplotype:ap16

AB243912 *Betula* *apoiensis*, haplotype:ap15

AB243898 *Betula* *ovalifolia*, haplotype:ov3

AB243897 *Betula* *ovalifolia*, haplotype:ov2

AB243895 *Betula* *middendorffii*, haplotype:mi

KT309023 *Betula* *ovalifolia* isolate 2

KT309022 *Betula* *ovalifolia* isolate 1

KT309021 *Betula* *pumila*

KT308989 *Betula* *tianschanica*

KT308966 *Betula* *raddeana*

AY761132 *Betula* *raddeana* isolate 3338

AY761131 *Betula* *pumila* isolate 3246

KT309019 *Betula* *nana* subsp. *exilis*

AB243894 *Betula* *davurica*, haplotype:da2

AB243883 *Betula* *globispica*, haplotype:gl

KT308918 *Betula* *chinensis* isolate 2

KT308917 *Betula* *chinensis* isolate 1

KT308906 *Betula* *fargesii*

KT308905 *Betula* *globispica* isolate 2

KT308904 *Betula* *globispica* isolate 1

AY761120 *Betula* *medwediewii* isolate 3465

AY761111 *Betula* *globispica* isolate 2942

AY761110 *Betula* *glandulosa* isolate 3251

AY761115 *Betula* *lenta* isolate 2936

AY352334 *Betula* *uber*

AY352330 *Betula* *lenta*

AJ006445 *Betula* *pendula*

AJ783645 *Betula* *insignis*

AJ251683 *Betula* *alba*

AM503889.2| *Betula* *pendula*

AB243907 *Betula* *apoiensis*, haplotype:ap5

AB243899 *Betula* *apoiensis*, haplotype:ap4

AB243891 *Betula* *platyphylla*, haplotype:pl

AB243889 *Betula* *ermanii*, haplotype:er4

AB243885 *Betula* *corylifolia*, haplotype:co2

AB243884 *Betula* *corylifolia*, haplotype:co1

AB243882 *Betula* *chichibuensis*, haplotype:ch2

AB243880 *Betula* *schmidtii*, haplotype:sc

KT309028 *Betula* *occidentalis* isolate 2

KT309013 *Betula* *papyrifera* isolate 3

KT309008 *Betula* *pendula* subsp. *mandshurica* isolate 5

KT309007 *Betula* *pendula* subsp. *pendula* isolate 6

KT309006 *Betula* *pendula* subsp. *pendula* isolate 5

KT309005 *Betula* *pendula* subsp. *mandshurica* isolate 4

KT309004 *Betula* *pendula* subsp. *szechuanica* isolate 3

KT309003 *Betula* *pendula* subsp. *szechuanica* isolate 2

KT309002 *Betula* *pendula* subsp. *pendula* isolate 4

KT309001 *Betula* *pendula* subsp. *pendula* isolate 3

KT309000 *Betula* *pendula* subsp. *pendula* isolate 2

KT308999 *Betula* *pendula* subsp. *mandshurica* isolate 3

KT308998 *Betula* *pendula* subsp. *pendula* isolate 1

KT308997 *Betula* *pendula* subsp. *szechuanica* isolate 1

KT308996 *Betula* *pendula* *mandshurica* isolate 2

KT308993 *Betula* *obscura*

KT308992 *Betula* *turkestanica*

KT308991 *Betula* *pendula* *mandshurica* isolate 1

KT308990 *Betula* *pendula*

KT308978 *Betula* *michauxii*

KT308968 *Betula* *browicziana*

KT308958 *Betula* *costata*

KT308957 *Betula* *ermanii* isolate 2

KT308956 *Betula* *ermanii* isolate 1

KT308944 *Betula* *luminifera* isolate 3

KT308943 *Betula* *luminifera* isolate 2

KT308942 *Betula* *hainanensis*

KT308941 *Betula* *cylindrostachya*

KT308940 *Betula* *alnoides*

KT308938 *Betula* *lenta* f. *uber* isolate 2

KT308937 *Betula* *lenta* f. *uber* isolate 1

KT308933 *Betula* *megrelica* isolate 2

KT308932 *Betula* *megrelica* isolate 1

KT308931 *Betula* *medwediewii* isolate 2

KT308930 *Betula* *medwediewii* isolate 1

KT308926 *Betula* *murrayana*

KT308925 *Betula* *alleghaniensis*

KT308920 *Betula* *schmidtii* isolate 2

KT308919 *Betula* *schmidtii* isolate 1

KT308916 *Betula* *chichibuensis* isolate 2

KT308915 *Betula* *chichibuensis* isolate 1

KT308914 *Betula* *calcicola*

KT308913 *Betula* *delavayi* isolate 1

KT308912 *Betula* *bomiensis* isolate 2

KT308911 *Betula* *bomiensis* isolate 1

KT308910 *Betula* *potaninii* isolate 2

KT308909 *Betula* *potaninii* isolate 1

KT308908 *Betula* *corylifolia* isolate 2

KT308907 *Betula* *corylifolia* isolate 1

JN247411 *Betula* *pendula* voucher MCA 221

FJ011779 *Betula* *schmidtii* voucher Lee s.n.

FJ011778 *Betula* *platyphylla* voucher Lee s.n.

FJ011777 *Betula* *pendula* voucher CS03022

AY763113 *Betula* *luminifera* isolate 2841

AY761133 *Betula* *schmidtii* isolate 2875

AY761128 *Betula* *platyphylla* isolate 2934

AY761127 *Betula* *pendula* isolate 2902

AY761125 *Betula* *occidentalis* isolate 2883

AY761122 *Betula* *nana* isolate 3337

AY761121 *Betula* *michauxii* isolate 3406

AY761117 *Betula* *luminifera* isolate 3299

AY761116 *Betula* *luminifera* isolate 2828

AY761114 *Betula* *humilis* isolate 2894

AY761107 *Betula* *delavayi* isolate 3462

AY761104 *Betula* *chichibuensis* isolate 2977

AY761101 *Betula* *alnoides* isolate 3352

AY761100 *Betula* *alleghaniensis* isolate 2880

AY352336 *Betula* *nana*

AY352332 *Betula* *pendula*

DQ397523 *Betula* *occidentalis*

AY763114 *Betula* *alnoides* isolate 3464

AF432067 *Betula* *papyrifera*

AJ783644 *Betula* *populifolia*

AJ783643 *Betula* *humilis*

AJ783642 *Betula* *pumila*

AJ783641 *Betula* *alnoides*

AB243911 *Betula* *apoiensis*, haplotype:ap12

AB243910 *Betula* *apoiensis*, haplotype:ap10

AB243909 *Betula* *apoiensis*, haplotype:ap11

AB243908 *Betula* *apoiensis*, haplotype:ap13

AB243906 *Betula* *apoiensis*, haplotype:ap9

AB243905 *Betula* *apoiensis*, haplotype:ap8

AB243904 *Betula* *apoiensis*, haplotype:ap7

AB243903 *Betula* *apoiensis*, haplotype:ap6

AB243902 *Betula* *apoiensis*, haplotype:ap3

AB243901 *Betula* *apoiensis*, haplotype:ap2

AB243900 *Betula* *apoiensis*, haplotype:ap1

AB243893 *Betula* *davurica*, haplotype:da1

AB243892 *Betula* *grossa*, haplotype:gr

AB243890 *Betula* *maximovicziana*, haplotype:ma

AB243888 *Betula* *ermanii*, haplotype:er3

AB243887 *Betula* *ermanii*, haplotype:er2

AB243886 *Betula* *ermanii*, haplotype:er1

KT309027 *Betula* *occidentalis* isolate 1

KT309026 *Betula* *humilis* isolate 3

KT309025 *Betula* *humilis* isolate 2

KT309024 *Betula* *humilis* isolate 1

KT309020 *Betula* *nana* isolate 2

KT309018 *Betula* *nana* isolate 1

KT309017 *Betula* *glandulosa* isolate 2

KT309016 *Betula* *cordifolia* isolate 2

KT309015 *Betula* *cordifolia* isolate 1

KT309014 *Betula* *papyrifera* var. *commutata*

KT309012 *Betula* *papyrifera* isolate 2

KT309011 *Betula* *papyrifera* isolate 1

KT309010 *Betula* *populifolia* isolate 3

KT309009 *Betula* *populifolia* isolate 2

KT308995 *Betula* *glandulosa* isolate 1

KT308994 *Betula* *populifolia* isolate 1

KT308988 *Betula × caerulea* isolate 2

KT308987 *Betula × caerulea* isolate 1

KT308986 *Betula* *middendorffii*

KT308985 *Betula* *minor*

KT308984 *Betula* *microphylla*

KT308983 *Betula* *pubescens* var. *litwinowii* isolate 2

KT308982 *Betula* *pubescens* var. *pubescens* isolate 4

KT308981 *Betula* *pubescens* var. *pubescens* isolate 3

KT308980 *Betula* *pubescens* var. *pumila* isolate 3

KT308979 *Betula × utahensis*

KT308977 *Betula* *pubescens* subsp. *celtiberica* isolate 2

KT308976 *Betula* *pubescens* var. *pumila* isolate 2

KT308975 *Betula* *pubescens* var. *fragrans* isolate 2

KT308974 *Betula* *pubescens* var. *fragrans* isolate 1

KT308973 *Betula* *pubescens* var. *pumila* isolate 1

KT308972 *Betula* *pubescens* subsp. *celtiberica* isolate 1

KT308971 *Betula* *pubescens* var. *litwinowii* isolate 1

KT308970 *Betula* *pubescens* var. *pubescens* isolate 2

KT308969 *Betula* *pubescens* var. *pubescens* isolate 1

KT308967 *Betula* *halophila*

KT308963 *Betula* *dahurica* isolate 2

KT308962 *Betula* *dahurica* isolate 1

KT308961 *Betula* *ashburneri* isolate 3

KT308960 *Betula* *lanata* isolate 2

KT308959 *Betula* *lanata* isolate 1

KT308955 *Betula* *utilis* var. *prattii*

KT308954 *Betula* *albosinensis* isolate 2

KT308953 *Betula* *ashburneri* isolate 2

KT308952 *Betula* *ashburneri* isolate 1

KT308951 *Betula* *utilis* var. jacquemontii

KT308950 *Betula* *utilis* var. *occidentalis* isolate 2

KT308949 *Betula* *utilis* isolate 2

KT308948 *Betula* *utilis* isolate 1

KT308947 *Betula* *albosinensis* var. septentrionalis

KT308946 *Betula* *maximovicziana* isolate 2

KT308945 *Betula* *maximovicziana* isolate 1

KT308936 *Betula* *lenta*

KT308935 *Betula* *grossa* isolate 2

KT308934 *Betula* *grossa* isolate 1

KT308924 *Betula* *albosinensis* isolate 1

KT308923 *Betula* *utilis* var. *occidentalis* isolate 1

FJ011780 *Betula* *utilis* voucher MacAtrher-Tibet Expedition 452

FJ011773 *Betula* *davurica* voucher Tibet218

FJ011772 *Betula* *davurica* voucher UNA63303V

FJ011771 *Betula* *davurica* voucher UNA64631H

FJ011770 *Betula* *davurica* voucher Lee s.n.

AY761134 *Betula* *utilis* isolate 2893

AY761130 *Betula* *pubescens* isolate 2895

AY761129 *Betula* *populifolia* isolate 2890

AY761126 *Betula* *papyrifera* isolate 2892

AY761119 *Betula* *maximovicziana* isolate 3459

AY761118 *Betula* *maximovicziana* isolate 2932

AY761113 *Betula* *grossa* isolate 3459

AY761112 *Betula* *grossa* isolate 2948

AY761109 *Betula* *fruticosa* isolate 3339

AY761108 *Betula* *ermanii* isolate 2961

AY761106 *Betula* *corylifolia* isolate 3457

AY761105 *Betula* *chinensis* isolate 2903

AY761102 *Betula* *apoiensis* isolate 3249

AY761099 *Betula* *albosinensis* isolate 3018

AY352337 *Betula* *costata*

AY352335 *Betula* *davurica*

AY352333 *Betula* *populifolia*

KT308929 *Betula* *insignis* subsp. *fansipanensis*

KT308928 *Betula* *insignis* isolate 2

AY761103 *Betula* *calcicola* isolate 3460

MH231211 *Betula* *atrata*

MH014808 *Betula* *borysthenica* (KW0061415, holotype)

MH238480 *Betula* *klokovii* (KW006422, holotype)

MH231207 *Betula* *kotulae* (KW006426, holotype)

MH178103 *Betula* *kotulae* (KW008349)

MH178104 *Betula* *kotulae* (KW06427)

MH178105 *Betula* *kotulae* (KW128013)

MH178106 *Betula* *kotulae* (KW128014)

MH178107 *Betula* *kotulae* (KW128016)

MH178108 *Betula* *kotulae* (KW128018)

MH231208 *Betula* *kotulae* (KW128019)

MH231209 *Betula* *kotulae* (KW128020)

MH178109 *Betula* *kotulae* (KW128022)

MH231210 *Betula* *kotulae* (KW128023)

MH238474 *Betula* *kotulae*

MH238475 *Betula* *kotulae* (LW032758)

MH231213 *Betula* *kotulae* (LW006898)

MH238478 *Betula* *kotulae* (LWS27022)

MH238477 *Betula* *kotulae* (LWS27026)

MH231214 *Betula* *kotulae*

MH238476 *Betula* *oycowiensis* (LWKS031322)

MH014809 *Betula* *pubescens* ssp. *Carpatica*

MH231206 *Betula* *pubescens* var. *sibakademica* (LE01041130, type)

MH238471 *Betula* *pubescens* var. *sibakademica* (KW128012)

MH178102 *Betula* *pubescens* var. *sibakademica* (KW128024)

MH238472 *Betula* *pubescens* var. *sibakademica*

MH238473 *Betula* *pubescens* var. *sibakademica*

MH231212 *Betula* *pubescens* var. *sibakademica* (LWKS031322)

Structural variant 2 (B)

AGGAACUUUAACGAAAGAGUGCCUCCGGCCGCCUCGGAAACGGUGUGCGUGCAGGAAAACAAUCUUGUCUAGAACCA
.((...((((....((((.((..(((.(((((.(((....)))...))).)).)))...)).))))...)))).)).


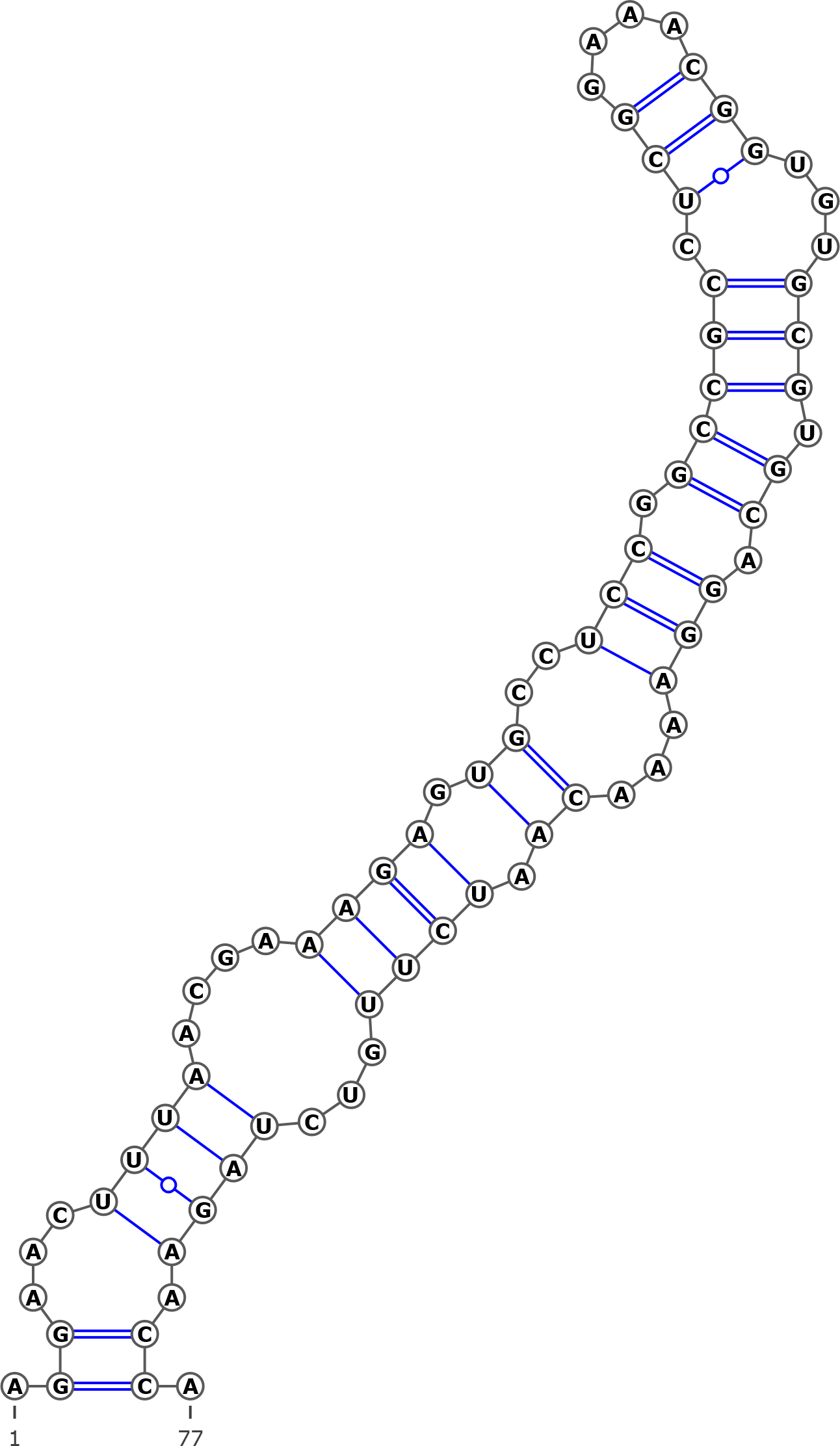


AJ783646 *Betula* *nigra*

KT308965 *Betula* *nigra*

KT308964 *Betula* *nigra* isolate 1

AY761124 *Betula* *nigra* isolate 2927

AY352331 *Betula* *nigra*

AJ783646 *Betula* *nigra*

Structural variant 3 (C)

AGGAAAUUUAACGAAAGAGUGCCUCYGGCCGCCUCGGAAACGGUGUGCGUGCGGGAGGUGAAUCUUGUCUAGAACCA
.((...((((....((((..(((((..(((((.(((....)))...))).))..)))))...))))...)))).)).


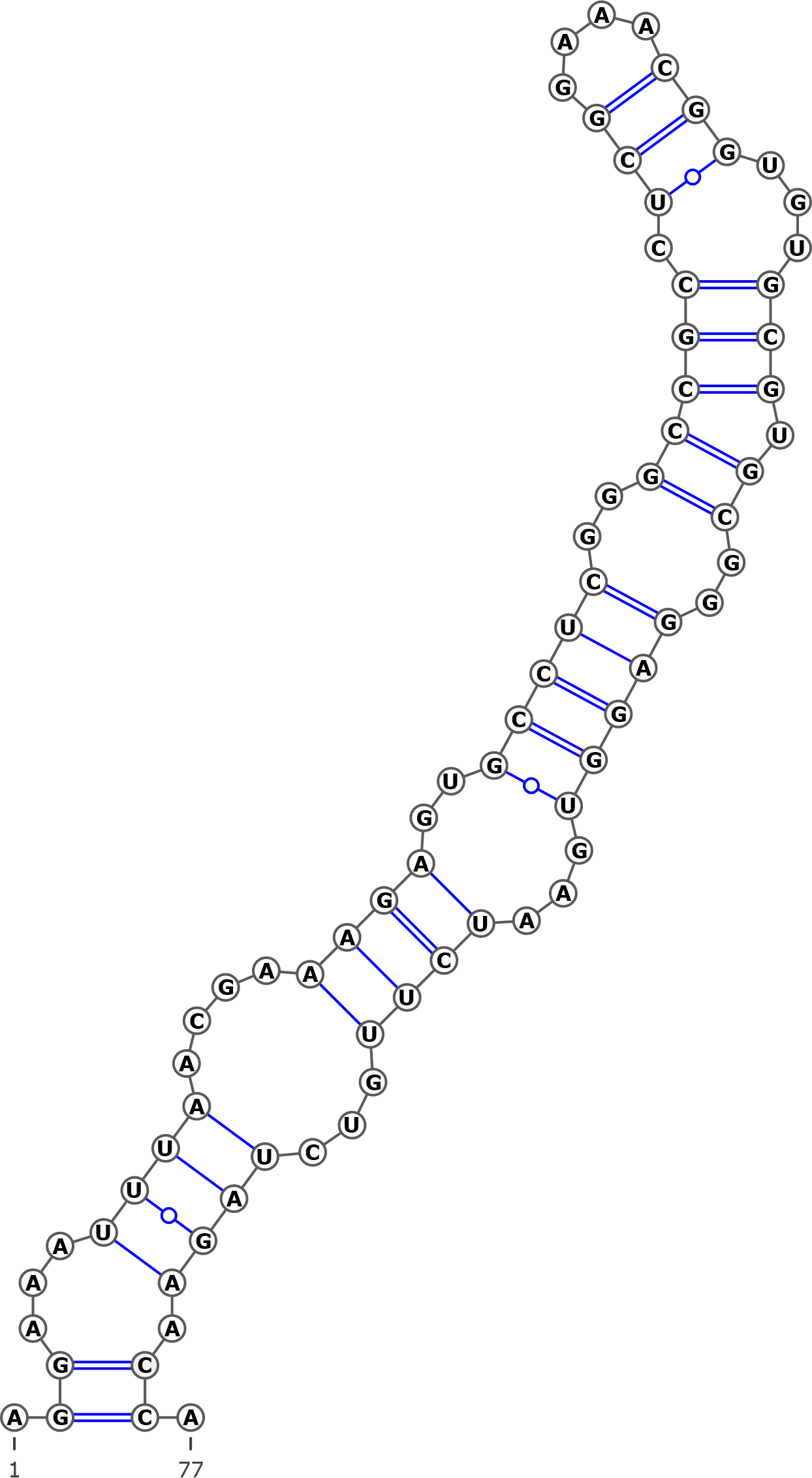


KT308921 *Betula* *delavayi* isolate 2

AY761103 *Betula* *calcicola* isolate 3460

Structural variant 4 (D)

AGGAACUUUAACGAAAGAGUGCCUCCGGCCGCCUCGGAAACGGUGUGCGUGCGGUAGGUGAAUCUUGUCUAGAACCA
.((...((((....((((..((((.(.(((((.(((....)))...))).)).).))))...))))...)))).)).


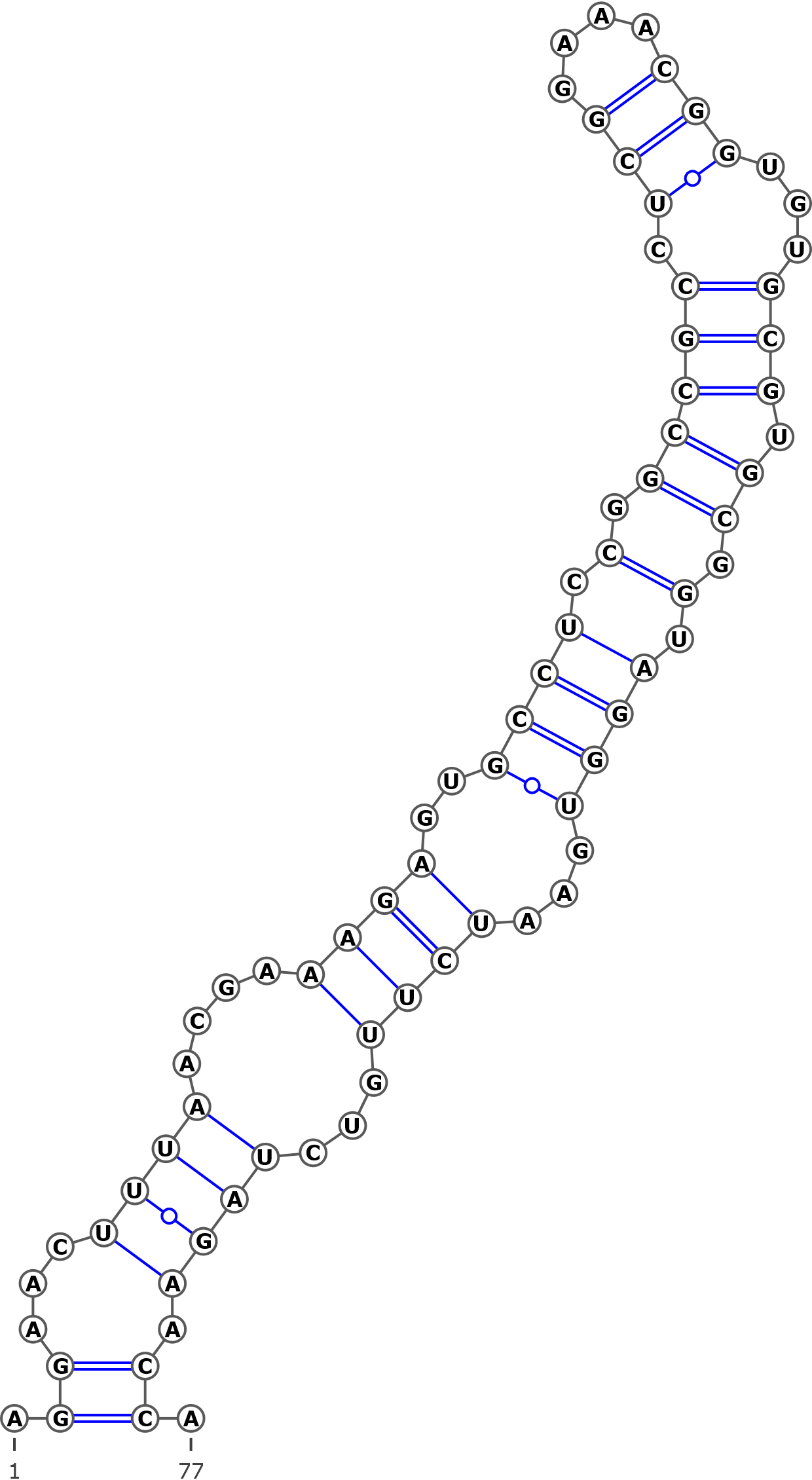


AB243881 *Betula* *chichibuensis*, haplotype:ch1
