# Supplementary material for: ITS secondary structure reconstruction to resolve taxonomy and phylogeny of the Betula L. genus
Source: PeerJ. 2021 Mar 23;9:e10889. doi: 10.7717/peerj.10889 (PMC7996101; doi:10.7717/peerj.10889)
Supplement: Supplemental Information 6 [file peerj-09-10889-s006.docx]

**Data S6.** Hemicompensatory base changes (hCBCs) in ITS1.

For every hCBC only one variant (state) provided as a list. The second one refers to all the other sequences used in this study.

**Helix 1**

Position 15 C/U, var. “U“:

AB243902 *Betula* *apoiensis* haplotype:ap3

Position 20 C/U, var. “U”:

AY761103 *Betula* *calcicola* isolate 3460

AY761104 *Betula* *chichibuensis* isolate 2977

AY761107 *Betula* *delavayi* isolate 3462

AY761133 *Betula* *schmidtii* isolate 2875

FJ011779 *Betula* *schmidtii* voucher Lee s.n.

KT308909 *Betula* *potaninii* isolate 1

KT308910 *Betula* *potaninii* isolate 2

KT308913 *Betula* *delavayi* isolate 1

KT308914 *Betula* *calcicola*

KT308915 *Betula* *chichibuensis* isolate 1

KT308916 *Betula* *chichibuensis* isolate 2

KT308919 *Betula* *schmidtii* isolate 1

KT308920 *Betula* *schmidtii* isolate 2

KT308921 *Betula* *delavayi* isolate 2

Position 32 C/U, var. “U”

AY761131 *Betula* *pumila* isolate 3246

AY761133 *Betula* *schmidtii* isolate 2875

FJ011779 *Betula* *schmidtii* voucher Lee s.n.

KT308919 *Betula* *schmidtii* isolate 1

KT308920 *Betula* *schmidtii* isolate 2

**Helix 2**

Position 75 C/U, var. „U“:

AJ783646 *Betula* *nigra*

AY352331 *Betula* *nigra*

AY761124 *Betula* *nigra* isolate 2927

AY763113 *Betula* *luminifera* isolate 2841

KT308911 *Betula* *bomiensis* isolate 1

KT308912 *Betula* *bomiensis* isolate 2

KT308964 *Betula* *nigra* isolate 1

KT308965 *Betula* *nigra* isolate 2

Position 76C/U var. „U“:

AB243886 *Betula* *ermanii* haplotype:er1

AB243887 *Betula* *ermanii* haplotype:er2

AB243888 *Betula* *ermanii* haplotype:er3

AB243889 *Betula* *ermanii* haplotype:er4

AB243899 *Betula* *apoiensis* haplotype:ap4

AB243900 *Betula* *apoiensis* haplotype:ap1

AB243901 *Betula* *apoiensis* haplotype:ap2

AB243902 *Betula* *apoiensis* haplotype:ap3

AB243903 *Betula* *apoiensis* haplotype:ap6

AB243904 *Betula* *apoiensis* haplotype:ap7

AB243905 *Betula* *apoiensis* haplotype:ap8

AB243906 *Betula* *apoiensis* haplotype:ap9

AB243907 *Betula* *apoiensis* haplotype:ap5

AB243910 *Betula* *apoiensis* haplotype:ap10

AB243911 *Betula* *apoiensis* haplotype:ap12

AY761099 *Betula* *albosinensis* isolate 3018

AY761102 *Betula* *apoiensis* isolate 3249

AY761108 *Betula* *ermanii* isolate 2961

AY761134 *Betula* *utilis* isolate 2893

FJ011773 *Betula* *davurica* voucher Tibet218

FJ011780 *Betula* *utilis* voucher MacAtrher-Tibet Expedition 452

KT308948 *Betula* *utilis* isolate 1

KT308949 *Betula* *utilis* isolate 2

KT308951 *Betula* *utilis* var. *jacquemontii*

KT308952 *Betula* *ashburneri* isolate 1

KT308953 *Betula* *ashburneri* isolate 2

KT308954 *Betula* *albosinensis* isolate 2

KT308955 *Betula* *utilis* var. prattii

KT308956 *Betula* *ermanii* isolate 1

KT308957 *Betula* *ermanii* isolate 2

KT308959 *Betula* *lanata* isolate 1

KT308960 *Betula* *lanata* isolate 2

KT308961 *Betula* *ashburneri* isolate 3

Position 73 C/U, var. “U”:

AY352333 *Betula* *populifolia*

AY761129 *Betula* *populifolia* isolate 2890

**Helix 3**

Position 81 C/U, var. “U”:

AY761103 *Betula* *calcicola* isolate 3460

AY761107 *Betula* *delavayi* isolate 3462

KT308909 *Betula* *potaninii* isolate 1

KT308910 *Betula* *potaninii* isolate 2

KT308913 *Betula* *delavayi* isolate 1

KT308914 *Betula* *calcicola*

KT308921 *Betula* *delavayi* isolate 2

Position 109 C/U, var. “U”:

AB243881 *Betula* *chichibuensis* haplotype:ch1

AB243882 *Betula* *chichibuensis* haplotype:ch2

AB243889 *Betula* *ermanii* haplotype:er4

AB243899 *Betula* *apoiensis* haplotype:ap4

AJ783646 *Betula* *nigra*

AY352331 *Betula* *nigra*

AY761103 *Betula* *calcicola* isolate 3460

AY761104 *Betula* *chichibuensis* isolate 2977

AY761107 *Betula* *delavayi* isolate 3462

AY761124 *Betula* *nigra* isolate 2927

KT308909 *Betula* *potaninii* isolate 1

KT308910 *Betula* *potaninii* isolate 2

KT308911 *Betula* *bomiensis* isolate 1

KT308912 *Betula* *bomiensis* isolate 2

KT308913 *Betula* *delavayi* isolate 1

KT308914 *Betula* *calcicola*

KT308915 *Betula* *chichibuensis* isolate 1

KT308916 *Betula* *chichibuensis* isolate 2

KT308921 *Betula* *delavayi* isolate 2

KT308964 *Betula* *nigra* isolate 1

KT308965 *Betula* *nigra* isolate 2

Position 84U/C var. “C”:

AY352330 *Betula* *lenta*

AY352334 *Betula* *uber*

AY352337 *Betula* *costata*

AY761100 *Betula* *alleghaniensis* isolate 2880

AY761103 *Betula* *calcicola* isolate 3460

AY761107 *Betula* *delavayi* isolate 3462

AY761115 *Betula* *lenta* isolate 2936

AY761120 *Betula* *medwediewii* isolate 3465

KT308909 *Betula* *potaninii* isolate 1

KT308910 *Betula* *potaninii* isolate 2

KT308913 *Betula* *delavayi* isolate 1

KT308914 *Betula* *calcicola*

KT308921 *Betula* *delavayi*

KT308925 *Betula* *alleghaniensis*

KT308926 *Betula* *murrayana*

KT308927 *Betula* *insignis* isolate 1

KT308928 *Betula* *insignis* isolate 2

KT308929 *Betula* *insignis* subsp. fansipanensis

KT308930 *Betula* *medwediewii* isolate 1

KT308931 *Betula* *medwediewii* isolate 2

KT308932 *Betula* *megrelica* isolate 1

KT308933 *Betula* *megrelica* isolate 2

KT308936 *Betula* *lenta*

KT308937 *Betula* *lenta* f. *uber* isolate 1

KT308938 *Betula* *lenta* f. *uber* isolate 2

Position 135 C/U, var. “U”:

AY761103 *Betula* *calcicola* isolate 3460

AY761107 *Betula* *delavayi* isolate 3462

KT308909 *Betula* *potaninii* isolate 1

KT308910 *Betula* *potaninii* isolate 2

KT308913 *Betula* *delavayi* isolate 1

KT308914 *Betula* *calcicola*

KT308921 *Betula* *delavayi* isolate 2

Position 133 U/C, var. “C”

AB243891 *Betula* *platyphylla* haplotype:pl

AJ006445 *Betula* *pendula*

AJ251683 *Betula* *alba*

AJ783644 *Betula* *populifolia*

AM503889.2 *Betula* *pendula*

AY352332 *Betula* *pendula*

AY352333 *Betula* *populifolia*

AY761114 *Betula* *humilis* isolate 2894

AY761127 *Betula* *pendula* isolate 2902

AY761128 *Betula* *platyphylla* isolate 2934

AY761129 *Betula* *populifolia* isolate 2890

AY761130 *Betula* *pubescens* isolate 2895

FJ011777 *Betula* *pendula* voucher CS03022

FJ011778 *Betula* *platyphylla* voucher Lee s.n.

JN247411 *Betula* *pendula* voucher MCA 221

KT308923 *Betula* *utilis* var. *occidentalis* isolate 1

KT308968 *Betula* *browicziana*

KT308969 *Betula* *pubescens* var. *pubescens* isolate 1

KT308970 *Betula* *pubescens* var. *pubescens* isolate 2

KT308971 *Betula* *pubescens* var. *litwinowii* isolate 1

KT308972 *Betula* *pubescens* subsp. *celtiberica* isolate 1

KT308973 *Betula* *pubescens* var. *pumila* isolate 1

KT308974 *Betula* *pubescens* var. *fragans* isolate 1

KT308975 *Betula* *pubescens* var. *fragans* isolate 2

KT308976 *Betula* *pubescens* var. *pumila* isolate 2

KT308977 *Betula* *pubescens* subsp. *celtiberica* isolate 2

KT308980 *Betula* *pubescens* var. *pumila* isolate 3

KT308981 *Betula* *pubescens* var. *pubescens* isolate 3

KT308982 *Betula* *pubescens* var. *pubescens* isolate 4

KT308983 *Betula* *pubescens* var. *litwinowii* isolate 2

KT308986 *Betula* *middendorffii*

KT308990 *Betula* *pendula*

KT308992 *Betula* *turkestanica*

KT308993 *Betula* *obscura*

KT308994 *Betula* *populifolia* isolate 1

KT308996 *Betula* *pendula* subsp. *mandshurica* isolate 2

KT308997 *Betula* *pendula* subsp. *szechuanica* isolate 1

KT308998 *Betula* *pendula* subsp. *pendula* isolate 1

KT308999 *Betula* *pendula* subsp. *mandshurica* isolate 3

KT309000 *Betula* *pendula* subsp. *pendula* isolate 2

KT309001 *Betula* *pendula* subsp. *pendula* isolate 3

KT309002 *Betula* *pendula* subsp. *pendula* isolate 4

KT309003 *Betula* *pendula* subsp. *szechuanica* isolate 2

KT309004 *Betula* *pendula* subsp. *szechuanica* isolate 3

KT309005 *Betula* *pendula* subsp. *mandshurica* isolate 4

KT309006 *Betula* *pendula* subsp. *pendula* isolate 5

KT309007 *Betula* *pendula* subsp. *pendula* isolate 6

KT309008 *Betula* *pendula* subsp. *mandshurica* isolate 5

KT309009 *Betula* *populifolia* isolate 2

KT309013 *Betula* *papyrifera*

MH014808 *Betula* *borysthenica*

MH014809 *Betula* *pubescens* ssp. *carpatica*

MH178101 *Betula* *pubescens* var. *sibakademica*

MH238476 *Betula* *oycowiensis*

MH238480 *Betula* *klokovii*

**Helix 4**

Position 165C/U, var. “U”

AB243910 *Betula* *apoiensis* haplotype:ap10

AY761107 *Betula* *delavayi* isolate 3462

KT308909 *Betula* *potaninii* isolate 1

KT308910 *Betula* *potaninii* isolate 2

KT308914 *Betula* *calcicola*

Position 171 C/U, var. “U”

AY352330 *Betula* *lenta*

AY352334 *Betula* *uber*

AY761115 *Betula* *lenta* isolate 2936

AY761116 *Betula* *luminifera* isolate 2828

AY761117 *Betula* *luminifera* isolate 3299

AY763113 *Betula* *luminifera* isolate 2841

KT308936 *Betula* *lenta*

KT308937 *Betula* *lenta* f. *uber* isolate 1

KT308938 *Betula* *lenta* f. *uber* isolate 2

Position 195 A/G, var. „G”:

AB243904 *Betula* *apoiensis* haplotype:ap7

Position 198 U/C, var. “C”:

AB243892 *Betula* *grossa* haplotype:gr

AY352330 *Betula* *lenta*

AY352334 *Betula* *uber*

AY352337 *Betula* *costata*

AY761100 *Betula* *alleghaniensis*

AY761112 *Betula* *grossa* isolate 2948

AY761113 *Betula* *grossa* isolate 3459

AY761115 *Betula* *lenta* isolate 2936

AY761120 *Betula* *medwediewii* isolate 3465

KT308925 *Betula* *alleghaniensis*

KT308926 *Betula* *murrayana*

KT308927 *Betula* *insignis* isolate 1

KT308928 *Betula* *insignis* isolate 2

KT308929 *Betula* *insignis* subsp. fansipanensis

KT308930 *Betula* *medwediewii* isolate 1

KT308931 *Betula* *medwediewii* isolate 2

KT308932 *Betula* *megrelica* isolate 1

KT308933 *Betula* *megrelica* isolate 2

KT308934 *Betula* *grossa* isolate 1

KT308935 *Betula* *grossa* isolate 2

KT308936 *Betula* *lenta*

KT308937 *Betula* *lenta* f. *uber* isolate 1

KT308938 *Betula* *lenta* f. *uber* isolate 2
