# Supplementary material for: ITS secondary structure reconstruction to resolve taxonomy and phylogeny of the Betula L. genus
Source: PeerJ. 2021 Mar 23;9:e10889. doi: 10.7717/peerj.10889 (PMC7996101; doi:10.7717/peerj.10889)
Supplement: Supplemental Information 7 [file peerj-09-10889-s007.docx]

**Data S7.** Hemicompensatory base changes (hCBCs) in ITS2.

For every hCBC only one variant (state) provided as a list. The second one refers to all the other sequences used in this study.

**Helix 1**

Position 73 C/U, var. “U”:

AJ783646 *Betula* *nigra*

AY352331 *Betula* *nigra*

AY761124 *Betula* *nigra* isolate 2927

KT308964 *Betula* *nigra* isolate 1

KT308965 *Betula* *nigra* isolate 2

**Helix 2**

Position 111 C/U, var. “U”:

AB243896 *Betula* *ovalifolia* haplotype:ov1

AB243909 *Betula* *apoiensis* haplotype:ap11

AB243910 *Betula* *apoiensis* haplotype:ap10

AB243911 *Betula* *apoiensis* haplotype:ap12

AB243913 *Betula* *apoiensis* haplotype:ap16

AB243914 *Betula* *apoiensis* haplotype:ap14

AB243915 *Betula* *apoiensis* haplotype:ap17

KT309022 *Betula* *ovalifolia* isolate 1

KT309023 *Betula* *ovalifolia* isolate 2

**Helix 3**

Position 118 G/A, var. “A”:

AB243901 *Betula* *apoiensis* haplotype:ap2

Position 119 C/U, var. “U”:

AB243881 *Betula* *chichibuensis* haplotype:ch1

AB243882 *Betula* *chichibuensis* haplotype:ch2

AY761104 *Betula* *chichibuensis* isolate 2977

KT308915 *Betula* *chichibuensis* isolate 1

KT308916 *Betula* *chichibuensis* isolate 2

Position 137 C/U, var. “U”:

AY761103 *Betula* *calcicola* isolate 3460

AY761107 *Betula* *delavayi* isolate 3462

KT308909 *Betula* *potaninii* isolate 1

KT308910 *Betula* *potaninii* isolate 2

KT308913 *Betula* *delavayi* isolate 1

KT308914 *Betula* *calcicola*

KT308921 *Betula* *delavayi* isolate 2

Position 191 C/U, var. “U”:

AJ783646 *Betula* *nigra*

AY352331 *Betula* *nigra*

AY761101 *Betula* *alnoides* isolate 3352

AY761116 *Betula* *luminifera* isolate 2828

AY761117 *Betula* *luminifera* isolate 3299

AY761124 *Betula* *nigra* isolate 2927

AY763113 *Betula* *luminifera* isolate 2841

AY763114 *Betula* *alnoides* isolate 3464

FJ011769 *Betula* *alnoides* voucher Wen6405

KT308911 *Betula* *bomiensis* isolate 1

KT308912 *Betula* *bomiensis* isolate 2

KT308922 *Betula* *delavayi* isolate 3

KT308939 *Betula* *luminifera* isolate 1

KT308940 *Betula* *alnoides*

KT308941 *Betula* cylindrostachya

KT308942 *Betula* hainanensis

KT308943 *Betula* *luminifera* isolate 2

KT308944 *Betula* *luminifera* isolate 3

KT308964 *Betula* *nigra* isolate 1

KT308965 *Betula* *nigra* isolate 2

Position 188 C/U, var. “U”:

AB243880 *Betula* *schmidtii* haplotype:sc

AB243886 *Betula* *ermanii* haplotype:er1

AB243888 *Betula* *ermanii* haplotype:er3

AB243899 *Betula* *apoiensis* haplotype:ap4

AB243900 *Betula* *apoiensis* haplotype:ap1

AB243901 *Betula* *apoiensis* haplotype:ap2

AB243902 *Betula* *apoiensis* haplotype:ap3

AB243912 *Betula* *apoiensis* haplotype:ap15

AY761133 *Betula* *schmidtii* isolate 2875

FJ011779 *Betula* *schmidtii* voucher Lee s.n.

KT308919 *Betula* *schmidtii* isolate 1

KT308920 *Betula* *schmidtii* isolate 2

KT308940 *Betula* *alnoides*

KT308959 *Betula* *lanata* isolate 1

KT308960 *Betula* *lanata* isolate 2

Position 194 U/C, var. “C”:

KT308926 *Betula* *murrayana*

Position 193 C/U, var. “U”:

FJ011770 *Betula* *davurica* voucher Lee s.n.

Position 198 C/U, var. “U”

KT308950 *Betula* *utilis* var. *occidentalis* isolate 2

**Helix 4**

Position 220 U/C, var. “C”:

AB243883 *Betula* *globispica* haplotype:gl

AJ783644 *Betula* *populifolia*

AJ783645 *Betula* *insignis*

AY761110 *Betula* *glandulosa* isolate 3251

AY761111 *Betula* *globispica* isolate 2942

AY761130 *Betula* *pubescens* isolate 2895

KT308904 *Betula* *globispica* isolate 1

KT308905 *Betula* *globispica* isolate 2

KT308906 *Betula* *fargesii*

KT308911 *Betula* *bomiensis* isolate 1

KT308912 *Betula* *bomiensis* isolate 2

KT308917 *Betula* *chinensis* isolate 1

KT308918 *Betula* *chinensis* isolate 2

KT308971 *Betula* *pubescens* var. *litwinowii* isolate 1

KT308973 *Betula* *pubescens* var. *pumila* isolate 1

KT308974 *Betula* *pubescens* var. *fragans* isolate 1

KT308975 *Betula* *pubescens* var. *fragans* isolate 2

KT308976 *Betula* *pubescens* var. *pumila* isolate 2

KT308980 *Betula* *pubescens* var. *pumila* isolate 3

KT308981 *Betula* *pubescens* var. *pubescens* isolate 3

KT308982 *Betula* *pubescens* var. *pubescens* isolate 4

KT308983 *Betula* *pubescens* var. *litwinowii* isolate 2

MH014808 *Betula* *borysthenica*

MH178101 *Betula* *pubescens* var. *sibakademica*

MH238476 *Betula* *oycowiensis*

Position 234 C/U, var. “U”:

AJ783646 *Betula* *nigra*

AY352331 *Betula* *nigra*

AY761101 *Betula* *alnoides*

AY761124 *Betula* *nigra*

AY763114 *Betula* *alnoides*

KT308940 *Betula* *alnoides*

KT308944 *Betula* *luminifera*

KT308964 *Betula* *nigra*

KT308965 *Betula* *nigra*

Position 223 C/U, var. “U”:

AB243914 *Betula* *apoiensis* haplotype:ap14

Position 225 C/U, var. “U”:

AB243881 *Betula* *chichibuensis* haplotype:ch1

AB243882 *Betula* *chichibuensis* haplotype:ch2

AB243896 *Betula* *ovalifolia* haplotype:ov1

AB243908 *Betula* *apoiensis* haplotype:ap13

AB243909 *Betula* *apoiensis* haplotype:ap11

AB243910 *Betula* *apoiensis* haplotype:ap10

AB243913 *Betula* *apoiensis* haplotype:ap16

AB243913 *Betula* *apoiensis* haplotype:ap16

AB243915 *Betula* *apoiensis* haplotype:ap17

AY761103 *Betula* *calcicola*

AY761104 *Betula* *chichibuensis*

AY761107 *Betula* *delavayi*

KT308909 *Betula* *potaninii*

KT308910 *Betula* *potaninii*

KT308913 *Betula* *delavayi*

KT308914 *Betula* *calcicola*

KT308915 *Betula* *chichibuensis*

KT308916 *Betula* *chichibuensis*

KT308921 *Betula* *delavayi*

KT309022 *Betula* *ovalifolia*

KT309023 *Betula* *ovalifolia*
