# Supplementary material for: ITS secondary structure reconstruction to resolve taxonomy and phylogeny of the Betula L. genus
Source: PeerJ. 2021 Mar 23;9:e10889. doi: 10.7717/peerj.10889 (PMC7996101; doi:10.7717/peerj.10889)
Supplement: Supplemental Information 19 [file peerj-09-10889-s019.docx]

**Table S1.** The birch ITS sequences retrieved from NCBI GenBank used in the study.

| **Species** | **ITS marker (haplotype)** | **NCBI GenBank accession number** |
| --- | --- | --- |
| *B. albosinensis* Burkill | ITS | KT308954.1 |
|  | ITS | KT308924.1 |
|  | ITS | AY761099.1 |
| *B. albosinensis* var*. septentrionalis* C. K. Schneider | ITS | KT308947.1 |
| *B. alleghaniensis* Britton | ITS | KT308925.1 |
|  | ITS | AY761100.1 |
|  | ITS2 | MG235784.1 |
| *B. alnoides* Buch.-Ham. | ITS | AJ783641.1 |
|  | ITS | KT308940.1 |
|  | ITS2 | FJ011769.1 |
|  | ITS | AY763114.1 |
|  | ITS | AY761101.1 |
| *B. apoiensis* Nakai | ITS (ap4) | AB243899.1 |
|  | ITS (ap1) | AB243900.1 |
|  | ITS (ap2) | AB243901.1 |
|  | ITS (ap3) | AB243902.1 |
|  | ITS (ap6) | AB243903.1 |
|  | ITS (ap7) | AB243904.1 |
|  | ITS (ap8) | AB243905.1 |
|  | ITS (ap9) | AB243906.1 |
|  | ITS (ap5) | AB243907.1 |
|  | ITS (ap13) | AB243908.1 |
|  | ITS (ap11) | AB243909.1 |
|  | ITS (ap10) | AB243910.1 |
|  | ITS (ap12) | AB243911.1 |
|  | ITS (ap15) | AB243912.1 |
|  | ITS (ap16) | AB243913.1 |
|  | ITS (ap14) | AB243914.1 |
|  | ITS (ap17) | AB243915.1 |
|  | ITS | AY761102.1 |
| *B. ashburneri* McAll. & Rushforth | ITS | KT308952.1 |
|  | ITS | KT308953.1 |
|  | ITS | KT308961.1 |
| *B. bomiensis* P.C.Li | ITS | KT308911.1 |
|  | ITS | KT308912.1 |
| *B. browicziana* Güner | ITS | KT308968.1 |
| *B. calcicola* (W.W.Sm.) P.C.Li | ITS | KT308914.1 |
|  | ITS | AY761103.1 |
| *B. chichibuensis* Hara | ITS | AY761104.1 |
|  | ITS | AB243881.1 |
|  | ITS | AB243882.1 |
|  | ITS | KT308915.1 |
|  | ITS | KT308916.1 |
| *B. chinensis* Maxim. | ITS | KT308917.1 |
|  | ITS | KT308918.1 |
|  | ITS | AY761105.1 |
| *B. cordifolia* Regel | ITS | KT309015.1 |
|  | ITS | KT309016.1 |
|  | ITS2 | MG235525.1 |
| *B. corylifolia* Regel & Maxim. | ITS | AB243884.1 |
|  | ITS | AB243885.1 |
|  | ITS | KT308907.1 |
|  | ITS | KT308908.1 |
|  | ITS | AY761106.1 |
| *B. costata* Trautv. | ITS | KT308958.1 |
|  | ITS | AY352337.1 |
| *B. cylindrostachya* Wall. | ITS | KT308941.1 |
| *B. davurica* Pall. | ITS | AB243893.1 |
|  | ITS | AB243894.1 |
|  | ITS | KT308962.1 |
|  | ITS | KT308963.1 |
|  | ITS | FJ011770.1 |
|  | ITS | FJ011771.1 |
|  | ITS | FJ011772.1 |
|  | ITS | FJ011773.1 |
| *B. delavayi* Franch. | ITS | KT308921.1 |
|  | ITS2 | KT308922.1 |
|  | ITS | KT308913.1 |
|  | ITS | AY761107.1 |
| *B. ermanii* Cham. | ITS | AB243886.1 |
|  | ITS | AB243887.1 |
|  | ITS | AB243888.1 |
|  | ITS | AB243889.1 |
|  | ITS | KT308956.1 |
|  | ITS | KT308957.1 |
|  | ITS | AY761108.1 |
|  | ITS2 | LC382206.1 |
|  | ITS2 | LC382207.1 |
| *B. fargesii* ([Franch.](https://www.ipni.org/a/2869-1)) [P.C.Li](https://www.ipni.org/a/13777-1) | ITS | KT308906.1 |
| *B. fruticosa* Pall. | ITS | AY761109.1 |
| *B. glandulosa* Michx. | ITS2 | JN998975.1 |
|  | ITS2 | JN998976.1 |
|  | ITS2 | JN998977.1 |
|  | ITS2 | JN998978.1 |
|  | ITS | KT309017.1 |
|  | ITS | KT308995.1 |
|  | ITS2 | FJ011774.1 |
|  | ITS | AY761110.1 |
|  | ITS2 | MG236767.1 |
| *B. globispica* Shirai | ITS | AB243883.1 |
|  | ITS | KT308904.1 |
|  | ITS | KT308905.1 |
|  | ITS | AY761111.1 |
| *B. grossa* Siebold & Zucc. | ITS | AB243892.1 |
|  | ITS | KT308934.1 |
|  | ITS | KT308935.1 |
|  | ITS | AY761112.1 |
|  | ITS | AY761113.1 |
| *B. hainanensis* J.Zeng & B.Q.Ren & J.Y.Zhu & Z.D.Chen | ITS | KT308942.1 |
| *B. halophila* Ching ex P.C.Li | ITS | KT308967.1 |
| *B. humilis* Schrank | ITS | AJ783643.1 |
|  | ITS | KT309024.1 |
|  | ITS | KT309025.1 |
|  | ITS | KT309026.1 |
|  | ITS | AY761114.1 |
| *B. insignis* Franch. | ITS | AJ783645.1 |
|  | ITS | KT308927.1 |
|  | ITS | KT308928.1 |
|  | ITS | KT308929.1 |
| *B. kenaica* W.H.Evans | ITS2 | MG236820.1 |
|  | ITS2 | MG234627.1 |
| *B. lanata* (Regel) V.N.Vassil | ITS | KT308959.1 |
|  | ITS | KT308960.1 |
| *B. lenta* f. *uber* (Ashe) McAll. & Ashburner | ITS | KT308937.1 |
|  | ITS | KT308938.1 |
| *B. lenta* L. | ITS | KT308936.1 |
|  | ITS2 | FJ011775.1 |
|  | ITS | AY761115.1 |
|  | ITS | AY352330.1 |
|  | ITS2 | MG237175.1 |
|  | ITS2 | MG234564.1 |
| *B. luminifera* H.J.P.Winkl. | ITS2 | KT308939.1 |
|  | ITS | KT308943.1 |
|  | ITS | KT308944.1 |
|  | ITS | AY763113.1 |
|  | ITS2 | AY761116.1 |
|  | ITS2 | AY761117.1 |
| *B. maximovicziana* Regel | ITS | AB243890.1 |
|  | ITS | KT308945.1 |
|  | ITS | KT308946.1 |
|  | ITS | AY761118.1 |
| *B. medwediewii* Regel | ITS | KT308930.1 |
|  | ITS | KT308931.1 |
|  | ITS | AY761120.1 |
| *B. megrelica* Sosn. | ITS | KT308932.1 |
|  | ITS | KT308933.1 |
| *B. michauxii* Spach | ITS | KT308978.1 |
|  | ITS | AY761121.1 |
|  | ITS2 | MG234781.1 |
| *B. microphylla* Bunge | ITS | KT308984.1 |
| *B. middendorffii* Trautv. & C.A.Mey. | ITS | AB243895.1 |
|  | ITS | KT308986.1 |
| *B. minor* (Tuck.) Fernald | ITS | KT308985.1 |
|  | ITS2 | MG237810.1 |
| *B. murrayana* B.V.Barnes & Dancik | ITS | KT308926.1 |
| *B. nana* L. | ITS2 | KT960160.1 |
|  | ITS2 | KT960259.1 |
|  | ITS2 | KT960303.1 |
|  | ITS | KT309018.1 |
|  | ITS | KT309019.1 |
|  | ITS | KT309020.1 |
|  | ITS | AY761122.1 |
|  | ITS | AY352336.1 |
|  | ITS2 | MG237429.1 |
|  | ITS2 | MG235907.1 |
| *B. neoalaskana* Sarg. | ITS2 | AY761123.1 |
|  | ITS2 | MG235191.1 |
|  | ITS2 | MG237484.1 |
| *B. nigra* L. | ITS | AJ783646.1 |
|  | ITS | KT308964.1 |
|  | ITS | KT308965.1 |
|  | ITS | AY761124.1 |
|  | ITS | AY352331.1 |
| *B. obscura* Kotula ex Fiek | ITS | KT308993.1 |
| *B. occidentalis* Hook. | ITS | KT309027.1 |
|  | ITS | KT309028.1 |
|  | ITS | AY761125.1 |
|  | ITS | DQ397523.1 |
|  | ITS2 | MG237590.1 |
|  | ITS2 | MG237283.1 |
|  | ITS2 | MG236556.1 |
| *B. ovalifolia* Rupr. | ITS2 | AB243896.1 |
|  | ITS | AB243897.1 |
|  | ITS | AB243898.1 |
|  | ITS | KT309022.1 |
|  | ITS | KT309023.1 |
|  | ITS2 | LC382204.1 |
|  | ITS2 | LC382205.1 |
| *B. papyrifera* Marshall | ITS | KT309011.1 |
|  | ITS | KT309012.1 |
|  | ITS | KT309013.1 |
|  | ITS2 | FJ011776.1 |
|  | ITS | AY761126.1 |
|  | ITS | AF432067.1 |
|  | ITS2 | MG236939.1 |
| *B. papyrifera* var*. commutata* (Regel) Fernald | ITS | KT309014.1 |
| *B. pendula* Roth | ITS | AJ006445.1 |
|  | ITS | AM503889.2 |
|  | ITS | KT308990.1 |
|  | ITS | JN247411.1 |
|  | ITS | FJ011777.1 |
|  | ITS | AY761127.1 |
|  | ITS | AY352332.1 |
| *B. pendula* subsp. *pendula* | ITS | KT308998.1 |
|  | ITS | KT309000.1 |
|  | ITS | KT309002.1 |
|  | ITS | KT309001.1 |
|  | ITS | KT309006.1 |
|  | ITS | KT309007.1 |
| *B. pendula* subsp. *mandshurica* (Regel) Ashburner & McAll. | ITS | KT308991.1 |
|  | ITS | KT309005.1 |
|  | ITS | KT308996.1 |
|  | ITS | KT309008.1 |
|  | ITS | KT308999.1 |
| *B. pendula* subsp. *szechuanica* (C.K.Schneid.) Ashburner & McAll. | ITS | KT308997.1 |
|  | ITS | KT309003.1 |
|  | ITS | KT309004.1 |
| *B. platyphylla* Sukaczev | ITS | AB243891.1 |
|  | ITS | FJ011778.1 |
|  | ITS | AY761128.1 |
| *B. platyphylla* var*. japonica* (Miq.) Hara | ITS2 | LC382208.1 |
|  | ITS2 | LC382209.1 |
| *B. populifolia* Marshall | ITS | AJ783644.1 |
|  | ITS | KT309009.1 |
|  | ITS | KT309010.1 |
|  | ITS | KT308994.1 |
|  | ITS | AY761129.1 |
|  | ITS2 | MG237745.1 |
| *B. potaninii* Batalin | ITS | KT308909.1 |
|  | ITS | KT308910.1 |
| *B. pubescens* Ehrh. | ITS | AY761130.1 |
| *B. pubescens* var. *pubescens* | ITS | KT308969.1 |
|  | ITS | KT308970.1 |
|  | ITS | KT308981.1 |
|  | ITS | KT308982.1 |
| *B. pubescens* subsp*. celtiberica* (Rothm. & Vasc.) Rivas.Martinez | ITS | KT308977.1 |
|  | ITS | KT308972.1 |
| *B. pubescens* var. *fragrans* Ashburner & McAll*.* | ITS | KT308974.1 |
|  | ITS | KT308975.1 |
| *B. pubescens* var. *litwinowii* (Doluch.) Ashburner & McAll. | ITS | KT308971.1 |
|  | ITS | KT308983.1 |
| *B. pubescens* var. *pumila* (L.) Govaerts | ITS | KT308973.1 |
|  | ITS | KT308976.1 |
|  | ITS | KT308980.1 |
| *B. pumila* L. | ITS | AJ783642.1 |
|  | ITS2 | JN998979.1 |
|  | ITS | KT309021.1 |
|  | ITS | AY761131.1 |
| *B. raddeana* Trautv. | ITS | KT308966.1 |
|  | ITS | AY761132.1 |
| *B. schmidtii* Regel | ITS | AB243880.1 |
|  | ITS | KT308919.1 |
|  | ITS | KT308920.1 |
|  | ITS | FJ011779.1 |
|  | ITS | AY761133.1 |
| *B. tianschanica* Rupr. | ITS | KT308989.1 |
| *B. turkestanica* Litv. | ITS | KT308992.1 |
| *B. utilis* D.Don | ITS | KT308948.1 |
|  | ITS | KT308949.1 |
|  | ITS | FJ011780.1 |
|  | ITS | AY761134.1 |
| *B. utilis* subsp*. jacquemonti* (Spach) Ashburner & McAll. | ITS | KT308951.1 |
| *B. utilis* var*. occidentalis* (Kitam.) Ashburner & A.D.Schill. | ITS | KT308950.1 |
|  | ITS | KT308923.1 |
| *B. utilis* var*. prattii* Burkill | ITS | KT308955.1 |
| *B.* × *caerulea* Blanch. | ITS | KT308987.1 |
| *B.* × *utahensis* Britton | ITS | KT308979.1 |
| *B. ovalifolia* × *B. ermanii* | ITS2 | LC382024.1 |
|  | ITS2 | LC382025.1 |
