# Supplementary material for: ITS secondary structure reconstruction to resolve taxonomy and phylogeny of the Betula L. genus
Source: PeerJ. 2021 Mar 23;9:e10889. doi: 10.7717/peerj.10889 (PMC7996101; doi:10.7717/peerj.10889)
Supplement: Supplemental Information 20 [file peerj-09-10889-s020.docx]

**Table S2.** The birch ITS sequenced by the authors and used in the study.

| **Species** | **Specimen voucher, ID*** | **ITS marker** | **NCBI GenBank accession number** |
| --- | --- | --- | --- |
| *Betula borysthenica* Klokov | KW000006422  KW000006415  (holotype) | ITS1  ITS2 | MH238479  MH014808 |
| *B. klokovii* Zaverucha | KW000006415  KW000006422  (holotype) | ITS1  ITS2 | MH238480  MH014819 |
| *B. oycowiensis* Besser | LW032763 | ITS1 | MH238476 |
| *B. pubescens* var*. sibakademica* (Baranov) Kuzeneva (≡*B. pubescens* f*. sibakademica* (Baranov) Tarieiev)** | LE01041130 (type) (contains 2 different samples on 1 sheet) | ITS1  ITS2 | MH178101 (R)  MH231206 (L) |
|  | KW128012 | ITS1  ITS2 | MH238471  MH014819 |
|  | KW128024 | ITS | MH178102  MH042911 |
|  | 031473 | ITS1 | MH238472  MH238473 |
|  | LWKS031322 | ITS1  ITS2 | MH231212  MH042915 |
| *B. kotulae* Zaverucha (= *B. pendula* Roth var. *obscura* (Kotula ex Fiek) Olšavská ≡ *B. pendula* Roth f. *obscura* (Kotula ex Fiek) Tarieiev** | KW006426 (holotype) | ITS1  ITS2 | MH231207  MH042917 |
|  | KW008349 | ITS | MH178103  MH042912 |
|  | KW06427 | ITS | MH178104 |
|  | KW128013 | ITS | MH178105  MH042913 |
|  | KW128014 | ITS | MH178106 |
|  | KW128016 | ITS | MH178107 |
|  | KW128018 | ITS | MH178108 |
|  | KW128019 | ITS1 | MH231208 |
|  | KW128020 | ITS1 | MH231209 |
|  | KW128022 | ITS | MH178109 |
|  | KW128023 | ITS1  ITS2 | MH231210  MH300135 |
|  | 031472 | ITS1 | MH238474 |
|  | LW032758 | ITS1 | MH238475 |
|  | LW006898 | ITS1  ITS2 | MH231213  MH042916 |
|  | LWS27022 | ITS1 | MH238478 |
|  | LWS27026 | ITS1 | MH238477 |
|  | live specimen | ITS1  ITS2 | MH231214  MH042918 |
| *B. atrata* Domin | 031470 | ITS1  ITS2 | MH231211  MH042914 |
| *B. pubescens* subsp. *carpatica* (Waldst. & Kit. ex Willd.) Asch. & Graebn. | live specimen | ITS | MH014809 |

*KW - M.G. Kholodny Institute of Botany, National Academy of Sciences, Kyiv, Ukraine; LW - Ivan Franko National University, Lviv, Ukraine; LE - Komarov Botanical Institute, Russian Academy of Sciences, Saint Petersburg, Russian Federation; numbers without acronym referred to former herbarium of Taras Shevchenko National University of Kyiv

**These sequences were used to study the taxonomy of dark-barked birches (Tarieiev et al. 2019)
